# Supplementary material for: The geologic history of marine dissolved organic carbon from iron oxides
Source: Nature. 2025 Aug 13;644(8078):945–51. doi: 10.1038/s41586-025-09383-3 (PMC12390840; doi:10.1038/s41586-025-09383-3)
Supplement: Supplementary file 1 — Supplementary Discussion, Supplementary Tables, Supplementary Figures and Supplementary References. [file 41586_2025_9383_MOESM1_ESM.pdf]

nature portfolio

https://doi.org/10.1038/s41586-025-09383-3

Supplementary information

The geologic history of marine dissolved organic carbon from iron oxides

In the format provided by the authors and unedited

Supplementary Discussion

Table of contents:

1 General approach S-3
2 Building Fe-OC carbon-loading and ^{13}C fractionation response curves S-3
2.1 Molecular and functional characteristics of experimental DOC S-3
2.2 Effects of co-precipitation procedural manipulations on Fe-OC carbon-loading and ^{13}C fractionation response curves S-4
3 Iron ooid formation mechanism and timescale. S-12
3.1 Iron ooid formation mechanism S-12
3.2 Iron ooid formation timescale and accretion rate S-14
4 Geologic descriptions of sampled formations S-15
4.1 Panarea Island, Italy (modern) S-15
4.2 Mahengentang Island, Indonesia (modern) S-16
4.3 Lisakovsk Channel Iron Deposit, Chegan Fm, Kazakhstan (∼28 Ma) . S-16
4.4 Bakchar Horizon, Lyulinvor Fm, Russia (56-59 Ma) S-17
4.5 Hatira Fm, Israel (100-113 Ma) S-17
4.6 Hidra Fm, Israel (100-113 Ma) S-18
4.7 Arroyofrío Bed, Chelva and Yatova Fms, Spain (157-166 Ma) S-18
4.8 Oolithe Ferrugineuse de Villers Fm, France (162-163 Ma) S-19
4.9 Ifenthal, Wutach, "Humphriesioolith", and "Parkinsoni-Württembergica Schichten" (informal) Fms, Switzerland (∼164-169 Ma) S-19
4.10 Blaukalk Member, Wedelsandstein Fm, Germany (168-170 Ma) S-20
4.11 Oolithe Ferrugineuse de Bayeux Fm, France (168-170 Ma) S-20
4.12 Presles Fm, Belgium (372-383 Ma) S-20
4.13 Westmoreland and Kirkland Fms, Clinton Gp, USA (∼435-440 Ma) . . S-21
4.14 Skovde Limestone, Gullhogen Fm, Sweden (462-463 Ma) S-21
4.15 Aseri Fm, Estonia (463-464 Ma) S-22
4.16 Kunda Oolite Bed, Sillaoru Fm, Estonia and Russia (464-467 Ma) . . . S-22
4.17 Šárka Fm, Czechia (∼470 Ma) S-23
4.18 Bliss Fm, USA (491-493 Ma) S-23
4.19 Galeros Fm, Chuar Gp, USA (∼750 Ma) S-24
4.20 McClure Fm, Katherine Gp, Canada (∼850 Ma) S-25
4.21 Sherwin Ironstone, Sherwin Fm, Roper Gp, Australia (∼1300 Ma) . . . S-26
4.22 Chuanlinggou Fm, Changcheng Gp, China (∼1650 Ma) S-26
5 Geologic sample screening, binning, and intra-period variability S-27
5.1 Sample screening S-27
5.2 Correlation with (paleo)latitude S-28
5.3 Intra-period spatial variability S-28
5.4 Time-binning S-30
6 Estimating DOC concentrations and $\delta^{13}C$ values from iron ooid Fe-OC S-30
6.1 Mathematical derivation S-31
6.2 Possible $f^i(t)$ scenarios S-33
6.3 Monte Carlo solution S-35
6.4 Sensitivity tests and validity of assumptions S-36
7 Interpreting organic $\delta^{13}C$ trends in multiple geologic materials S-40
8 Supplementary Data Tables S-41

9 Supplementary Data Figures S-46

1 General approach

Reconstructing marine DOC concentration in the geologic past is a substantial challenge in Earth Science, mainly due to the lack of direct observational constraints. This work aims to fill this knowledge gap by leveraging results from soil science and environmental chemistry. Two primary observations underpin our approach and guide our development and calibration of a proxy for past DOC concentration: (i) iron (oxyhydr)oxides entrap and preserve DOC[32,34,125], and (ii) co-precipitated OC content depends on the initial solution DOC/Fe(III) ratio[35]. We specifically apply this proxy to co-precipitated mineral-bound OC in iron (oxyhydr)oxide ooids due to their unique sedimentological requirements, which ensure an in-situ marine production signal that remains uninfluenced by detrital inputs. All geologic ooid samples used here underwent a screening protocol using petrographic and chemical characterization to constrain and assess their formation and preservation. When combined with laboratory synthesis experiments to quantify and calibrate iron-bound OC loading curves and ^{13}C fractionation factors, our geologic records of iron ooid OC content and δ^{13}C values represent the first (to our knowledge) Earth-history reconstructions of marine DOC concentration and carbon-isotope composition. Each aspect of this process is detailed in subsequent sections.

2 Building Fe-OC carbon-loading and ^{13}C fractionation response curves

2.1 Molecular and functional characteristics of experimental DOC

Prior to performing any synthesis experiments, we first characterized the molecular compositions of our three DOC sources—modern-marine (M-DOC), cyanobacterial (C-DOC), and fulvic acid (FA)—using absorbance and fluorescence spectroscopy to discern any compositional influence on resulting Fe-OC loadings and/or ^{13}C fractionation factors (Methods). Importantly, if Fe-OC signals are intrinsically linked to DOC structure, then any observed trends through geologic time could reflect a shift in molecular composition in addition to DOC concentration and δ^{13}C values. We thus incorporate fluorescence spectroscopy-based molecular insight when interpreting the Fe-OC signals of our precipitation experiments and iron oxides ooid record.

This technique leverages the fact that fluorescent dissolved organic matter (FDOM) absorbs and re-emits light within the UV to visible range and that the exact wavelengths involved depend on its molecular size and composition. Insight into the composition of complex organic matter mixtures can therefore be gained by spanning several excitation and emission wavelengths to build an Excitation-Emission Matrix (EEM) of fluorescence intensity. Resulting EEMs display unique peaks (termed “A”, “B”, “C”, “M”, and “T”) that relate to specific functional groups and thus organic matter origin, behavior, and biogeochemical dynamics[126]. Absorbance and EEMs results are often further distilled into several diagnostic metrics, including the fluorescence index (FI), specific UV absorbance at $254\,\mathrm{nm}$ (SUVA$_{254}$), several spectral slopes ($S_{275-295}$ and $S_{350-400}$), and the slope ratio (S$_R$; see Methods for definitions). Several studies have correlated these metrics with independently derived estimates of DOC molecular size and composition, particularly aromaticity[40,65–67].

Absorbance- and EEMs-derived metrics reveal variability in the molecular size and composition of each DOC source considered here (Fig. S1; Table S1). Briefly, each DOC source composition can be summarized as follows:

i. M-DOC is primarily composed of non-aromatic compounds with roughly proportional contributions of low- and high-molecular weight DOC. This sample displays SUVA$_{254}$ = 1.30 and FI = 1.95; both values indicate ≈ 10 to $15\,\%$ aromaticity according to published global regressions of SUVA$_{254}$ and FI vs. ^{13}C nuclear magnetic resonance (NMR)-derived

aromaticity measurements[66,67]. M-DOC is further described by S_R = 1.29, similar to values found in nutrient-rich coastal-ocean waters that contain a mixture of low- and high-molecular weight DOC and are relatively un-impacted by processing due to heterotrophic respiration and/or photodegradation[40]. Such similarity is consistent with the fact that our microcosm cultures were designed to imitate coastal marine ecosystems dominated by eukaryotic primary producers.

ii. C-DOC contains significantly more aromatic compounds and low-molecular weight DOC as compared to M-DOC. Specifically, this sample displays $SUVA_{254}$ = 4.05 and FI = 3.06; while the latter value is outside of the regression range reported by Ref.[66], this $SUVA_{254}$ value suggests $\approx$30 % aromaticity according to Ref.[67]. Still, this $SUVA_{254}$ value is higher than those from most natural samples and should thus be interpreted with caution. C-DOC is further described by S_R = 2.20, similar to previously reported values for surface DOC in the more open-ocean, cyanobacterially dominated continental shelves (although still significantly lower than the ultra-oligotrophic Sargasso Sea)[40]. Higher S_R values compared to M-DOC suggest a higher contribution by low-molecular weight DOC in C-DOC, possibly due to increased photo-bleaching of this sample[40]. Although the exact culturing methods of this commercially purchased sample are unknown to us, these results are broadly consistent with observations in more cyanobacterially dominated regions of the ocean.

iii. FA is primarily composed of non-aromatic material and displays a higher prevalence of high- relative to low-molecular weight compounds. In particular, this sample displays $SUVA_{254}$ = 1.65 and FI = 2.23; both values are similar to, although slightly higher than, those observed for M-DOC. Following Ref.[67], this $SUVA_{254}$ value corresponds to $\approx$ 15 to 20 % aromaticity (like for C-DOC, this FI value is outside of the regression range reported by Ref.[66]). Furthermore, FA displays a lowest slope ratio of any sample measured here, with S_R = 0.67, indicating the importance of unprocessed high-molecular weight DOC. This S_R value is identical to those reported previously for humic terrestrial systems such as swamps, as expected given that DOC in such environments is dominated by humic and fulivc acids[40].

2.2 Effects of co-precipitation procedural manipulations on Fe-OC carbon-loading and ^{13}C fractionation response curves

Before utilizing our iron ooid Fe-OC record as a proxy for marine DOC concentration and isotope composition, it is first crucial to ensure that our interpretations are not impacted by biases that could arise from our chosen calibration curve experimental conditions and/or post-synthesis processing steps. We therefore devised a series of experimental tests to determine the sensitivity of Fe-OC loadings and isotope compositions to environmental conditions (i.e., solution temperature, pH, DOC concentrations, Fe concentrations, dissolved silica concentrations), mineral precipitation conditions (i.e., experiment duration, iron source, and timing of DOC addition), and sample preparation protocols (i.e., rinsing, ferrihydrite removal, adsorbed OC removal). Synthesized precipitates in all tests were confirmed to be mineralogically pure goethite (Figs. S2-S4) or hematite (Figs. S5-S7); results from all tests are reported in the Supplementary Data. Each effect is summarized as follows:

Effect of solution temperature. To assess the effect of temperature on Fe-OC signals, we conducted goethite synthesis experiments under alkaline conditions (pH = 11.4 to 11.5) at tem-

peratures ranging from $4\,°C$ to $95\,°C$ using FA as a representative DOC source. All experiments for this test were conducted at least in triplicate with $FeCl_3$ as the Fe(III) source and DOC addition after ferrihydrite precipitation; they were aged for 7 to 14 days and span a DOC/Fe(III) range of ≈ 2 orders of magnitude.

Results suggest a small impact of solution temperature on Fe-OC loadings (Fig. S8). For example, Fe-OC loadings for the "−Fh/PC −ads. OC" fraction with DOC/Fe(III) = 0.017 to 0.019 these loadings are are $0.11 \pm 0.01\,\%$ at $25\,°C$ ($n = 3$) and $0.17 \pm 0.01\,\%$ at $70\,°C$ ($n = 6$); similarly, with DOC/Fe(III) = 0.29 are $0.55 \pm 0.02\,\%$ at $4\,°C$ ($n = 6$), $0.49 \pm 0.01\,\%$ at $50\,°C$ ($n = 6$), and $0.63 \pm 0.11\,\%$ at $95\,°C$ ($n = 6$). Co-precipitated OC content may therefore be slightly elevated at high temperature under these conditions for low DOC/Fe(III) ratios, but this difference becomes statistically insignificant at higher DOC/Fe(III) ratios. The consistency in Fe-OC loadings across a broad temperature range from 4 to $95\,°C$ reinforces the notion that Fe-OC loading is robust to temperature variations under the studied conditions.

In contrast, the temperature effect on ^{13}C composition exhibits more nuanced behavior. Observed ^{13}C offsets (defined as $\Delta^{13}C = \delta^{13}C_{DOC} - \delta^{13}C_{Fe-OC}$) remain relatively consistent from $4\,°C$ to $70\,°C$. For example, for the "−Fh/PC −ads. OC" fraction with DOC/Fe(III) ratios of 0.017 to 0.019, the ^{13}C offsets are $9.7 \pm 0.2\,‰$ at $25\,°C$ ($n = 3$) and $12.1 \pm 0.2\,‰$ at $70\,°C$ ($n = 6$) while at DOC/Fe(III) = 0.29, the offsets are $-0.5 \pm 0.1\,‰$ at $4\,°C$ ($n = 6$), $0.8 \pm 0.3\,‰$ at $50\,°C$ ($n = 6$), and $8.8 \pm 0.2\,‰$ at $95\,°C$ ($n = 6$). A notable deviation is observed at $95\,°C$. At this temperature, $\Delta^{13}C$ value are significantly larger than for lower temperatures—particularly when DOC/Fe(III) $\geq$ 0.25—indicating larger ^{13}C fractionation. This suggests a divergence from the temperature insensitivity observed at lower temperatures and points to a possible thermal threshold in the Fe-OC system's response to isotopic fractionation. This threshold may relate to thermal degradation of substrate organic compounds (e.g., decarboxylation), which would lead to ^{13}C-depleted residual substrate either due to kinetic fractionation or site-specific isotopic differences (e.g., if carboxyl groups are enriched in ^{13}C); further research is needed to confirm the fractionation mechanism at higher temperatures.

While temperature does appear to exert a small control on Fe-OC signals—particularly $\delta^{13}C$—recent studies have shown that global ocean temperatures likely remained relatively constant over the past 2 billion years of Earth's history[42]. Nevertheless, in-situ temperatures at the site of iron ooid formation may be elevated relative to the global ocean (e.g., due to upward diffusion of Fe-rich hydrothermal fluids at the sediment-water interface); for example, modern iron ooid formation sites have been shown to reach temperatures of $\approx 40\,°C$ (c.f., $\approx 15\,°C$ for typical continental shelf bottom waters)[43]. Importantly, although elevated, ooid formation sites never reach in-situ temperatures as high as $95\,°C$. Thus, any potential ^{13}C bias within the observed temperature range is less than the observed variability between geologic formations of a given age (see "Intra-period spatial variability", below). We therefore ignore any temperature effect in our geologic record, but we refrain from interpreting temporal variability smaller than $\approx 0.05\,\%$ for Fe-OC loadings and $\approx 3\,‰$ for $\delta^{13}C$, as these could be driven by local temperature variability.

Effect of solution pH. Like for temperature, we assessed the effect of pH on Fe-OC signals by conducting goethite synthesis experiments under acidic (pH = 1.8), circumneutral (pH = 5-7), and alkaline (pH = 11.5) conditions using FA as a representative DOC source. All experiments for this test were conducted at least in triplicate at $25\,°C$ (circumneutral and alkaline) or 50 °C (acidic; room temperature acidic synthesis are not possible using our protocol) with $FeCl_3$ (circumneutral and alkaline) or $Fe(NO_3)_3$ (acidic) as the Fe(III) source and DOC addition after ferrihydrite precipitation; they were aged for 5 to 10 days and span a DOC/Fe(III) range of ≈ 2

orders of magnitude.

Results suggest a small impact of solution pH on Fe-OC loading within the acidic to circumneutral range, but a larger offset for alkaline solutions (Fig. S9). For example, Fe-OC loadings for the "$-$Fh/PC $-$ads. OC" fraction with DOC/Fe(III) = 0.018 to 0.039 are $0.35 \pm 0.02\,\%$ for acidic pH ($n = 6$), $0.27 \pm 0.01\,\%$ for circumneutral pH ($n = 3$), and $0.11 \pm 0.01\,\%$ for alkaline pH ($n = 3$). Lower Fe-OC loadings for alkaline solutions may be due to the lower DOC/Fe(III) ratio for this experiment relative to acidic and circumneutral experiments (0.018 vs. 0.030 to 0.039). However, this offset persists at higher DOC/Fe(III) ratios; for example, Fe-OC loadings for the "$-$Fh/PC $-$ads. OC" fraction with DOC/Fe(III) = 0.157 to 0.167 are $1.23 \pm 0.07\,\%$ for circumneutral pH ($n = 3$) and $0.28 \pm 0.01\,\%$ for alkaline pH ($n = 3$) (acidic experiments were not performed at this DOC/Fe(III) ratio). Iron ooids precipitated in alkaline solutions under these conditions therefore yield lower Fe-OC loadings than those precipitated in acidic and circumneutral solutions.

Similarly, solution pH exerts a negligible control on Fe-OC ^{13}C compositions within the acidic to circumneutral range, but a larger offset is observed in alkaline solutions, especially for lower DOC/Fe(III) ratios. For example, Fe-OC ^{13}C offsets for the "$-$Fh/PC $-$ads. OC" fraction with DOC/Fe(III) = 0.018 to 0.039 are $-0.8 \pm 0.3\,‰$ for acidic pH ($n = 6$), $0.9 \pm 0.2\,‰$ for circumneutral pH ($n = 3$), and $9.7 \pm 0.2\,‰$ for alkaline pH ($n = 3$). However, this offset for alkaline solutions largely disappears at higher DOC/Fe(III) ratios; for example, Fe-OC ^{13}C offsets for the "$-$Fh/PC $-$ads. OC" fraction with DOC/Fe(III) = 0.157 to 0.167 are $0.0 \pm 0.1\,‰$ for circumneutral pH ($n = 3$) and $1.2 \pm 0.1\,\%$ for alkaline pH ($n = 3$) (acidic experiments were not performed at this DOC/Fe(III) ratio).

Thus, solution pH could exert a non-negligible control on Fe-OC loadings and δ^{13}C values only if DOC/Fe(III) ratios are low and solutions are highly alkaline. However, recent studies have shown that seawater pH has remained between ≈ 6.5 and 8.0 throughout Earth's history[127], similar to our circumneutral pH experiments. Furthermore, any local pH variability at the site of iron ooid formation would likely push solutions toward acidic, rather than alkaline, conditions (e.g., due to upward diffusion of acidic, Fe-rich hydrothermal fluids at the sediment-water interface). Given that our acidic and circumneutral experiments exhibit similar Fe-OC loadings and ^{13}C compositions, we conclude that pH-dependent isotope effects are likely small and we ignore any potential pH effect when interpreting our geologic record.

Effect of adjusting DOC concentrations vs. Fe concentrations to manipulate DOC/Fe(III). To assess how initial solution absolute DOC and Fe(III) concentrations impact resulting Fe-OC signals, we conducted several experiments in which we varied either DOC or Fe(III) concentrations while holding the other constant. For goethite, this included experiments at $25\,°\mathrm{C}$ and circumneutral conditions (pH = 5-7) using $Fe(Cl)_3$ as an iron source and M-DOC, C-DOC, or FA as a DOC source. For hematite, this included experiments at $50\,°\mathrm{C}$ and circumneutral conditions (pH = 8) using $Fe(Cl)_3$ as an iron source and M-DOC or C-DOC as a DOC source. All experiments were performed at least in triplicate and allowed to ripen for 5 to 120 days.

Results for all conditions show that Fe-OC loadings and ^{13}C compositions depend only on DOC/Fe(III) ratio and not on absolute concentrations (Figs. S10, S11). For example, for the "$-$Fh/PC $-$ads. OC" fraction of goethite with FA, Fe-OC loadings were $0.27 \pm 0.01\,\%$ and Δ^{13}C values were $0.9 \pm 0.2\,‰$ when precipitated with concentrations of [Fe(III)] = $396.0\,\mathrm{mM}$ and [DOC] = $15.5\,\mathrm{mM}$ ($n = 3$), whereas Fe-OC loadings were $0.23 \pm 0.03\,\%$ and Δ^{13}C values were $2.8 \pm 0.2\,‰$ when precipitated with concentrations of [Fe(III)] = $99.0\,\mathrm{mM}$ and [DOC] = $3.9\,\mathrm{mM}$ ($n = 5$); DOC/Fe(III) ratios in both experiments were 0.039. Despite a four-fold difference in absolute Fe(III) and DOC concentrations, both experiments yield nearly identical

Fe-OC loadings and Δ^{13}C values. Similar results are observed for both mineralogies and all DOC sources at all tested DOC/Fe(III) ratios (Figs. S10, S11), providing strong evidence that it is the initial DOC/Fe(III) ratio—and not absolute concentrations—that control resulting Fe-OC signals. We therefore combine experiments performed at different Fe(III) concentrations when calculating our final Fe-OC calibration curves (see "Final Fe-OC calibration curves used for geologic reconstructions", below), and we discuss changing DOC/Fe(III) ratios through time when interpreting our geologic record.

Effect of synthesis duration. To discern the impact of synthesis duration on resulting Fe-OC signals, we conducted two sets of experiments in which we aged precipitates formed under the same conditions for varying amounts of time. For goethite, this included experiments performed at $25\,^{\circ}\mathrm{C}$ under circumneutral conditions (pH = 5-7) using FA as a DOC source, $FeCl_3$ as the Fe(III) source, and DOC addition after ferrihydrite precipitation; these were aged for 10 to 120 days and span a DOC/Fe(III) range of $\approx$ 3 orders of magnitude. For hematite, this included experiments performed at $50\,^{\circ}\mathrm{C}$ under circumneutral conditions (pH = 8) using M-DOC as a DOC source, $FeCl_3$ as the Fe(III) source, and DOC addition after ferrihydrite precipitation; these were aged for 5 to 30 days and span a DOC/Fe(III) range of $\approx$ 3 orders of magnitude.

Within the time range considered here, results for both sets of experiments indicate that synthesis duration exerts a negligible control on resulting Fe-OC signals (Fig. S12). Rather, Fe-OC loadings and isotope compositions appear temporally invariant. For example, for the "$-$Fh/PC $-$ads. OC" fraction for goethite with a ratio of DOC/Fe(III) = 0.039, Fe-OC loadings were $0.27 \pm 0.01\,\%$ and Δ^{13}C values were $0.9 \pm 0.2\,‰$ after 10 days ($n = 3$), whereas Fe-OC loadings were $0.23 \pm 0.02\,\%$ and Δ^{13}C values were $2.8 \pm 0.2\,‰$ after 120 days ($n = 10$). Similarly, for the "$-$Fh/PC $-$ads. OC" fraction for hematite with a ratio of DOC/Fe(III) = 0.018 to 0.020, Fe-OC loadings were $0.13 \pm 0.01\,\%$ and Δ^{13}C values were $-4.7 \pm 0.1\,‰$ after 5 days ($n = 3$), whereas Fe-OC loadings were $0.14 \pm 0.01\,\%$ and Δ^{13}C values were $-6.3 \pm 0.2\,‰$ after 30 days ($n = 10$). Similar results are observed for other DOC/Fe(III) ratios.

The chemical mechanism that controls co-precipitated Fe-OC signals likely operates during the transformation from poorly crystalline precursors (i.e., ferrihydrite) to crystalline phases (i.e., goethite or hematite) through a dissolution and re-precipitation process[128]. Our experimental results strongly indicate that this process is complete after ≈ 5 days at environmentally relevant temperatures and pH conditions, and that continued aging during ooid formation has little impact on Fe-OC signals (however, diagenetic alteration could still alter preserved signals; see discussion on model assumption (ii), below). We therefore apply our experimental Fe-OC loading and isotope fractionation calibration curves to natural iron ooids, which form over centuries to millennia (see "Iron ooid formation timescale and accretion rate", below).

Effect of Fe(III) source. We assessed the impact of experimental Fe(III) source on Fe-OC signals by conducting goethite synthesis experiments under alkaline (pH = 11.5) conditions using either $FeCl_3$ or $Fe(NO_3)_3$ as the Fe(III) source and FA as the DOC source. All experiments were performed at least in triplicate at $70\,^{\circ}\mathrm{C}$ with DOC addition after ferrihydrite precipitation; they were aged for 7 to 10 days and span a DOC/Fe(III) range of ≈ 2 orders of magnitude. Results indicate that Fe(III) source imparts a small but non-zero control on Fe-OC loadings but a negligible control on Δ^{13}C values (Fig. S13). For example, for the "$-$Fh/PC $-$ads. OC" fraction with a ratio of DOC/Fe(III) = 0.017 to 0.019, Fe-OC loadings were $0.17 \pm 0.01\,\%$ and Δ^{13}C values were $12.1 \pm 0.2\,‰$ with $FeCl_3$ as the Fe(III) source ($n = 6$), whereas Fe-OC loadings were $0.23 \pm 0.01\,\%$ and Δ^{13}C values were $11.4 \pm 0.2\,‰$ with $Fe(NO_3)_3$ as the Fe(III) source.

Observed offsets in Fe-OC loadings could result from differences in competitive interactions between DOC and various anions within the crystal lattice during crystal growth; if true, such offsets would thus be linked to differences in solution anion composition (e.g., Cl^- vs. NO_3^-). Here, we treat our $Fe(Cl)_3$ experiments as more representative of seawater conditions since these were conducted with Cl^- concentrations of $150\,\mathrm{mM}$, approaching the concentration of natural seawater. In contrast, seawater NO_3^- concentrations have likely remained several orders of magnitude lower than those used in our experiments throughout Earth's history. Nevertheless, Fe-OC loading differences of $\approx 0.05\,\%$ between Fe(III) sources and thus solution anion compositions are smaller than the typical observed variability between geologic formations of a given age (see "Intra-period spatial variability", below). We therefore ignore any potential Fe(III) source and/or anion composition effect in our geologic record, but we refrain from interpreting temporal variability smaller than $\approx 0.05\,\%$ for Fe-OC loadings, as these could be driven by solution composition.

Effect of parent solution silica concentration. Given that ancient oceans were likely more silica-rich than today—especially during the Archean and early Proterozoic Eons[37,38]—we conducted hematite synthesis experiments across a range of silica concentrations that represent proposed Precambrian ocean conditions to investigate the influence of silica on Fe-OC loadings and ^{13}C fractionation (i.e., $0.53\,\mathrm{mM}$, $1.06\,\mathrm{mM}$, and $2.13\,\mathrm{mM}$ silica). All experiments were performed at $50\,^{\circ}\mathrm{C}$ under circumneutral pH conditions using $FeCl_3$ as the Fe(III) source and C-DOC as the DOC source; DOC and silica were added after ferrihydrite precipitation, and samples were aged for 14 days. Because the initial C-DOC stock was completely consumed during our experiments, material used for these experiments was extracted separately from that used for all other experiments following identical protocols (see Methods).

Results indicate that increasing silica concentrations impart a small but non-negligible influence on Fe-OC loadings and ^{13}C fractionations relative to silica-free experiments (Fig. S14). For example, at a DOC/Fe(III) range of 0.028-0.031, Fe-OC loadings were $0.19 \pm 0.01\,\%$ with $\Delta^{13}C$ values of $-7.57 \pm 1.93\,‰$ at $0.53\,\mathrm{mM}$ SiO_2 ($n = 6$), $0.21 \pm 0.01\,\%$ with $\Delta^{13}C$ values of $-6.77 \pm 2.43\,‰$ at $1.06\,\mathrm{mM}$ SiO_2 ($n = 6$), and $0.19 \pm 0.02\,\%$ with $\Delta^{13}C$ values of $-6.61 \pm 2.24\,‰$ at $2.13\,\mathrm{mM}$ SiO_2 ($n = 6$). All results for silica-containing samples are thus statistically identical regardless of silica concentration. In comparison, silica-free experiments with DOC/Fe(III) of 0.031 exhibited Fe-OC loadings of $0.13 \pm 0.03\,\%$ and $\Delta^{13}C$ values of $-12.65 \pm 1.68\,‰$ (n=6). While the effect of silica on Fe-OC loadings and ^{13}C fractionation of hematite suggests that the presence of silica slightly enhances OC loading and results in slightly heavier $\Delta^{13}C$ values for a given DOC/Fe(III) ratio, the overall impact remains minor and observed trends with increasing DOC/Fe(III) remain consistent across all experimental conditions. Nevertheless, the results observed here imply that DOC concentrations and $\delta^{13}C$ values reconstructed using silica-free calibration curves should both be regarded as maximum values.

Effect of DOC addition timing. We tested the effect of DOC addition timing since crystalline phases to not precipitate directly from solution but rather represent the product of poorly crystalline precursor transformation through dissolution and re-precipitation reactions[128]. For this test, we conducted goethite synthesis experiments at $25\,^{\circ}\mathrm{C}$ under alkaline (pH = 11.5) conditions using $FeCl_3$ as the Fe(III) source and FA as the DOC source, which as either added before or after ferrihydrite precipitation. All experiments were performed at least in triplicate, aged for 7 days, and span a DOC/Fe(III) range of $\approx$ 2 orders of magnitude. Results indicate that Fe(III) introduction timing imparts no significant impact on Fe-OC signals, particularly for experiments performed at all but the lowest DOC/Fe(III) ratios (Fig. S15). For example, for the

"−Fh/PC −ads. OC" fraction with a ratio of DOC/Fe(III) = 0.016 to 0.018, Fe-OC loadings were $0.08 \pm 0.01\,\%$ and Δ^{13}C values were $9.7 \pm 0.2\,‰$ when FA was added before ferrihydrite precipitation ($n = 3$), whereas Fe-OC loadings were $0.11 \pm 0.01\,\%$ and Δ^{13}C values were $9.7 \pm 0.2\,‰$ when FA was added after ferrihydrite precipitation ($n = 3$).

This result is consistent with the well-studied pathway of crystalline iron oxide formation, in which continuous dissolution and re-precipitation gives way to a state of equilibrium over a time span of days to weeks[128–130]. Furthermore, previous studies have shown that the presence of DOC has little impact on electron-transfer atom exchange and the transformation to crystalline phases, although it can have a large impact on resulting crystalline phase particle size, particularly at high DOC concentrations[131]. We therefore suggest that co-precipitated Fe-OC signals within the final crystalline phases are set by initial DOC complexation with ferrihydrite, which in-turn depends only on adsorption affinity and thus DOC/Fe(III) ratio, independent of whether DOC was complexed during or after ferrihydrite precipitation. Nevertheless, for consistency, we only include experiments in which DOC was introduced after ferrihydrite formation when calculating our experimental Fe-OC loading and isotope fractionation calibration curves (see "Final Fe-OC calibration curves used for geologic reconstructions", below).

Effect of rinsing and desorption procedure. Lastly, we assessed how removing ferrihydrite-bound and adsorbed OC impacts Fe-OC signals (see "Removal of ferrihydrite-bound and adsorbed OC", above, for method details). This rinsing and desorption procedure is critical for isolating and analyzing only OC that is co-precipitated with crystalline iron oxides. In contrast, ferrihydrite-bound and/or adsorbed OC is not expected to persist over geologic timescales[35, 131] and should be removed before utilizing calibration curves derived from synthesis experiments to reconstruct Earth-history records. We emphasize that our approach specifically describes OC co-precipitation with newly formed iron (hdyr)oxide minerals, a process that is fundamentally different from adsorption onto pre-existing surfaces where surface area and reactive site densities directly dictate OC loading capacities. Insufficient removal of non-geologically preserved OC when generating calibration curves could bias results if this material significantly alters predicted Fe-OC loadings or ^{13}C compositions; we explicitly test this across a range of experimental conditions. For goethite, this includes experiments performed under alkaline conditions (pH = 11.5) at $25\,°$C and $70\,°$C with $FeCl_3$ as the Fe(III) source and FA as the DOC source (added both before and after ferrihydrite precipitation for $25\,°$C experiments); circumneutral conditions (pH = 5-7) at $25\,°$C with $FeCl_3$ as the Fe(III) source and FA as the DOC source; and acidic conditions (pH = 1.8) at $50\,°$C with $Fe(NO_3)_3$ as the Fe(III) source and FA as the DOC source. For hematite, this includes experiments performed under circumneutral conditions (pH = 8.0) at $50\,°$C with $FeCl_3$ as the Fe(III) source and M-DOC as the DOC source and acidic conditions (pH = 1.5) at $75\,°$C with $FeCl_3$ as the Fe(III) source and FA as the DOC source.

In all cases, Fe-OC loadings either decreased (circumneutral to alkaline conditions) or increased slightly (acidic conditions) when moving from "raw" to "−Fh/PC −ads. OC" fractions (Figs. S16, S17). For example, for a ratio of DOC/Fe(III) = 0.016 to 0.039, Fe-OC loading decreased relative to the "raw" fractions for each circumneutral and alkaline experiment by: goethite, alkaline, $25\,°$C: $32 \pm 13\,\%$ for "−Fh/PC" ($n = 6$) and $52 \pm 22\,\%$ for "−Fh/PC −ads. OC" ($n = 6$); goethite, alkaline, $70\,°$C: $14 \pm 8\,\%$ for "−Fh/PC" ($n = 6$) and $22 \pm 9\,\%$ for "−Fh/PC −ads. OC" ($n = 6$); goethite, circumneutral, $25\,°$C: $29 \pm 18\,\%$ for "−Fh/PC" ($n = 6$) and $39 \pm 25\,\%$ for "−Fh/PC −ads. OC" ($n = 6$); and hematite, circumneutral, $50\,°$C: $24 \pm 12\,\%$ for "−Fh/PC" ($n = 6$) and $28 \pm 14\,\%$ for "−Fh/PC −ads. OC" ($n = 6$). For acidic experiments under the same range of DOC/Fe(III) ratios, Fe-OC loadings either increased slightly (goethite, acidic, $25\,°$C) or increased slightly then decreased (hematite, acidic, $75\,°$C) when moving from

"raw" to "−Fh/PC" and "−Fh/PC −ads. OC" fractions. In summary, Fe-OC loading is almost always highest in the "raw" fraction, which includes low-crystallinity iron oxides and ferrihydrite, likely due to the larger surface area of ferrihydrite relative to crystalline goethite and hematite[45]. Similarly, Fe-OC loadings are almost always higher in the "−Fh/PC" relative to the "−Fh/PC −ads. OC" fraction, as the former retains organic compounds that are adsorbed to—rather than co-precipitated with—crystalline phases. Nevertheless, Fe-OC loadings increase with increasing DOC/Fe(III) ratios across all protocol stages.

In contrast, δ^{13}C changes between protocol stages exhibited more nuanced behavior. For example, for a DOC/Fe(III) ranging from 0.016 to 0.039, Fe-OC δ^{13}C values changed relative to the "raw" fractions for each experiment by: goethite, alkaline, $25\,°$C: $-4.0 \pm 1.0\,‰$ for "−Fh/PC" ($n = 6$) and $-4.4 \pm 0.8\,‰$ for "−Fh/PC −ads. OC" ($n = 6$); goethite, alkaline, $70\,°$C: $-5.0 \pm 1.2\,‰$ for "−Fh/PC" ($n = 6$) and $-3.6 \pm 1.2\,‰$ for "−Fh/PC −ads. OC" ($n = 6$); goethite, circumneutral, $25\,°$C: $1.6 \pm 1.9\,‰$ for "−Fh/PC" ($n = 6$) and $3.4 \pm 1.6\,‰$ for "−Fh/PC −ads. OC" ($n = 6$); goethite, acidic, $50\,°$C: $-1.1 \pm 0.8\,‰$ for "−Fh/PC" ($n = 6$) and $-0.9 \pm 0.6\,‰$ for "−Fh/PC −ads. OC" ($n = 6$); hematite, circumneutral, $50\,°$C: $-2.1 \pm 0.2\,‰$ for "−Fh/PC" ($n = 6$) and $0.0 \pm 0.7\,‰$ for "−Fh/PC −ads. OC" ($n = 6$); and hematite, acidic, $75\,°$C: $0.5 \pm 0.4\,‰$ for "−Fh/PC" ($n = 3$) and $0.0 \pm 0.5\,‰$ for "−Fh/PC −ads. OC" ($n = 3$). In summary, when moving from "raw" to "−Fh/PC −ads. OC" fractions, alkaline goethite experiments exhibit an increase in Δ^{13}C (i.e., decrease in $\delta^{13}C_{Fe-OC}$), circumneutral goethite experiments exhibit a decrease in Δ^{13}C, and hematite experiments under all conditions exhibit little to no change in Δ^{13}C.

Under most experimental conditions, our step-wise washing procedure exhibits pronounced impacts on measured Fe-OC loadings and/or isotope compositions. Future studies are needed to further investigate the kinetic processes governing Fe-OC formation, complexation, and stability. Regardless of the exact kinetic process, this result underscores the need to remove non-geologically preserved organic compounds (i.e., those adsorbed to surfaces or associated with poorly crystalline phases) before utilizing co-precipitation experiments to generate Fe-OC calibration curves. We therefore utilize the final fraction (i.e., "−Fh/PC −ads. OC")—posited here as the most representative of geological hematite and goethite—for all synthetic and geologic iron oxides used to generate calibration curves and Earth-history records in this study.

Final Fe-OC calibration curves used for geologic reconstructions. To interpret our geologic records and extract DOC signals throughout Earth's history, we generated calibration curves to relate Fe-OC loadings and $\delta^{13}C_{Fe-OC}$ values to initial solution DOC/Fe(III) ratios and $\delta^{13}C_{DOC}$ values for each DOC source. Considering the effects of all synthesis conditions and rinsing procedures discussed above, calibration curves were generated using results from experiments performed under the following conditions: For goethite, T = $25\,°$C; pH = 5-7; and duration = 10-14 days (M-DOC and C-DOC) or 10-120 days (FA). For silica-free hematite, T = $50\,°$C; pH = 8.0; and duration = 5-30 days (M-DOC), 5-14 days (C-DOC), or 14 days (FA). Hematite calibration curves with C-DOC were additionally generated for each tested silica concentration under the following conditions: T = $50\,°$C; pH = 8.0; and duration = 14 days. For both minerals, DOC/Fe(III) was adjusted over ≈ 3 orders of magnitude by manipulating either DOC or Fe(III) concentrations; $Fe(Cl)_3$ was used as the Fe(III) source; DOC was added after ferrihydrite precipitation; and "−Fh/PC −ads. OC" fractions were analyzed.

For both minerals and all DOC sources, a clear dose-dependent response is observed in which Fe-OC loadings increase with increasing DOC/Fe(III) (Figs. S18-S19). Across the DOC/Fe(III) ratios considered here, Fe-OC loadings span from $\approx 0.01\,wt\,\%$ to $>1.0\,wt\,\%$ for goethite and from $\approx 0.01\,wt\,\%$ to $\approx 0.4\,wt\,\%$ for hematite. This two order-of-magnitude range

in Fe-OC wt % indicates that iron ooid Fe-OC loading is a sensitive and robust tracer for marine DOC/Fe(III) ratio, particularly under DOC-poor conditions when loading curves are steepest.

Interestingly, the quantitative Fe-OC wt % for both minerals at a given DOC/Fe(III) ratio depends linearly on DOC slope ratio (S_R), a proxy for the degree of processing by heterotrophic respiration and/or photodegradation (see "Molecular and functional characteristics of experimental DOC", above)[40]. For example, Fe-OC loadings in goethite with DOC/Fe(III) = 0.16 to 0.18 decrease moving from FA (S_R = 0.67) to M-DOC (S_R = 1.29) to C-DOC (S_R = 2.20) with a slope of $-0.47\,\mathrm{wt}\,\%\,{S_R}^{-1}$ ($n = 3$, $r^2 = 0.995$; Fig. S23). Similarly, loadings in hematite with DOC/Fe(III) = 0.06 to 0.07 decrease with a slope of $-0.08\,\mathrm{wt}\,\%\,{S_R}^{-1}$ ($n = 3$, $r^2 = 0.993$; Fig. S23). These results indicate that—all else being equal—more processing by heterotrophic respiration or photodegradation will lead to lower Fe-OC loadings. Future work is warranted to determine the chemical mechanism of this result. Nevertheless, we assess the impact of molecular composition on our Earth-history DOC concentration predictions by inverting our measured iron ooid record using calibration curves for several possible DOC composition scenarios (see "Estimating DOC concentrations and δ^{13}C values from iron ooid Fe-OC", below).

Carbon-isotope fractionation exhibits more complex behavior (Figs. S18-S19). For goethite, using either M-DOC or FA as the DOC source yields positive Δ^{13}C values (i.e., Fe-OC is more depleted in ^{13}C than substrate DOC) as high as 12 ‰. In contrast, C-DOC exhibits slightly negative but largely invariant Δ^{13}C values. For hematite, all three DOC sources yield negative Δ^{13}C values (i.e., Fe-OC is more enriched in ^{13}C than substrate DOC) as low as -20 ‰. This diverging fractionation behavior does not clearly depend on any DOC composition metric measured here. Additionally, it is unlikely that fractionation results only from preferential co-precipitation of a compositionally and thus isotopically unique subset of the overall DOC reservoir, as evidenced by the observation that FA—which is comprised of a single molecular structure or small group of compositionally similar molecular structures—exhibits a different sign and magnitude of fractionation when co-precipitated with goethite and hematite. Rather, we hypothesize that observed fractionation results from a kinetic isotope effect whose magnitude depends on DOC/Fe(III) ratio. Further work is clearly warranted to investigate the exact mechanism(s) governing fractionation, particularly the difference in sign and magnitude observed for different minerals and DOC sources.

Nevertheless, for both minerals and all DOC sources, the magnitude of Δ^{13}C decreases with increasing DOC/Fe(III) ratio and converges to $\Delta^{13}\mathrm{C} = 0$ (i.e., no fractionation) when DOC/Fe(III) $>\approx 0.1$. This indicates that observed Fe-OC δ^{13}C values can be interpreted directly as DOC δ^{13}C values when DOC concentrations are high. Still, like for DOC concentration predictions, we assess the impact of mineralogically and DOC compositionally unique fractionation factors on our Earth-history DOC δ^{13}C predictions by inverting our measured iron ooid record using calibration curves for several possible DOC composition scenarios (see "Estimating DOC concentrations and δ^{13}C values from iron ooid Fe-OC", below).

To perform such inversions, we fit each Fe-OC loading and Δ^{13}C calibration curve using either a power law (for concentrations) or a dual exponential (for Δ^{13}C) function. The former was chosen as it represents a Freundlich sorption isotherm[39] whereas the latter was chosen to empirically capture Δ^{13}C trends while ensuring that values converge to zero and high DOC/Fe(III) ratios. Specifically, we calculated Fe-OC loading calibration curves as

$$w^{i,m} = \beta_0^{i,m} \left(x^i\right)^{\beta_1^{i,m}}, \tag{S1}$$

where $w^{i,m}$ is the Fe-OC loading (in wt. %) of DOC source i onto iron-oxide mineral m; $\beta_0^{i,m}$ and $\beta_1^{i,m}$ are empirically fit power-law constants; x^i is the DOC/Fe(III) concentration ratio ($\mathrm{mol\,mol^{-1}}$) for DOC source i; i = FA, C-DOC, or M-DOC; and m = goethite or hematite.

Similarly, we calculated Fe-OC isotope fractionation calibration curves as

$$\Delta^{i,m} = \beta_2^{i,m} \exp\left(\beta_3^{i,m} x^i\right) + \beta_4^{i,m} \exp\left(\beta_5^{i,m} x^i\right) \tag{S2}$$

where $\Delta^{i,m}$ is the Δ^{13}C value (in ‰) of DOC source i onto iron-oxide mineral m and $\beta_2^{i,m}$ through $\beta_5^{i,m}$ are empirically fit dual exponential constants. We fit Eqs. S1-S2 to results for each mineral and DOC source using ordinary least squares (OLS) regression (Figs. S18-S19). Model fits for all DOC sources for both minerals always display $r^2 \geq 0.83$ (and typically $r^2 \geq 0.95$), with resulting root-mean square errors (RMSE) always $\leq 0.09\,\mathrm{wt}\,\%$ for Fe-OC loadings (typically $\leq 0.05\,\mathrm{wt}\,\%$) and $\leq 1.3\,‰$ for Δ^{13}C values (typically $\leq 1.0\,‰$; Table S2). Such high degree of data-model agreement further confirms the utility of iron oxide Fe-OC signals as proxies for marine DOC concentrations in the geologic past.

Finally, individual β values for each regression are highly correlated—omission of this correlation would lead to improper error propagation when estimating Earth-history trends. We therefore propagated uncertainty using a Monte Carlo approach by solving Eqs. S1-S2 for 1,000,000 random draws including analytical error and calculating the covariance of resulting β values (Figs. S20-S22). All fitting results for calibrations used in our Earth-history reconstructions are reported in the Supplementary Data.

3 Iron ooid formation mechanism and timescale.

Before reconstructing geologic records, we attempted to constrain iron ooid formation mechanism and timescale. Below, we describe insight gained by studying two modern sites in which iron (oxyhydr)oxide ooids are actively forming: (i) the Panarea volcanic complex in Italy's Aeolian archipelago and (ii) a hydrothermal vent field near Mahengentang Island in Indonesia. While informative, such insights are inherently incomplete; in particular, the precise Fe(III) concentrations at both sites are currently unconstrained. We additionally discuss the potential role of microbial activity in facilitating ooid formation.

3.1 Iron ooid formation mechanism

Insight from modern sites. Formation mechanism can be inferred from observed characteristics at both modern sites. At Panarea Island, hydrothermal iron is believed to be actively supplied since the last volcanic eruptive phase in the late Pleistocene to early Holocene (i.e., $\geq$ 8.7 ka based on the timing of volcanic edifice formation)[44, 132]. Such iron supply leads to ooids that exhibit concentric layers of alternating poorly crystalline iron (oxyhydr)oxides and goethite enveloping a volcanic or biogenic fossil core. XRD analysis, elemental mapping, and Raman microspectroscopy further elucidate the compositional and mineralogical architecture of these ooids (Figs. 1, S25-S27). Results indicate that goethite is the dominant crystalline phase and that various element associations are consistent with a marine setting with hydrothermal influence. At Mahengentang Island, iron ooids similarly form in a warm (i.e., $\approx 42\,°\mathrm{C}$ at the sediment-water interface), shallow marine hydrothermal vent field that is estimated to be active at least since 4.5 ka (based on the timing of sea-level rise)[43]. Resulting ooids exhibit concentric layers of poorly crystalline iron (oxyhydr)oxide, low-crystallinity goethite, and amorphous silica enveloping a predominantly volcanic core. Like for Panarea Island ooids, XRD and elemental mapping results are consistent with a marine setting with hydrothermal influence, as evidenced by the distributed presence of phosphorus and sulfur (Figs. S28-S29). The alternation between goethite and poorly crystalline layers at both sites is interpreted to reflect distinct depositional settings or accretion events, suggesting temporal cyclicity in environmental conditions as ooids form.

The potential role of local conditions, direct microbial precipitation, and diagenesis. One concern of our approach is the possibility that preserved Fe-OC loadings and δ^{13}C values do not capture global trends but rather represent local- or even single grain-scale signals. In particular, if iron (oxyhydr)oxide formation and ooid precipitation is directly facilitated by microbial activity, then ooids may incorporate organic matter from microbial biomass or extracellular polymeric substances (EPS) rather than from marine DOC. Furthermore, the presence of EPS could lead to entrainment of local, detrital POC grains. If true, then preserved Fe-OC signals would be biased to reflect the activity and ^{13}C fractionation of whichever microbial communities are hosted within or around iron oxide grains. This phenomenon is commonly implicated when describing the mechanism of carbonate ooid formation[133,134], but it has not yet been thoroughly tested for iron ooids. Here, we present four lines of evidence against the importance of local microbial activity in driving iron ooid Fe-OC signals. We additionally discuss any sample-specific evidence when describing the relevant formations below (see "Geologic descriptions of sampled formations").

First, Fe-OC loadings and δ^{13}C values are remarkably consistent for contemporaneous formations sampled over spatial scales of $\sim$ 100 to $\sim$ 1000 km. This includes signals from two Cretaceous formations collected across Israel, nine Jurassic formations collected across western Europe, four Ordovician formations collected across northern and eastern Europe, and two Tonian formations collected across North American (see "Intra-period spatial variability", below). In contrast, such consistency over large spatial scales would not be expected if local conditions—for example, influence of terrestrial runoff and local detrital inputs or spatially unique microbial matabolisms driven by regionally heterogeneous nutrient regimes—were the principal driver of Fe-OC signals. We interpret this result as evidence that microbial activity at the regional scale does not drive observed signals.

Second, we performed petrographic analysis and elemental mapping on all samples contained within our records—including actively forming ooids from both modern sites—with the aim of identifying possible EPS incorporation (see "Geologic descriptions of sampled formations", below). EPS are high molecular weight biopolymers secreted by microorganisms into their environment that often form a protective and adhesive matrix (termed a "biofilm") around microbial cells[135]. EPS can significantly influence the local environment, both chemically by inducing mineral precipitation[136] and physically by trapping and entraining particles within the biofilm[133,134]. Thus, if microbial activity at the grain scale were an important driver of ooid formation—for example, by entraining iron (oxyhydr)oxide grains within a biofilm—then we would expect to observe distinct morphological features due to EPS incorporation as well as localized or "patchy" Fe-OC. However, this is never observed. Instead, our Raman microspectroscopy results consistently indicate that Fe-OC within all analyzed modern and ancient ooids is homogenously distributed throughout the iron oxide layers (Fig. 1). Raman spectra line scans and spatial maps show consistent G-band intensity characteristic of Fe-OC material across all cross sections, with no localized hotspots. This homogeneity does not support the presence of concentrated EPS biofilms or concentrated microbial cell residues but rather implies that Fe-OC was incorporated uniformly during abiotic mineral precipitation from seawater. Combined, the absence of radial cortex features and observed homogeneous Fe-OC distributions provide strong evidence against the importance of EPS and microbial activity in forming iron ooids.

Third, some microaerophilic iron oxidizing bacteria—which have been invoked to explain geologic deposits such as banded iron formation precipitation—produce iron (oxyhdr)oxides with distinctive stalk and stem morphologies[137]. Any observation of such morphologies in our ooids would therefore imply that iron (oxyhdr)oxides in these grains are directly formed by microorganisms and that any Fe-OC signals reflect direct microbial exudates rather than ma-

rine DOC. However, like for EPS incorporation, biogenic stalks and stems are never observed in any iron ooid included in our dataset. Furthermore, petrographic results argue against the importance of diagenetic recrystallization, which would remove and overprint any diagnostic microbial morphologies. Rather, all ooids included in our dataset display finely laminated goethite or hematite rims, indicating preservation of primary morphologies and structures. We therefore interpret the retention of fine laminae combined with the absence of biogenic stalks and stems as evidence against the importance of direct biogenic iron (oxyhydr)oxide precipitation. Nevertheless, not all iron oxidizing bacteria produce diagnostic stalks and stems, so this line of evidence alone should be interpreted with caution.

Finally, we analyzed microbial community compositions of surface-bound and internal DNA on modern ooids from Panarea Island, Italy[44] using 16S rRNA gene amplicon sequencing. Approximately 177 ASVs remained after filtering to remove possible contaminant taxa (i.e., contained in reagent-only negative controls). At the family level, *Unclassified Bacteria*, *Desulfonatronaceae*, and *Rhizobiaceae* were the most abundant taxa; importantly, well-known taxa for iron oxidation such as *Mariprofundus* (within Zetaproterobacteria)[138,139], *Gallionellaceae* (e.g., genera *Gallionella* and *Sideroxydans*; within Gammaproteobacteria)[140,141], *Pseudoalteromonadaceae* (within Gammaproteobacteria)[142] and *Hyphomonadaceae* (within Alphaproteobacteria)[143,144] were not detected in our ooid sample (Fig. S24). Still, some detected taxa may contain organisms that are relevant for iron cycling. For example, the haloalkaliphilic lithotrophic sulfate-reducing bacterial family *Desulfonatronaceae* may be involved in iron reduction[145]. However, there is no evidence that members of this family can also perform iron oxidation. Similarly, *Rhizobiaceae*-associated bacteria have been detected in marine manganese-oxidizing enrichment cultures[146], although members of this family are primarily known for their role in nitrogen fixation. Other major taxa detected include *Caminicellaceae*, *Marinobacteraceae*, *Pseudonocardiaceae*, and *Thermoanaerobaculaceae*. Microbial populations belonging to *Caminicellaceae* and *Marinobacteraceae* have been isolated from deep-sea hydrothermal vents and can be thermophilic and halophilic[147–150]. Bacteria within *Marinobacteraceae* are able to perform Fe(II) oxidation under heterotrophic conditions[151–153]. However, not all *Marinobacter* species are capable of iron oxidation; the importance of the presence of this family thus remains ambiguous. Finally, the family *Thermoanaerobaculaceae* contains thermophilic anaerobic microbes that are capable of reducing sulfate[154]. Notably, no other major taxa identified here were reported as iron-oxidizing associated microbial communities, even though we cannot exclude possible contamination from sampling, storage, sequencing facilities.

These combined observations strongly support the interpretation that local- and grain-scale microbial activity exhibits a minimal impact on our record. Thus, while biological pathways cannot be definitively ruled out, we parsimoniously interpret actively forming iron ooids to precipitate abiotically in the presence of hydrothermal iron supply and gentle wave action and to incorporate dissolved constituents directly from seawater into their crystal lattice.

3.2 Iron ooid formation timescale and accretion rate

An additional concern is the possibility that Fe-OC incorporates detrital inputs (e.g., DOC emitted at hydrothermal vents, DOC with non-marine sources)[155,156], which are not representative of typical marine conditions and thus could lead to biased Fe-OC signals. At modern sites, radiocarbon analysis of co-precipitated Fe-OC is an ideal means to test this possibility, since detrital inputs are by definition ^{14}C-free (i.e., Fm = 0). Contrary to this concern, radiocarbon activities of Fe-OC in bulk ooids ("−Fh/PC −ads. OC" fraction) indicate average ages of $5203 \pm 76\ ^{14}\mathrm{C\,yr}$ at Panarea Island (Fm = 0.5232 ± 0.005, $n = 1$) and $3552 \pm 73\ ^{14}\mathrm{C\,yr}$ at Mahengentang Island

(Fm = 0.6472 ± 0.006, $n = 1$), despite the known presence of hydrothermal fluids at these sites (Table S3).

Such ages could be interpreted as an admixture of Fe-OC derived from 52 to $65\,\%$ modern marine DOC (Fm $\sim$ 1, here ignoring water-column pre-aging of semi-recalcitrant to recalcitrant marine DOC for simplification)[13] and 35 to $48\,\%$ ^{14}C-free detrital DOC (Fm = 0). However, such interpretation would require that all concentric layers observed within a given ooid accrete on the order of decades (i.e., without significant radioactive decay of Fe-OC after incorporation into ooids). This is inconsistent with independently estimated timescales of ooid site formation of $\geq$ 8.7 kyr at Panarea Island and 4.5 to 15 kyr at Mahengentang Island[43,44]. We therefore interpret measured ^{14}C ages as reflecting the mean age of all concentric layers in a given ooid. If we first make the simplifying assumptions that DOC is not pre-aged in the water column, OC content is evenly distributed between all layers, and each ooid has been accreting continuously, then our radiocarbon results imply that ooids began accreting $\approx$10 400 ^{14}C yr ago at Panarea Island and $\approx$7000 ^{14}C yr ago at Mahengentang Island. These ages are within the range of site formation timescale estimates[43,44], suggesting that individual ooid grains have been actively forming since both sites developed.

Still, these ages are higher than minimum formation timescale estimate at each site (i.e., 8.7 ka and 4.5 ka for Panarea Island and Mahengentang Island, respectively). This could result from some combination of (i) minor detrital DOC contributions or (ii) pre-aging of DOC in the water column prior to incorporation into iron ooids. If the latter explanation is true, this would imply that DOC is pre-aged by $\sim$1700-2400 years upon incorporation into ooids. Although significantly younger than modern deep-ocean DOC, this age is similar to measured ^{14}C ages of modern surface-ocean DOC, which includes a mixture of material ranging from labile to recalcitrant (e.g., Refs.[13,15,53]). Thus, ^{14}C ages of modern ooids appear consistent with the interpretation that labile, semi-labile, semi-recalcitrant, and recalcitrant DOC compounds are continuously co-precipitated. Nevertheless, given the large and unknown uncertainties associated with each assumption in this estimation (i.e., time of ooid formation initiation, even Fe-OC content distribution, and continuous accretion), such results should be interpreted cautiously.

Furthermore, iron ooid accretion rates can be estimated by combining accretion timescales calculated here with constraints on ooid thickness. Importantly, this yields an average net accretion rate over the entire ooidal lifespan; we do not explicitly consider alternative periods of abrasion and accretion. Microscopy images indicate ooid diameters of $\approx$ 200 to 300 μm with $\approx$ 60 to 75 μm cores at Panarea Island and $\approx$1100 μm with $\approx$400 μm cores at Mahengentang Island (Fig. S25-S29). Combined with our ^{14}C-based accretion timescales, this yields accretion rates of $\sim$ 5 to 50 μm kyr^{-1} at both sites. We therefore treat each ooid in our geologic record as capturing a $\sim$ 10 kyr snapshot of marine DOC signals.

4 Geologic descriptions of sampled formations

Here, we describe the geologic context for all iron ooid formations included in this study. Optical and electron microscopy images, bulk-rock and picked-ooid XRD-based mineralogy assessments, and SEM-EDS-based elemental maps for representative samples from each formation are shown in Figs. S25-S123. Modern latitude, paleolatitude, and extracted ooid XRF results for all sampled formations are included in the Supplementary Data.

4.1 Panarea Island, Italy (modern)

Actively forming iron ooids have recently been discovered at $\approx$80 m water depth in the Panarea volcanic complex in the Aeolian archipelago, Italy[44,132]. Hydrothermal iron supply leading to

ooid formation has been ongoing at least since the last volcanic eruptive phase in the late Pleistocene to early Holocene (i.e., ≈10 to 15 ka). Resulting ooids—which form in $\approx 10\,^{\circ}\mathrm{C}$ water and can reach several millimeters in diameter—feature concentric layers composed of alternating poorly crystalline iron (oxyhydr)oxides (dark red rings in Fig. S25A-B) and goethite (light orange rings in Fig. S25A-B) that surround a nucleus of volcanic (rock fragments, pumice, glass scoria, volcanogenic mineral phases) or biogenic fossil origin. Our XRD analysis reveals that the crystalline component of the ooids is predominantly goethite, with no other phases detected, whereas the bulk sediment predominantly exhibits quartz (Fig. S25C). Furthermore, elemental maps (Figs. S26-S27) show that goethite is associated with Cl, Mg, and P, while ooid nuclei are volcanogenic mineral phases associated with Si, Al, Ca and K. Collectively, these findings suggest formation in a modern marine setting influenced by hydrothermal input. Poorly crystalline vs. goethite layers are interpreted to reflect distinct depositional settings; the observation that they alternate thus implies temporal variations in depositional environment as ooids form[44]. Biogenic fossil assemblages that constitute ooid nuclei are dominated by several species of foraminifera, indicating that the depositional environment is influenced by "normal" background marine sedimentation in addition to hydrothermal activity[44].

4.2 Mahengentang Island, Indonesia (modern)

Actively forming iron ooids have also been observed in a shallow marine hydrothermal vent field near Mahengentang Island, Indonesia[43]. These unconsolidated ooids are comprised of concentric layers of poorly crystalline iron (oxyhydr)oxide (dark red rings in Fig. S28A), low-crystallinity goethite (light orange rings in Fig. S28A), and amorphous silica (black rings in Fig. S28A) that envelop a core of predominantly volcanic origin[43]. Our XRD analysis reveals that the crystalline ooid component is predominantly goethite, with no other phases detected, whereas the bulk sediment predominantly exhibits quartz and andesite components (Fig. S28C). Furthermore, elemental maps (Fig. S29) show that goethite is associated with K and P, while andesite-rich ooid nuclei are characterized by associations with Na, Ca, Si, and Al. This ooid deposit, with a maximum thickness of $\approx 0.5\,\mathrm{m}$, has been forming in warm shallow environment ($\approx 42\,^{\circ}\mathrm{C}$ at the sediment-water interface) since at least $5\,\mathrm{ka}$[43].

4.3 Lisakovsk Channel Iron Deposit, Chegan Fm, Kazakhstan (∼28 Ma)

The Lisakovsk channel island deposit (CID), situated within the Turgay depression in northern Kazakhstan, is a prolific source of ironstone. Geochronological constraints place the Lisakovsk CID within the Chegan Formation[157]. The Lisakovsk CID showcases both shallow marine and fluvial depositional environments—as evidenced by the presence of freshwater fauna and flora[157]—and is dominantly associated with a fluvial milieu characterized by poorly sorted quartz sands interspersed with goethite ooids and iron-bearing sediments[158]. This observed lithofacies diversity is evidence for formation in a deltaic channel environment.

Lisakovsk CID ironstones materialized through progressive dissolution and replacement of unstable allogenic components. Nearby Upper Cretaceous marine ironstone deposits within the Turgay depression are the most parsimonious sources of metal for this replacement[157,159]. These geochemical signatures, combined with a lack of significant detrital inputs, likely imply a predominantly hydrogenic origin of Lisakovsk CID ironstones. Ooids comprising these ironstones are goethite rich and are cemented by carbonate with dispersed quartz grains (Fig. S30). Furthermore, elemental maps (Figs. S31-S32) show distinct Fe-rich ooid rims and Al- and Si-rich cores, potentially comprised of Al-silicates. Although goethite ooids in the Lisakovsk CID were initially believed to be deltaic in origin, recent work suggests that they originated from the

nearby Cretaceous Ayat Formation. These ooids appear to have undergone minimal alteration before being re-deposited in the adjacent channel[160]. We retain the Lisakovsk CID goethite ooids in our compilation to mitigate potential bias due to subjective sample screening. Still, we highlight the probability that Fe-OC results may be influenced by continental inputs and that these ooids may be assigned an improper age.

4.4 Bakchar Horizon, Lyulinvor Fm, Russia (56-59 Ma)

The Lyulinvor Formation is situated ≈200 km west of Tomsk, Russia in the southeastern West-Siberian Basin. This formation exhibits coastal marine facies[161] that contain ooidal ironstones, sandstones, siltstones, and clays[162]. Of particular interest here, ooidal ironstones found within the Narym, Bakchar, and Kolpashevo horizons are comprised of goethite-hydrogoethite and chlorite (i.e., chamosite)-hydrogoethite[162]. Biostratigraphic data indicate that these three ooidal ironstone horizons span ages ranging from the Late Cretaceous (Turonian) to the Eocene[161,163,164].

Samples analyzed in this study are specifically derived from the Paleocene aged Bakchar horizon, which was deposited in a marginal marine setting and contains three distinct facies: (i) an uncemented goethite ooidal ironstone at the base, (ii) uncemented chamosite-rich ironstones, and (iii) siderite-cemented goethite ooidal ironstone further up-section. The observation that goethite ooids (and occasional quartz grains) are unconsolidated implies that this horizon has not experienced deep burial[165]. Rather, the presence of siderite cements in facies (iii) has been proposed to result from methane migration and subsequent alkalinity generation by microbial oxidation during the Paleocene-Eocene Thermal Maximum (see main text for discussion of methane impacts on resulting Fe-OC signatures)[162]. In accordance with our exclusion protocols, samples analyzed here derive from facies (i); they lack ferrous minerals and show no signs of methane-driven reduction of iron (oxyhydr)oxides. Ooids comprising these ironstones are goethite rich and are loosely cemented by carbonate with quartz grains (Fig. S33). Furthermore, elemental maps (Fig. S34) indicating that goethite ooids sit within a matrix of Si-rich quartz grains, while co-occurrence of Al, P, S and K indicate the presence of Fe-rich silicates.

4.5 Hatira Fm, Israel (100-113 Ma)

The Early Cretaceous Hatira Formation, located in the Negev Desert, southern Israel, is primarily comprised of continental facies interspersed by occasional thin marine transgressions. Of particular interest here is the Hatira Makhtesh, a unique erosional feature characterized by ≈400 m of friable continental standstones punctuated by four marine sequences composed of limestones, dolomites, and marls that contain trace goethite ooidal ironstones[166]. Goethite ooids were collected for this study from several locations in the marine units of the Hatira Makhtesh. Sampled ooids are spherical and are cemented by dolomite and calcite carbonates rich in marine fossils (Figs. S35-S40). Elemental maps (Figs. S41-S46) confirm the Mg- and Ca-rich dolomite matrix and Fe-rich goethite ooids. Ooid-containing members were determined to be Albian in age based on biostratigraphy. Specifically, the presence of the ammonite *Knemiceras syriacum* and the gastropods *Pseudomesalia deserti* and *Diastoma ornatum* matches the assemblage of a reference collection based on Albian outcrops in Bir Lagma, Sinai, accessed through Dr. Zeev Lewy, Geological Survey of Israel. The Hatira Formation has remained well-preserved; it was subjected to a maximum burial depth of ≈1100 m and experienced low burial temperatures from 22 °C to 27.5 °C[167]. This excellent preservation, along with consistent geological characteristics across a ≈25 km spatial scale, establishes the Hatira Formation as an ideal model to assess Fe-OC spatial variability at the formation scale.

4.6 Hidra Fm, Israel (100-113 Ma)

Contemporaneous in age to the Hatira Formation, the Hidra formation is located in the Galilee and Golan regions of northern Israel. It is comprised of several facies including sandstone, fossiliferous limestone containing iron ooids, limestone containing *Orbitolina* foraminifera, and shale[168]. The Hidra Formation has been confirmed as Albian in age based on stratigraphic relationships and ostracod biostratigraphy[169]. Thus, by comparing the Hatira and Hidra formations, we are able to assess super regional-scale (i.e., order hundreds of kilometers) variability in the signatures of preserved iron ooid of the same age. Samples analyzed in this study were collected from the Rehes Ramim section. Forming the upper portion of the Hidra Formation, this section is characterized by limestone containing abundant marine fossils, siliciclastics, and goethite ooids; it is thought to represent a shallow subtidal to outer platform depositional environment[170].

For this study, well-preserved samples were collected across a 60 m transect through an abandoned underground mineshaft[42]. By descending at an angle to the dip, the mineshaft geometry captures lateral variability while also allowing for detailed stratigraphic descriptions across several meters of section. In addition to mineshaft walls, samples were collected from nearby boulders and outcrops up to $\approx$100 m from the mine entrance. Sampled ooids are spherical goethite ooids which were cemented by fossils-rich calcite cement (Figs. S47-S53. Elemental maps (Figs. S54-S60) confirm the Ca-rich calcite cement and the Fe-rich spherical goethite ooids. Like the contemporaneous Hatira Formation, the Hidra formation represents a model formation from which we sampled across a $\sim$ 10 km spatial distance. Through these samples, we aimed to determine expected Fe-OC signal variability within a single rock formation. Combined, the results from these two formations represent an ideal test to constrain $\sim$ 100 km spatial variability in Fe-OC signals (see "Intra-period spatial variability", below).

4.7 Arroyofrío Bed, Chelva and Yatova Fms, Spain (157-166 Ma)

The Middle- to Late-Jurassic (Callovian-Oxfordian) aged Arroyofrío Bed is located in modern-day Iberian Chain and sits within a shallow-marine carbonate platform that communicated with the western Tethys Ocean. The bed is comprised of several iron ooid limestone layers and transitions into a black, fossil-rich, micritic limestone sequence towards the northwest. Although characterized by stratigraphic discontinuities, the bed's temporal profile is well established through a rich ammonite biostratigraphy record[171]. Predominantly carbonate facies manifest as bioclastic wackestones to packstones containing iron ooids. These are understood to have formed in a shallow subtidal environment on an epicontinental carbonate platform. Earlier studies leveraged mineralogical and elemental composition data to postulate a shallow marine depositional environment; they proposed the iron source to be the result of local volcanic material alterations[172]. Anisotropy magnetic susceptibility analyses are additionally consistent with subtidal paleocurrent data, further corroborating a marine origin for these ooids[173].

Samples examined in this study were collected from several stratigraphic levels within the Arroyofrío Bed. Ooids are situated in a carbonate matrix and are primarily composed of goethite along with some detrital aluminosilicate-rich material (Figs. S61-S62). Furthermore, elemental maps (Figs. S63-S64) confirm that P-rich iron ooids are sparse. Sampled ooids are described as spherical goethite ooids in a carbonate matrix.

4.8 Oolithe Ferrugineuse de Villers Fm, France (162-163 Ma)

The Oolithe Ferrugineuse de Villers Formation is located in Villers-sur-Mer within the Vaches Noires cliffs in Normandy, France. This formation is characterized by a roughly five-meter thick stratum of white, rust-colored, or yellow limestone. The limestone is rich in clay coupled with micritic cement; it is sporadically interlayered with marl and is abundant in iron ooids[174]. This formation is marked by bioturbation and is believed to have been deposited under marine conditions that corresponded with a highstand system[174]. Abundant ammonite fossils can be found throughout the unit; biostratigraphic analyses place these in the Middle to Late Jurassic Callovian to Lower Oxfordian[175].

Samples collected for this study comprise a coral- and gastropod-bearing, ooid-rich fossiliferous carbonate. Two distinct varieties of ooids are observed: (i) smaller goethite ooids and (ii) larger calcite ooids. The textural relationships, together with the lack of multi-coating in the carbonate ooids, may suggest that carbonate ooids were formed by replacement of iron (oxyhydr)oxides during diagenesis. As a result, the goethite ooids are likely preserving a primary signal rather than one altered through diagenesis. Sampled ooids are described as spherical goethite ooids in a calcite matrix (Figs. S65-S66). Furthermore, elemental maps (Figs. S67-S68) highlight the preservation of iron relics within the calcite ooids, further supporting the idea that the goethite ooids retain a primary origin.

4.9 Ifenthal, Wutach, "Humphriesioolith", and "Parkinsoni-Württembergica Schichten" (informal) Fms, Switzerland ($\sim$164-169 Ma)

The Middle-Jurassic Ifenthal and Wutach Formations, as well as the informal "Humphriesioolith" Formation and "Parkinsoni-Württembergica Schichten", are predominantly situated in northern Switzerland. Parts of these formations are described by several facies representing an array of depositional marine environments[176,177]. Of particular interest here, they contain abundant iron ooid deposits that are primarily composed of goethite, although chamosite ooids have additionally been reported in the literature[178]. Samples analyzed here were sub-sampled from the boreholes: Bözberg-1 (BOZ1-1-406.52, 506.72) , Marthalen-1 (MAR1-1-502.35, 502.81, 546.02, 547.75), and Trüllikon-1 (TRU1-1-766.72), which were collected as part of the Swiss National Cooperative for the Disposal of Radioactive Waste (Nagra)[179–181]. These cores, separated by $\approx$50 km, offer an additional opportunity to examine the lateral distribution of ooid signatures in extremely well-preserved samples at the regional scale[182]. Furthermore, samples from this region are characterized by uniquely low burial temperatures, typically not exceeding 100 °C[183,184]. The formations of interest are known to harbor a diverse array of fossils including ammonites and palynomorphs; biostratigraphic analyses place them in the Callovian to Bajocian stages of the Middle Jurassic epoch[185,186].

Studied samples are composed of purely goethite ooids situated within a carbonate or silicious matrix. Some samples contained ooids displaying partial replacement of goethite by chamosite; such samples were excluded from this work to avoid potential biases (i.e., since the impact of mineral replacement on Fe-OC signatures remains unknown). Specifically, sampled ooids are described as spherical goethite ooids in a dolomite-rich matrix (Figs. S69-S72). Furthermore, elemental maps (Figs. S73-S80) confirm the Fe-rich nature of the ooids and reveal the presence of minor Al- and Si-bearing phases, indicating trace amounts of aluminosilicates.

4.10 Blaukalk Member, Wedelsandstein Fm, Germany (168-170 Ma)

The Blaukalk member of the Wedelsandstein Formation in southern Germany is a yellow, fossiliferous limestone member that extends ≈2 m in thickness. It showcases an array of marine fauna including bivalves, serpulids, bryozoans, ammonites, and sponges; it is thus interpreted as a shallow marine depositional environment[187,188]. Biostratigraphic dating of ammonites places the Blaukalk member in the Bajocian stage of the Middle Jurassic epoch[187,188]. In this study, we include one fine-grained limestone sample collected near Zimmern ob Rottweil, Baden-Württemberg; this sample contains a fossil of the ammonite species *Garantiana subfurcatum* as well as an abundance of goethite ooids. Sampled ooids are described as spherical goethite ooids in a calcite matrix (Fig. S81). Furthermore, elemental maps suggest the presence of Al and Si within goethite ooids, indicating trace amount of aluminosilicates (Fig. S82).

4.11 Oolithe Ferrugineuse de Bayeux Fm, France (168-170 Ma)

The Oolithe Ferrugineuse de Bayeux Formation is located in the historical Bajocian stratotype at Sainte-Honorine-des-Pertes, Bayeux in Normandy, France. It is thin and condensed, with a thickness of ≈0.5 m[189]. Spanning from the base of the Humphriesianum zone to the Parkinsoni zone, the formation is structured into four beds[190], each marked by the presence of rounded ferruginous oncoids, ferruginous stromatolite pavements, and an abundance of well-sorted ferruginous ooids[191]. Two lines of evidence indicate this succession formed in a deep-marine ramp environment: (i) ooids have been described to be associated with "algal filaments"[189], and (ii) planktonic foraminifera have been detected within individual ooids[191]. Furthermore, Ref.[191] suggested a potential microbial origin for these ooids, although we find no petrographic evidence for microbially induced biomineralization of iron oxides (e.g., diagnostic biogenic iron stalk or stem morphologies or incorporation of EPS)[134,137].

Samples analyzed for this study are characterized by goethite ooids embedded in a carbonate-rich matrix and accompanied by detrital quarts grains (Figs. S83-S84). Furthermore, elemental maps (Figs. S85-S86) suggest the presence of Al and Si within goethite ooids, indicating trace amounts of aluminosilicates.

4.12 Presles Fm, Belgium (372-383 Ma)

Located at the northern fringe of the Dinant Syncline and the southern periphery of the Namur Syncline in Belgium (modern-day Ny, Humian, and Marloie regions), the Presles Formation contains two distinct lithological units: (i) The ≈4 m thick lower unit is composed of clay and bioclastic limestone that are rich in brachiopods and crinoids; it is punctuated by several hematite-rich ooidal ironstone strata. (ii) The ≈6 m thick upper unit is composed of green shale with a low abundance of hematite ooids[192]. Although dominated by hematite, small green chamosite ooids can be found in association with the larger hematite ooids in Belgium's Ny region. However, hematite completely disappears in Humain and Marloie regions, where only ellipsoidal and flattened chamosite ooids are observed[193]. The formation exhibits a very low-grade metamorphism, falling below the anchizone facies[194]. Biostratigraphic analyses—particularly the presence of diagnostic conodont fossils—firmly dates the Presles Formation to the Frasnian stage of the Late Devonian epoch[192].

For this study, we analyzed Presles Formation samples consisting of quartz and hematite ooids embedded in a dolomite-rich matrix (Fig. S87). These samples contain a large number of hematite ooids, with chamosite relics discernable as Si, Al, and Mg enrichments within ooid cores (Figs. S88-S89), indicating chamosite was replaced by hematite during early diagenesis.

4.13 Westmoreland and Kirkland Fms, Clinton Gp, USA (~435-440 Ma)

The Clinton Group is found in the Appalachian Foreland Basin in Pennsylvania and New York, USA. It formed in the aftermath of the Taconic Orogeny and either preceeded or was concurrent with deformation related to the Salinic Disturbance; its stratigraphy is highly regional. Ironstone deposits are thus situated across several formations: Rose Hill and Keefer Formations in Pennsylvania, and Westmoreland, Dawes, and Kirkland Formations in New York. These formations, with a very low metamorphic grade at or below the anchizone facies[195], further subdivided into four distinct packages delineated by unconformities. Brachiopod, ostracod, and conodont biostratigraphy places these ironstone deposits within the late Telychian to early Sheinwoodian ages[195,196].

These ironstones typically exhibit oolitic horizons <0.5 m thick and linked with sandstones marked by flaser bedding, wave ripples, hummocky cross-stratification, and burrows. Ironstone layers are frequently found alongside intraclast breccias containing skeletal fragments and phosphate granules, suggesting storm-driven reworking of the iron ooid horizons[197]. In situ ooidal ironstones are also evident; they are characterized by burrows and phosphate nodules and are assumed to have been deposited in shallow marine shoal settings at or above storm wave base[195,197]. Ooids are chiefly composed of hematite laminae that envelop nuclei of skeletal fragments or quartz grains, although ooids with alternating laminae of chamosite and hematite are also observed in some cases. The absence of noticeable iron depletion in underlying strata implies the source of ferrous iron for ironstone genesis did not stem from a local benthic iron flux[198], but rather by authigenic precipitation of Fe minerals during transgression[199]. Ref.[195] originally proposed that these ironstones formed as authigenic berthierine ooids in suboxic seawater. However, the presence of diverse animal ecosystems and bioturbating organisms indicates oxic bottom water conditions at the time of deposition[197]. Formation of these ironstones is therefore now postulated to have occurred in shallow marine settings along the fringes of a redox-stratified basin, near the confluence of the redoxcline and the seafloor[197].

Samples analyzed for this study were collected from the Westmoreland and Kirkland Formation; they consist of hematite ooids interspersed with traces of berthierine and embedded in a matrix that is predominantly hematitic and siliceous (Fig. S90). This matrix is substantially higher in berthierine content than embedded ooids, suggesting a possible post-deposition—and thus diagenetic—berthierine origin (Figs. S91-S92).

4.14 Skovde Limestone, Gullhogen Fm, Sweden (462-463 Ma)

The 0.2 m thick Skovde Limestone forms a basal component of the Gullhogen Formation, which outcrops in the Gullhogen Quarry in the Vestergotland province of southern Sweden. This calcilutite unit is characterized as a reddish limestone that is densely populated with hematite ooids[200]. Importantly, chamosite ooids are absent from the Skovde Limestone (as observed at Gullhogen Quarry) but can be identified in underlying strata. There exists an unconformity at the limestone's upper boundary; however, this does not result in a sharp lithological transition but rather a progression to gray limestones and mudstones, both of which contain an increased percentage of skeletal grains (i.e, between 3 and 24 %)[201]. The unit is unmetamorphosed and is considered to be well preserved, with limited diagenetic alteration[202]. Furthermore, the Skovde Limestone displays signs of bioturbation and shows pervasive stromatolites. It is thus interpreted to have formed in a marine environment with variable water depth ranging from a deeper sea[203] to a shallow-water setting with intermittent exposure[201]. Biostratigraphy based on cephalopods and ostracodes assigns the bed to the Lasnamägi Stage (Darriwilian age) of the Middle Ordovician[201].

The examined samples in this study feature sub-spherical hematite ooids that appear to be reworked set within a fossil-rich matrix of carbonate cement (Fig. S93). Elemental mapping (Figs. S94-S95) reveals an absence of pronounced Al and Si concentrations within the hematite ooids. Furthermore, a notable difference is observed in Ca concentration between the carbonate matrix and the interspersed fossils.

4.15 Aseri Fm, Estonia (463-464 Ma)

The Aseri Formation of Estonia is composed of bioclastic limestones with unevenly distributed goethite ooids; these ooids are abundant in the upper and lower parts of the formation, although lateral variation does exist[204]. Furthermore, phosphatic ooids can also be identified in western Estonia. The Aseri Formation exhibits a very low-grade metamorphism, falling below the anchizone facies[205], due to the low burial depth and general absence of tectonic activity[206]. Furthermore, extensive biostratigraphic analyses place it in the Aseri Stage[207] of the Middle Ordovician. Specifically, this formation hosts dignostic *Piretella tridactyla* and *Euprimites effusus* ostracods close to its lower boundary[201,208]. The Aseri Formation additionally corresponds to the lower portion of the *Didymograptus murchisoni* graptolite zone and broadly to the *Eoplacognathus suecicus* conodont zone[208].

As evidenced by their fossils assemblage and lithology, these facies were likely deposited in a cold, shallow Ordovician sea characterised by slow carbonate production and a low supply of clastic materials[209]. The ooids analyzed here were collected from northern coastal Estonia[210]. They are described as spherical goethite ooids in a carbonate matrix made of dolomite (Fig. S96). Furthermore, elemental maps (Figs. S97-S98) suggest the presence of Al and Si within the Fe-rich goethite ooids, indicating aluminosilicates.

4.16 Kunda Oolite Bed, Sillaoru Fm, Estonia and Russia (464-467 Ma)

Spread across Estonia and western Russia, the Kunda oolite bed of the Sillaoru Formation comprises glauconitic sandy limestone and dolostone; it is densely filled with goethite ooids that typically nucleate around skeletal or glauconite grains[211]. The Sillariou Formation shows no evidence of metamorphic alteration[212,213]. Goethite ooids are up to 1 mm in diameter and show no evidence of co-precipitated or remplacement chamosite, indicating a primary nature for these ooids. Skeletal fragments are primarily (i.e., 50 to 70 %) comprised of trilobites and ostracods[211]. Despite its thin structure—measuring $\leq$1 m thick—the Kudna Oolite Bed displays a lateral extent of $\approx$1200 km; its eastern extent manifests in outcrops and drill cores from several islands in Lake Lagoda (near St. Petersburg, Russia)[212].

Based on diagnostic Rare Earth Element (REE) patterns of ooids, as well as associated clay mineral assemblages, Ref.[212] posited a volcanic iron source for the Kunda Oolite Bed. Furthermore, diagnostic trilobite biostratigraphy places this formation in the Kundan Stage (Darriwilian Stage) of the Middle Ordovician[204]. Similar to the Aseri Formation, this evidence combined suggests a shallow, cold marine depositional environment for the Kunda Oolite Bed.

The samples examined here were collected in Estonia, along the Baltic–Ladoga Clint (escarpment), over a distance of about 200 km. These goethite ooids have an ellipsoidal shape and display signs of ductile deformation, indicating they were formed prior to early burial (Fig. S99). Additionally, these ooids contain high levels of Cl and Ti and are cemented by carbonate that is occasionally P-rich (Figs. S100-S101). For analytical consistency and accuracy, only undistorted ooids were chosen for further study.

4.17 Šárka Fm, Czechia (∼470 Ma)

Situated within the Bohemian Massif between Prague and Pilsen, Czechia, the Prague Basin forms a core segment of the Teplá-Barrandian Unit[214–217]. This region is characterized by a Neoproterozoic (Cadomian) basement unconformably overlain by Lower Paleozoic (Cambrian to Middle Devonian) volcano-sedimentary sequences[218]. Two prominent formations, the Klabava and the Šárka, contain significant ironstone deposits. The underlying Klabava Formation comprises a sequence of varied volcanogenic rocks and is distinguished by high hematite content and calcareous cement[219]. In contrast, the overlying Šárka Formation is characterized by a consistent series of oolitic ironstones with no interspersed volcanogenic layers[219]. This ironstone is predominantly composed of red hematite and grey chamosite ooids, densely packed within a chamosite and hematite matrix, and is largely absent of pebbles, coarse siliciclastic material, and macrofossils[220]. The Šárka Formation is additionally typified by its prevalence of grey to dark grey shales and siliceous nodules; it exhibits preservation conditions indicative of low metamorphic grades, commonly below the prehnite–pumpellyite phase. This is evidenced by the well-preserved nature of diverse fossils, including trilobites, brachiopods, and bivalves[221].

The appearance of vast oolitic ironstone beds in the Šárka Formation indicates significant shifts in sedimentation conditions at its lower boundary and suggests large-scale shoreline displacement towards the north-west at this time. It is hypothesized[215] that nearby cliffs composed of Precambrian cherts—which were a primary source of pebbles to the Klabava Formation—may have been submerged and subsumed during the initiation of Šárka Formation deposition. This interpretation is consistent with the region's geologic history, which includes significant sea-level shifts and periods of tectonic instability[215]. Furthermore, fossil abundance and assemblages within Šárka Formation ironstones exhibit large differences compared to adjacent volcanogenic rocks of the Klabava Formation. Whereas the latter are rich in shells of orthid brachiopods and hexactine spicules of siliceous sponges[216,222], the former primarily consist of organophosphatic ungulate brachiopods, bryozoan-like biota encrusting pebbles, and fragmented portions of stromatolitic mats[223]. Biostratigraphy of diverse marine fauna, with dominating trilobites, points to a lower Darriwilian stage[224], and a shallow-water, high-energy depositional environment near the paleo-shoreline[225].

Hematite ooids from the Šárka Formation analyzed for this study exhibit signs of plastic deformation, indicating formation prior to early burial. Almost entirely composed of hematite with finely preserved layers, they have a spherical shape ≈0.5 mm in size (Figs. S102-S103) and are embedded within a quartz- and silicate-rich matrix (Figs. S104-S105). Undistorted ooids were targeted for further examination to preserve analytical consistency and accuracy.

4.18 Bliss Fm, USA (491-493 Ma)

The Cambrian to Ordovician Bliss Formation, situated within the Laurentian rifted passive margin, is prominently exposed in southwestern New Mexico, USA. This formation's basal section exhibits a range of lithologies and thicknesses. In particular, Ref.[226] proposed two unmetamorphosed members: (i) a lower member predominantly composed of quartz-rich sandstone and strongly hematitic lithologies, and (ii) an upper member with abundant glauconite and a higher prevalence of carbonates. The lower member is hypothesized to have been deposited in a shallow marine setting based of lithological characteristics[226]. Furthermore, trilobite biostratigraphy (i.e., *Saratogia* and *Rasettia*) assigns a late Cambrian age to this member of the Bliss formation; this is further corroborated by detrital zircon geochronology[227].

Samples analyzed here were specifically collected from two sections in the lower member that are noted for their hematitic ooidal ironstone content and lack of recrystallization[42]. These

spherical hematite ooids are embedded in a ferruginous sandstone (Fig. S106), as evidenced by elemental mapping analysis (Fig. S107).

4.19 Galeros Fm, Chuar Gp, USA (∼750 Ma)

The Galeros Formation is located within well-preserved Tonian strata of the Chuar Group, which are exposed in an expansive, doubly plunging syncline along several tributaries of the Colorado River in the eastern Grand Canyon, Arizona, USA[228]. With a thickness of ≈1600 m, the Chuar Group is predominantly composed of organic-rich shale featuring subordinate meter-scale sandstone and dolomite layers[228,229]. The relative abundance of different iron minerals has been interpreted as evidence of ferruginous seawater dominated basinal conditions[230]. The Chuar Group is partitioned into three unmetamorphosed formations in gradational contact: (i) the basal Nankoweap Formation, (ii) the middle Galeros Formation, and (iii) the overlying Kwagunt Formation[228,229,231]. The Galeros Formation is further divided into: (i) the ironstone-rich Duppa Member, (ii) the Carbon Canyon Member, (iii) the Jupiter member and (vi) the Tanner Member[232].

Within the Galeros Formation, ironstone is found exclusively in the Duppa Member near the contact with the overlying Carbon Canyon Member[233]. With a thickness of ≈30 to 180 m, the Duppa Member is dominated by mudstone with sandstone and limestone interbeds. Sandstones featuring symmetrical ripple marks and mudcracks are common in the upper Galeros Formation[228]. These sedimentary structures indicate an intertidal to subtidal marine setting[228,234]. The primary ironstone lithology is ferruginous sandstone, which is comprised of rounded to subangular quartz grains in a fine-grained hematite matrix and cemented by silica and coarse hematite crystals[232]. This ironstone features a thin (i.e., ≤30 cm) oolite horizon[233] with poorly-cemented beds of iron ooids that are ≈ 0.4 mm in diameter and contain fine, concentric laminae of hematite with variable silica content. Some iron ooids appear to have experienced plastic deformation (Figs. S108-S109), suggesting that they were initially deposited as iron oxyhydroxides and deformed during sedimentation prior to lithification.

The Chuar Group additionally retains diverse microfossil assemblages, including vase-shaped microfossils and acritarchs with complex morphological ornamentation[235,236]. Moving up section, the Kwagunt Formation showcases a continuous decline in acritarch diversity, succeeded by the emergence of vase-shaped microfossils in the upper Awatubi Member that reach their peak abundance in the upper Walcott Member[236]. Detrital zircons have provided a refined age understanding: the Chuar Group in the Grand Canyon has revealed maximum depositional ages of $770.1 \pm 0.5\,\mathrm{Ma}$, with an additional young zircon mode at $775.7 \pm 0.3\,\mathrm{Ma}$[237]. This new dating aligns with earlier U-Pb ages of detrital zircons from basal Nankoweap Formation yield U-Pb ages of $782.0 \pm 6.8\,\mathrm{Ma}$, thus providing a maximum depositional age for the Chuar Group[229]. Additionally, zircons from a tuff within the uppermost Walcott Member of the Kwagunt Formation yield a weighted-mean U-Pb age of $729.0 \pm 0.9\,\mathrm{Ma}$, thus providing a minimum depositional age of the Chuar Group[238]. In addition, Re-Os dating of organic-rich carbonates in the Carbon Canyon Member of the Galeros Formation yield an age of $757.0 \pm 6.8\,\mathrm{Ma}$ whereas Re-Os dating of marcasite nodules in the Awatubi Member yield an age of $751.0 \pm 7.6\,\mathrm{Ma}$[238]; these represent maximum and minimum ages, respectively, for the Duppa Member.

Samples for this study were collected from the Duppa Member; they commonly present a quartz nucleus encapsulated within a matrix comprised of hematite and quartz. Although ooids occasionally display deformation, only undistorted ooids were selected for further examination to preserve analytical consistency and accuracy. The ooids studied here are hematite-rich, with an average size of ≈ 0.4 mm (Figs. S108-S109). They are embedded within a ferruginous

siliceous cement, which is abundant in quartz grains (Figs. S110-S111).

4.20 McClure Fm, Katherine Gp, Canada (~850 Ma)

The Tonian Katherine Group is a siliciclastic succession located in the Mackenzie and Wernecke Mountains on the border of the Yukon and Northwest Territories, Canada. This stratigraphic package is dominated by sandstone with minor amounts of siltstone and mudstone and contains interspersed carbonate horizons; it is believed to have formed in a shallow marine setting[239,240]. The Katherine Group sits above the Dolores Creek Formation, the basal unit of the Mackenzie Mountains Supergroup, and below the Little Dal Group. It is divided into several formations; in ascending stratigraphic order, these are: (i) the Eduni, (ii) Tawu, (iii) Grafe River, (iv) Etagochile, (v) Shattered Range, (vi) McLure, and (vii) Abraham Plains Formations[239].

The ironstone unit of interest for our study is located within the unmetamorphosed McClure Formation of the upper Katherine Group in the Wernecke Mountains, Yukon Territory[239,241]. Three ferruginous intervals—each <5 m thick and thought to represent seafloor hardgrounds—have been identified in this region. These intervals feature hematite-cemented sandstones and hematite-chamosite oncoid/pisoid-bearing ironstones that are interspersed with well-rounded quartz grains and sit within a fine-grained hematite matrix. The oncoids and pisoids—which typically form around intraclastic lithic fragments of laminated hematite siltstone or other pisoids—create distinctive strata. The best-preserved pisoid/oncoid cortices exhibit fine hematite layers that can be locally overlaid by mottled chamosite[239]. Furthermore, these intervals show signs of wetting and drying cycles as indicated by the presence of desiccation cracks and fenestrae. They are primarily interbedded with dark grey mudstones and siltstones that exhibit abundant mudcracks, as well as coarse sandstones that exhibit wave ripples, and are overlain by thick-bedded (>2 m), massive to cross-stratified and highly mature orthoquartzites[239].

Although the Katherine Group stratigraphy in the Yukon Territory remains informally defined, lithostratigraphic profiles—including the presence of characteristic stromatolites in underlying strata—correlate this sedimentary package to the McClure Formation of the Mackenzie Mountains[242]. The McClure Formation, in turn, has been suggested to be directly equivalent to the Aok Formation of the Shaler Supergroup[243]. The maximum age of the Katherine ironstone is constrained by the Re-Os isochron age of $896 \pm 44\,\mathrm{Ma}$ obtained from black shales of the basal Dolores Creek Formation[244]. Furthermore, the Little Dal Basalt, which correlates geochemically with mafic intrusions that intersect the overlying Little Dal Group, provides a minimum age of ~775 Ma[244]. Finally, a large negative carbon isotope anomaly recorded in the Rams Head Formation of the Little Dal Group aligns with the globally contemporaneous Bitter Springs Anomaly dated to ≈811.5 Ma[245,246]. These data combined suggest that the Katherine ironstones were likely deposited at ~850 Ma, although further geochronological constraints are needed to confirm this estimate.

For this study, we hand-picked two varieties of hematite-rich samples collected from McClure Formation ironstones: (i) fine hematite laminae and (ii) hematite grains within oncoids (Figs. S112-S113). These samples are described as large laminated hematite nodules filled with finer iron-silicate cement with sprinkled hematite-rich coated grains (Figs. S114-S115). We interpret these features as early diagenetic mud that was disturbed by currents and consequently became laminated. This mud could have been an ideal site for microbial activity and biofilm formation if these processes were significant contributors to the formation of these rocks. By comparing Fe-OC signals from both hematite varieties, the McClure Formation provides an ideal opportunity to test the importance of EPS incorporation on our Fe-OC signals. Interestingly, Fe-OC loadings and δ^{13}C values from fine hematite laminae and hematite-rich grains

within the matrix are identical within analytical uncertainty ($0.03\,\%$ difference in Fe-OC content and $0.2\,‰$ difference in δ^{13}C value). This result provides further evidence that local EPS incorporation within laminated iron oxide ooids does not drive observed Fe-OC signals.

4.21 Sherwin Ironstone, Sherwin Fm, Roper Gp, Australia (∼1300 Ma)

The Sherwin Ironstone Formation is a constituent of the Roper Group in Northern Australia, a 1 to 5 km thick succession of dominantly unmetamorphosed siliciclastic sedimentary rocks deposited during the Ectasian Period of the Mesoproterozoic Era[247,248]. The Sherwin Ironstone was initially considered to be part of the McMinn Formation, but has since been reclassified as a separate formation within the McMinn sequence[249]. The McMinn sequence directly overlies the Valkerri Formation, which is characterized by deep-water black shale facies. Moving up section within the sequence, the high-energy Moroak Sandstone Formation transitions seamlessly into the Sherwin Ironstone Formation, which consists of crudely bedded to trough cross-stratified sandstones, siltstones, and mudstones with oolitic ironstone lenses[249]. It is ≈10 m thick[248], includes both ooids and pisolites, and is interpreted to have been deposited in a shallow, restricted marine setting[250,251].

A single zircon U-Pb age of $1492 \pm 4\,\mathrm{Ma}$ from the underlying Mainoru Formation[247] and a Rb-Sr age of $1429 \pm 31\,\mathrm{Ma}$ from the overlying Kyalla Formation[252] would place the Sherwin Ironstone Formation at ∼1450 Ma. However, more recent detrital zircon U-Pb geochronology provides a minimum age of $1313 \pm 47\,\mathrm{Ma}$ for the overlying Kyalla Formation but a maximum age of ∼1345 to 1320 Ma for the underlying Moroak Sandstone Formation[253]. Furthermore, the upper Velkerri Formation—which underlies the Moroak Sandstone Formation—yields a whole-rock Re-Os isochron age of $1361 \pm 21\,\mathrm{Ma}$[254]. In light of these recently generated younger ages, it is now estimated that the Sherwin Ironstone Formation was deposited at ∼1300 Ma.

For this study, we sampled unweathered drill cores containing predominantly hematite ooids that display contributions from chamosite or greenalite and are at times bound by diagenetic siderite[249]. The ooids, measuring up to 8 mm in diameter, typically contain a quartz or ooid intraclast nucleus and are surrounded by a quartz-rich matrix[248]. Furthermore, these ooids are interbedded with coarse-grained ferruginous sandstones[251]. In some cases, the matrix is reported to be dominated by the Fe(II) silicates berthierine and greenalite[255]. Nevertheless, samples of this kind were not included in this study as they were absent from our sample set. The ooids under examination are characterized as spherical hematite ooids set within a siliceous and ferruginous matrix interspersed with quartz grains (Figs. S116-S117). Elemental mapping reveals Al- and Fe-rich cores in these ooids, potentially indicating the presence of Fe-rich silicates (Figs. S118-S119).

4.22 Chuanlinggou Fm, Changcheng Gp, China (∼1650 Ma)

Part of the Changcheng Group, the Chuanlinggou Formation constitutes the oldest sedimentary succession in the Yanshan basin, a continental rift on the northern margin of the North China Block[256]. The Changcheng Group dates to ∼1800 Ma and encompasses the Changzhougou, Tuanshanzi, and Dahongyu formations; it is dominated by siliciclastic sedimentary rocks with a metamorphic grade generally below prehnite-pumpellyite facies[257] and partially comprises the Statherian Chuanlinggou ironstone, also known as the Xuanlong-type iron deposit[258]. Within this group, the Chuanlinggou Formation in the Jixian area primarily comprises silty shale interspersed with sandstone, thus indicating deposition in a littoral intertidal marine environment[259]. In particular, the lower section is described by ironstone, silty shale, and interbedded sandstone, which transition upwards into subtidal black shales with frequent intercalations of intertidal

sandstone and dolostone. A distinct 20 m thick ironstone member is present at the base of the formation and is characterized by three main facies: (i) oolitic, (ii) stromatolitic, and (iii) granular, all of which are interbedded with sandstones.

The iron mineralogy of the is ironstone member is predominantly hematite, with minor pyrite and siderite reported in the oolitic facies. Furthermore, the oolitic facies contain coarse sand-sized coated grains typically interspersed with finer quartz sand grains. The iron ooids themselves are commonly spherical to ellipsoidal and range from 0.3 to 4 mm in diameter; however, a small minority display squashed ellipsoidal and irregular dumbbell shapes[42]. Hematite ooids exhibit concentric iron oxide layers; such layers also occur around quartz grains as massive cement. In contrast to the oolitic facies, the stromatolitic ironstone is composed of densely packed, centimeter-scale vertical stromatolite columns that are thought to result from microbial activity. Finally, the granular facies contain medium sand-sized hematite grains lacking internal structure[256].

Two zircon U-Pb geochronology studies estimate a minimum age for the Chuanlinggou Formation of $1625 \pm 6\,\mathrm{Ma}$[260] and $1625.9 \pm 8.9\,\mathrm{Ma}$[261] based on the overlying Dahongyu volcanic rocks in the Jixian area. Refs.[262,263] subsequently refined the age model and report an age of 1700 to 1600 Ma for the entire Changcheng Group. A maximum depositional age for the Chuanlinggou Formation of $1716 \pm 3\,\mathrm{Ma}$ has been estimated from fine-grained sandstones in the middle of the formation[264]. This result is consistent with a U-Pb age of $1657 \pm 17\,\mathrm{Ma}$ for the youngest detrital zircon from the basal Chuanlinggou Formation (determined using laser ablation inductively coupled plasma mass spectrometry, LA-ICP-MS)[265]. Similarly, two estimates of the minimum depositional age for the Chuanlinggou Formation have been generated: $1638 \pm 14\,\mathrm{Ma}$ based on U-Pb dating of a diabase[266] and $1634 \pm 9\,\mathrm{Ma}$ based on diorite porphyporphyrite that intrudes the formation[267]. Finally, recent work based on new U-Pb zircon ages has further constrained the age of the marine oolitic ironstone of the Chuanlinggou Formation to ∼1650 Ma[265]; we adopt this age here.

For this study, we analyzed complete hematite ooids, which often exhibit well-preserved fine layers encircling a quartz nucleus (Figs. S120-S121). The analyzed ooids are described as large (up to 1 mm) spherical hematite ooids that set within a siliceous and ferruginous matrix interspersed with quartz grains (Figs. S122-S123).

5 Geologic sample screening, binning, and intra-period variability

5.1 Sample screening

We employed a screening procedure to exclude samples which may have undergone (post depositional) alterations that affect Fe-OC loadings and δ^{13}C values. Specifically, samples had to meet the following criteria to be included in our record: (i) They are confidently marine in origin; for Phanerozoic samples, this is mainly accomplished by the presence of marine fossils whereas for Precambrian samples this mainly accomplished by consulting literature interpretations based on trace element and isotope compositions, correlation to well-known marine strata, etc. (ii) They contain only iron oxide ooids with no evidence of iron silicate ooids (e.g., chamosite); any samples that contain iron silicate ooids were explicitly excluded, as iron oxides in such samples likely represent pseudomorphs rather than primary precipitates. (iii) They contain pristine, whole ooids (i.e., not cracked or broken); in contrast, cracked or broken ooids may be influenced by later-stage fluid alteration. (iv) They are not clearly impacted by alteration, crystal re-growth, or fluid flow, as assessed by microscopy. Still, while we employed strict protocols for exclusion based on several analytical methods (i.e., microscopy, texture analysis, elemental mapping), such screening is inherently challenging and no screening procedure is

flawless. It therefore remains possible that some samples influenced by diagenetic alteration are included in our final record.

5.2 Correlation with (paleo)latitude

To determine whether observed trends are driven by spatial sampling biases through time, rather than true temporal variability, we assessed our Fe-OC signal results as functions both of modern latitude and paleolatitude at the time of sample deposition (calculated using the Paleolatitude Calculator and additional Refs.[268–272]). First, correlations with modern latitudes would be expected if post-exhumation alteration at the outcrop scale were overprinting primary signals; for example, acid rain in the twentieth century, which was more prevalent in the northern hemisphere, or remineralization of Fe-OC by modern microbial communities, which would be expected to occur faster in the warmer, wetter tropics. In contrast to these concerns, we observe no statistically significant correlation between Fe-OC loadings or δ^{13}C values with modern latitude at the point of sample collection (Fig. S124A-B). Second, correlations with paleolatitudes would be expected if there existed a large latitudinal gradient in DOC signals and if our record exhibits a latitudinal sampling bias through time (e.g., sampling of higher latitude formations in the Proterozoic or vice versa). Although globally averaged DOC concentration is not a strong function of latitude in the modern ocean[273], such concentrations can nevertheless vary spatially by a factor of ≈ 2 (i.e., higher in equatorial upwelling zones, lower in the Antarctic circumpolar current). Similar variations likely existed throughout Earth's history. However, like for modern latitudes, we observe no statistically significant correlation between Fe-OC loadings or δ^{13}C values with paleolatitude at the time of sample deposition (Fig. S124C-D). Additionally, since ocean temperature varies latitudinally, this lack of correlation with latitude (and likely formation water temperature) further supports our conclusion based on Fe-OC synthesis experiments that observed signals are independent of temperature within an environmentally relevant range (see "Effects of co-precipitation procedural manipulations on Fe-OC carbon-loading and ^{13}C fractionation response curves", above). In summary, we conclude that observed signals are not driven by spatial sampling biases, neither at the time of sample deposition nor the time of sample collection.

5.3 Intra-period spatial variability

To estimate the expected variability in Fe-OC signals at any given point in time, we analyzed samples from multiple formations within the same geologic periods spanning spatial distances up to ~ 10000 km. Barring inputs from local point source (e.g., river outflow), we expect intra-period marine DOC concentration and isotope composition to be relatively homogeneous. Thus, if our record does indeed capture global trends, then Fe-OC signals in samples from formations within the same geologic period should exhibit little inter-formation variability. In contrast, if intra-period signal variability is large relative to any observed temporal trends, then such trends could be driven by biases due to local variability (e.g., variations in depositional environment or precipitation mechanism).

In all cases, we observe little inter- or intra-formation spatial variability between samples from the same geologic periods (Fig. S125). Specifically, we examined:

i. *Two modern sites with actively forming iron ooids* (Panarea Island and Mahengetang Island). These sites are separated by $\sim$ 10000 km and thus represent expected spatial variability in the modern ocean. Both sites display similar Fe-OC loadings and δ^{13}C values (Panarea Island: Fe-OC = $0.20 \pm 0.02\,\mathrm{wt}\,\%$, δ^{13}C = $-17.3 \pm 0.6\,‰$, $n = 2$;

Mahengetang Island: Fe-OC = 0.36 ± 0.02 wt %, δ^{13}C = -19.0 ± 1.6 ‰, $n = 2$). Using the mean values for each site thus yields average modern signals of Fe-OC = 0.28 ± 0.12 wt % and δ^{13}C = -18.1 ± 1.2 ‰.

ii. *Two Cretaceous formations across Israel* (Hatira and Hidra Fms). These formations are separated by $\sim$ 100 km, and several samples across the entire outcrop were analyzed for each. In both cases, intra-formation Fe-OC loading and δ^{13}C value variability is reasonably small (albeit larger than analytical uncertainty), and average values for both formations are statistically identical (Hatira: Fe-OC = 0.31 ± 0.13 wt %, δ^{13}C = -21.0 ± 1.5 ‰, $n = 15$; Hidra: Fe-OC = 0.47 ± 0.07 wt %, δ^{13}C = -19.7 ± 1.3 ‰, $n = 20$). As these exhibit the highest sampling density for any formations within our record, we adopt this uncertainty as a reasonable and conservative estimate of intra-formation variability.

iii. *Eight Jurassic formations across Western Europe* (Arroyofrío Bed, Wedelsandstein, Oolithe Ferrugineuse de Villers, Ifenthal, “Humphriesioolith”, “Parkinsoni-Württembergica Schichten”, and Oolithe Ferrugineuse de Bayeux Fms). These formations are separated by $\sim$ 1000 km. With the exception of the Oolithe Ferrugineuse de Bayeux Formation, which exhibits statistically significantly higher Fe-OC loadings, all other formations display similar Fe-OC loadings and δ^{13}C values (Arroyofrío Bed: Fe-OC = 0.41 ± 0.04 wt %, δ^{13}C = -22.4 ± 0.3 ‰, $n = 2$; Wedelsandstein: Fe-OC = 0.47 ± 0.01 wt %, δ^{13}C = -25.4 ± 0.6 ‰, $n = 2$; Oolithe Ferrugineuse de Villers: Fe-OC = 0.44 ± 0.01 wt %, δ^{13}C = -21.7 ± 0.3 ‰, $n = 2$; Ifenthal: Fe-OC = 0.32 wt %, δ^{13}C = -21.0 ‰, $n = 1$; Humphriesioolith: Fe-OC = 0.38 ± 0.06 wt %, δ^{13}C = -22.7 ± 0.9 ‰, $n = 5$; Parkinsoni-Württembergica Schichten: Fe-OC = 0.53 ± 0.12 wt %, δ^{13}C = -24.8 ± 1.0 ‰, $n = 2$; Oolithe Ferrugineuse de Bayeux: Fe-OC = 0.93 ± 0.04 wt %, δ^{13}C = -24.6 ± 0.1 ‰, $n = 2$). Using the mean values for each formation (Omitting the Oolithe Ferrugineuse de Bayeux) thus yields average Jurassic signals of Fe-OC = 0.43 ± 0.07 wt % and δ^{13}C = -23.0 ± 1.7 ‰ (when including the Oolithe Ferrugineuse de Bayeux, average values change to Fe-OC = 0.50 ± 0.20 wt % and δ^{13}C = -23.2 ± 1.7 ‰). This inter-formation variability is similar to the intra-formation variability observed for the Cretaceous, further supporting the use of these values as reasonable uncertainty estimates for a given geologic period.

iv. *Four Ordovician formations across northern and eastern Europe* (Skovde Limestone, Aseri, Šárka, Sillarou Fms). This includes two goethite- (Aseri, Sillarou Fms) and two hematite-containing (Skovde Limestone, Šárka Fm) formations separated by $\sim$ 100 km. All formations yield statistically identical Fe-OC loadings, although geothite-containing samples may display slightly higher δ^{13}C values than hematite-containing samples (Skovde Limestone: Fe-OC = 0.33 ± 0.04 wt %, δ^{13}C = -28.6 ± 1.1 ‰, $n = 3$; Aseri: Fe-OC = 0.21 ± 0.01 wt %, δ^{13}C = -26.1 ± 2.3 ‰, $n = 4$; Šárka: Fe-OC = 0.16 ± 0.01 wt %, δ^{13}C = -28.8 ± 0.3 ‰, $n = 2$; Sillarou: Fe-OC = 0.22 ± 0.03 wt %, δ^{13}C = -24.1 ± 0.7 ‰, $n = 3$). Using the mean values for each formation (combining goethite- and hematite-containing formations) yields average Ordovician signals of Fe-OC = 0.23 ± 0.07 wt % and δ^{13}C = -26.9 ± 2.2 ‰ (when separated by mineralogy, δ^{13}C uncertainty reduces to ± 1.4 ‰ for goethite- and ± 0.1 ‰ for hematite-containing samples). This uncertainty is again similar to the intra-formation variability observed for the Cretaceous.

v. *Two Tonian formations across North America* (Galeros and McClure Fms). These formations, separated by $\sim$ 1000 km, yield similar Fe-OC signals (Galeros: Fe-OC = 0.05 ± 0.01 wt %, δ^{13}C = -27.0 ± 0.1 ‰, $n = 2$; McClure: Fe-OC = 0.07 ± 0.02 wt %,

$\delta^{13}C = -28.1 \pm 0.2‰$, $n = 2$). Using the mean values for each formation yields average Tonian signals of Fe-OC = $0.06 \pm 0.01\,wt\,\%$ and $\delta^{13}C = -27.6 \pm 0.8‰$, again similar to—although slightly smaller than—intra-formation variability observed for the Cretaceous.

In summary, we estimate that spatial variability within and between formations from a given geological period will yield Fe-OC loading and $\delta^{13}C$ signal uncertainty of $\sim 0.07\,wt\,\%$ and $\sim 1.5‰$, respectively. We conclude that any temporal trends larger than this variability are unlikely to be a result of spatial heterogeneity.

5.4 Time-binning

Given the observed lack of intra-period spatial variability, we adopted a time binning strategy of 100 Myr to comprehensively represent data for each geologic period; the underlying hypothesis is that variations in marine DOC concentration and $\delta^{13}C$ values are negligible over this time window. In adopting this approach, we conservatively propagate uncertainty from data-rich periods to data-poor periods of Earth's history. Specifically, if uncertainty within a given period is small—or if a period is represented by a lone sample with only analytical error—then we instead assign a more conservative uncertainty value of $\pm 0.07\,wt\,\%$ and $\pm 1.5‰$ for Fe-OC loadings and $\delta^{13}C$ values, as calculated based on observed spatial variability for data-rich periods.

6 Estimating DOC concentrations and $\delta^{13}C$ values from iron ooid Fe-OC

We developed a Monte Carlo inverse model framework to estimate seawater DOC concentrations and $\delta^{13}C$ values through geologic time—including uncertainty—using our ooid Fe-OC record (Fig. 2) combined with our experimental Fe-OC carbon-loading and ^{13}C-fractionation response curves (Figs. S18-S19). This exercise requires five simplifying assumptions:

i. *Marine DOC at all points in geologic time is described by a mixture of modern-marine-like, cyanobacterially derived, or terrestrial soil-like DOC end members* (i.e., our M-DOC, C-DOC, and FA experimental materials). This treatment inherently ignores other potential sources of compositionally unique DOC that are not captured by these end members; for example, black carbon from hydrothermal inputs[155,156] or sulfurized DOC[274,275].

ii. *Iron oxide ooids represent primary or early secondary marine precipitates that have not undergone transformation during post-burial diagenesis* (here termed "open system formation"). This assumption is required to apply our experimental goethite and hematite Fe-OC calibration curves to natural samples. In contrast, if ooids—particularly hematite ooids—represent post-burial diagenetic alteration of primary precipitates such as iron-rich silicates (here termed "closed system transformation"), then the application of our calibration curves could lead to biased interpretations. This assumption is supported by our screening protocol, which aims to remove samples that may have undergone post-depositional mineral transformation (see "Geologic sample screening, binning, and intra-period variability", above).

iii. *Fe-OC loadings and isotope compositions reflect a weighted average of signals derived from each end member.* That is, we assume the total co-precipitated DOC can be treated as a superposition of co-precipitation loadings for individual end members. This treatment inherently ignores any potential interactions between compounds derived from different end members that may impact resulting Fe-OC signals. A corollary of this assumption

is that all co-precipitated OC in geologic iron-oxide ooids is contained within the oxide phase and is not associated with accessory phases such as silicates or quartz, which cannot be analytically separated.

iv. *Fe(II) flux at the sites of iron ooid formation has remained constant through geologic time.* Because Fe(II) is oxidized to Fe(III) when exposed to oxygenated water, this flux will control local Fe(III) concentrations at the site of ooid formation. Importantly, this is not equivalent to assuming that *open-ocean dissolved iron* concentrations have remained constant through time, which are known to decrease as the ocean becomes progressively oxygenated [e.g., Ref.[24,276]]. Rather, our assumption requires that the flux of upward-diffusing iron from upwelling, microbial, and/or hydrothermal processes in marine sediments does not display a secular trend. This assumption allows us to convert measured DOC/Fe(III) concentration ratios into predicted DOC concentrations; a corollary states that actively precipitating iron ooids in the modern ocean accurately capture the modern DOC/Fe(III) concentration ratio at the site of formation.

v. *At a given point in geologic time, DOC derived from different end members exhibits the same isotope composition.* This assumption is supported by ^{13}C surveys of modern DOC, which show ≈ 1 ‰ variability of average DOC $\delta^{13}C$ values across a range of environments—including coastal surface ocean, continental shelf surface ocean, North Atlantic deep waters, and oligotrophic tropical Pacific deep waters—despite large changes in DOC concentration and source contributions[52,53]. Such limited isotopic variability in the modern ocean further supports our interpretation of iron ooid Fe-OC $\delta^{13}C$ values as recorders of open-ocean DOC isotope compositions. However, this assumption can fail if there was an admixture of isotopically unique end members in the geologic past, in particular chemolithoautotrophy by non-canonical (i.e., non-Calvin–Benson–Bassham) carbon fixation pathways[277].

Given these assumptions, we generate geologic DOC concentration and $\delta^{13}C$ records with only one free parameter—the fractional contribution of each end member at each point time. Below, we first mathematically derive our model before describing six possible fractional contribution scenarios. Finally, we discuss the validity of our simplifying assumptions, including potential implications if they do not hold.

6.1 Mathematical derivation

DOC concentrations. As described above, Fe-OC loadings follow power-law functions with respect to the initial solution DOC/Fe(III) concentration ratio (Figs. S18-S19; see section "Building Fe-OC carbon-loading and ^{13}C fractionation response curves"). For time-dependent loadings, this can be written mathematically as

$$w^{i,m}(t) = \beta_0^{i,m} \left[x^i(t)\right]^{\beta_1^{i,m}}, \tag{S3}$$

where $w^{i,m}(t)$ is the Fe-OC loading (in wt. %) of DOC end-member i onto iron-oxide mineral m at time t; $\beta_0^{i,m}$ and $\beta_1^{i,m}$ are empirically fit power-law constants (Figs. S20-S21, Table S2); $x^i(t)$ is the DOC/Fe(III) concentration ratio ($\mathrm{mol\,mol^{-1}}$) for compounds derived from end-member i at time t; i = M-DOC, C-DOC, or FA according to assumption (i); and m = goethite or hematite. Assumption (ii) states that $x^i(t)$ values calculated in Eq. S3 represent (coastal) marine rather than post-diagenetic transformation signals. Following assumption (iii), we then

treat the total Fe-OC loading on a given ooid as a superposition of power-law functions for individual DOC end members such that

$$W^m(t) = \sum_i w^{i,m}(t). \tag{S4}$$

We similarly treat the total amount of DOC in the ocean—and thus the total DOC/Fe(III) concentration ratio—as the sum of DOC derived from individual end members such that

$$X(t) = \sum_i x^i(t). \tag{S5}$$

Eq. S5 can alternatively be written as

$$1 = \sum_i f^i(t), \tag{S6}$$

where

$$f^i(t) = x^i(t)/X(t) \tag{S7}$$

is the fractional contribution of end member i at time t such that $f^i(t) \in (0,1)$ (i.e., the sum-to-unity constraint). Substituting Eqs. S3 and S7 into Eq. S4 yields a prediction for total Fe-OC loading as a function of total DOC/Fe(III) concentration ratio in seawater:

$$W^m(t) = \sum_i \beta_0^{i,m} \left[f^i(t)X(t)\right]^{\beta_1^{i,m}}. \tag{S8}$$

Finally, since Fe(III) concentrations at both modern ooid formation sites remain unknown, we estimate DOC concentrations through time by normalizing $X(t)$ to the DOC/Fe(III) concentration ratio of the modern ocean at the site(s) of actively precipitating iron ooids. We define

$$[\text{DOC}]^*(t) = \frac{[\text{DOC}](t)}{[\text{DOC}](t_0)}, \tag{S9}$$

where t_0 is the modern day. Following assumption (iv), we treat Fe(III) concentration at the site of ooid formation as constant through time such that $[\text{Fe(III)}](t_0) = [\text{Fe(III)}](t) = [\text{Fe(III)}]$. Eq. S9 can thus be rewritten as

$$\begin{aligned} [\text{DOC}]^*(t) &= \frac{\frac{[\text{DOC}](t)}{[\text{Fe(III)}]}}{\frac{[\text{DOC}](t_0)}{[\text{Fe(III)}]}} \\ &= \frac{X(t)}{X(t_0)}. \end{aligned} \tag{S10}$$

We constrain $X(t_0)$ using our measured $W^m(t_0)$ values for actively precipitating goethite ooids, and we assume that all DOC at these sites is derived from M-DOC, i.e., $f^{\text{FA}}(t_0) = f^{\text{C-DOC}}(t_0) = 0$ and $f^{\text{M-DOC}}(t_0) = 1$. This assumption is consistent with the known dominance of eukaryotic primary producers in modern coastal water settings. We can thus analytically solve Eq. S8 for $X(t_0)$, which yields

$$X(t_0) = \left[\frac{W^{\text{goethite}}(t_0)}{\beta_0^{\text{M-DOC,goethite}}}\right]^{-\beta_1^{\text{M-DOC,goethite}}}. \tag{S11}$$

Finally, substituting Eq. S10 into Eq. S8 yields

$$W^m(t) = \sum_i \beta_0^{i,m} \left[f^i(t)X(t_0)[\text{DOC}]^*(t)\right]^{\beta_1^{i,m}}. \tag{S12}$$

Eqs. S11 and S12 describe our marine DOC concentration model.

DOC carbon-isotope compositions. Marine DOC ^{13}C compositions can be calculated as

$$\delta(t) = \delta^m(t) + \Delta^m(t), \tag{S13}$$

where $\delta(t)$ is the δ^{13}C value of marine DOC at time t, $\delta^m(t)$ is the bulk δ^{13}C value of Fe-OC in iron-oxide mineral m at time t, and $\Delta^m(t) = \delta(t) - \delta^m(t)$ is the δ^{13}C offset between these two reservoirs. Notably, $\Delta^m(t)$ is approximately equal to the ^{13}C fractionation factor, $^{13}\varepsilon$, but we retain the former notation here since the latter is reserved to describe a single, specific process or mechanism, which may hold true for Fe-OC co-precipitation (i.e., there may be several chemically unique mechanisms operating in parallel). Following assumption (v), we treat $\Delta^m(t)$ as an average of δ^{13}C offsets for different end members weighted by their proportional contributions to the total Fe-OC. That is, we write

$$\Delta^m(t) = \frac{1}{W^m(t)} \sum_i w^{i,m}(t)\Delta^{i,m}(t), \tag{S14}$$

where $\Delta^{i,m}(t)$ is the δ^{13}C offset for end member i at time t. Unlike Fe-OC loadings, ^{13}C offsets follow dual exponential functions with respect to the initial solution DOC/Fe(III) concentration ratio (Figs. S18-S19; see section "Building Fe-OC carbon-loading and ^{13}C fractionation response curves"). This can be written mathematically for time-dependent offsets as

$$\Delta^{i,m}(t) = \beta_2^{i,m} \exp\left[\beta_3^{i,m} x^i(t)\right] + \beta_4^{i,m} \exp\left[\beta_5^{i,m} x^i(t)\right] \tag{S15}$$

where $\beta_2^{i,m}$, $\beta_3^{i,m}$, $\beta_4^{i,m}$, and $\beta_5^{i,m}$ are empirically fit dual exponential constants (Figs. S20-S21, Table S2). Substituting Eqs. S3, S14, and S15 into Eq. S13 yields

$$\delta(t) = \delta^m(t) + \frac{1}{W^m(t)} \sum_i \left\{\beta_0^{i,m} \left[x^i(t)\right]^{\beta_1^{i,m}}\right\} \left\{\beta_2^{i,m} \exp\left[\beta_3^{i,m} x^i(t)\right] + \beta_4^{i,m} \exp\left[\beta_5^{i,m} x^i(t)\right]\right\}. \tag{S16}$$

Finally, utilizing the definitions of $f^i(t)$ (Eq. S7) and [DOC]$^*(t)$ (Eq. S10), Eq. S16 can be rewritten as

$$\begin{aligned}\delta(t) = \delta^m(t) + \frac{1}{W^m(t)} \sum_i \left\{\beta_0^{i,m} \left[f^i(t)X(t_0)[\text{DOC}]^*(t)\right]^{\beta_1^{i,m}}\right\} \times \\ \left\{\beta_2^{i,m} \exp\left[\beta_3^{i,m} f^i(t)X(t_0)[\text{DOC}]^*(t)\right] + \beta_4^{i,m} \exp\left[\beta_5^{i,m} f^i(t)X(t_0)[\text{DOC}]^*(t)\right]\right\}.\end{aligned} \tag{S17}$$

Eqs. S11, S12, and S17 describe our marine DOC δ^{13}C model.

6.2 Possible $f^i(t)$ scenarios

The only free parameter in our marine DOC concentration and δ^{13}C model is $f^i(t)$, the fractional contribution of each end member to total marine DOC at time t. To solve our model and to explore sensitivity of predicted DOC concentrations and δ^{13}C values to this free parameter, we examine six different $f^i(t)$ evolution scenarios (Fig. S126). Specifically, we consider:

i. *Cyanobacteria only.* We first consider the case where all DOC is derived from cyanobacteria throughout geologic time. In terms of our experimental end member materials, this is equal to setting $f^{\text{C-DOC}}(t) = 1$ and $f^{\text{M-DOC}}(t) = f^{\text{FA}}(t) = 0$ for all t (Fig. S126A). Although this scenario is inconsistent with the known importance of eukaryotic primary producers in marine ecosystems since at least $\sim$780 Ma[54, 278], it is nevertheless a useful end-member scenario to bracket the sensitivity of model results to prescribed DOC sources and thus molecular compositions. Furthermore, we [state we test absence and concentration of silica for this scenario]

ii. *Modern-marine analog only.* Second, we consider the case where all DOC is derived from modern-marine-like eukaryotic sources throughout geologic time. In terms of our experimental end member materials, this is equal to setting $f^{\text{M-DOC}}(t) = 1$ and $f^{\text{C-DOC}}(t) = f^{\text{FA}}(t) = 0$ for all t (Fig. S126B). Although this scenario is inconsistent with evidence for the importance of cyanobacterial primary production throughout much of the Proterozoic[54,278], it is again a useful end-member scenario to bracket the sensitivity of model results to prescribed DOC sources and thus molecular compositions, similar to the "cyanobacteria only" scenario, above.

iii. *Soil humics only.* For completion, we next consider a scenario where all DOC is derived from soil humic-like substances throughout geologic time. In terms of our experimental end member materials, this is equivalent to setting $f^{\text{FA}}(t) = 1$ and $f^{\text{M-DOC}}(t) = f^{\text{C-DOC}}(t) = 0$ for all t (Fig. S126C). This scenario is inconsistent with the known evolutionary history of land plants, which first appeared in the Devonian and only evolved modern-like vascular structures—including deep rooting networks and accompanying organic-acid-rich soil profiles—in the Carboniferous[279]. Nevertheless, this scenario remains useful for two reasons: (i) to bracket the sensitivity of our results to prescribed DOC molecular compositions, as above, and (ii) to constrain the the potential impact of possible inputs from terrestrial DOC runoff, particularly since the Carboniferous.

iv. *Unconstrained $f^i(t)$ (minimize propagated error).* Fourth, we find the $f^i(t)$ values that minimize the propagated Monte Carlo error on predicted [DOC]$^*(t)$ and $\delta(t)$ at each t. For this scenario, we only consider C-DOC and M-DOC contributions (i.e., $f^{\text{FA}}(t) = 0$ for all t). We then solve the minimization function

$$\min_{f^i(t)} \sqrt{\sigma^2_{[\text{DOC}]^*}[f^i(t)] + \sigma^2_{\delta}[f^i(t)]}, \tag{S18}$$

where $\sigma^2_{[\text{DOC}]^*}[f^i(t)]$ and $\sigma^2_{\delta}[f^i(t)]$ are the Monte Carlo propagated variances of [DOC]$^*(t)$ and $\delta(t)$ estimates as functions of $f^i(t)$, subject to the sum-to-unity constraint of Eq. S6 (Fig. S126D). This scenario implicitly assumes that the solution with the lowest propagated error—which includes analytical and experimental uncertainty—is the most geologically feasible. While this is likely not true (e.g., solutions can be biased by larger uncertainty in a given co-precipitation experiment, which is unrelated to geologic signals), this scenario is nevertheless useful to explore the structure of $f^i(t)$ evolution in a purely unconstrained model.

v. *Rise of algae.* This scenario follows a prescribed microorganism evolution in which cyanobacteria dominated marine primary production until $\sim$780 Ma, after which eukaryotes grew in importance and came to dominate (Fig. S126E). In terms of our experimental end member materials, this is equivalent to setting $f^{\text{FA}}(t) = 0$ for all t; $f^{\text{C-DOC}}(t \geq 780\text{ Ma}) = 0.95$, then linearly decreasing to $f^{\text{C-DOC}}(t \leq 500\text{ Ma}) = 0.05$; and $f^{\text{M-DOC}}(t \geq 780\text{ Ma}) = 0.05$, then linearly increasing to $f^{\text{M-DOC}}(t \leq 500\text{ Ma}) = 0.95$. Both $f^{\text{C-DOC}}$ and $f^{\text{M-DOC}}$ are allowed to vary randomly by $\pm 5\,\%$ at all t. This scenario is consistent with biomarker (e.g., *n*-alkyl membrane lipid and steroid) records that have been interpreted as evidence for the dominance of cyanobacterial primary producers—which presumably sink slowly due to their small cell size and thus release significant DOC in the surface ocean—before the Cryogenian period, followed by a rapid shift to dominance by eukaryotic primary producers—which presumably sink rapidly due to their larger cell size and thus remove OC from the surface ocean—in the Phanerozoic [e.g., Refs.[23,48,280]]. This

presumed dominance of slowly sinking cyanobacteria accompanied by DOC release for much of the Proterozoic was central to the initial development of the "large DOC reservoir" hypothesis of Ref.[6]. Although the oldest known (putatively stem-group) eukaryotic body fossils date at least to $1650\,\mathrm{Ma}$ Ma[281] and major eukaryotic clades likely diverged by $1300\,\mathrm{Ma}$ Ma[54], this scenario implicitly assumes that these organisms remained unimportant in terms of biomass and primary production for nearly one billion years.

vi. *Proterozoic active eukaryotes.* Finally, we consider a recently hypothesized scenario in which eukaryotes comprise at least half of total marine biomass—and eukaryotic algae account for approximately half of marine primary production—throughout much of the Proterozoic (Fig. S126F)[49]. In terms of our experimental end member materials, this is equivalent to setting $f^{\text{FA}}(t) = 0$ for all t; $f^{\text{C-DOC}}(t = 1780\text{ Ma}) = 0.50$, then linearly decreasing to $f^{\text{C-DOC}}(t \leq 500\text{ Ma}) = 0.05$; and $f^{\text{M-DOC}}(t = 1780\text{ Ma}) = 0.50$, then linearly increasing to $f^{\text{M-DOC}}(t \leq 500\text{ Ma}) = 0.95$. As with the "rise of algae" case, both $f^{\text{C-DOC}}$ and $f^{\text{M-DOC}}$ are allowed to vary randomly by $\pm 5\,\%$ at all t. This scenario is consistent with the recent discovery of abundant so-called "proto-steroids" in sedimentary rocks throughout the Mesoproterozoic, which are chemically distinct from steroids produced by crown-group eukaryotes and therefore previously escaped detection and investigation[50]. This discovery implies the existence of Proterozoic ecosystems containing abundant and diverse stem-group eukaryotes that lacked a complete sterol biosynthesis pathway. This idea is further supported by a recent model of marine ecosystem structure that reconstructs the size distribution of preserved eukaryotic body fossils[49]. According to this model, observed Mesoproterozoic body fossil size distributions require that eukaryotic photosynthesis had already evolved and proliferated as early as $1600\,\mathrm{Ma}$. This scenario reconciles the well-known antiquity of (putatively stem group) eukaryotic body fossils with the lack observable sterane biomarkers prior to $\sim 780\,\mathrm{Ma}$[48, 281].

6.3 Monte Carlo solution

To estimate marine DOC concentrations and ^{13}C compositions—including uncertainty—through geologic time, we solve Eqs. S11, S12, and S17 in Monte Carlo fashion for each of the six $f^i(t)$ scenarios described above in five steps:

i. First, we solve Eq. S11 for $X(t_0)$ at both of our modern sites—Panarea Island, Italy and Mahengentang Island, Indonesia—using measured $W^{\text{goethite}}(t_0)$ values and experimental estimates of $\beta_j^{\text{M-DOC,goethite}}$, where $j = 0, 1$. We calculate the average of $X(t_0)$ determined at both sites and use this as input to our Earth-history model.

ii. Second, we solve Eq. S12 for $[\text{DOC}]^*(t)$ using our measured geologic iron ooid $W^m(t)$ results, averaged into 100 Myr time bins; calculated $X(t_0)$ from step (i); experimental estimates of each $\beta_j^{i,m}$, where $j = 0, 1$; and prescribed $f^i(t)$ values (for all but scenario 4; see below).

iii. Third, we solve Eq. S17 for $\delta(t)$ using our measured geologic iron ooid $W^m(t)$ and $\delta^m(t)$ results, averaged into 100 Myr time bins; calculated $X(t_0)$ from step (i); experimental estimates of each $\beta_j^{i,m}$, where $j = 0, \ldots, 5$; prescribed $f^i(t)$ values (for all but scenario 4; see below); and $[\text{DOC}]^*(t)$ estimates from step (ii).

iv. Fourth, we repeat steps (i) to (iii) 1,000,000 times, randomly adding error to each input parameter as follows:

- $W^m(t)$ [including $W^{\text{goethite}}(t_0)$] and $\delta^m(t)$: pull from Gaussian distributions with standard deviations equal to those calculated for time-binned samples within each 100 Myr period (see "Geologic sample screening, binning, and intra-period variability", above).
- $\beta_j^{i,m}$ ($j = 0, \ldots, 5$): Re-calculate regression fits to experimental data using Eqs. S1-S2, including error pulled from Gaussian distributions with standard deviations equal to analytical error for each point of each calibration curve in Figs. S18-S19. This inherently accounts for any covariance between $\beta_j^{i,m}$ values (Figs. S20-S21).
- $f^i(t)$: For scenarios 5 and 6 only, pull from uniform distributions than span $\pm 5\,\%$ of the prescribed value. For scenario 4 only, repeat steps (ii)-(iii) for different $f^i(t)$ values (step size of 0.05 units) and solve Eq. S18; retain the resulting $f^i(t)$ that minimizes error as the "best fit" result.

v. Finally, average resulting [DOC]$^*(t)$ and $\delta(t)$ values [and retained $f^i(t)$ values for scenario 4 only] and calculate summary statistics for each 100 Myr time bin ($\pm 1\sigma$, interquartile ranges, $95\,\%$ confidence intervals).

6.4 Sensitivity tests and validity of assumptions

We assess the validity of each assumption in detail, including calculating useful end-member scenarios and sensitivity tests:

Assumption (i). Possible end-member sources. We assume that M-DOC, C-DOC, and FA (or molecularly similar material) are the only possible DOC sources to iron ooids throughout our record; this assumption is required to utilize our experimental Fe-OC calibration curves to reconstruct DOC concentrations and δ^{13}C values. In contrast, molecularly unique DOC sources such as black carbon from hydrothermal inputs[155,156] or sulfurized DOC[274,275] could exhibit unique Fe-OC calibration curves that are not captured here. Quantitative importance of such material would thus bias our reconstructions. Despite this concern, additional DOC sources are unlikely to qualitatively change the interpretation of our record for two reasons.

First, our three experimental DOC materials exhibit an EEMS slope ratio (S$_R$) range that captures most variability observed in modern coastal to continental shelf waters[40]. Because S$_R$ exhibits a strong control on Fe-OC loadings (Fig. S23), our chosen DOC sources likely span the expected range of loadings and thus calculated DOC concentrations for a given measured $\text{wt}\,\%$ Fe-OC. Although significantly higher S$_R$ values may be possible for some DOC end members (e.g., highly photo-bleached material), S$_R$ estimates for sources such as black carbon and sulfurized DOC remain unknown to us. Future work is thus needed to estimate the quantitative impact of these DOC sources on Fe-OC signals. Nevertheless, higher S$_R$ values would lead to lower Fe-OC loadings for a given DOC/Fe(III) ratio (Fig. S23); incorporation of this material would then lead to higher calculated DOC concentrations for a given measured $\text{wt}\,\%$ Fe-OC.

Second, our reconstructed Earth history DOC concentration and δ^{13}C trends are largely insensitive to the chosen calibration curve [i.e., the six $f^i(t)$ scenarios considered here] as well as the presence and concentration of dissolved silica in the "cyanobacteria only" scenario (Fig. S127). Specifically, average predicted DOC concentrations never differ at any point in our record by more than a factor of ≈ 2 between scenarios, and results for all scenarios are always statistically identical (Fig. S127A). Similarly, average predicted δ^{13}C values in the Phanerozoic never differ by more than $\approx 8\,‰$ between scenarios, and results for all scenarios are again statistically identical (Fig. S127B). Although larger differences up to $\approx 15\,‰$ can exist between

scenarios in the Proterozoic (i.e., between cyanobacteria only and soil humics only), a general rise toward modern values starting in the Cambrian is observed for all scenarios. Thus, while incorporation of additional end members not considered here could lead to small quantitative shifts in our record, this is unlikely to impact the general interpreted trajectory of DOC concentrations and ^{13}C compositions through geologic time.

Assumption (ii). Iron ooids as primary or early secondary marine precipitates. We assume that all iron (hydr)oxides either precipitate directly in seawater or form as early oxidation products of iron silicates (e.g., chamosite) at the sediment-water interface. In contrast, if iron ooids represent later-stage diagenetic products, then Fe-OC signals cannot be reliably interpreted as marine DOC proxies. This assumption must be satisfied for goethite ooids, since these are observed to form directly in the modern ocean[43,44], but is less constrained for hematite ooids. We thus consider two possible formation pathways:

Open system formation. In this scenario, iron oxides either precipitated directly in seawater or underwent transformation from initial iron-silicates through dissolution and re-precipitation on the seafloor. Importantly, early mineralogical transitions from chamosite have been documented[282–285] and follow the general reaction (here written for hematite, similar reactions apply for goethite formation)

$$\underbrace{(\mathrm{Mg,Fe})_5\mathrm{Al(Si_3Al)O_{10}(OH)_8}}_{\text{chamosite}} + \mathrm{O_2} \longrightarrow 2\,\underbrace{\mathrm{Fe_2O_3}}_{\text{hematite}} + \mathrm{MgO} + \mathrm{Al_2O_3} + 3\,\mathrm{SiO_2} + 4\,\mathrm{H_2O}. \quad \text{(S19)}$$

If this dissolution and re-precipitation mechanism occurs shortly after sediment deposition on the seafloor, then preserved Fe-OC signals in resulting iron oxides should still reflect a primary seawater signature. Petrographic analysis suggests that some hematite-bearing ooid samples in our record likely underwent this mineralogical transformation. For example, the Devonian aged Presles Fm, Belgium contains hematite ooids embedded in a matrix of silicified dolomite with cores enriched in Al, Mg, and Si relative to their outer rims (Fig. S89). This indicates a primary Fe(II)-bearing silicate core that was subsequently oxidized and transformed. In contrast, rims may have precipitated directly as hematite on the ocean floor, suggesting that core transformation indeed occurred early and that Fe-OC signals reflect primary marine DOC signatures. Furthermore, results argue against dissimilatory iron reduction, which would instead lead to a hematite core surrounded by Fe(II)-bearing silicate outer rims (or Al-, Mg-, and Si-enriched oxide rims if Fe(II)-bearing silicates were subsiquently re-oxidized). Additionally, such reduction (with or without re-oxidation) would likely erase finely laminated petrographic structures, particularly in outer rims. However, neither phenomenon is ever observed, thus precluding dissimilatory iron reduction in any sample studied here. This scenario therefore satisfies our assumption.

Closed system transformation. In this scenario, iron silicates transform into oxides only after sediment is buried and disconnected from the open ocean. We consider this scenario unlikely as it would require either (i) Fe(III)-bearing primary iron silicates (e.g., nontronite), inconsistent with the presence of Mg in ooid cores, which instead suggests Fe(II)-bearing iron silicates (e.g., chamosite); or (ii) significant downward flux of O_2 or other oxidant into deep sediments (i.e., to satisfy Eq. S19)[286], inconsistent with known bottom- and pore-water hypoxia—and thus sub-centimeter sediment O_2 penetration depths—in the Precambrian. Nevertheless, closed system transformation would imply that our experimental hematite Fe-OC calibration curves do not apply. Rather, Fe-OC loadings and δ^{13}C values would be governed by calibration curves for the primary mineral (e.g., chamosite). Organic compounds would then be retained as co-precipitated Fe-OC after closed-system transformation; preserved hematite Fe-OC signals

would still be governed by primary marine signatures, but via an iron silicate intermediate. Reconstructed DOC concentrations and δ^{13}C values using our hematite calibration curves could therefore be systematically biased. This scenario does not quantitatively satisfy our assumption but should still lead to qualitatively meaningful temporal Fe-OC trends.

Results imply either that the "open system formation" scenario applies to all samples in our record or that the "closed system transformation" scenario does not lead to meaningful biases (i.e., nearly identical chamosite and hematite calibration curves). This is evidenced by the fact that goethite and hematite Fe-OC signals yield statistically identical reconstructed DOC signatures—particularly concentrations—during geologic periods in which both mineralogies overlap (Fig. S127). Furthermore, our petrographic and Raman spectroscopic observations—i.e., the preservation of fine laminations and uniform Fe-OC distributions—support an interpretation in which Fe-OC signals predominantly record primary seawater DOC rather than late-stage diagenesis (e.g., dissolution and recrystallization, potentially memetic in nature), post-depositional microbial reworking, or detrital POC inputs. We therefore interpret Fe-OC in both minerals as reflecting marine signals, but we cannot definitively exclude the possibility that samples have undergone some degree of diagenetic alteration, potentially impacting Fe-OC trends.

Assumption (iii). Superposition of source contributions. This assumption is necessary to convert Fe-OC signals into marine DOC concentrations and δ^{13}C values for all but the single-end-member scenarios. Although we did not explicitly perform synthesis experiments with M-DOC, C-DOC, and FA mixtures, each tested DOC source is itself a mixture of several compounds (with the exception of FA). The observation that Fe-OC signals are qualitatively similar between sources supports the hypothesis that interactions between organic molecules will not change the overall co-precipitation behavior. Furthermore, because all DOC sources show dose-dependent loading behavior (albeit with quantitatively different loading curves), reconstructed DOC concentration trends through time should be viewed as robust to any interactions between organic molecules. Similarly, $\Delta^{13}\mathrm{C} \to 0$ at DOC/Fe(III) ratios predicted for most of our record other than the Neoproterozoic. Thus, δ^{13}C trends are likely robust and directly capture DOC signals with minimal fractionation, regardless of DOC source molecular interactions.

The corollary states that all co-precipitated OC is contained within the iron oxide mineral phase. This is supported by our XRD and elemental mapping results, which clearly identify most samples as containing iron (hydr)oxide cores, with a subset containing cores composed of andesite or quartz (Fig. S25–S123). The only samples deviating from this trend are those of the Presles Fm., which contain cores that can contain significant kaolinite and/or chamosite contributions (Fig. S87–S89). To test the effect of possible OC co-precipitation within non-iron-oxide phases (e.g., silicates), we performed a Fe-OC content correction based on XRF-derived Fe contents of extracted ooids. Assuming ideal mineral formulas [i.e., FeO(OH) = $63\,\mathrm{wt}\,\%$ Fe; Fe_2O_3 = $70\,\mathrm{wt}\,\%$ Fe], we estimate that all ooids are primarily composed of iron oxides, averaging $\sim 85\,\mathrm{wt}\,\%$ for both mineralogies (Fig. S128). Such high and consistent oxide content supports the hypothesis that all co-precipitated OC is associated with the iron oxide phase. Nevertheless, we tested this assumption by solving our model using corrected Fe-OC loadings [i.e., $W^m_{\mathrm{corr}}(t) = W^m(t)/f_{\mathrm{ox}}$, where $W^m_{\mathrm{corr}}(t)$ is the corrected Fe-OC loading and f_{ox} is the XRF-based $\mathrm{wt}\,\%$ iron oxide fraction], assuming no OC is associated with accessory minerals. Because iron oxide contents are high and consistent, results are indistinguishable from those determined using uncorrected Fe-OC loadings (Fig. S129). We therefore conclude that any OC associated with accessory phases does not influence DOC concentrations and δ^{13}C values predicted here.

Assumption (iv). No change in Fe(II) flux at the site of ooid formation through time. Because Fe(II) is oxidized to Fe(III) when exposed to oxygenated water, Fe(II) flux will control local Fe(III) concentrations at the site of ooid formation. This assumption is therefore required to translate Fe-OC loadings into absolute DOC concentrations. Importantly, its validity has no impact on Δ^{13}C and thus reconstructed DOC δ^{13}C trends, which only depend on DOC/Fe(III) ratio and not on absolute concentrations (Figs. S10-S11). While this assumption can impact our absolute DOC concentration estimates, our DOC δ^{13}C record is therefore robust to changes in Fe(II) flux.

Still, several lines of evidence suggest hydrothermal Fe(II) flux has remained relatively constant since $\approx$2000 Ma, even as open-ocean dissolved iron concentrations have decreased drastically over this time[24, 276]. First, although fluxes changed considerably in the Archean, the global lithospheric heat loss model of Ref.[51] predicts only a $\approx$40 % secular decrease in iron flux to Earth's surface since 2000 Ma. Similarly, Refs.[287,288] predict little-to-no change in surface heat flow from the mantle—which largely controls hydrothermal element fluxes—over this period. Combined with our observed lack of spatial heterogeneity in Fe-OC signals from similarly aged formations (Fig. S125), these result imply near constant hydrothermal Fe(II) flux at any given ooid formation site through space and time.

Second, we consider a hypothetical scenario where DOC concentrations have remained constant through time[7] and our Fe-OC loading record is instead controlled only by changes in Fe(II) flux, for example due to upwelling of ferruginous deep seawater. If true, this would require a $\sim$100-fold decrease in Fe(II) flux across the Proterozoic followed by a similar sized increase at the Precambrian-Cambrian boundary (Fig. 2), opposite of predicted upwelling trends[46]. Under the "large Proterozoic DOC reservoir" model of Ref.[6], the required Fe(II) flux decrease through the Proterozoic would be even larger (i.e., $\sim$100,000-fold) to explain our observations. Furthermore, while $\sim$10-fold variability in hydrothermal fluid and element flux throughout Earth's history is permissible by some models, the changes that would be required here are unreasonably large. Thus, while we cannot exclude the possibility that Fe(II) flux has changed modestly through space and time, we conclude that such changes were likely insufficient to explain the orders-of-magnitude variability in reconstructed DOC/Fe(III) ratios observed in our record.

Assumption (v). No difference in δ^{13}C for different sources. This assumption is required to translate end members weighted by their relative abundance in marine DOC to end members weighted by their relative abundance in Fe-OC; these weightings need not be identical due to the differential Fe-OC loadings for different end members at a given DOC/Fe(III) ratio (Figs. S18-S19). This assumption is relaxed only in the special case of equal Fe-OC loadings for all end members. That is, if $f^i(t) = w^{i,m}(t)/W^m(t)$ for all i, then Eq. S14 reduces to

$$\Delta^m(t) = \sum_i f^i(t)\Delta^{i,m}(t), \tag{S20}$$

which is simply the average of end-member fractionation factors weighted by their relative abundance in marine DOC. In this case, each end member need not be described by the same δ^{13}C value, since the weightings in Fe-OC are identical to those in marine DOC.

To assess the magnitude of potential biases due to this assumption, we calculated "true" vs. "reconstructed" marine DOC δ^{13}C values as a function of the δ^{13}C offset between M-DOC and C-DOC for two scenarios: (i) $[\text{DOC}]^*(t) = 0.1$ as reconstructed by hematite, representing Proterozoic conditions; and (ii) $[\text{DOC}]^*(t) = 1$ as reconstructed by goethite, representing Phanerozoic conditions (Fig. S130). In the Proterozoic case, an unrealistically large end-member offset in marine DOC of $\delta^{13}\text{C}_{\text{M-DOC}} - \delta^{13}\text{C}_{\text{C-DOC}} = 20$ ‰ would translate to a true-minus-predicted bias

of $\approx 6\,‰$ at $f^{\text{M-DOC}}(t) \approx 0.1$ (Fig. S130A). This bias approaches zero as $f^{\text{M-DOC}} \rightarrow 0.65$, the point at which the special case of Eq. S20 is met, and as $f^{\text{M-DOC}} \rightarrow 0$ or $f^{\text{M-DOC}} \rightarrow 1$, i.e., when all DOC is derived from a single end member. In the Phanerozoic case, the same unrealistically large end-member offset in marine DOC of $\delta^{13}C_{\text{M-DOC}} - \delta^{13}C_{\text{C-DOC}} = 20\,‰$ would translate a maximum true-minus-predicted bias of $\approx 4\,‰$ at $f^{\text{M-DOC}}(t) \approx 0.35$ (Fig. S130B). This bias again approaches zero in the single end-member limits of $f^{\text{M-DOC}} \rightarrow 0$ and $f^{\text{M-DOC}} \rightarrow 1$.

However, it is unlikely that different quantitatively important end members exhibited $\delta^{13}C$ offsets as large as $20\,‰$ at any point in our iron ooid record. This is because oxygenic photosynthesis via the Cavin-Benson-Bassham (CBB) cycle has likely been a dominant metabolism at least since the Mesoproterozoic, before the beginning of our record[289]. All organisms that fix carbon via the CBB cycle utilize the enzyme ribulose 1,5-bisphosphate carboxylase oxygenase (RuBisCO)[277], which exhibits an *in vitro* ^{13}C fractionation factor between CO_2 and organic matter that differs by a maximum of $\approx 10\,‰$ across all marine oxygenic photosynthetic organisms studied to date (i.e., RuBisCO types IA, IB, ID, and II)[290]. In contrast, chemolithoautotrophic metabolisms—which can express drastically different fractionation factors—were likely quantitatively unimportant in terms of DOC generation throughout our record. Differences in expressed fractionation between oxygenic photosynthetic organisms are further dampened when analyzed *in vivo*, likely due to the importance of carbon-concentrating mechanisms (CCMs) in modern photosynthetic organisms[290]. However, CCMs may not have yet evolved at the start of our iron ooid record[5]. Nevertheless, more realistic end-member $\delta^{13}C$ offsets of ≈ 5 (after the advent of CCMs) to $10\,‰$ (before the advent of CCMs) would lead to smaller biases in reconstructed average DOC $\delta^{13}C$ values that never exceed 2 to $3\,‰$. This bias is within the typical model uncertainty for any given geologic period.

7 Interpreting organic $\delta^{13}C$ trends in multiple geologic materials

Finally, we compare our iron ooid Fe-OC record to compiled data from other geologic organic carbon geologic: kerogen, crude oil, and carbonate-associated OC from carbonate rocks (Fig. S131). What emerges is a clear secular pattern of progressively heaver carbon-isotope compositions with time since at least $2000\,\text{Ma}$; this trend is particularly evident in kerogen, crude oil, and Fe-OC records (Fig. S131A). For example, kerogen $\delta^{13}C$ shifts from values as low as $-40\,‰$ at $2000\,\text{Ma}$ to values as high as $-15\,‰$ in the Pliocene, near the modern day. Interestingly, while carbonate-associated OC exhibits significantly more scatter than any other record (Fig. S131B), the most negative $\delta^{13}C$ values observed within a given time period exhibit a similar secular trend toward progressively heavier carbon-isotope compositions. When interpreting carbonate-associated OC in this way, all records agree that OC $\delta^{13}C$ values increased by ≈ 20 to $25\,‰$ since $2000\,\text{Ma}$.

8 Supplementary Data Tables

Table S1: Fluorescence and absorbance results for DOC starting materials. Units for each metric are reported in parentheses. Abbreviations refer to: M-DOC = modern marine analog DOC; C-DOC = cyanobacterial DOC; FA = fulvic acid; FDOM = fluorescent dissolved organic matter; RU = Raman Units; FI = Fluorescence Index.

**Parameter**	**M-DOC**	**C-DOC**	**FA**
Fluorescence metrics			
FDOM (RU)	10.48	180.70	56.06
A (%)	29.7	28.6	27.5
B (%)	11.9	7.6	11.5
C (%)	15.7	27.5	21.4
M (%)	24.6	17.8	23.8
T (%)	18.1	18.5	15.8
FI (unitless)	2.23	3.06	1.95
Absorbance metrics			
$SUVA_{254}$ ($\mathrm{mg\,L^{-1}\,m^{-1}}$)	1.30	4.05	1.65
$S_{275-295}$ ($\mathrm{nm^{-1}}$)	0.017	0.018	0.011
$S_{350-400}$ ($\mathrm{nm^{-1}}$)	0.013	0.008	0.016
S_R (unitless)	1.29	2.20	0.67

Table S2: Fe-OC loading and Δ^{13}C calibration curve model fit statistics. Ordinary least squares (OLS) root mean square error (RMSE) and regression r^2 values for all power-law and dual exponential calibration curve fits. Results are reported as mean and standard deviation ($\pm 1\sigma$) for 10,000 Monte Carlo iterations.

	**RMSE**	**r^2**
Goethite		
M-DOC		
Fe-OC loading	0.028 ± 0.012	0.987 ± 0.022
Δ^{13}C	1.281 ± 0.010	0.829 ± 0.002
C-DOC		
Fe-OC loading	0.041 ± 0.011	0.984 ± 0.017
Δ^{13}C	0.202 ± 0.019	0.905 ± 0.022
FA		
Fe-OC loading	0.092 ± 0.010	0.961 ± 0.013
Δ^{13}C	0.956 ± 0.027	0.946 ± 0.003
Hematite		
M-DOC		
Fe-OC loading	0.041 ± 0.001	0.880 ± 0.088
Δ^{13}C	1.244 ± 0.049	0.845 ± 0.012
C-DOC		
Fe-OC loading	0.010 ± 0.004	0.986 ± 0.021
Δ^{13}C	0.914 ± 0.054	0.977 ± 0.003
FA		
Fe-OC loading	0.010 ± 0.006	0.990 ± 0.026
Δ^{13}C	0.423 ± 0.039	0.975 ± 0.004

Table S3: Radiocarbon ages of modern goethite ooids. Ages are reported in units of fraction modern (Fm) as well as ^{14}C yr (Ref.[82]), with uncertainty reported as $\pm 1\sigma$.

**Sample**	**Fm**	**^{14}C age**
Panarea Island, Italy	0.5232 ± 0.005	5203 ± 76
Mahengentang Island, Indonesia	0.6472 ± 0.006	3552 ± 73

Table S4: Primers used for first-step PCR amplification of the bacterial 16S rRNA gene.

**Primer**	**Sequence**
Illumina16S-515F	TCGTCGGCAGCGTCAGATGTGTATAAGAGACAG<br>NNNNN GTGYCAGCMGCCGCGGTAA
Illumina16S-926R	GTCTCGTGGGCTCGGAGATGTGTATAAGAGACAG<br>NNNNN CCGYCAATTYMTTTRAGTTT

Table S5: Thermal profile used for first-step PCR amplification of the bacterial 16S rRNA gene.

**Step**	**Temperature**	**Time**
Initial Denaturation	95 °C	120 s
32 cycles consisting of:		
Denaturation	95 °C	45 s
Annealing	50 °C	45 s
Extension	68 °C	90 s
Final Elongation	68 °C	300 s
Refrigeration	4 °C	∞

9 Supplementary Data Figures

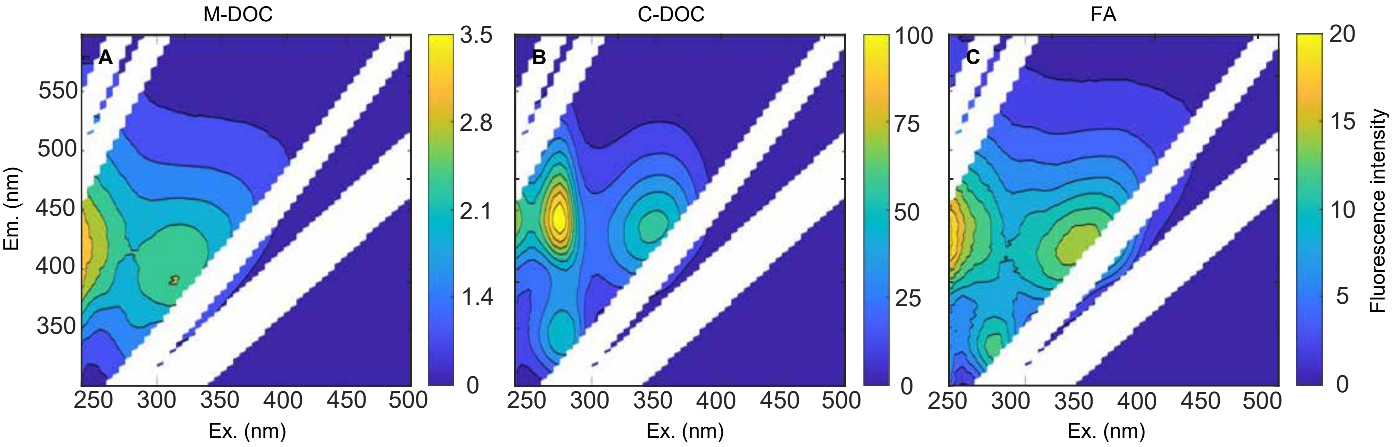

Fig. S1: Excitation-emission fluorescence matrices (EEMs) for DOC types used in this study. (**A**) modern marine analog DOC ("M-DOC"), (**B**) cyanobacterial DOC ("C-DOC"), and (**C**) fulvic acid ("FA"). All panels show fluorescence intensity as a function of excitation and emission wavelength.

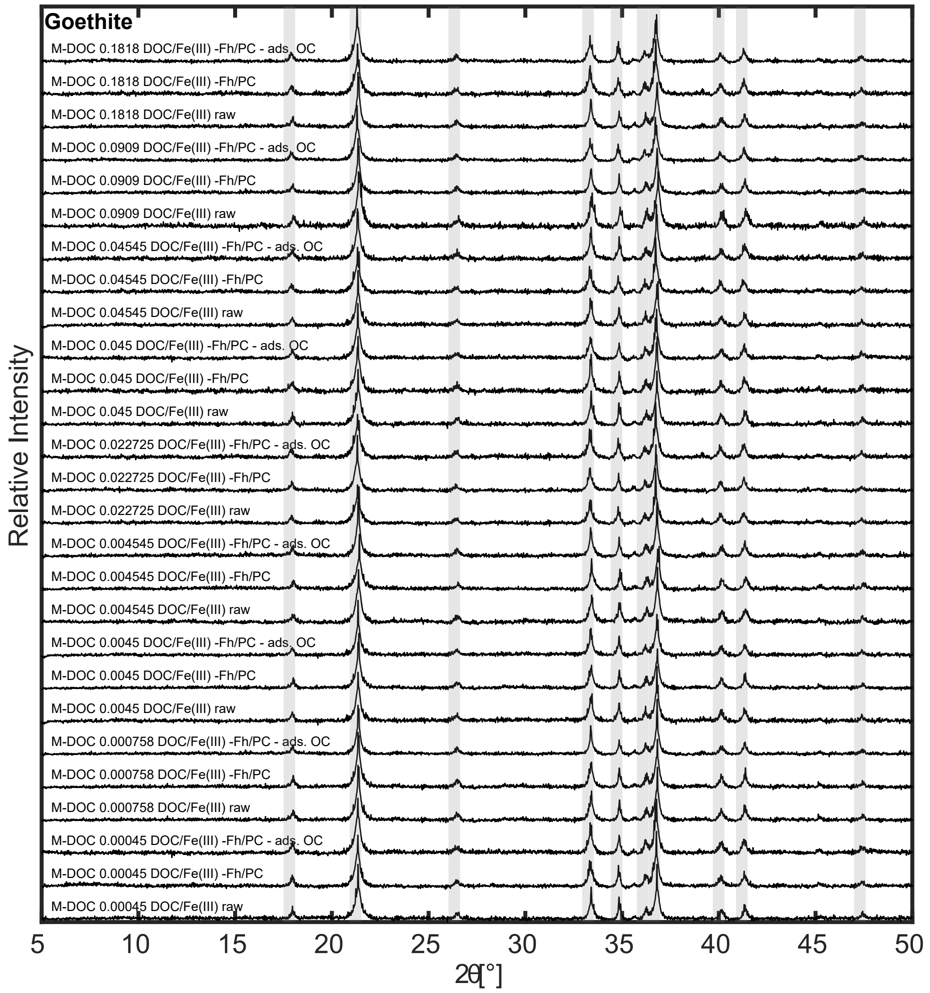

Fig. S2: X-ray diffractograms of goethite synthesized under circumneutral pH in the presence of M-DOC. Gray lines denote diagnostic goethite peaks (ICDD PDF 01-076-9683); no other mineral signatures were detected. Annotations specify: (i) DOC type (i.e., "M-DOC", "C-DOC", or "FA"), (ii) carbon to iron molar ratio in the initial solution ("DOC/Fe(III)"), and (iii) processing stage (i.e., "raw", "−Fh/PC", or "−Fh/PC −ads. OC"; see Methods for processing details).

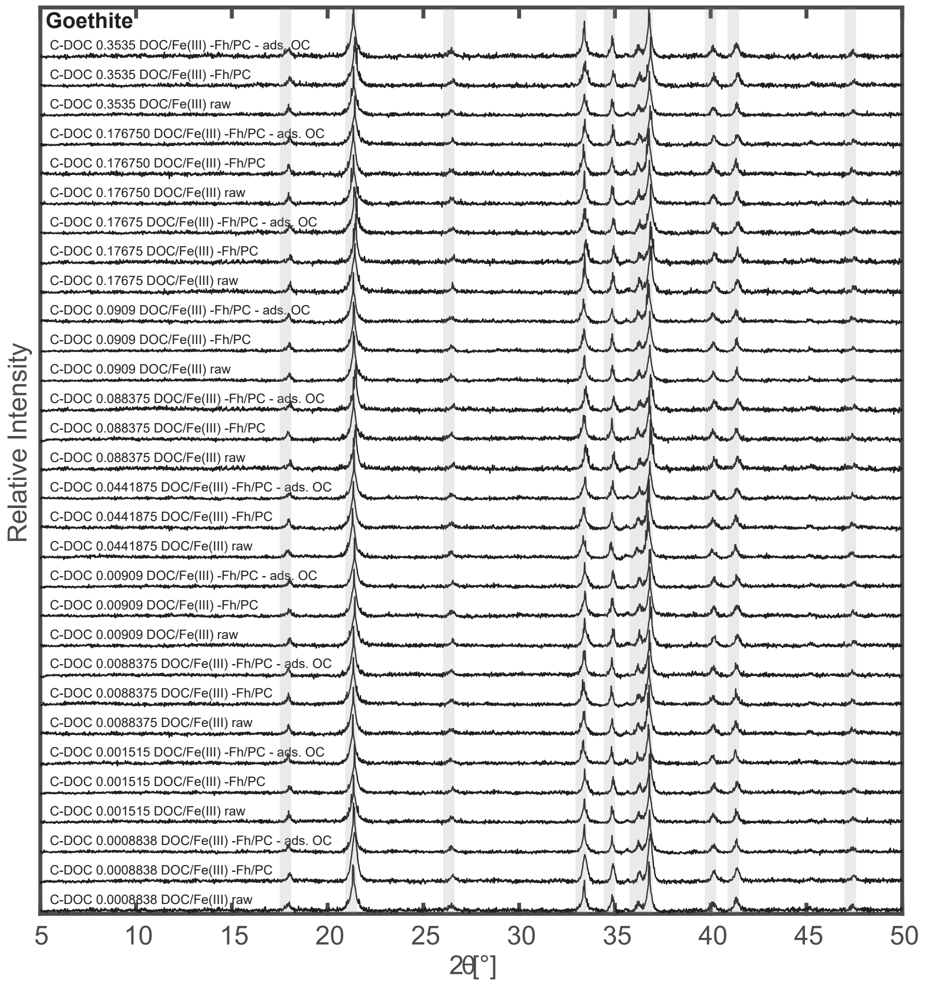

Fig. S3: X-ray diffractograms of goethite synthesized under circumneutral pH in the presence of C-DOC. Gray lines and annotations are the same as described in Fig. S2.

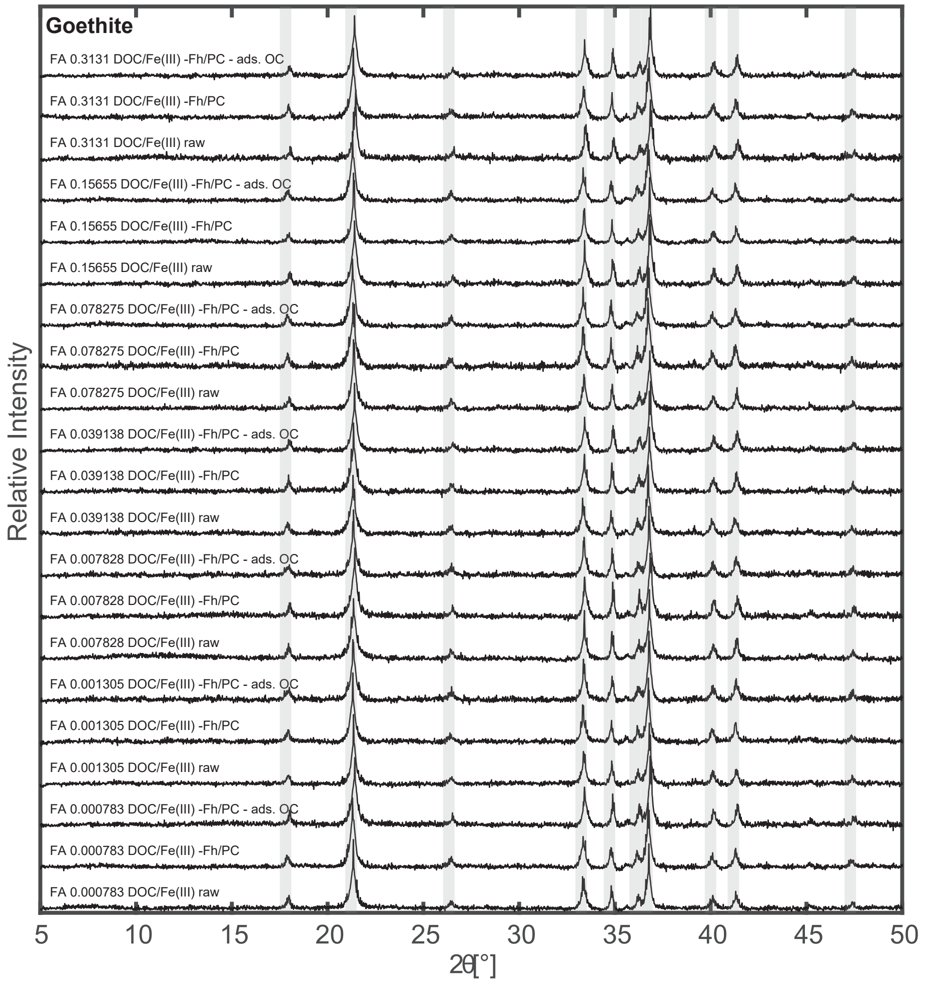

Fig. S4: X-ray diffractograms of goethite synthesized under circumneutral pH in the presence of FA. Gray lines and annotations are the same as described in Fig. S2.

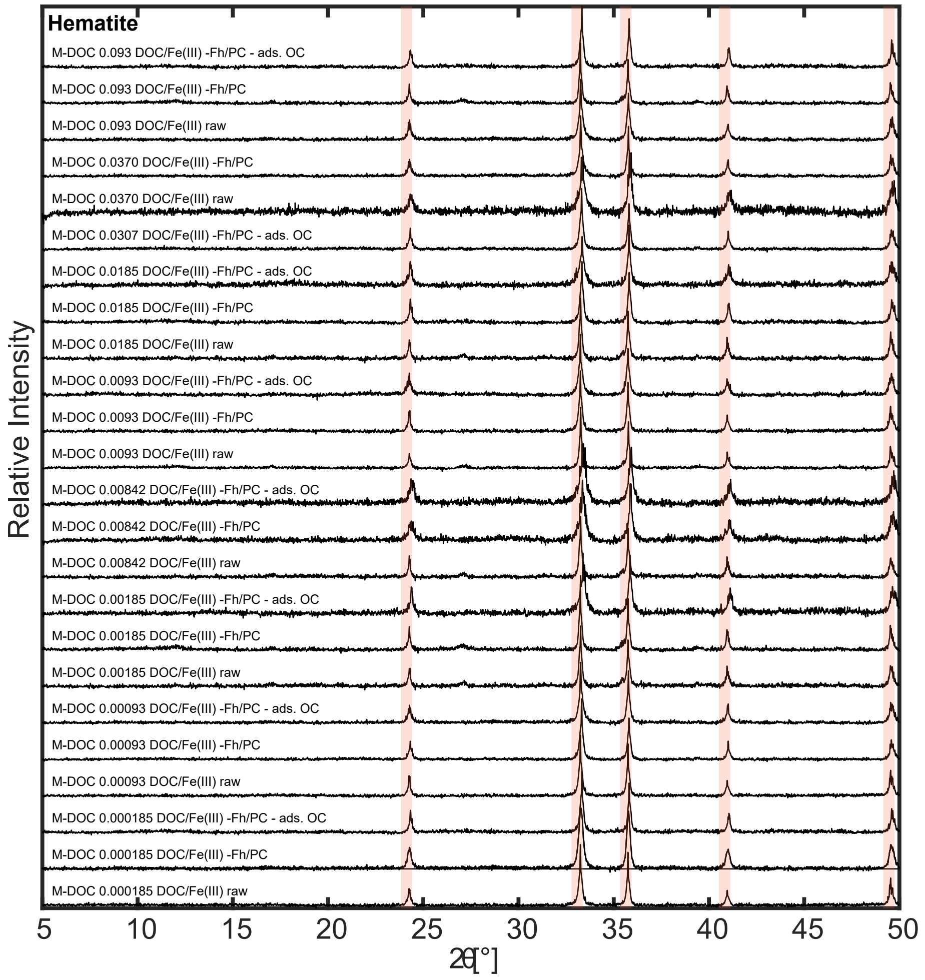

Fig. S5: X-ray diffractograms of hematite synthesized under circumneutral pH in the presence of M-DOC. Red lines denote diagnostic hematite peaks (ICDD PDF 01-073-9835); no other mineral signatures were detected. Annotations specify: (i) DOC type (i.e., "M-DOC", "C-DOC", or "FA"), (ii) carbon to iron molar ratio in the initial solution ("DOC/Fe(III)"), and (iii) processing stage (i.e., "raw", "−Fh/PC", or "−Fh/PC −ads. OC"; see Methods for processing details).

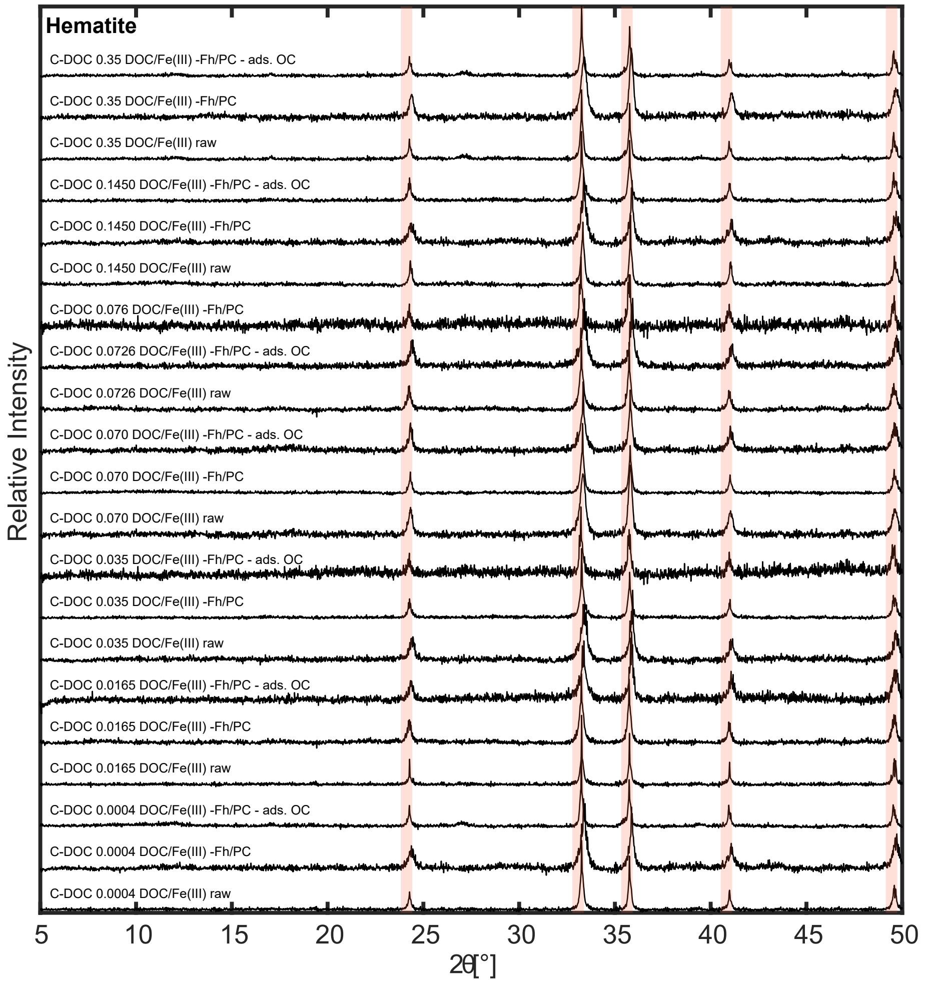

Fig. S6: X-ray diffractograms of hematite synthesized under circumneutral pH in the presence of C-DOC. Red lines and annotations are the same as described in Fig. S5.

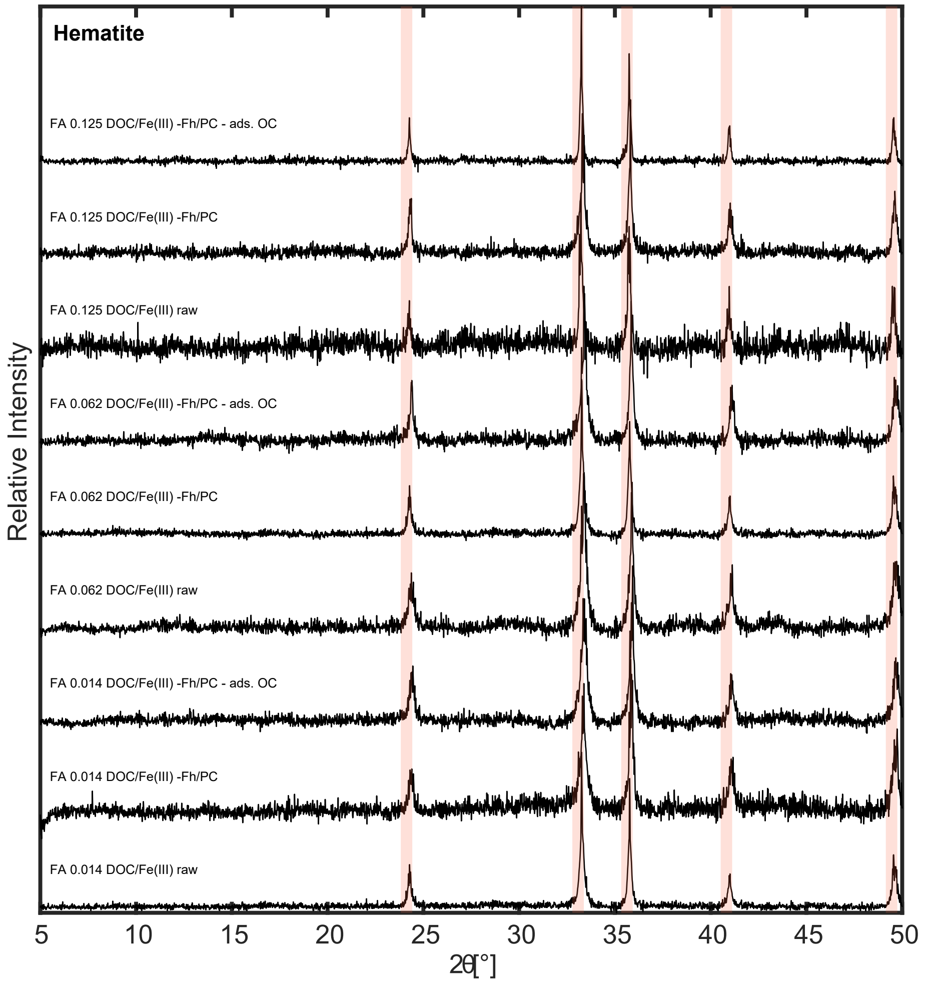

Fig. S7: X-ray diffractograms of hematite synthesized under circumneutral pH in the presence of FA. Red lines and annotations are the same as described in Fig. S5.

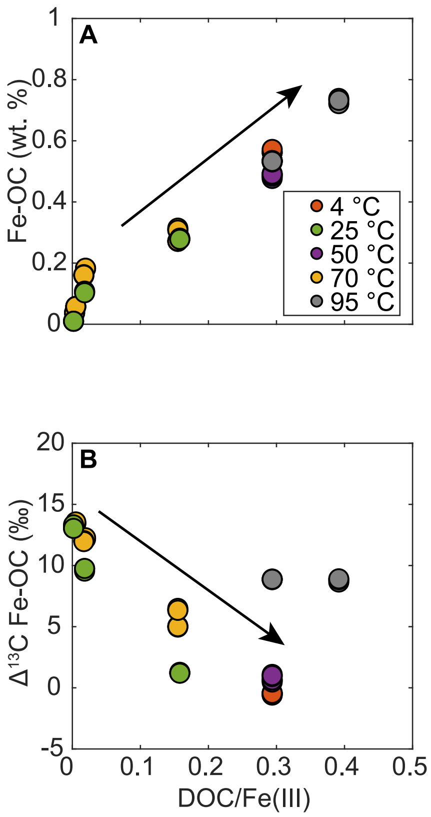

Fig. S8: Impact of temperature on goethite Fe-OC signals. (**A**) Fe-OC loadings (in wt %) and (**B**) isotope fractionations ($\Delta^{13}C = \delta^{13}C_{Fe-OC} - \delta^{13}C_{DOC}$, in ‰) as a function of temperature and DOC/Fe(III) ratio. Marker colors indicate experiment temperature, which ranged from 4 °C to 95 °C. Fe-OC loadings are largely independent of temperature, as are $\Delta^{13}C$ values for experiments performed between 4 °C and 70 °C. In contrast, $\Delta^{13}C$ exhibits a marked deviation at 95 °C, indicating a lighter isotopic composition than is observed for lower, more environmentally relevant temperatures. Experimental conditions were: pH = 11.4 to 11.5, FA as DOC source, $FeCl_3$ as Fe(III) source, DOC addition after ferrihydrite precipitation, aged 7 to 14 days, measurement of "−Fh/PC −ads. OC" fractions.

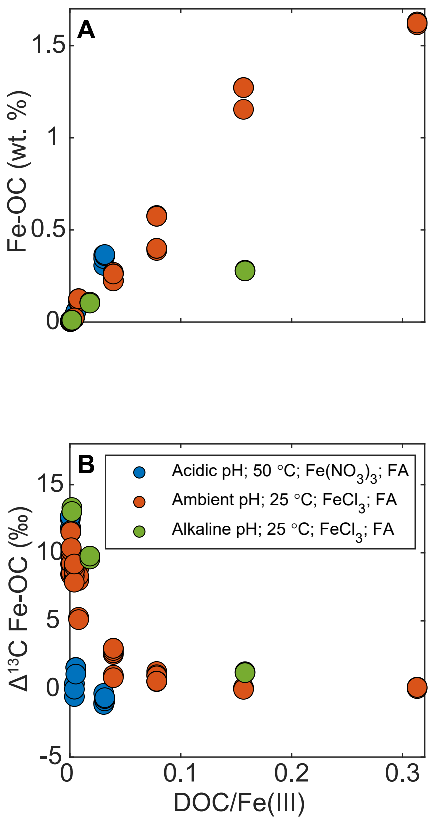

Fig. S9: Impact of pH on goethite Fe-OC signals. (**A**) Fe-OC loadings (in wt %) and (**B**) isotope fractionations ($\Delta^{13}C = \delta^{13}C_{Fe-OC} - \delta^{13}C_{DOC}$, in ‰) as a function of pH and DOC/Fe(III) ratio. Marker colors indicate experiment pH, which ranged from 1.8 to 11.5. Fe-OC loadings are largely independent of pH, as are $\Delta^{13}C$ values for experiments performed in the acidic to circumneutral pH range. In contrast, $\Delta^{13}C$ exhibits a marked deviation at pH = 11.5, indicating a lighter isotopic composition than is observed for lower, more environmentally relevant pH values. Experimental conditions were: 25 °C (circumneutral and alkaline) or 50 °C (acidic), FA as DOC source, $FeCl_3$ (circumneutral and alkaline) or $Fe(NO_3)_3$ (acidic) as Fe(III) source, DOC addition after ferrihydrite precipitation, aged 5 to 10 days, measurement of "−Fh/PC −ads. OC" fractions.

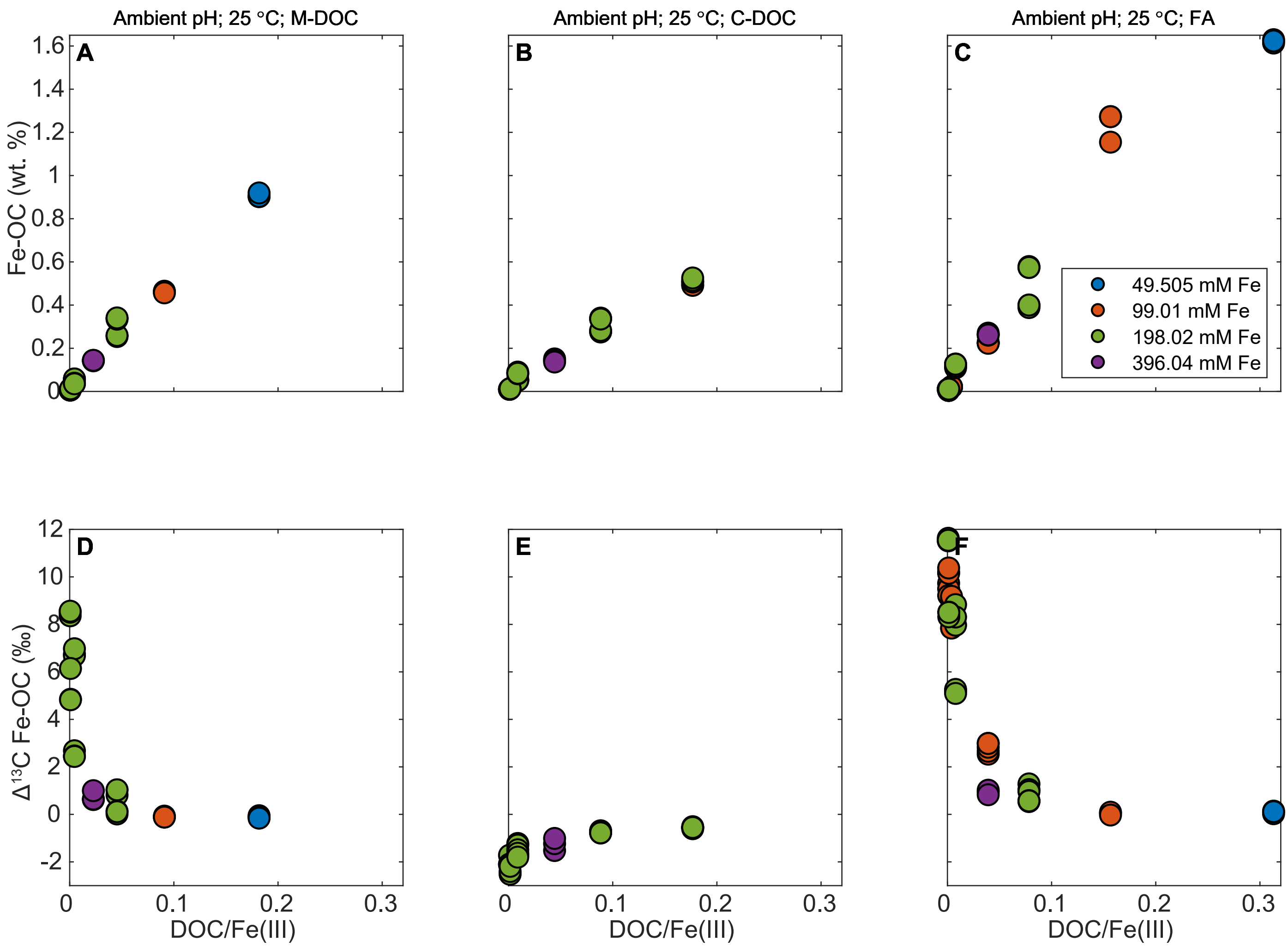

Fig. S10: Impact of adjusting DOC vs. adjusting Fe(III) concentrations on goethite Fe-OC signals. (**A**-**C**) Fe-OC loadings (in wt %) and (**D**-**F**) isotope fractionations ($\Delta^{13}C = \delta^{13}C_{Fe-OC} - \delta^{13}C_{DOC}$, in ‰) as a function of DOC/Fe(III) ratio, either manipulated by adjusting DOC concentrations or Fe(III) concentrations. Marker colors indicate experiment Fe(III) concentrations, which ranged from 50 mM to 400 mM. Fe-OC loadings and $\Delta^{13}C$ values are largely independent of whether DOC or Fe(III) concentrations are adjusted. Experimental conditions were: 25 °C; pH = 5-7; (**A**, **D**) M-DOC, (**B**, **E**) C-DOC, or (**C**, **F**) FA as DOC source; $FeCl_3$ as Fe(III) source, DOC addition after ferrihydrite precipitation, aged 5 to 120 days, measurement of "−Fh/PC −ads. OC" fractions.

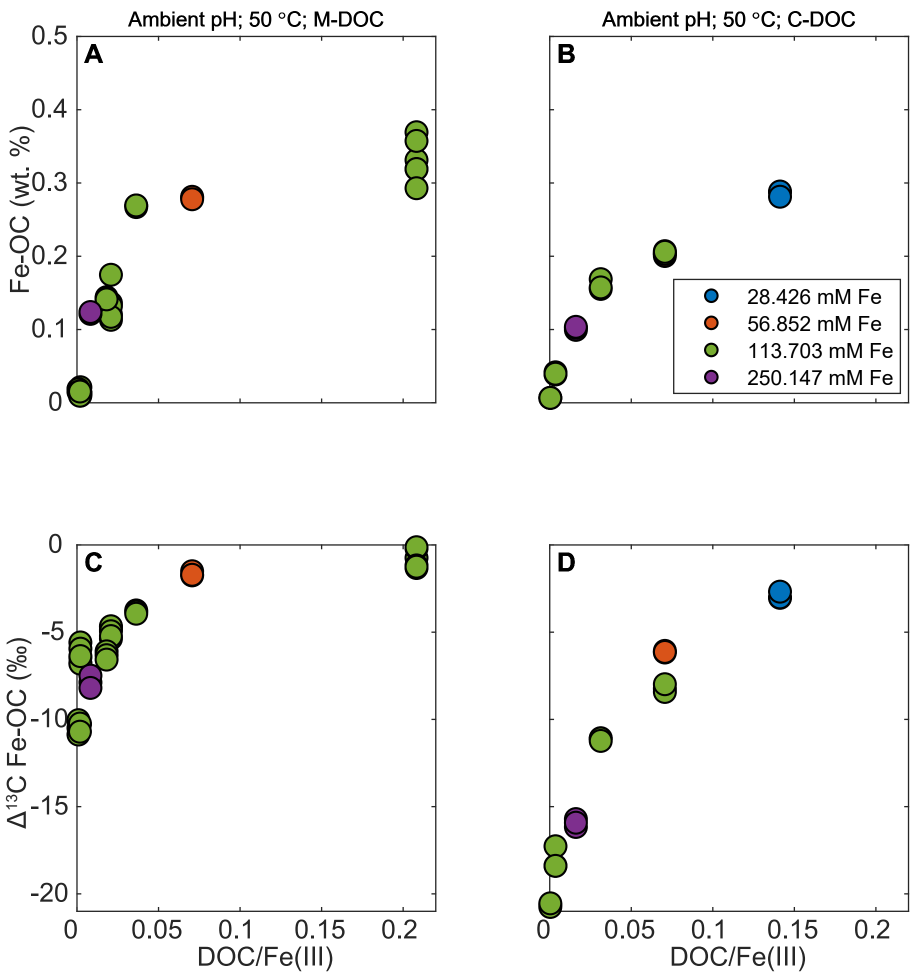

Fig. S11: Impact of adjusting DOC vs. adjusting Fe(III) concentrations on hematite Fe-OC signals. (**A**-**B**) Fe-OC loadings (in wt %) and (**C**-**D**) isotope fractionations ($\Delta^{13}C = \delta^{13}C_{Fe-OC} - \delta^{13}C_{DOC}$, in ‰) as a function of DOC/Fe(III) ratio, either manipulated by adjusting DOC concentrations or Fe(III) concentrations. Marker colors indicate experiment Fe(III) concentrations, which ranged from 30 mM to 250 mM. Fe-OC loadings and $\Delta^{13}C$ values are largely independent of whether DOC or Fe(III) concentrations are adjusted. Experimental conditions were: 50 °C; pH = 8; (**A**, **C**) M-DOC, (**B**, **D**) C-DOC as DOC source; $FeCl_3$ as Fe(III) source, DOC addition after ferrihydrite precipitation, aged 5 to 120 days, measurement of "−Fh/PC −ads. OC" fractions.

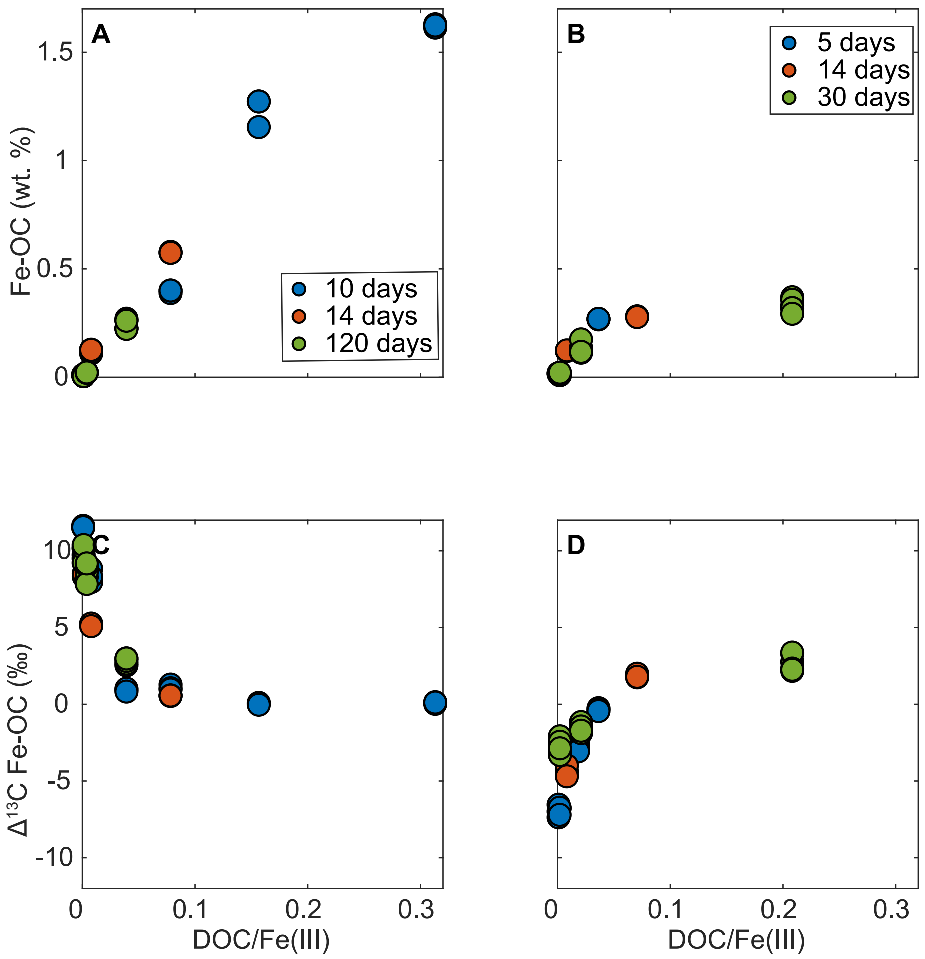

Fig. S12: Impact of synthesis duration on goethite and hematite Fe-OC signals. (**A**-**B**) Fe-OC loadings (in wt %) and (**C**-**D**) isotope fractionations ($\Delta^{13}C = \delta^{13}C_{Fe-OC} - \delta^{13}C_{DOC}$, in ‰) as a function of synthesis duration and DOC/Fe(III) ratio for (**A**, **C**) goethite and (**B**, **D**) hematite. Marker colors indicate experiment duration, which ranged from 5 to 120 days. Fe-OC loadings and $\Delta^{13}C$ values are largely independent of synthesis duration. Goethite experimental conditions were: 25 °C; pH = 5-7; FA as DOC source; $FeCl_3$ as Fe(III) source, DOC addition after ferrihydrite precipitation, measurement of "−Fh/PC −ads. OC" fractions. Hematite experimental conditions were: 50 °C; pH = 8; M-DOC as DOC source; $FeCl_3$ as Fe(III) source, DOC addition after ferrihydrite precipitation, measurement of "−Fh/PC −ads. OC" fractions.

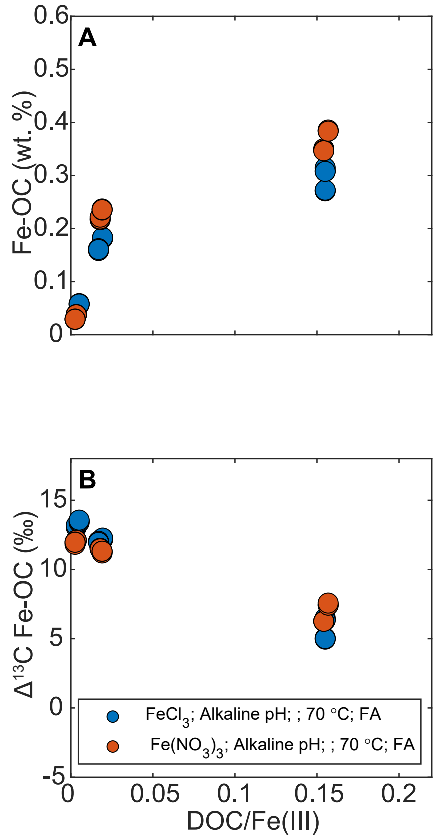

Fig. S13: Impact of Fe(III) source on goethite Fe-OC signals. (**A**) Fe-OC loadings (in wt %) and (**B**) isotope fractionations ($\Delta^{13}C = \delta^{13}C_{Fe-OC} - \delta^{13}C_{DOC}$, in ‰) as a function of DOC/Fe(III) ratio for two diferent Fe(III) sources (marker colors). Fe-OC loadings and $\Delta^{13}C$ values are largely independent of Fe(III) source. Experimental conditions were: 70 °C, pH = 11.5, FA as DOC source, DOC addition after ferrihydrite precipitation, aged 7 to 10 days, measurement of "−Fh/PC −ads. OC" fractions.

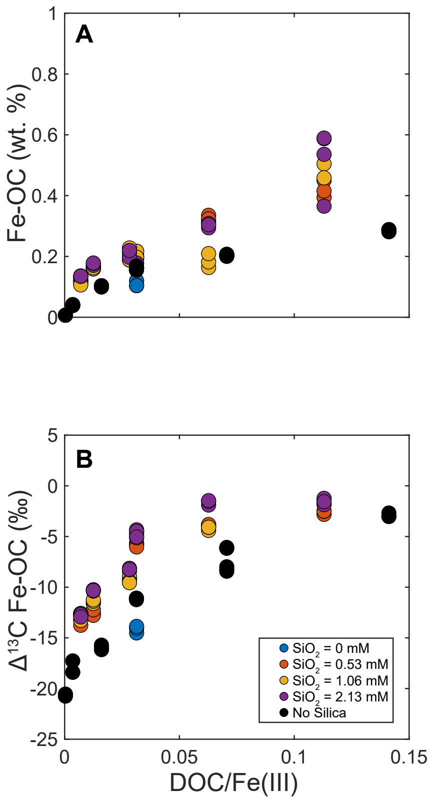

Fig. S14: Impact of parent solution silica concentration on hematite Fe-OC signals. (**A**) Fe-OC loadings (in wt %) and (**B**) isotope fractionations ($\Delta^{13}C = \delta^{13}C_{Fe-OC} - \delta^{13}C_{DOC}$, in ‰) as a function of DOC/Fe(III) ratio for four diferent SiO_2 concentrations (marker colors). Fe-OC loadings and $\Delta^{13}C$ values are largely independent of the presence and concentration of dissolved silica. Experimental conditions were: 50 °C, pH = 8, C-DOC as DOC source, DOC addition after ferrihydrite precipitation, aged 5 to 14 days, measurement of "−Fh/PC −ads. OC" fractions.

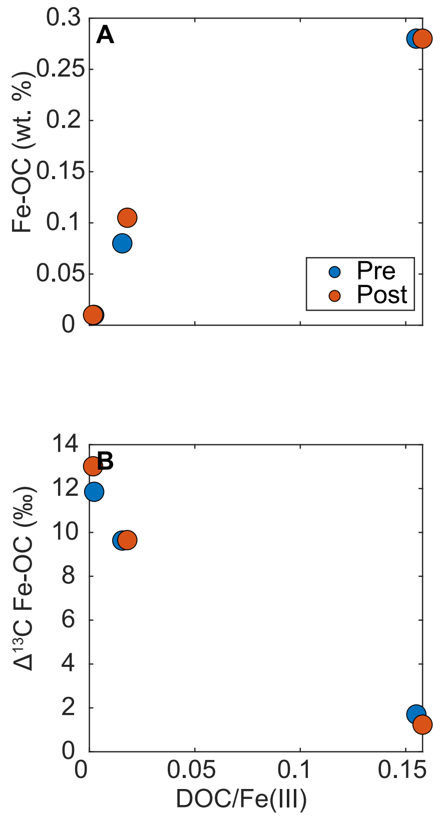

Fig. S15: Impact of DOC introduction timing on goethite Fe-OC signals. (**A**) Fe-OC loadings (in wt %) and (**B**) isotope fractionations ($\Delta^{13}C = \delta^{13}C_{Fe-OC} - \delta^{13}C_{DOC}$, in ‰) as a function DOC/Fe(III) ratio for pre- vs. post-ferrihydrite DOC introduction (marker colors). Fe-OC loadings are largely independent of DOC introduction timing. Experimental conditions were: 25 °C, pH = 11.5, FA as DOC source, $FeCl_3$ as Fe(III) source, aged 7 days, measurement of "−Fh/PC −ads. OC" fractions.

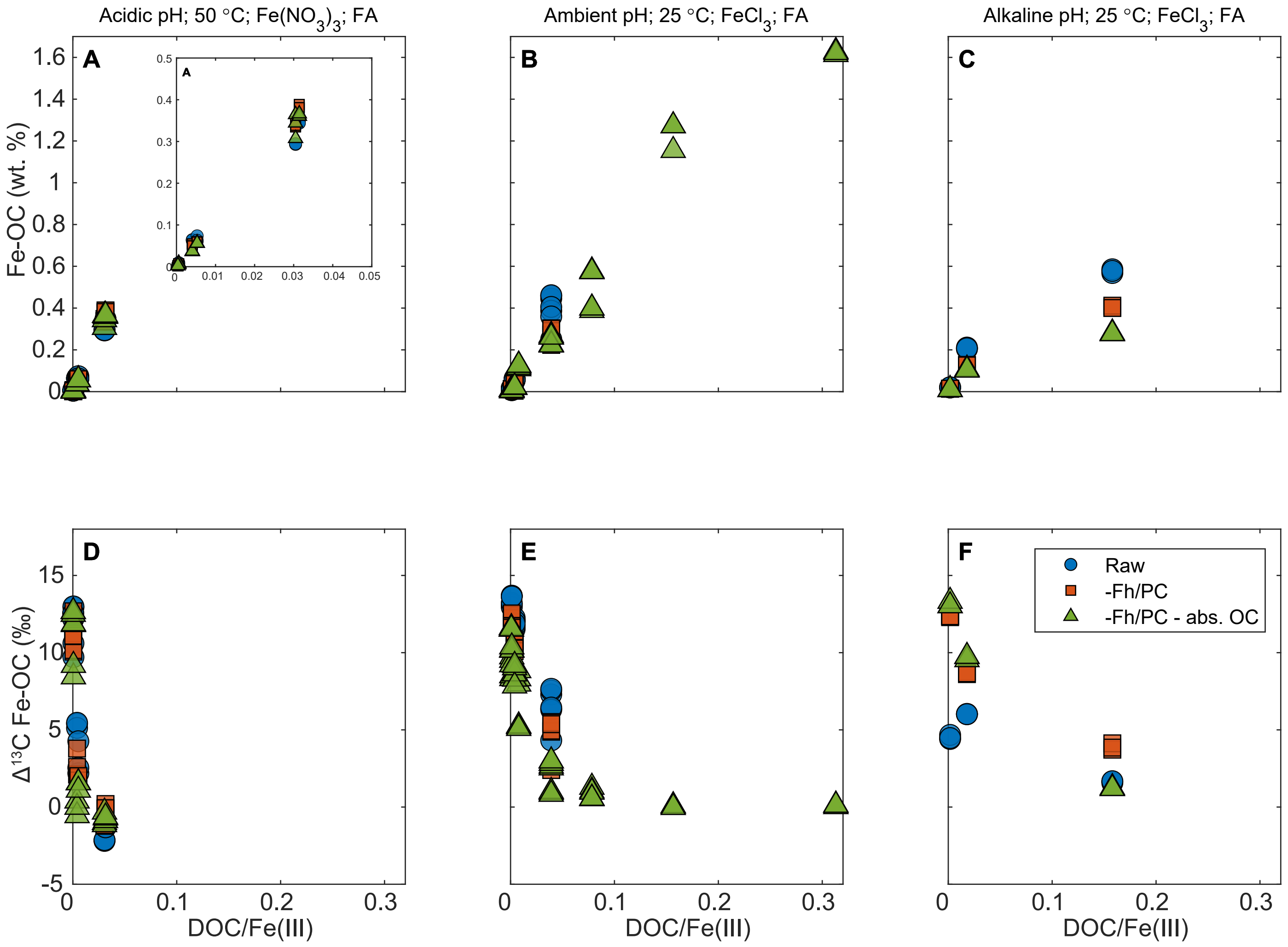

Fig. S16: Impact of rinsing and desorption procedure on goethite Fe-OC signals. (**A-C**) Fe-OC loadings (in wt %) and (**D-F**) isotope fractionations ($\Delta^{13}C = \delta^{13}C_{Fe-OC} - \delta^{13}C_{DOC}$, in ‰) as a function of DOC/Fe(III) ratio for "raw", "−Fh/PC", and "−Fh/PC −ads. OC" fractions (marker color and shape) at several experimental conditions. In all cases, rinsing and desorption steps can exhibit large impacts on Fe-OC signals, highlighting the need to remove ferrihydrite-bound and adsorbed OC prior to analysis. Experimental conditions were: (**A**, **D**) 50 °C or (**B**, **C**, **E**, **F**) 25 °C; (**A**, **D**) pH = 1.8, (**B**, **E**) pH = 5-7, or (**C**, **F**) pH = 11.5; FA as DOC source; (**A**, **D**) $Fe(NO_3)_3$ or (**B**, **C**, **E**, **F**) $FeCl_3$ as Fe(III) source; DOC addition after ferrihydrite precipitation, aged 5 to 120 days. Semi-transparent markers are individual analyses ($n = 3$ per sample) and opaque markers are sample means. Insets show zoomed-in view of the low DOC/Fe(III) range.

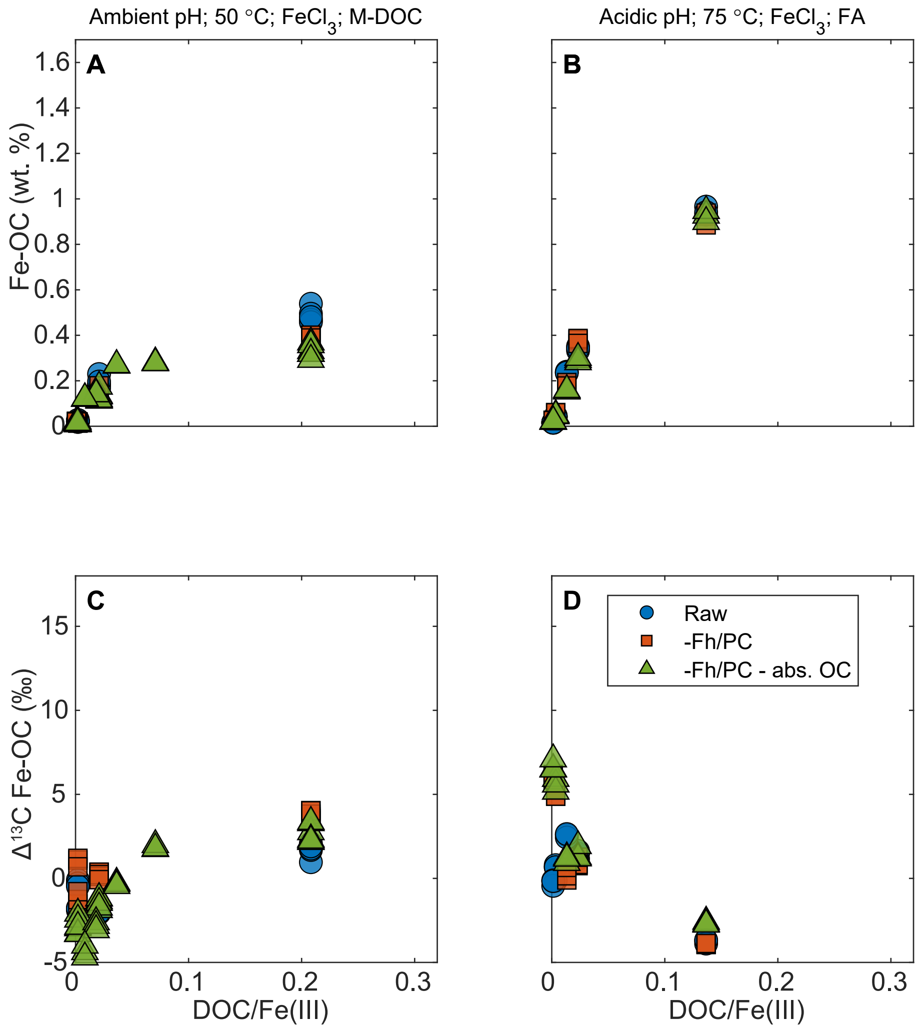

Fig. S17: Impact of rinsing and desorption procedure on hematite Fe-OC signals. (**A**-**B**) Fe-OC loadings (in wt %) and (**C**-**D**) isotope fractionations ($\Delta^{13}C = \delta^{13}C_{Fe-OC} - \delta^{13}C_{DOC}$, in ‰) as a function of DOC/Fe(III) ratio for "raw", "−Fh/PC", and "−Fh/PC −ads. OC" fractions (marker color and shape) at several experimental conditions. In all cases, rinsing and desorption steps can exhibit large impacts on Fe-OC signals, highlighting the need to remove ferrihydrite-bound and adsorbed OC prior to analysis. Experimental conditions were: (**A**, **C**) $50\,^{\circ}C$ or (**B**, **D**) $75\,^{\circ}C$; (**A**, **C**) pH = 8.0 or (**B**, **D**) pH = 1.8; (**A**, **C**) M-DOC or (**B**, **D**) FA as DOC source; $FeCl_3$ as Fe(III) source; DOC addition after ferrihydrite precipitation; aged 5 to 120 days. Semi-transparent markers are individual analyses ($n = 3$ per sample) and opaque markers are sample means.

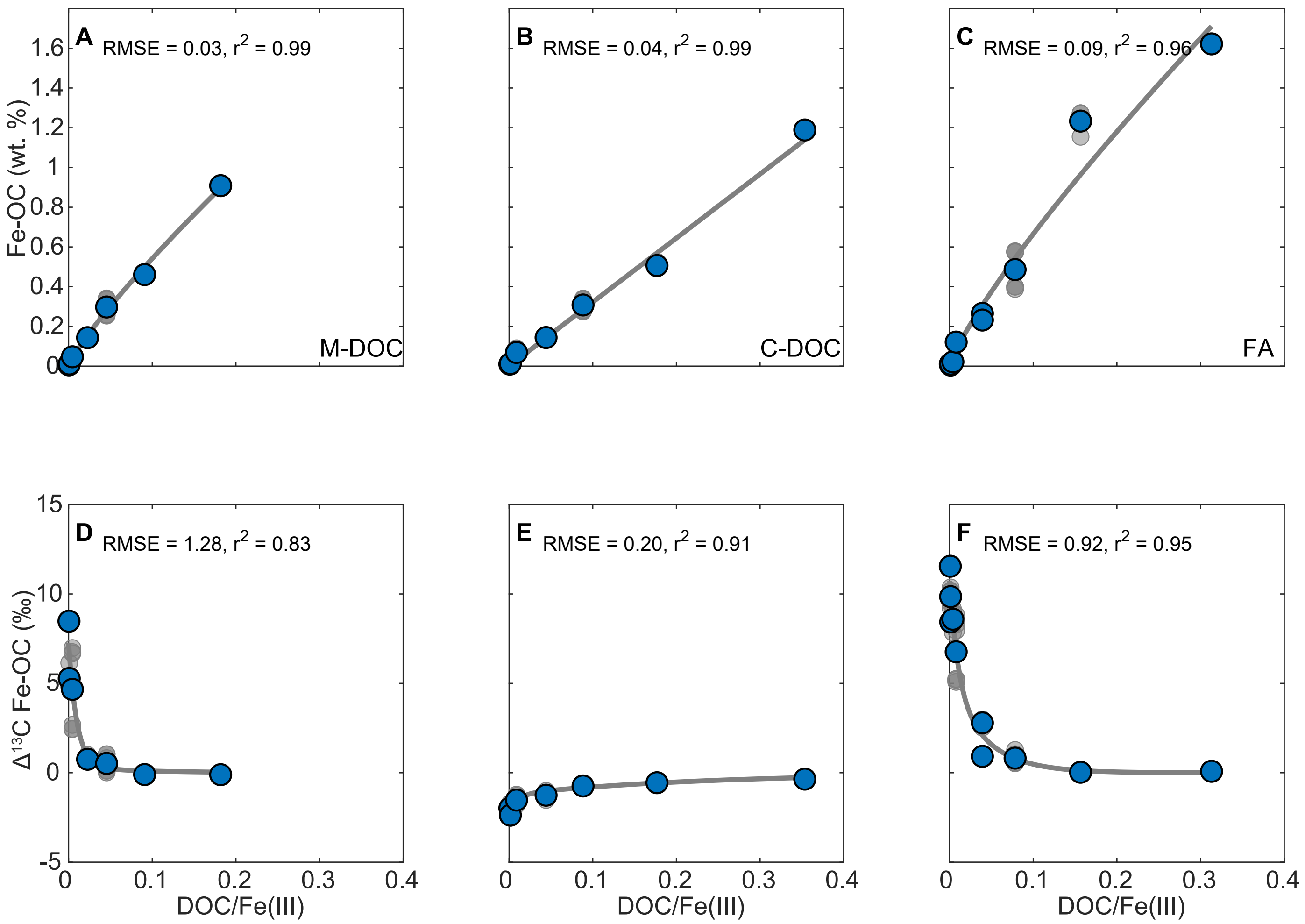

Fig. S18: Final goethite DOC/Fe(III) vs. Fe-OC response curves. (**A**-**C**) Fe-OC loadings (in wt %) and (**D**-**F**) isotope fractionations ($\Delta^{13}C = \delta^{13}C_{Fe-OC} - \delta^{13}C_{DOC}$, in ‰) as a function of DOC/Fe(III) ratio for (**A**, **D**) M-DOC, (**B**, **E**) C-DOC, and (**C**, **F**) FA as DOC sources. Experimental conditions were: 25 °C; pH = 5-7; $FeCl_3$ as Fe(III) source; DOC addition after ferrihydrite precipitation; aged (**A**, **B**, **D**, **E**) 10 to 14 days or (**C**, **F**) 10 to 120 days; measurement of "−Fh/PC −ads. OC" fractions. Semi-transparent markers are individual analyses ($n = 3$ per sample) and opaque markers are sample means. Thick gray lines are best-fit OLS regressions, with reported regression uncertainty (r^2, RMSE).

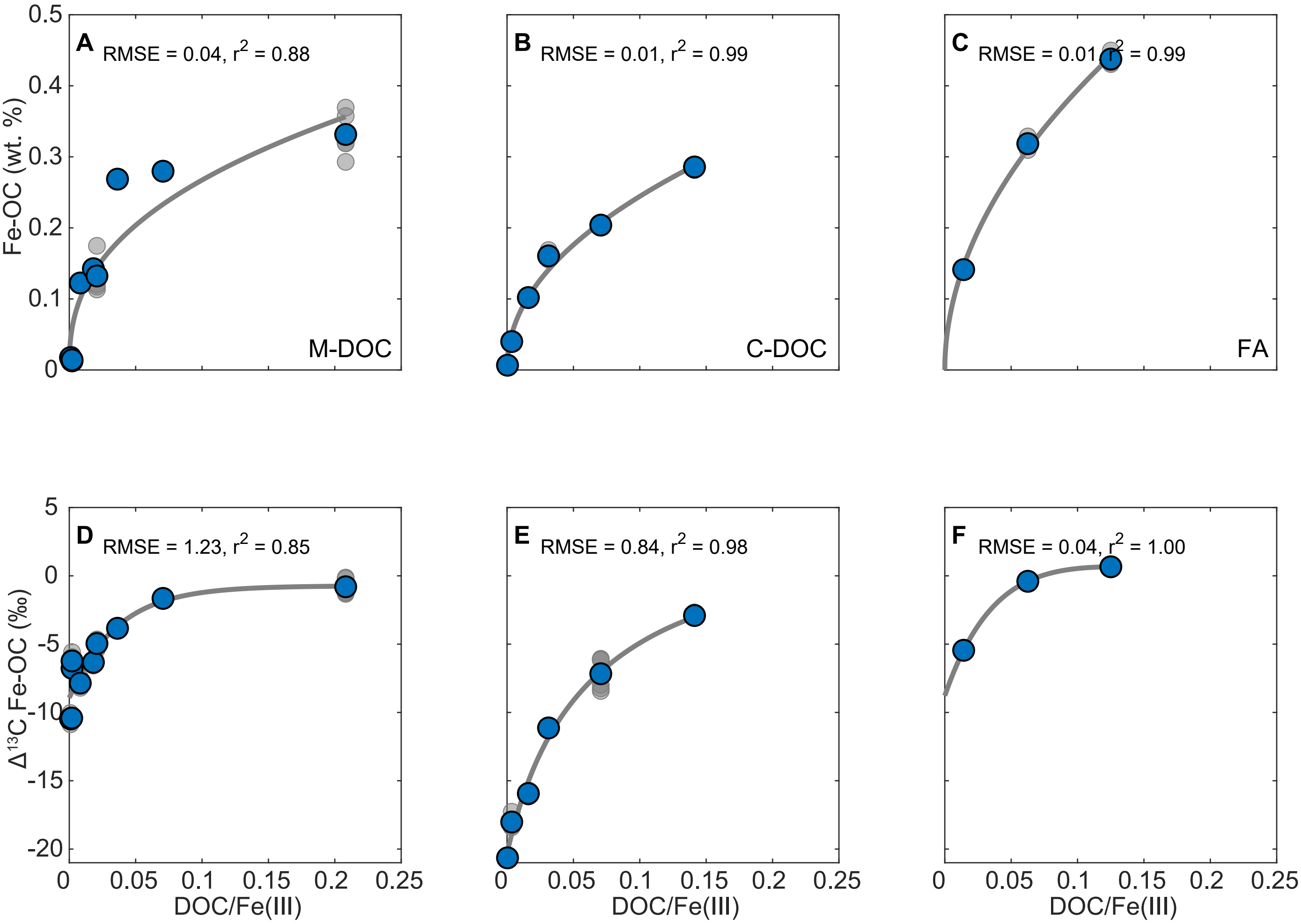

Fig. S19: Final hematite DOC/Fe(III) vs. Fe-OC response curves. (**A**-**C**) Fe-OC loadings (in wt %) and (**D**-**F**) isotope fractionations ($\Delta^{13}C = \delta^{13}C_{Fe-OC} - \delta^{13}C_{DOC}$, in ‰) as a function of DOC/Fe(III) ratio for (**A**, **D**) M-DOC, (**B**, **E**) C-DOC, and (**C**, **F**) FA as DOC sources. Experimental conditions were: 50 °C; pH = 8; $FeCl_3$ as Fe(III) source; DOC addition after ferrihydrite precipitation; aged (**A**, **D**) 5 to 30 days, (**B**, **E**) 5 to 14 days, or (**C**, **F**) 14 days; measurement of "−Fh/PC −ads. OC" fractions. Semi-transparent markers are individual analyses ($n = 3$ per sample) and opaque markers are sample means. Thick gray lines are best-fit OLS regressions, with reported regression uncertainty (r^2, RMSE).

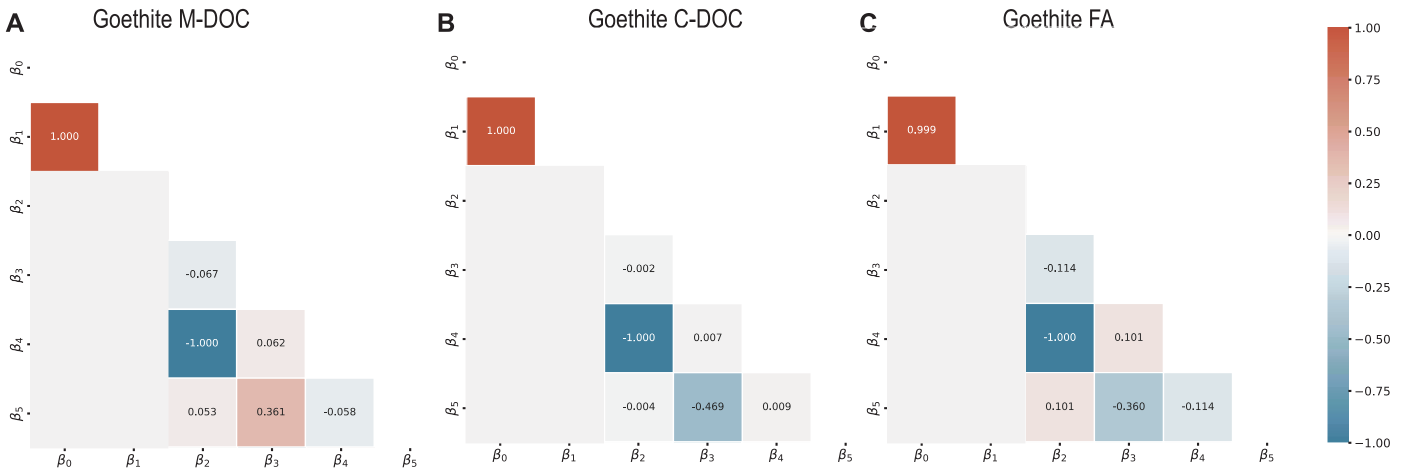

Fig. S20: Goethite calibration curve fit parameter correlation matrix. Correlations between fit parameters (Fe-OC loadings: β_0-β_1; Δ^{13}C fractionations: β_2-β_5) for goethite co-precipitated with (**A**) M-DOC, (**B**) C-DOC, and (**C**) FA. Numbers and colors represent correlation strength, ranging from -1 (strong negative correlation, blue) to $+1$ (strong positive correlation, red).

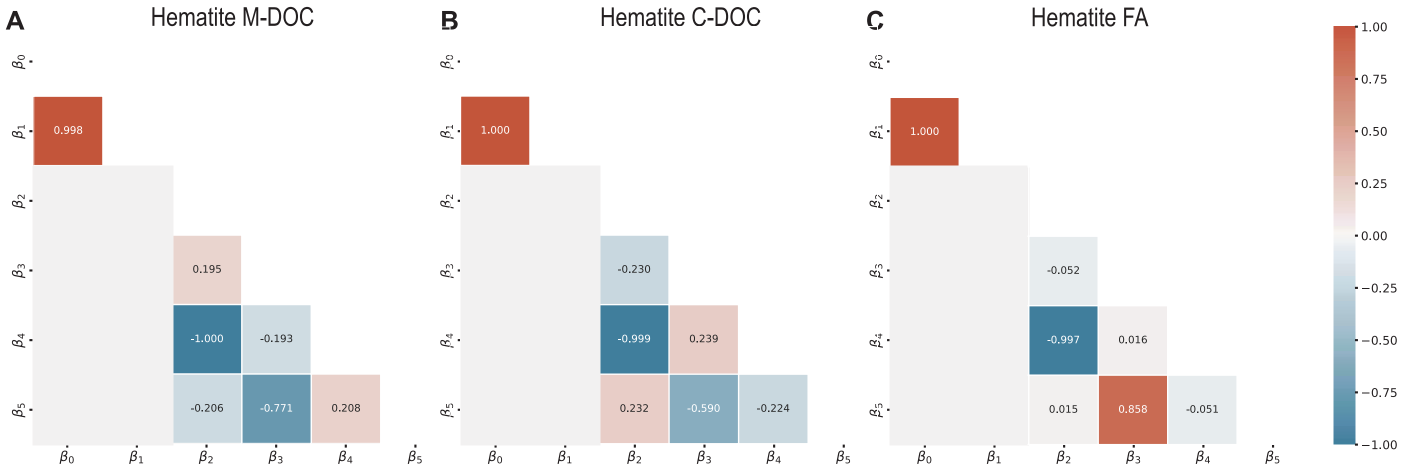

Fig. S21: Hematite calibration curve fit parameter correlation matrix. Correlations between fit parameters (Fe-OC loadings: β_0-β_1; Δ^{13}C fractionations: β_2-β_5) for hematite co-precipitated with (**A**) M-DOC, (**B**) C-DOC, and (**C**) FA. Numbers and colors represent correlation strength, ranging from -1 (strong negative correlation, blue) to $+1$ (strong positive correlation, red).

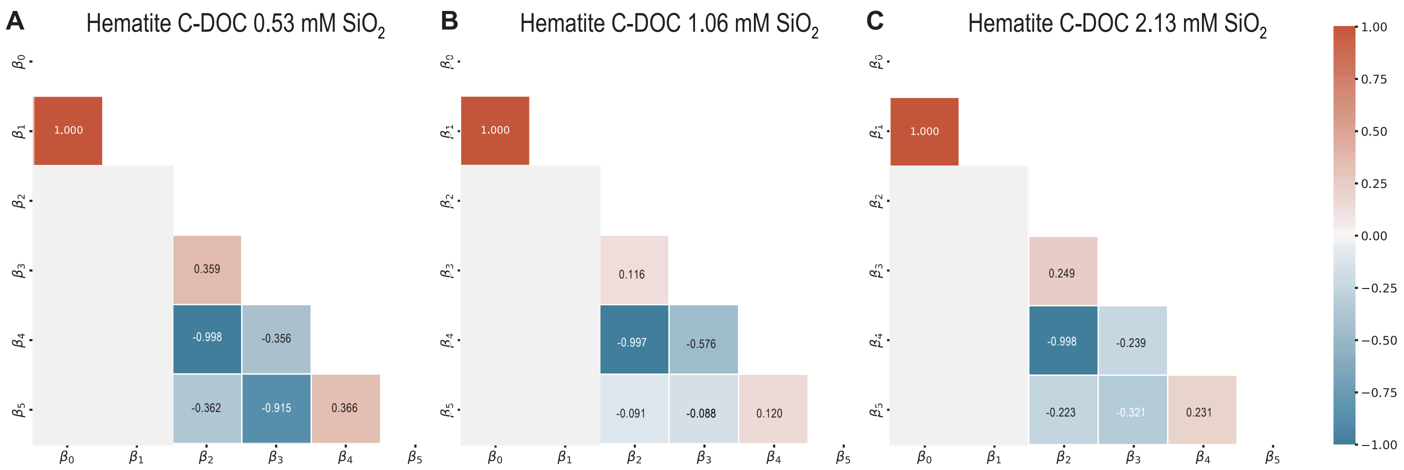

Fig. S22: Hematite calibration curve fit parameter correlation matrix with added SiO_2. Correlations between fit parameters (Fe-OC loadings: β_0–β_1; Δ^{13}C fractionations: β_2–β_5) for hematite co-precipitated with C-DOC in the presence of SiO_2 at concentrations of (**A**) 0.53 mM, 1.06 mM, and 2.13 mM. Numbers and colors represent correlation strength, ranging from -1 (strong negative correlation, blue) to $+1$ (strong positive correlation, red).

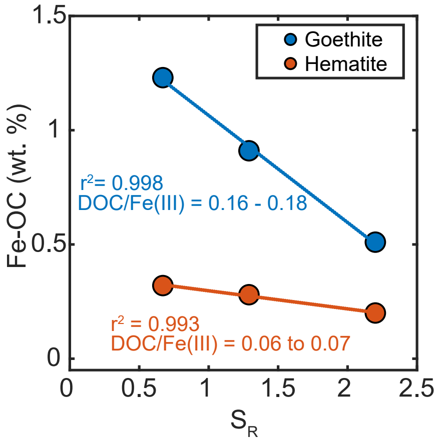

Fig. S23: DOC compositional control on Fe-OC loadings. Fe-OC loadings as a function of DOC source slope ratio (S_R) for goethite (blue) and hematite (orange) at select DOC/Fe(III) ratios. Thick blue (goethite) and orange (hematite) lines are best-fit OLS regressions, including coefficients of determination (r^2 values).

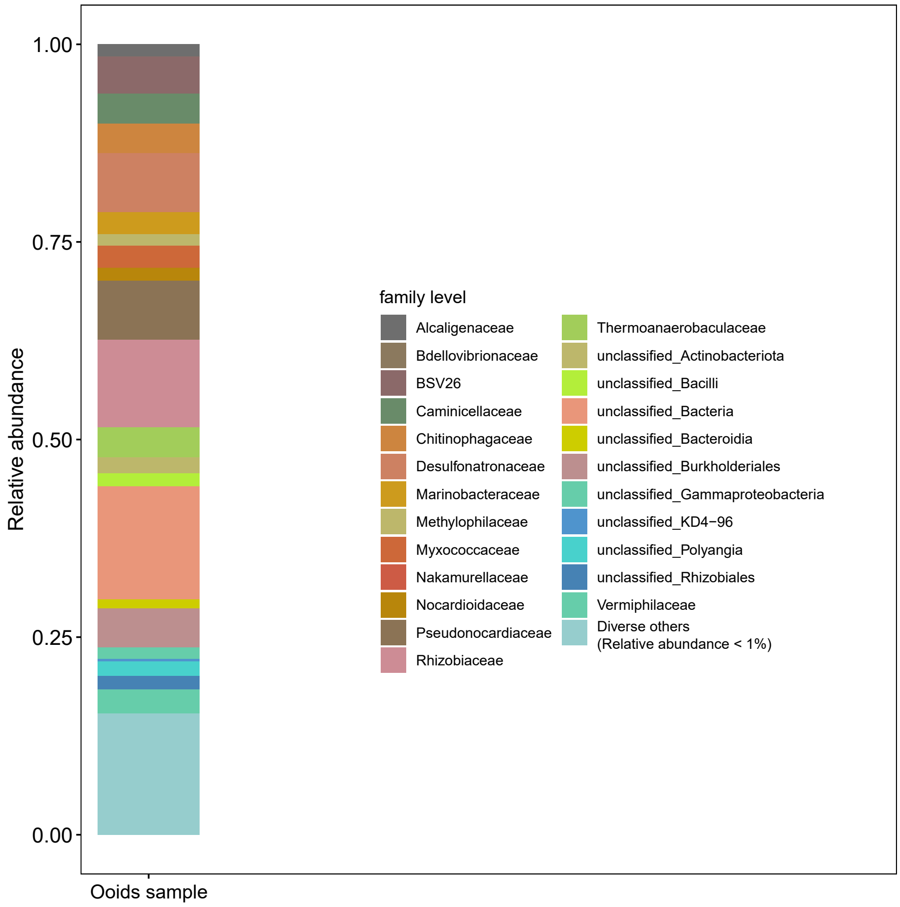

Fig. S24: Microbial community composition based on 16S rRNA gene amplicon sequencing. Colors represent relative abundance of microbial groups in a single successful DNA extract of non-sonicated modern ooids from Panarea Island, Italy[44], after filtering out reagent-contaminated taxa. "Diverse others" group represents microbial communities with $<1\%$ relative abundance. Importantly, well-known taxa for iron oxidation such as *Mariprofundus* (within Zetaproterobacteria)[138,139], *Gallionellaceae* (e.g., genera *Gallionella* and *Sideroxydans*; within Gammaproteobacteria)[140,141], *Pseudoalteromonadaceae* (within Gammaproteobacteria)[142] and *Hyphomonadaceae* (within Alphaproteobacteria)[143,144] were not detected. Thus, 16S rRNA gene while amplicon sequencing results cannot completely eliminate the possibility of iron-oxidizing bacteria in our ooid samples, they imply a microbial community in which this metabolism is rare. We interpret this as evidence that ooid-bound Fe-OC does not represent local, grain-scale microbial inputs.

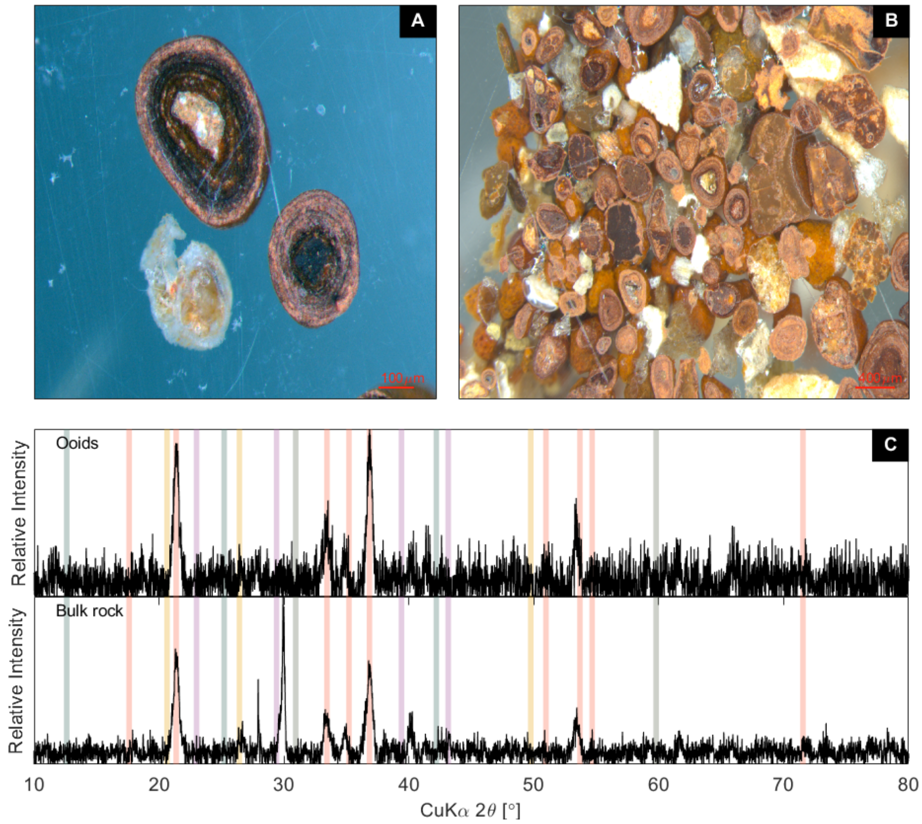

Fig. S25: Panarea Island, Italy petrography (sample IO-OM 1). (**A**-**B**) Optical microscopy images (SEM images were attempted for this sample but did not yield additional information due to porosity and epoxy resin artifacts) and (**C**) XRD diffractogram of bulk rock and individual ooids. Vertical bars in (**C**) refer to diagnostic XRD peaks for: red = goethite or hematite (depending on sample); orange = quartz; purple = dolomite; green = calcite; dark green= kaolinite/chamosite. The images reveal a vibrant core of ooids, primarily made up of rock fragments. This core is encased in alternating layers composed of goethite (bright) and amorphous iron oxyhydroxides, notably ferrihydrite (dark).

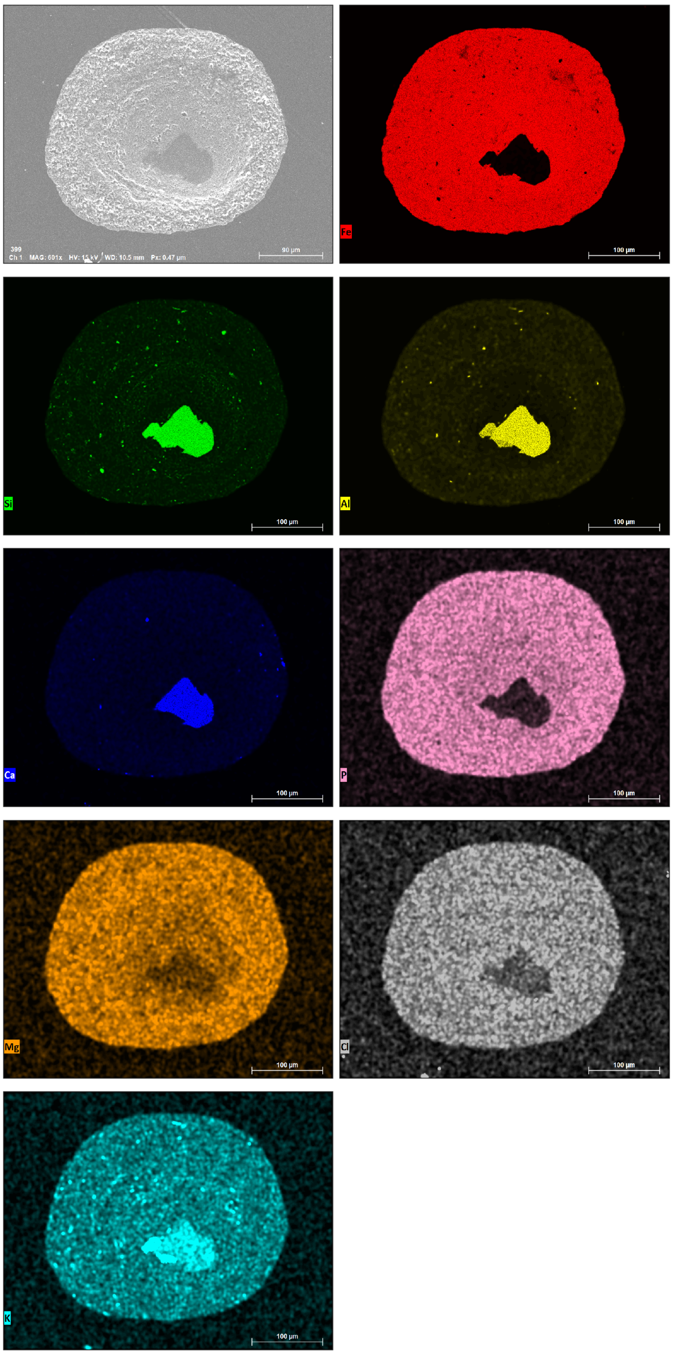

Fig. S26: Panarea Island, Italy SEM-EDS elemental maps (sample IO-OM 1). Element designations include Ca (blue), Fe (red), Mg (orange), Si (green), Cl (gray), K (teal), P (pink), and Al (yellow). Distinct features include Fe-rich ooid rims and Al-, Ca-, K-, and Si-rich cores, indicating the presence of Fe (oxihydr)oxide laminae and an andesitic core. The associated presence of Mg, P, and Cl with the ooids suggests their formation in a contemporary marine environment, likely influenced by hydrothermal vent fluids. All elements that yielded a signal above the noise threshold are shown.

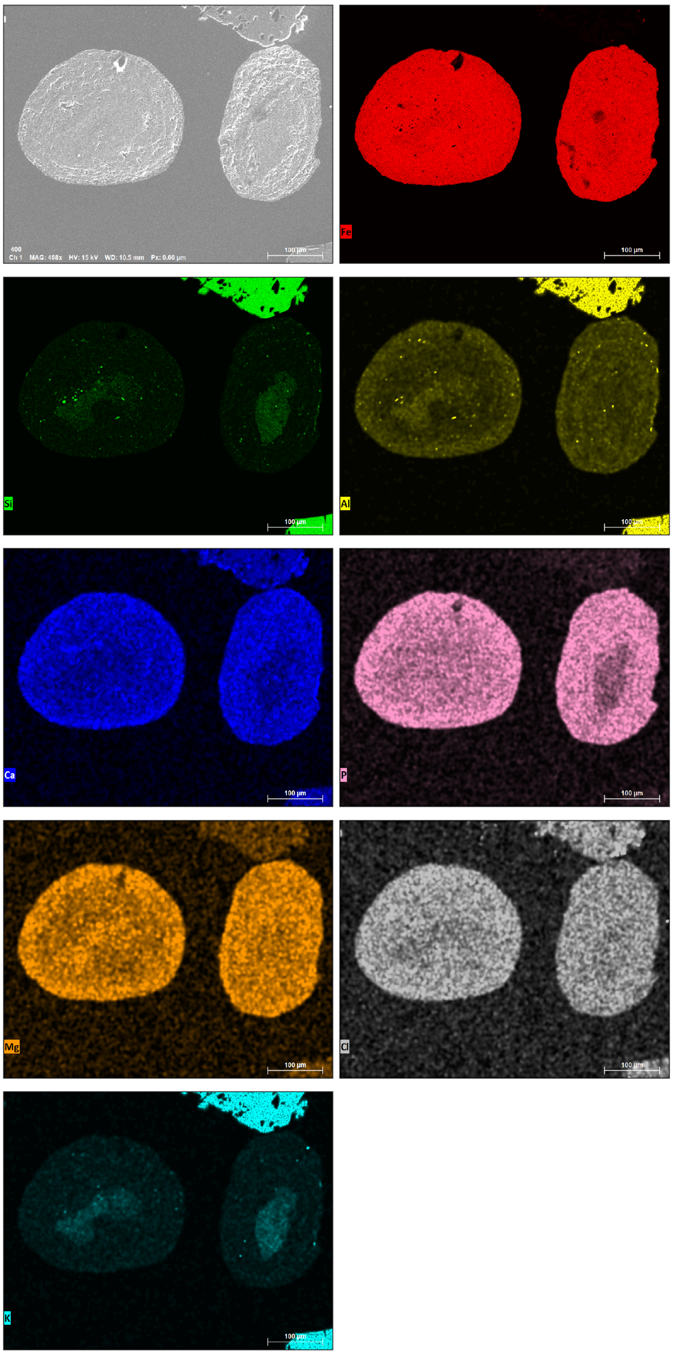

Fig. S27: Panarea Island, Italy SEM-EDS elemental maps (sample IO-OM 1). As for Fig. S26 but showing different ooids from the same sample.

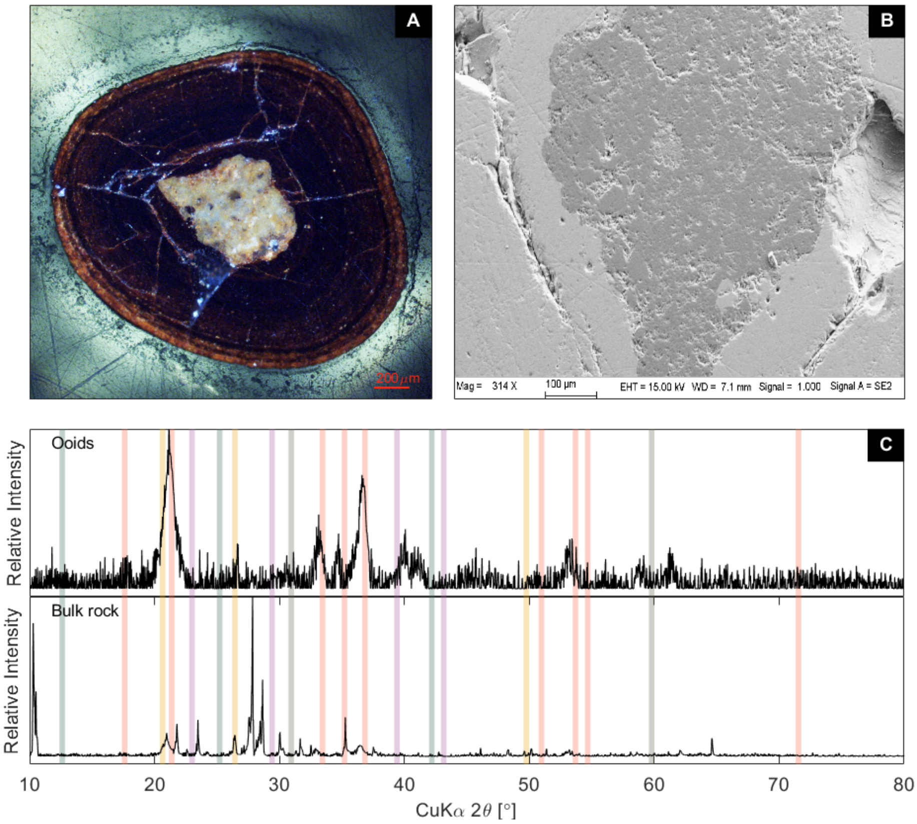

Fig. S28: Mahengetang Island, Indonesia petrography (sample IO-OM 4). (**A**) Optical and (**B**) electron microscopy images and (**C**) XRD diffractogram of bulk rock and individual ooids. Vertical bars in (**C**) are the same as described in Fig. S25. The images reveal an ooid core, primarily made up of andesite fragments. This core is encased in alternating layers composed of goethite (bright) and amorphous iron oxyhydroxides, notably ferrihydrite (dark).

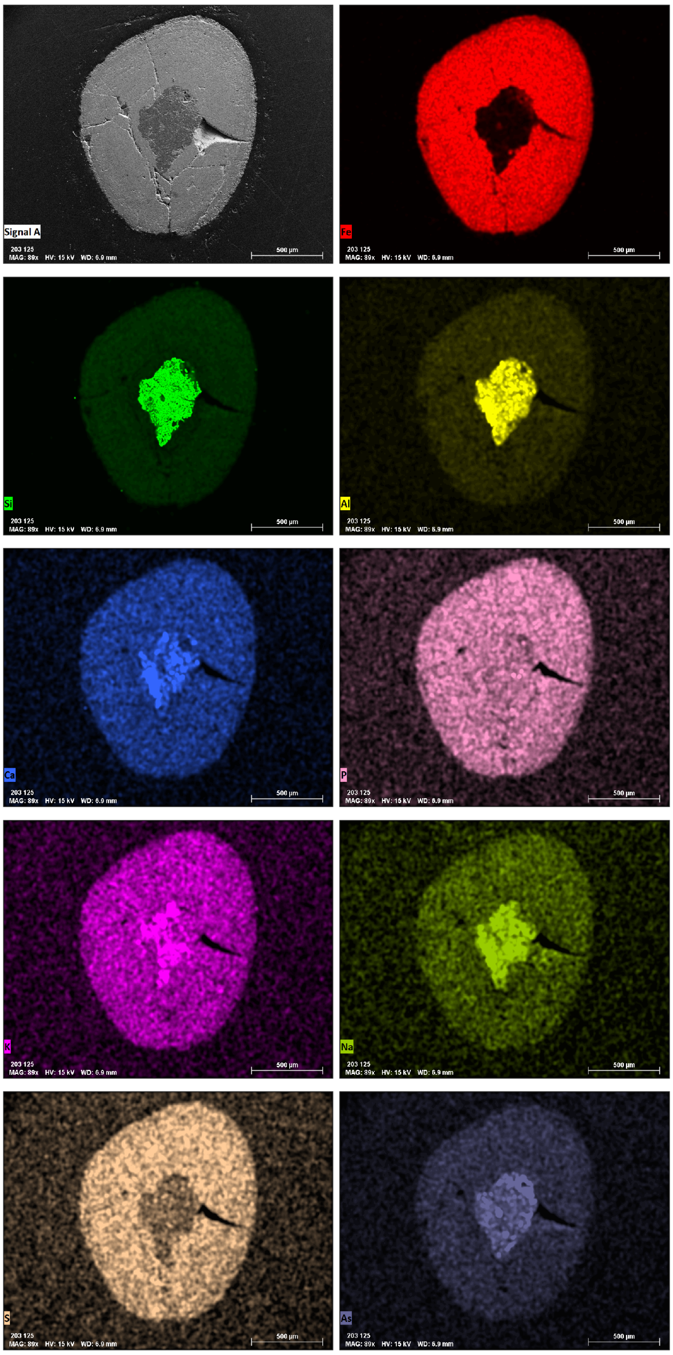

Fig. S29: Mahengetang Island, Indonesia SEM-EDS elemental maps (sample IO-OM 4). Element designations include Ca (blue), Fe (red), Mg (orange), Si (green), Cl (gray), K (purple), P (pink), Na (bright green), S (beige), As (purple), and Al (yellow). Distinct features include Fe-rich ooid rims and Al-, Na-, Ca-, and Si-rich cores. The associated presence of P and S with the ooids suggests their formation in a contemporary marine environment, likely influenced by hydrothermal vent fluids. All elements that yielded a signal above the noise threshold are shown.

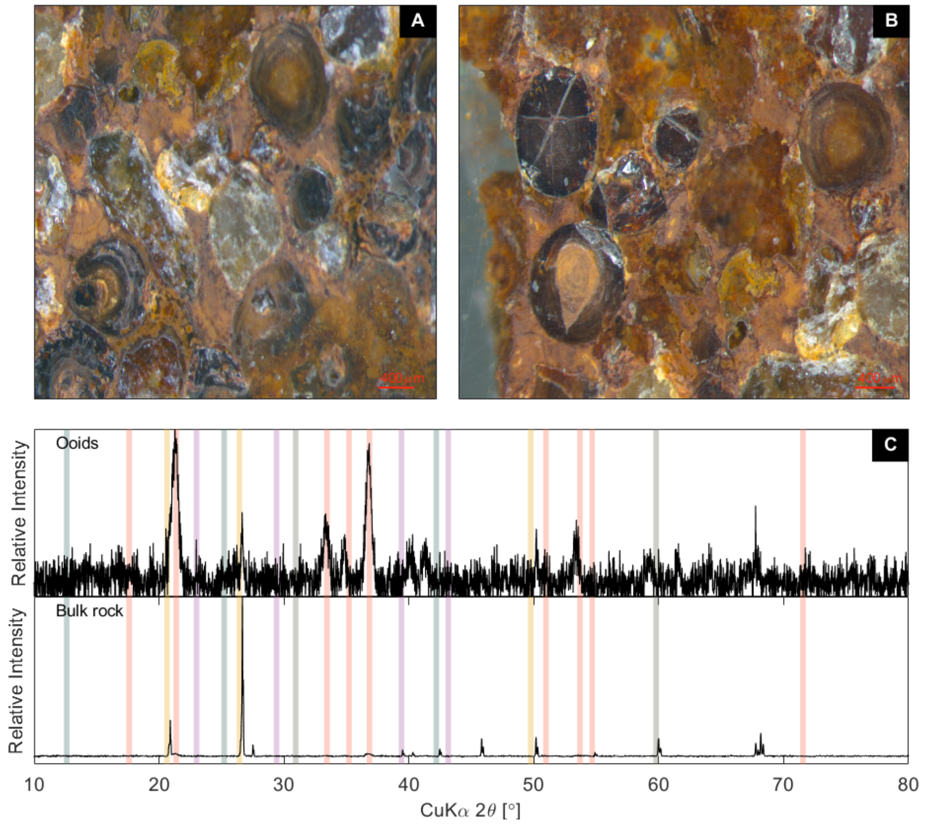

Fig. S30: Lisakovsk deposit, Kazakhstan petrography (sample IO-OM 5). (**A-B**) Optical microscopy images (SEM images were attempted for this sample but did not yield additional information due to porosity and epoxy resin artifacts) and (**C**) XRD diffractogram of bulk rock and individual ooids. Vertical bars in (**C**) are the same as described in Fig. S25. The images display a spherical goethite ooids embedded in a quartz and carbonate rich cement.

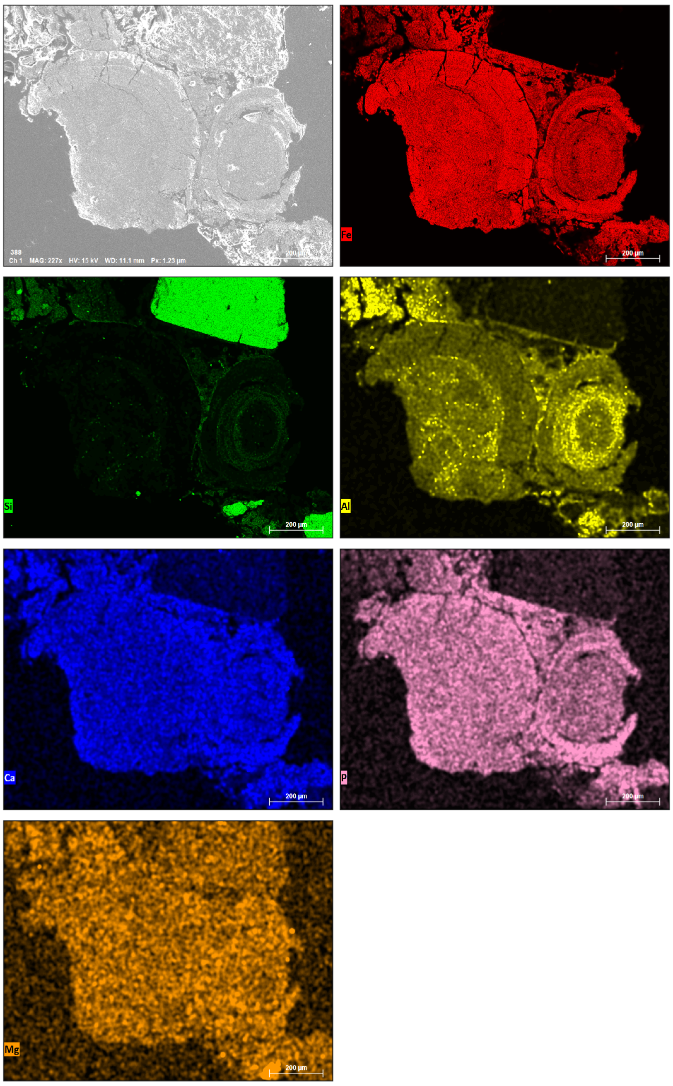

Fig. S31: Lisakovsk deposit, Kazakhstan SEM-EDS elemental maps (sample IO-OM 5). Element designations include Ca (blue), Fe (red), Mg (orange), Si (green), P (pink), and Al (yellow). Distinct features include Fe-rich ooid rims and Al- and Si-rich cores, suggesting a minor silicate presence. The associated presence of P, Ca, and Mg surrounding the ooids indicates the existence of carbonate-rich cement, which aligns with results from bulk rock XRD analysis. All elements that yielded a signal above the noise threshold are shown.

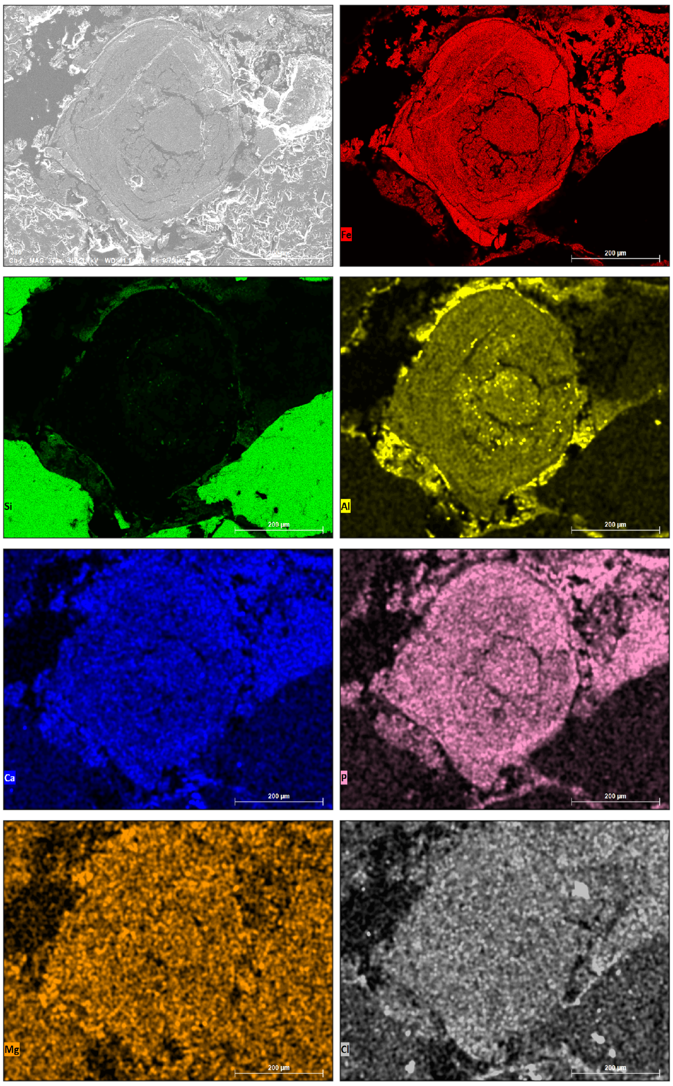

Fig. S32: Lisakovsk deposit, Kazakhstan SEM-EDS elemental maps (sample IO-OM 5). As for Fig. S31 but showing different ooids from the same sample.

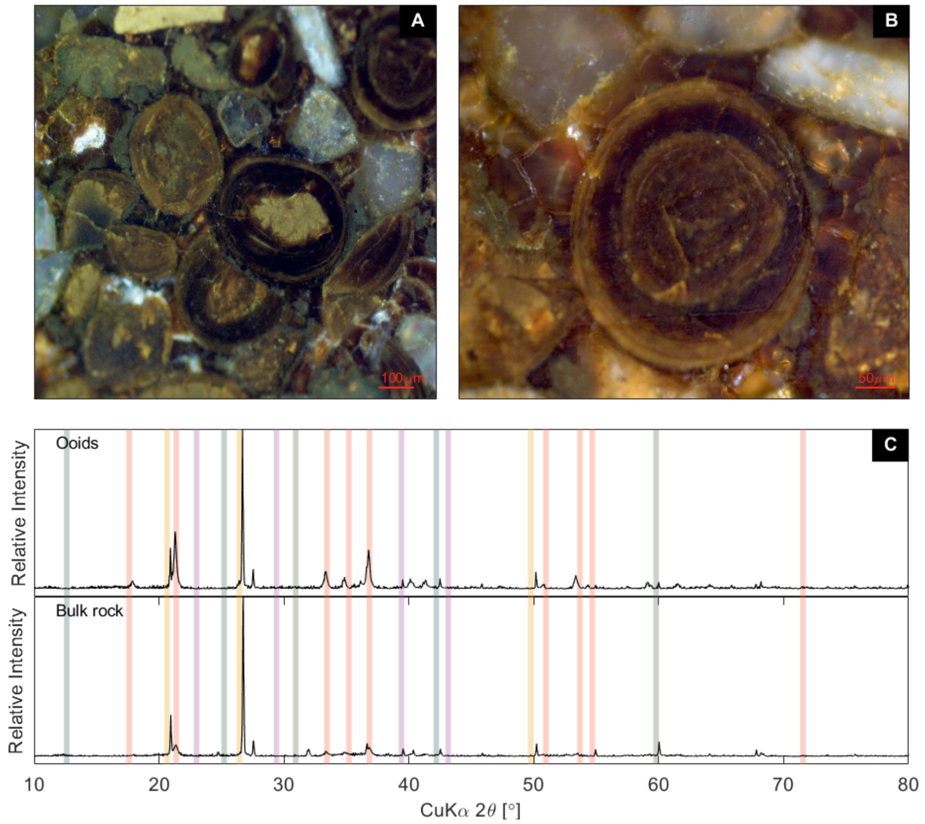

Fig. S33: Bakchar Horizon, Lyulinvor Fm, Russia petrography (sample IO-OM 9). (**A-B**) Optical microscopy images (SEM images were attempted for this sample but did not yield additional information due to porosity and epoxy resin artifacts) and (**C**) XRD diffractogram of bulk rock and individual ooids. Vertical bars in (**C**) are the same as described in Fig. S25. The images display spherical goethite ooids embedded in sandstone cemented by carbonate.

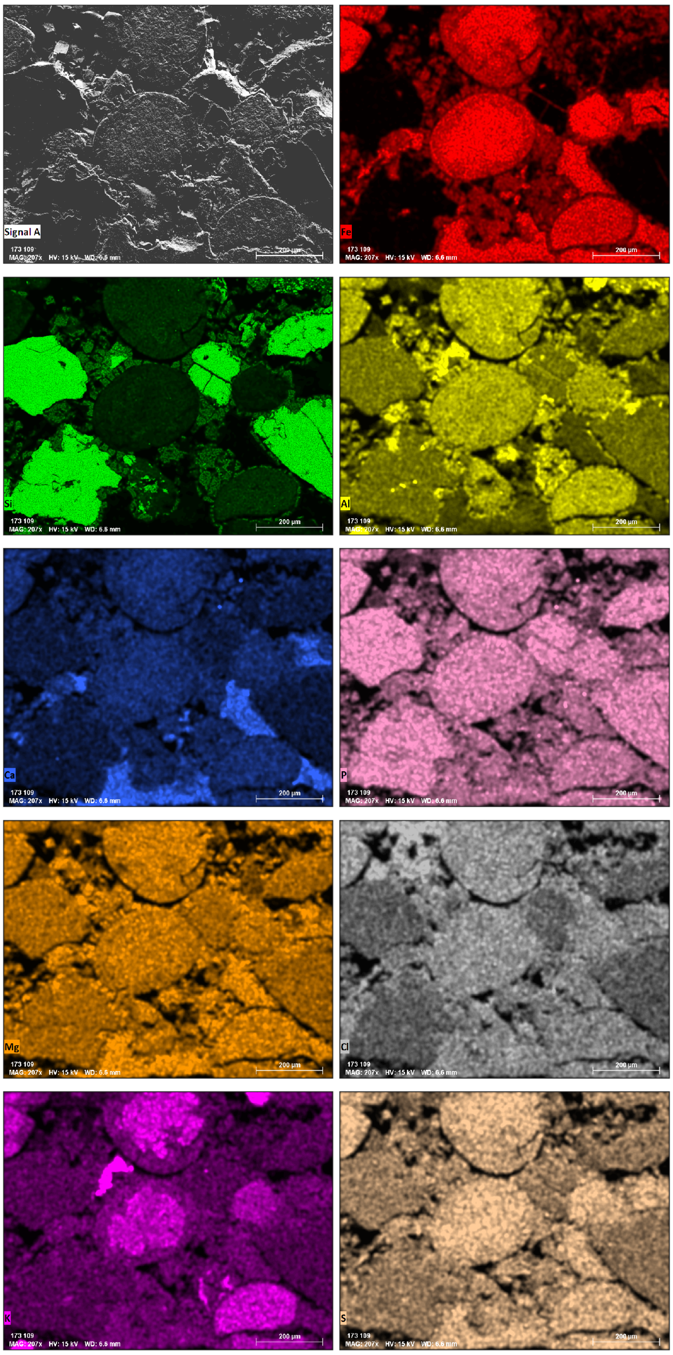

Fig. S34: Bakchar Horizon, Lyulinvor Fm, Russia SEM-EDS elemental maps (sample IO-OM 9). Element designations include Ca (blue), Fe (red), Mg (orange), Si (green), Cl (gray), P (pink), K (purple), S (beige), and Al (yellow). Distinct features include Fe-rich ooids sitting in a matrix of Si-rich quartz grains. The associated presence of Al, P, S, and K indicates the presence of Fe-rich silicates. All elements that yielded a signal above the noise threshold are shown.

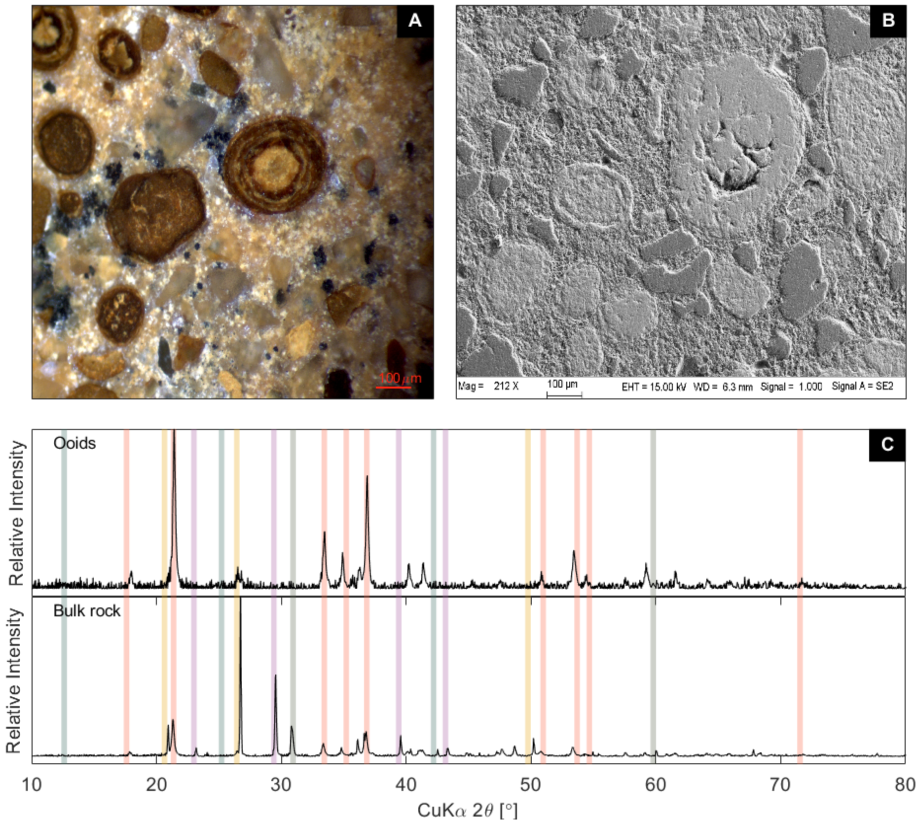

Fig. S35: Hatira Fm, Israel petrography (sample IO-OM 16, subsample 1). (**A**) Optical and (**B**) electron microscopy images and (**C**) XRD diffractogram of bulk rock and individual ooids. Vertical bars in (**C**) are the same as described in Fig. S25. The images display spherical goethite ooids embedded in calcite and dolomite matrix and several quartz grains.

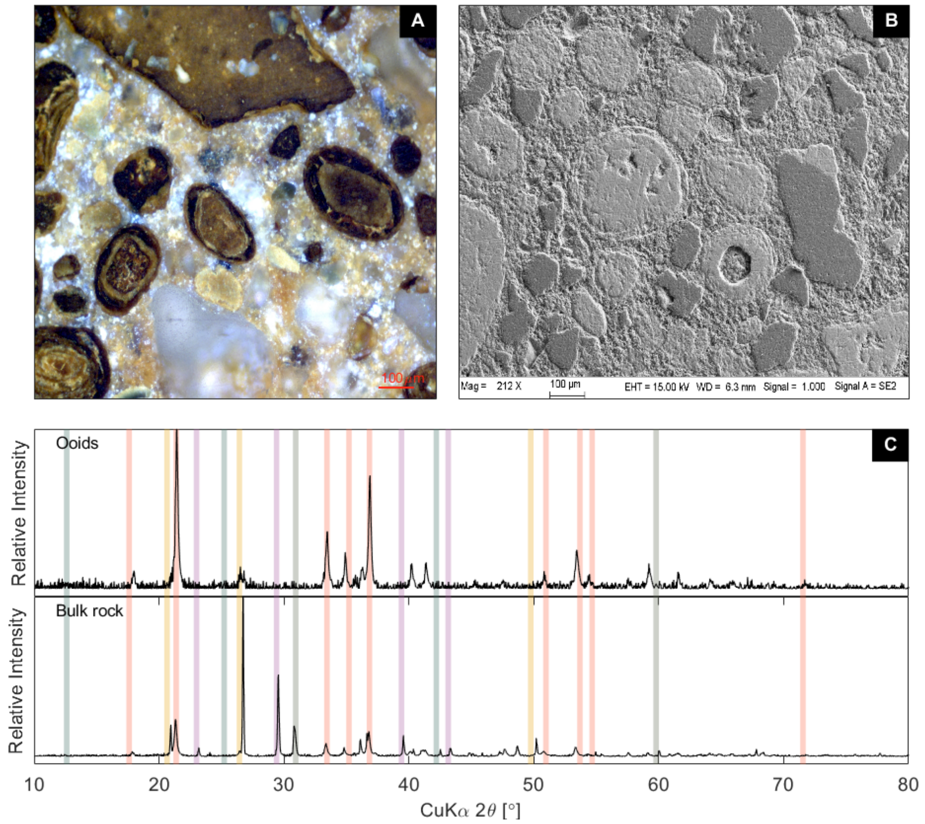

Fig. S36: Hatira Fm, Israel petrography (sample IO-OM 16, subsample 2). As for Fig. S35 but showing ooids from a different subsample of the same sample. Vertical bars in (**C**) are the same as described in Fig. S25.

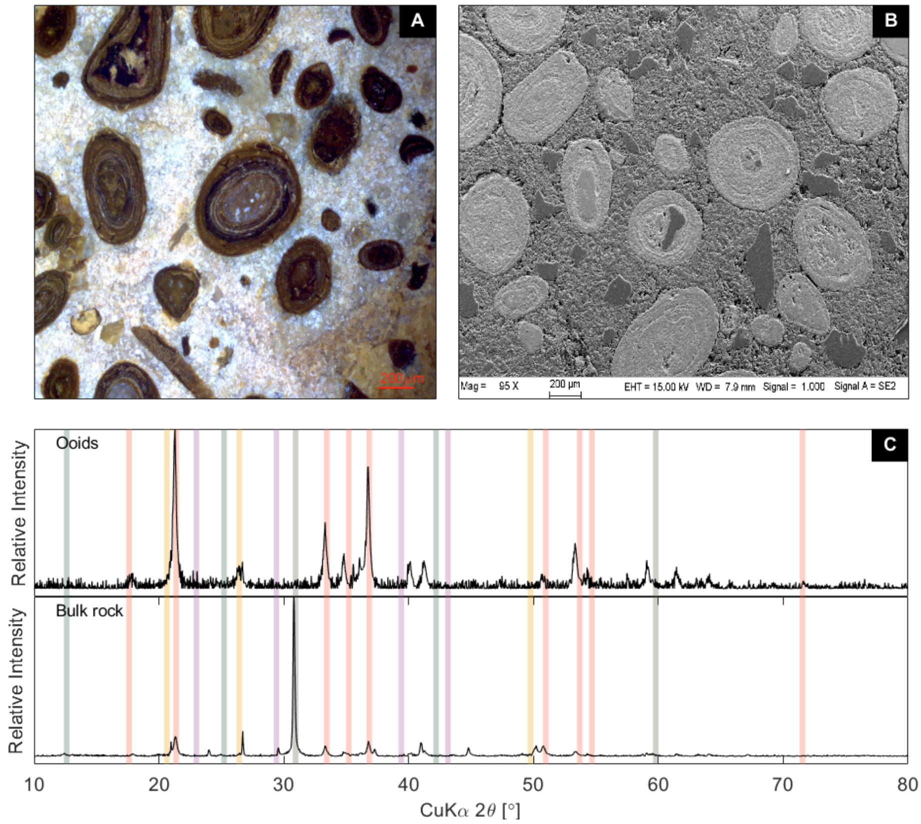

Fig. S37: Hatira Fm, Israel petrography (sample IO-OM 17). As for Fig. S35 but showing ooids from a different sample from the same formation. Vertical bars in (**C**) are the same as described in Fig. S25.

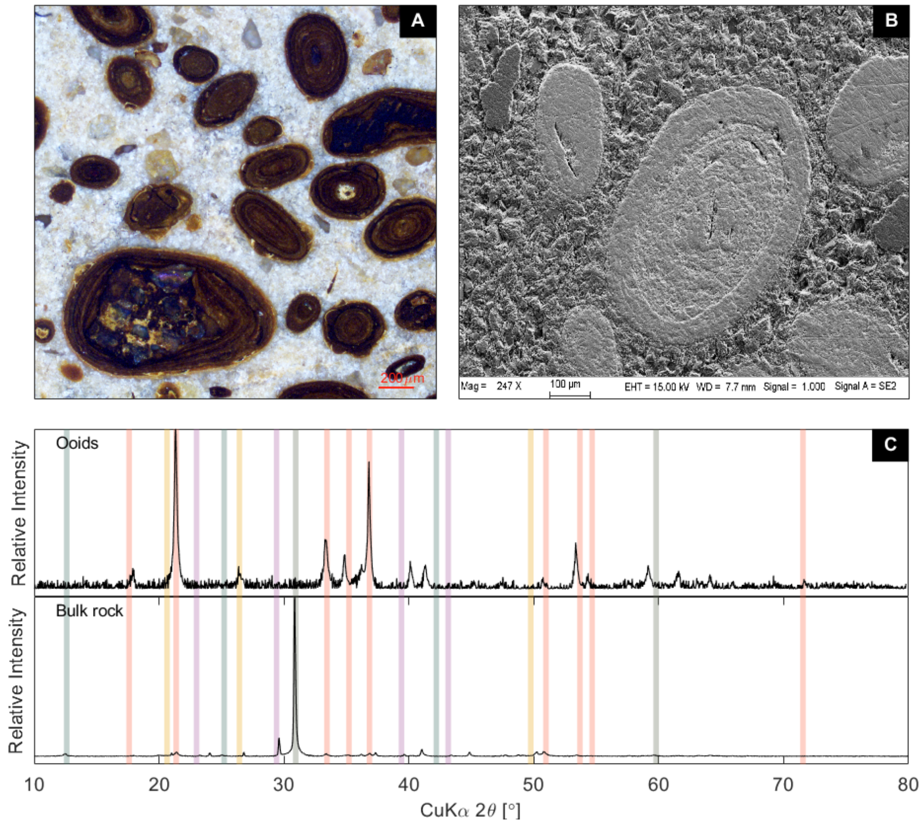

Fig. S38: Hatira Fm, Israel petrography (sample IO-OM 20, subsample 1). As for Fig. S35 but showing ooids from a different sample from the same formation. Vertical bars in (**C**) are the same as described in Fig. S25.

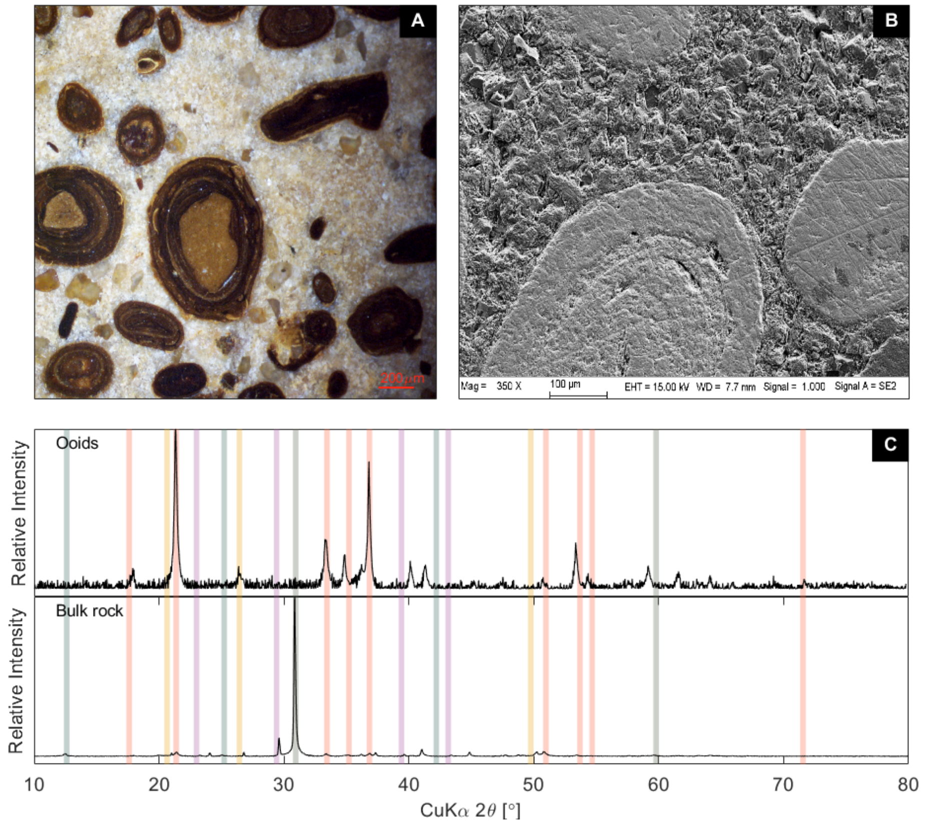

Fig. S39: Hatira Fm, Israel petrography (sample IO-OM 20, subsample 2). As for Fig. S35 but showing ooids from a different sample from the same formation (different subsample from the same sample as Fig. S38). Vertical bars in (**C**) are the same as described in Fig. S25.

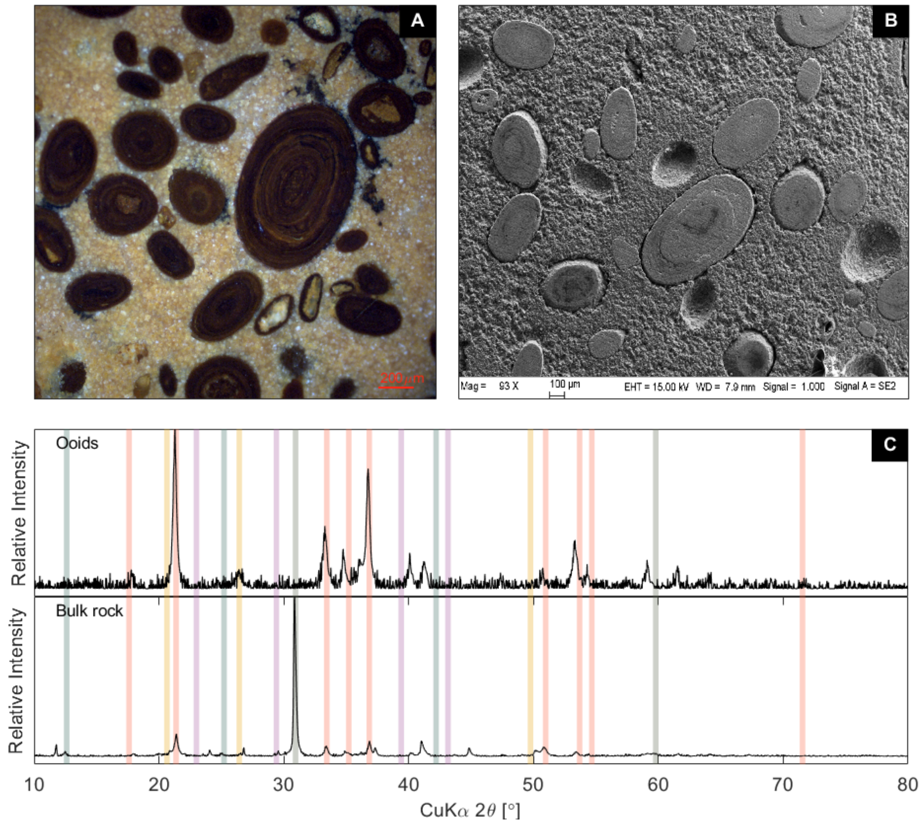

Fig. S40: Hatira Fm, Israel petrography (sample IO-OM 26). As for Fig. S35 but showing ooids from a different sample from the same formation. Vertical bars in (**C**) are the same as described in Fig. S25.

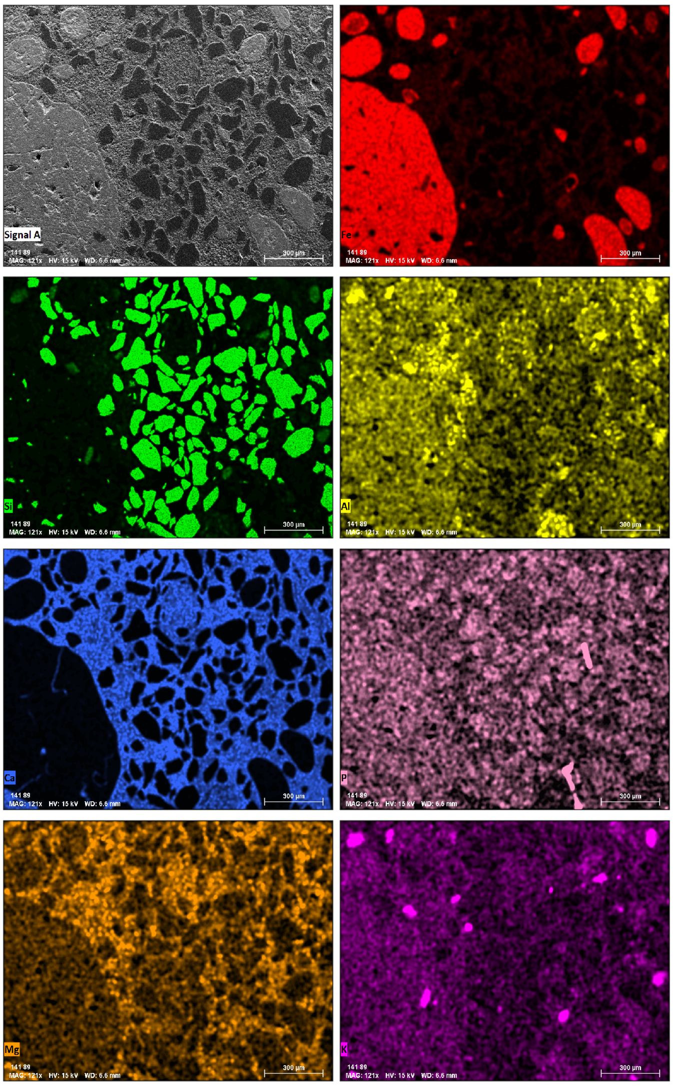

Fig. S41: Hatira Fm, Israel SEM-EDS elemental maps (sample IO-OM 16, subsample 1). Element designations include Ca (blue), Fe (red), Mg (orange), Si (green), P (pink), K (purple), and Al (yellow). Distinct features include Fe-rich ooids embedded in a Ca- and Mg-rich carbonate cement. All elements that yielded a signal above the noise threshold are shown.

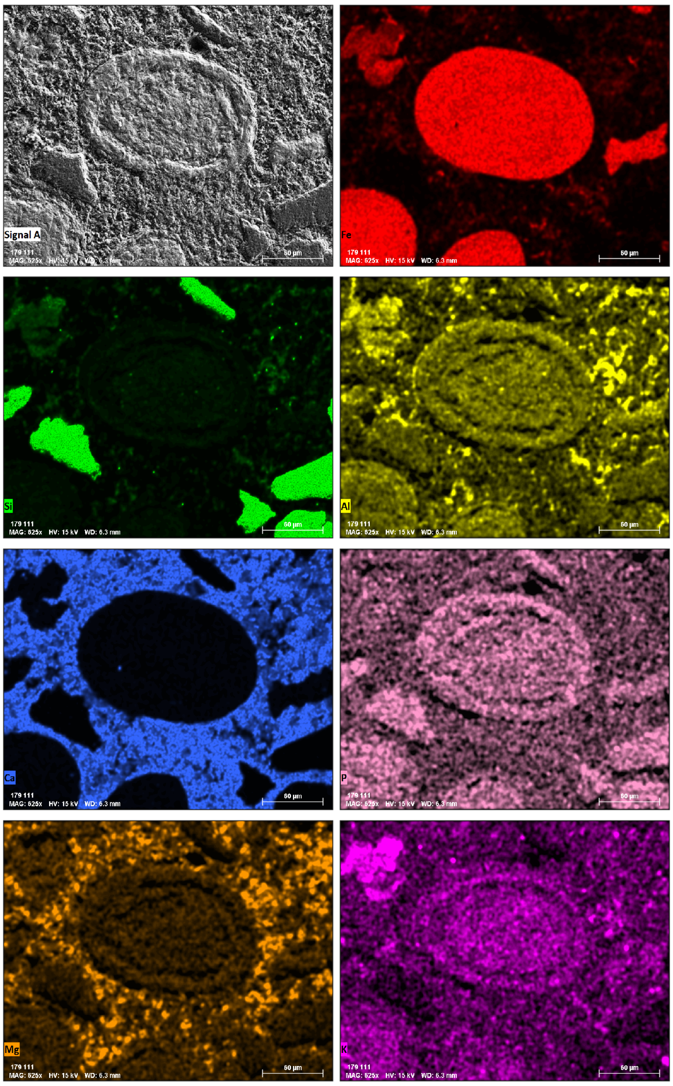

Fig. S42: Hatira Fm, Israel SEM-EDS elemental maps (sample IO-OM 16, subsample 2). As for Fig. S41 but showing ooids from a different subsample of the same sample.

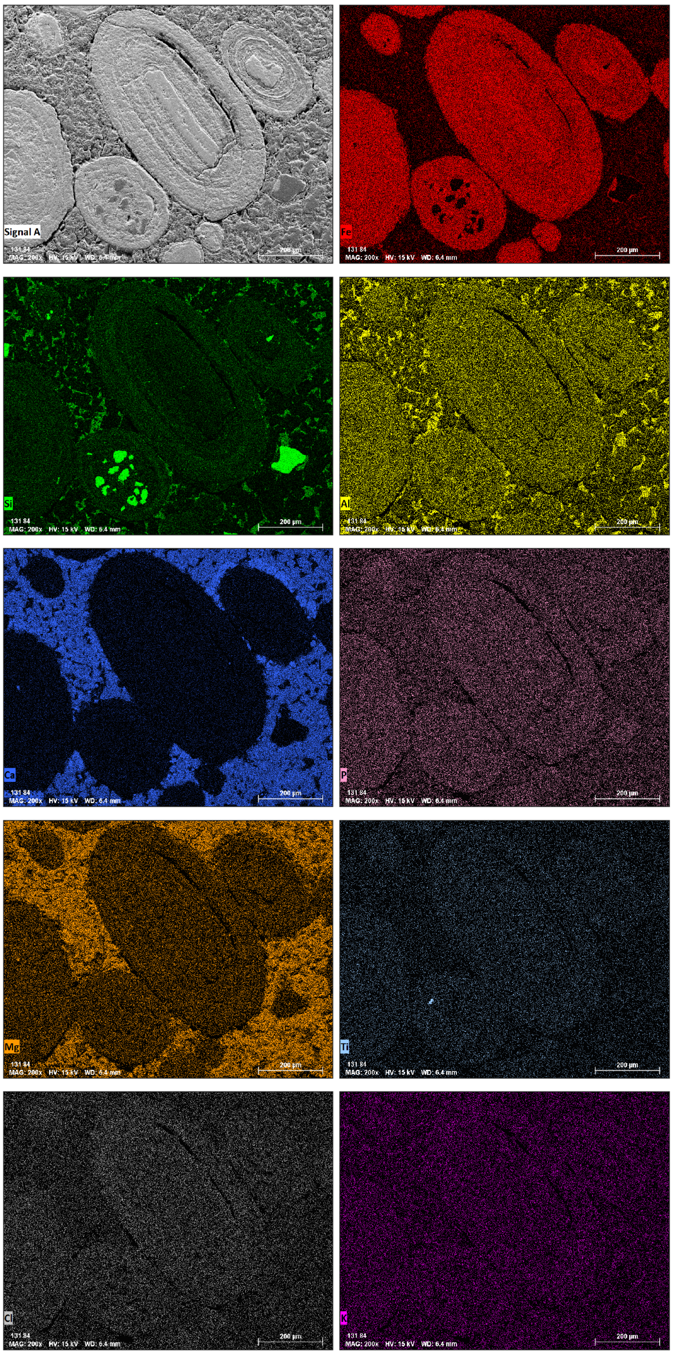

Fig. S43: Hatira Fm, Israel SEM-EDS elemental maps (sample IO-OM 17). As for Fig. S41 but showing ooids from a different sample from the same formation and including element designations for Ti (light blue) and Cl (gray).

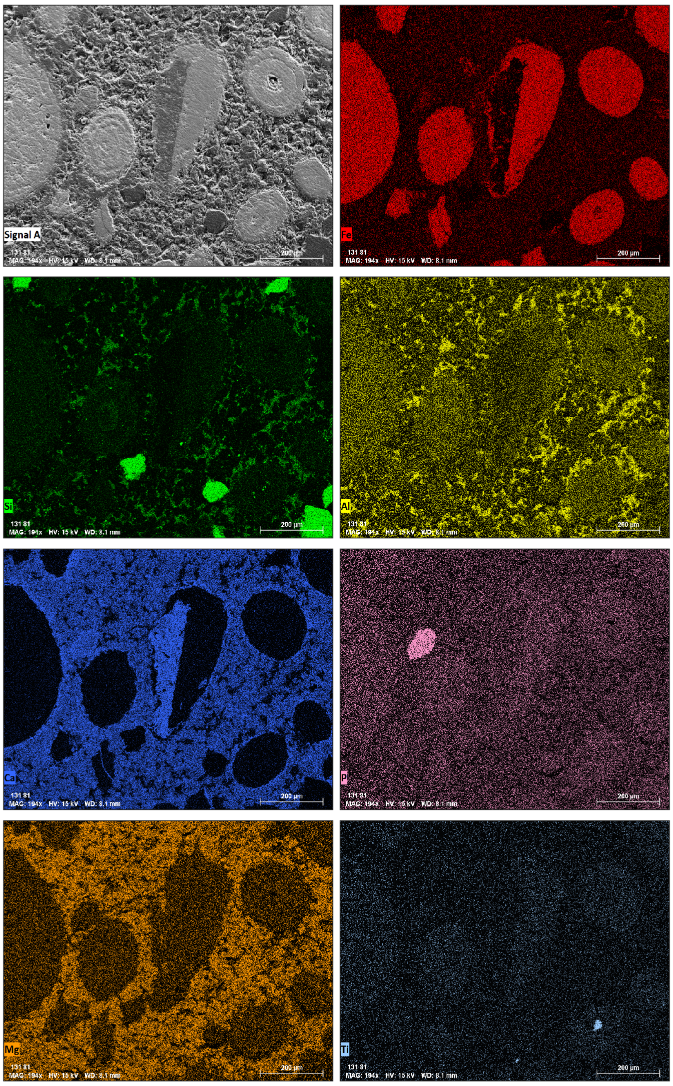

Fig. S44: Hatira Fm, Israel SEM-EDS elemental maps (sample IO-OM 20, subsample 1). As for Fig. S41 but showing ooids from a different sample from the same formation, omitting element designations for K and including element designations for Ti (light blue). Note the neo-formation of (Mg-poor) calcite replacing goethite.

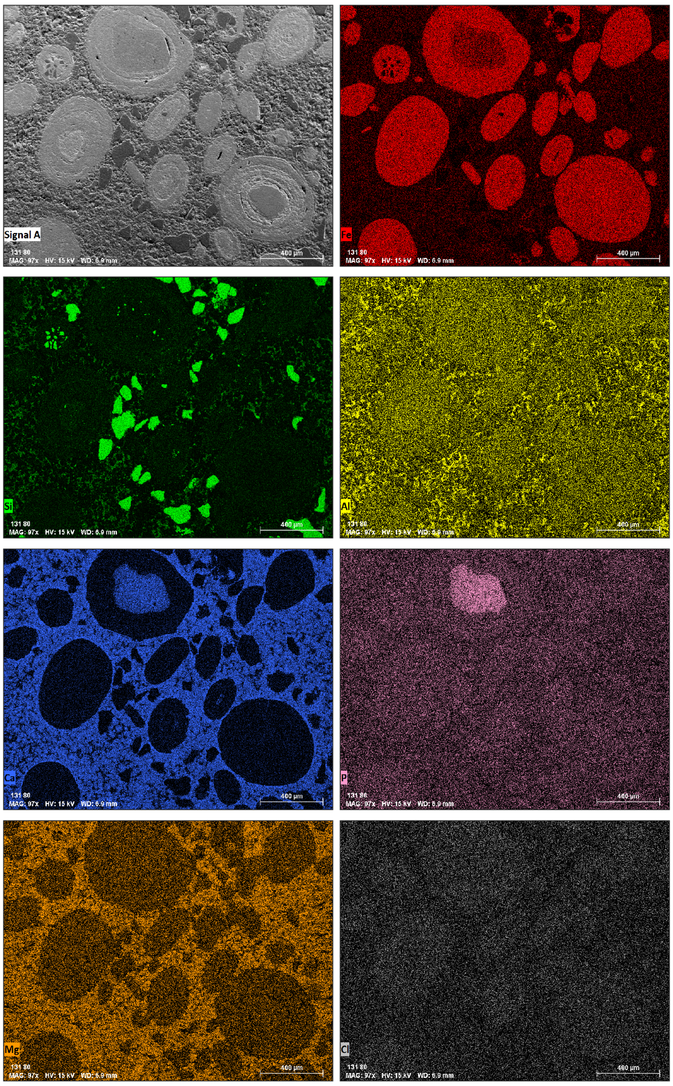

Fig. S45: Hatira Fm, Israel SEM-EDS elemental maps (sample IO-OM 20, subsample 2). As for Fig. S41 but showing ooids from a different sample from the same formation (different subsample from the same sample as Fig. S44), omitting element designations for K and including element designations for Cl (gray). Note the Ca- and P-rich core of one goethite ooid.

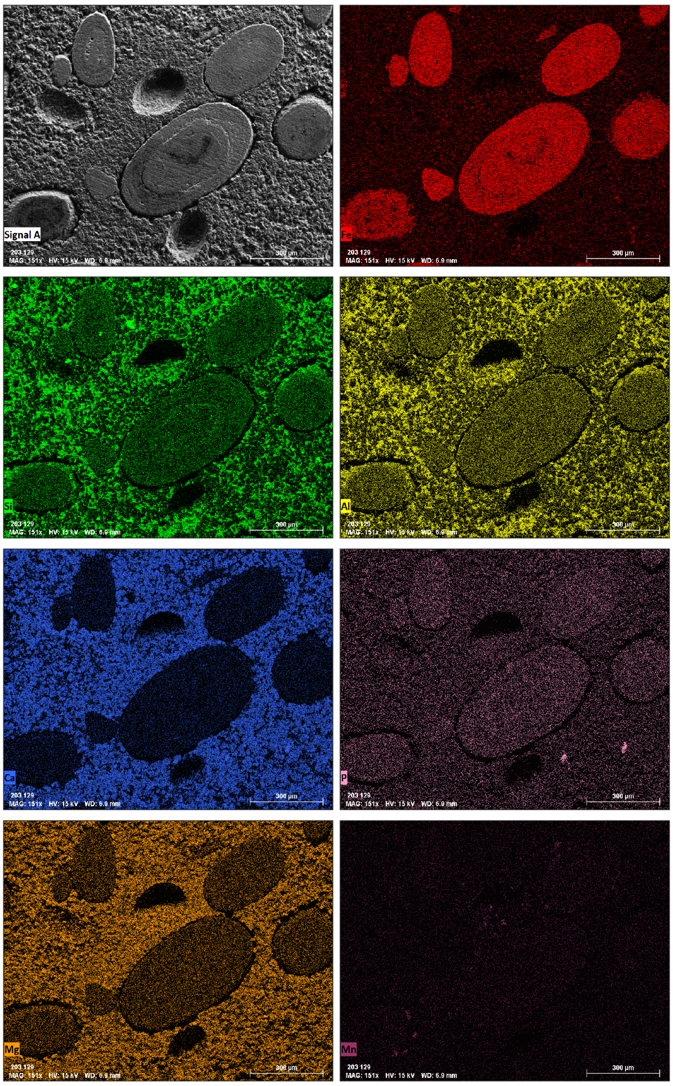

Fig. S46: Hatira Fm, Israel SEM-EDS elemental maps (sample IO-OM 26). As for Fig. S41 but showing ooids from a different sample from the same formation and including element designations for Mn (fuchsia). Note the Ca- and P-rich core of one goethite ooid.

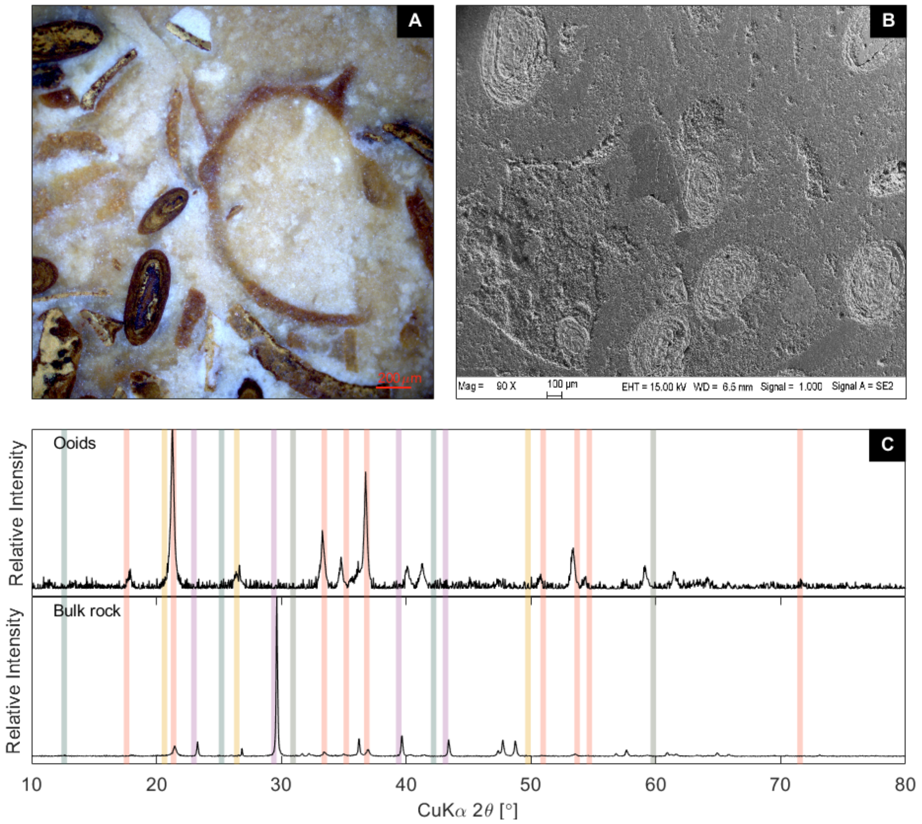

Fig. S47: Hidra Fm, Israel petrography (sample IO-OM 34, subsample 1). (**A**) Optical and (**B**) electron microscopy images and (**C**) XRD diffractogram of bulk rock and individual ooids. Vertical bars in (**C**) are the same as described in Fig. S25. The images display matrix-supported spherical goethite ooids in a calcite matrix filled with bivalve tests.

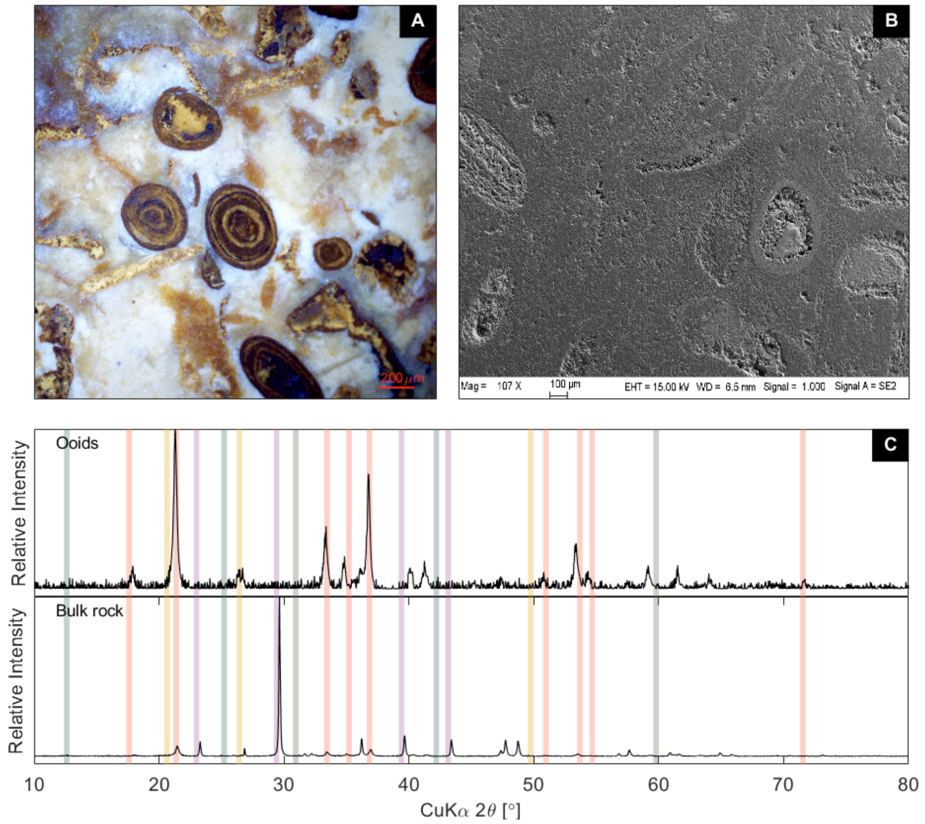

Fig. S48: Hidra Fm, Israel petrography (sample IO-OM 34, subsample 2). As for Fig. S47 but showing ooids from a different subsample of the same sample. Vertical bars in (**C**) are the same as described in Fig. S25.

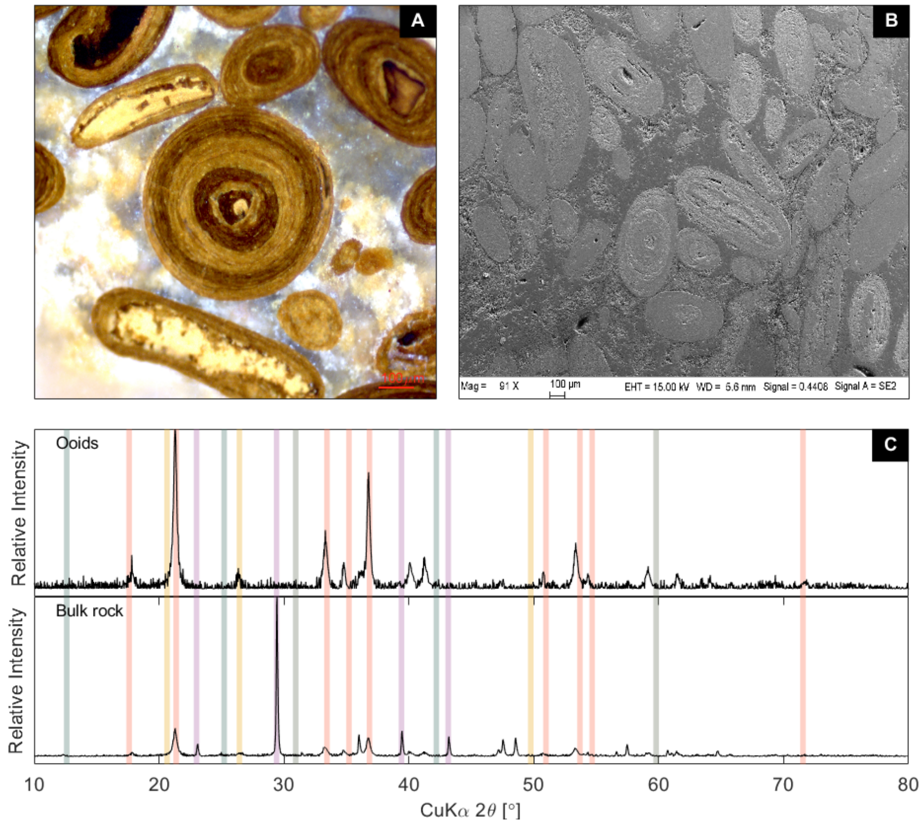

Fig. S49: Hidra Fm, Israel petrography (sample IO-OM 35, subsample 1). As for Fig. S47 but showing ooids from a different sample from the same formation. Vertical bars in (**C**) are the same as described in Fig. S25.

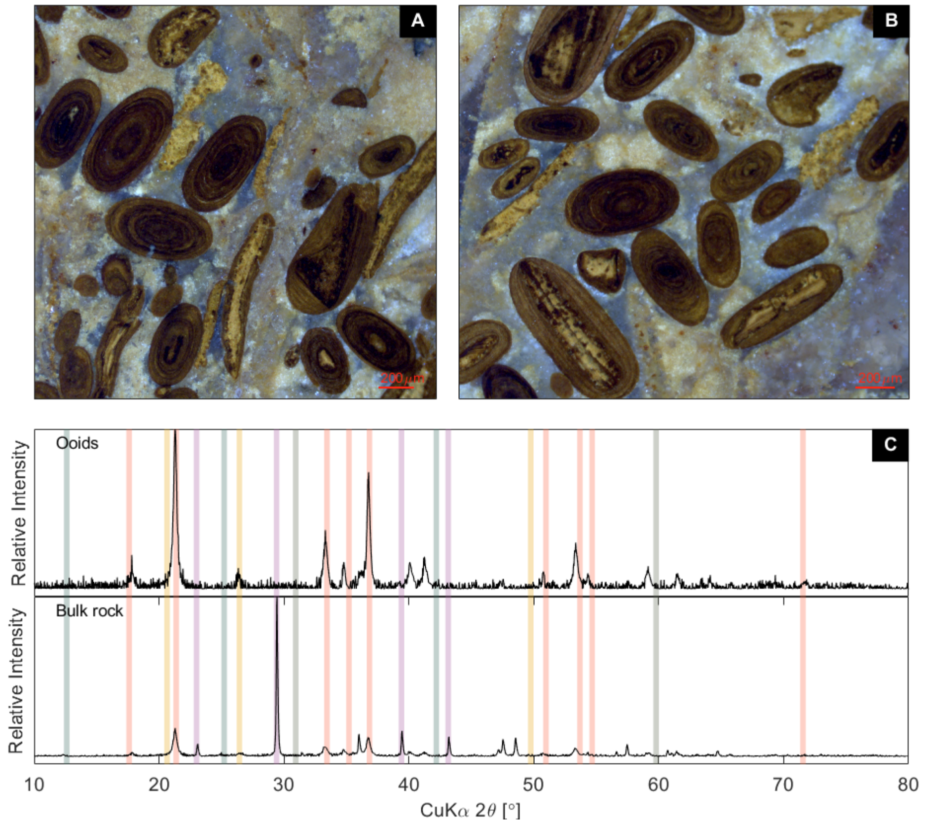

Fig. S50: Hidra Fm, Israel petrography (sample IO-OM 35, subsample 2). As for Fig. S47 but showing ooids from a different sample from the same formation (different subsample from the same sample as Fig. S49). Vertical bars in (**C**) are the same as described in Fig. S25. SEM images were attempted for this sample but did not yield additional information due to porosity and epoxy resin artifacts.

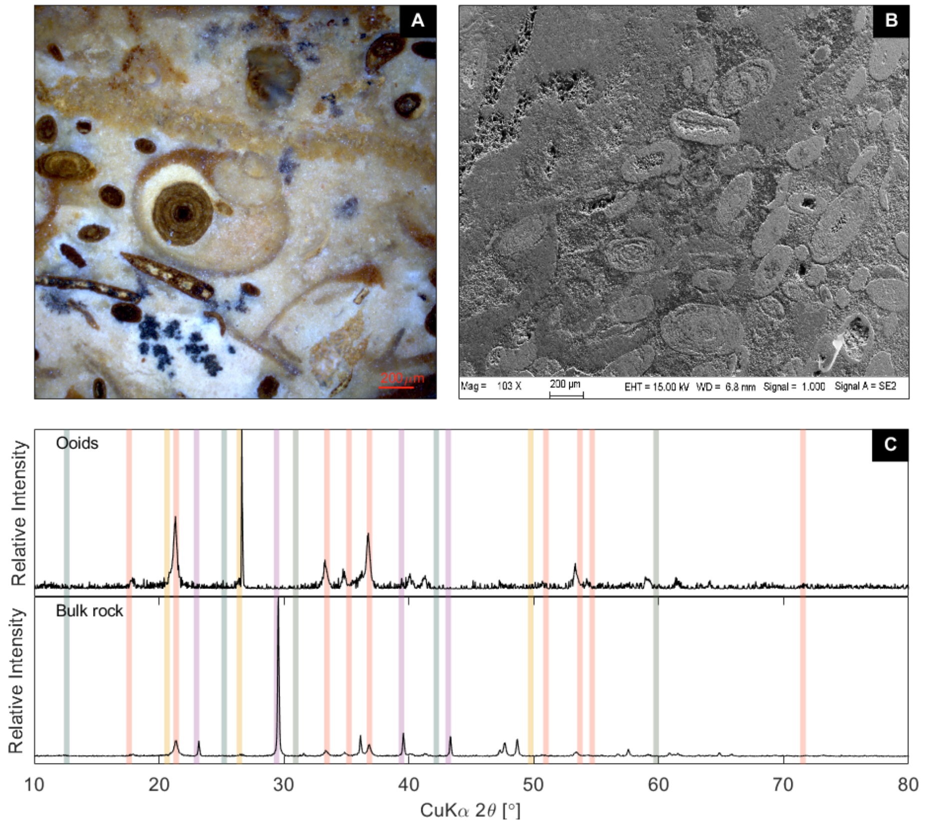

Fig. S51: Hidra Fm, Israel petrography (sample IO-OM 36). As for Fig. S47 but showing ooids from a different sample from the same formation. Vertical bars in (**C**) are the same as described in Fig. S25.

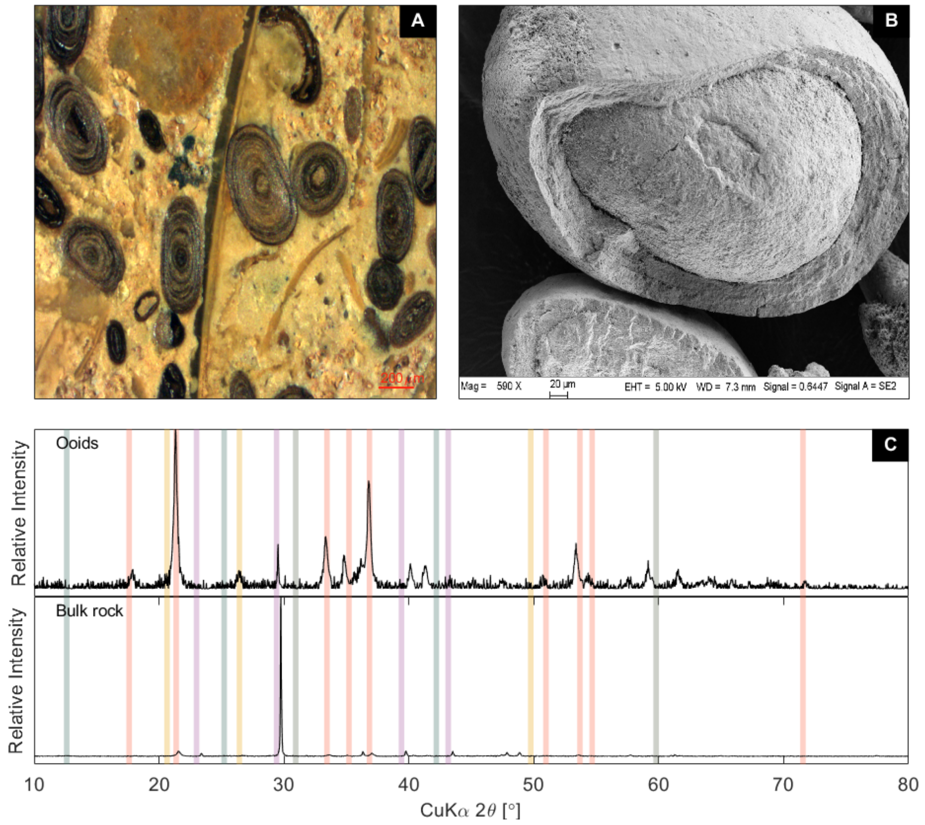

Fig. S52: Hidra Fm, Israel petrography (sample IO-OM 37). As for Fig. S47 but showing ooids from a different sample from the same formation. Vertical bars in (**C**) are the same as described in Fig. S25.

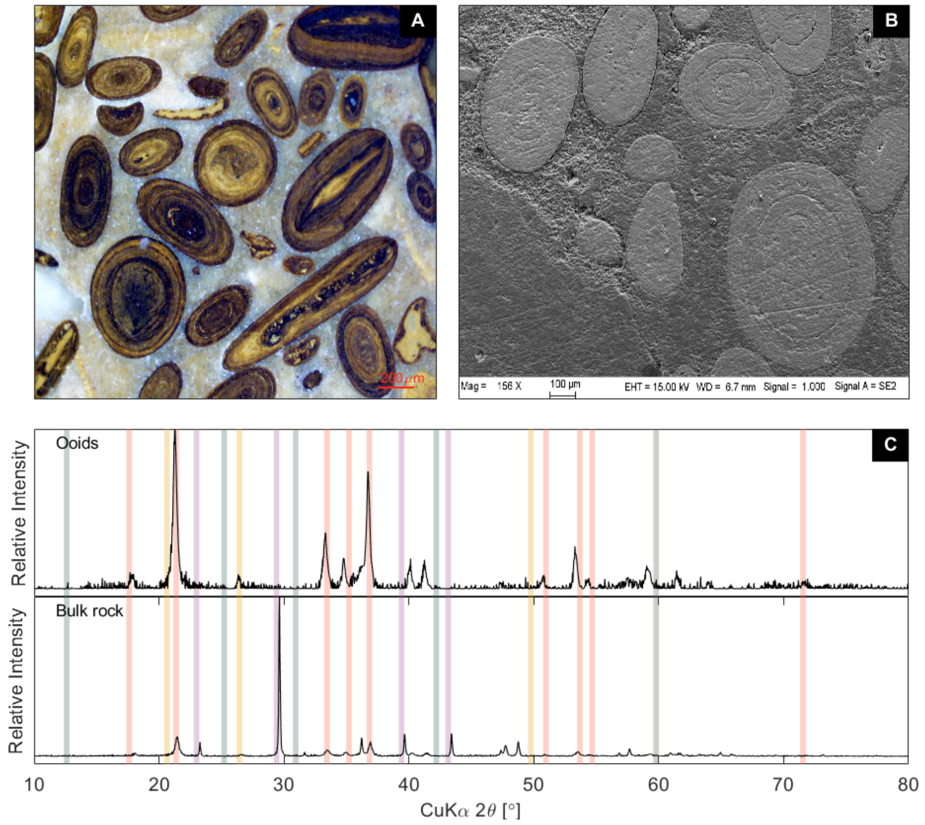

Fig. S53: Hidra Fm, Israel petrography (sample IO-OM 43). As for Fig. S47 but showing ooids from a different sample from the same formation. Vertical bars in (**C**) are the same as described in Fig. S25.

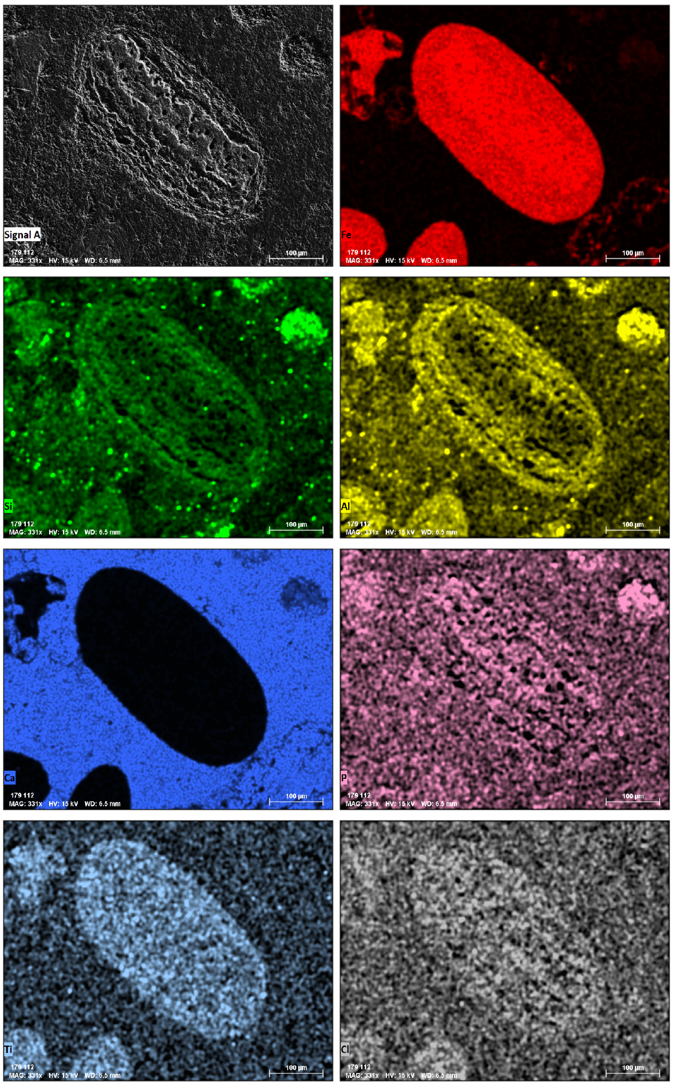

Fig. S54: Hidra Fm, Israel SEM-EDS elemental maps (sample IO-OM 34, subsample 1). Element designations include Ca (blue), Fe (red), Si (green), P (pink), Ti (teal), Cl (gray), and Al (yellow). Distinct features include Fe-rich ooids embedded in a Ca-rich carbonate cement as well as the presence of Al and Si in ooid rims, indicating traces of silicates. All elements that yielded a signal above the noise threshold are shown.

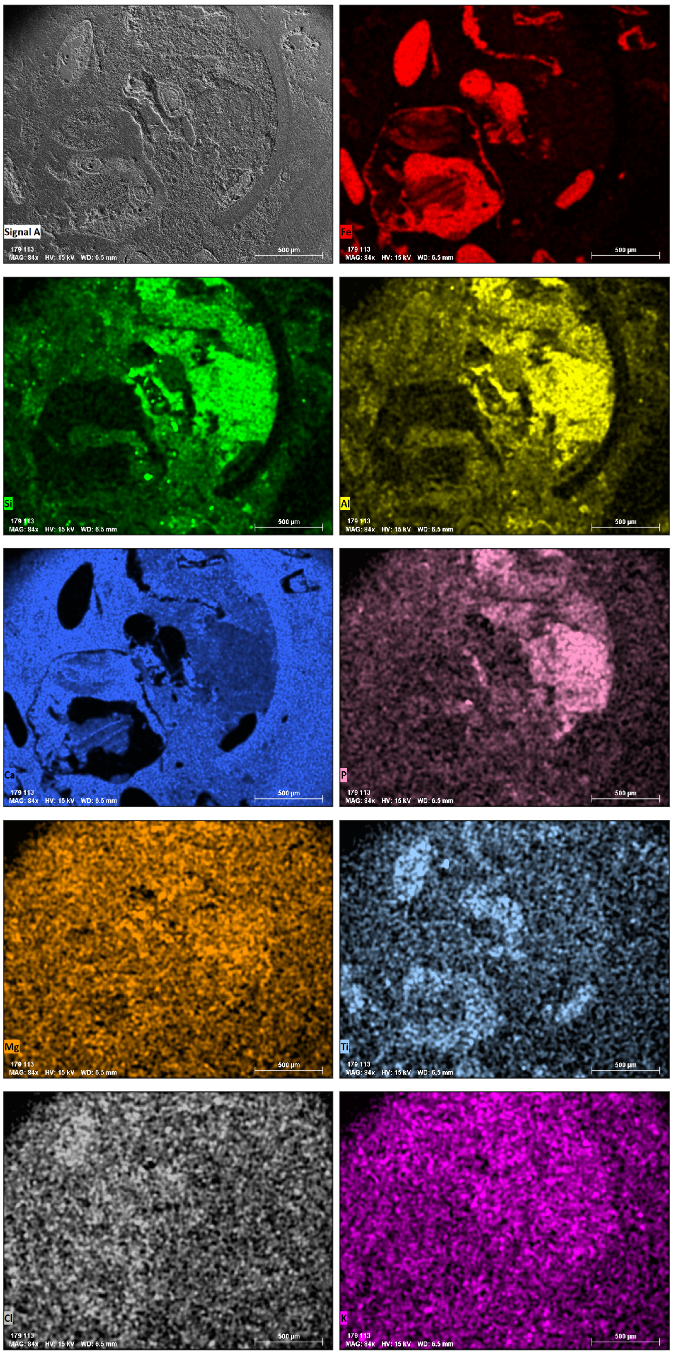

Fig. S55: Hidra Fm, Israel SEM-EDS elemental maps (sample IO-OM 34, subsample 2). As for Fig. S54 but showing ooids from a different subsample of the same sample and including element designations for K (purple) and Mg (orange).

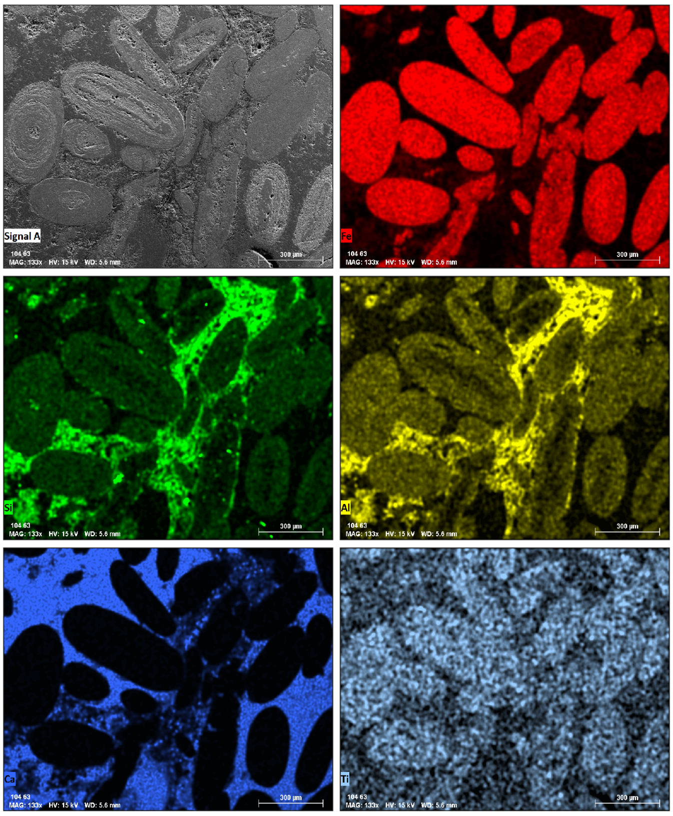

Fig. S56: Hidra Fm, Israel SEM-EDS elemental maps (sample IO-OM 35, subsample 1). As for Fig. S54 but showing ooids from a different sample from the same formation and omitting element designations for Cl and P.

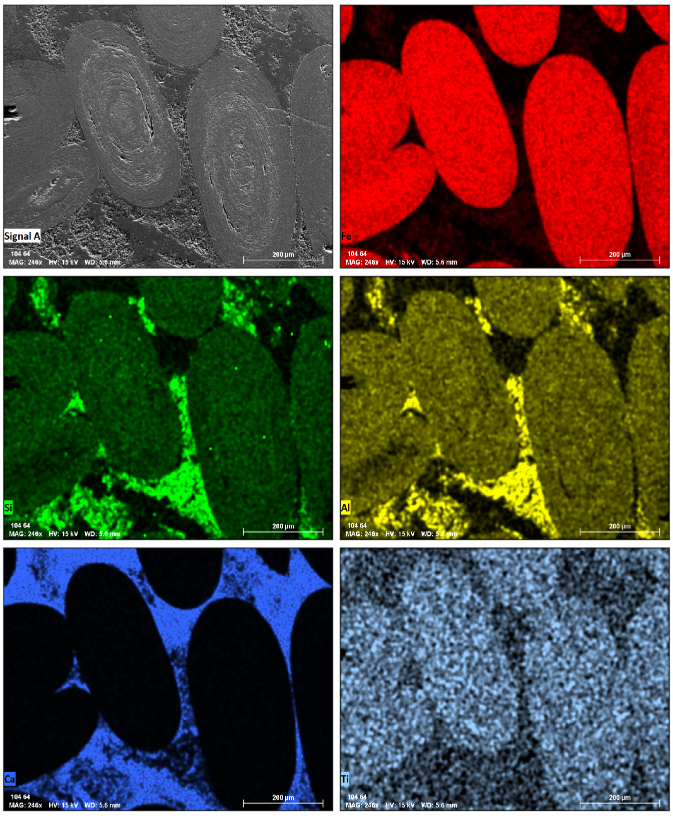

Fig. S57: Hidra Fm, Israel SEM-EDS elemental maps (sample IO-OM 35, subsample 2). As for Fig. S54 but showing ooids from a different sample from the same formation (different subsample from the same sample as Fig. S56) and omitting element designations for Cl and P.

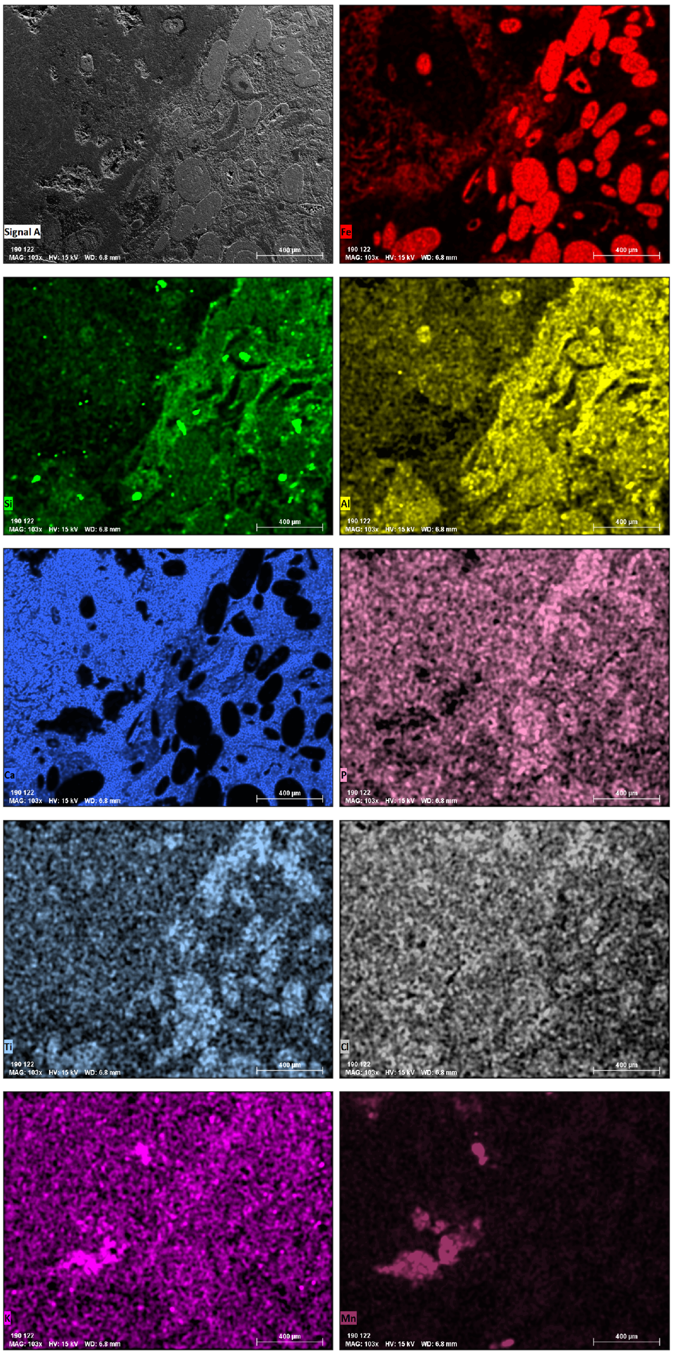

Fig. S58: Hidra Fm, Israel SEM-EDS elemental maps (sample IO-OM 36). As for Fig. S54 but showing ooids from a different sample from the same formation and including element designations for K (purple) and Mn (fuchsia). Ellipsoidal ooids composed predominantly of goethite show a distinct correlation with elements Ti, P, and Cl. These ooids are encased within a matrix comprising Ca-rich carbonate and Si- and Al-rich silicate cements. Additionally, occasional zones displaying a correlation between Mn and K suggest the presence of diagenetic Mn-oxides.

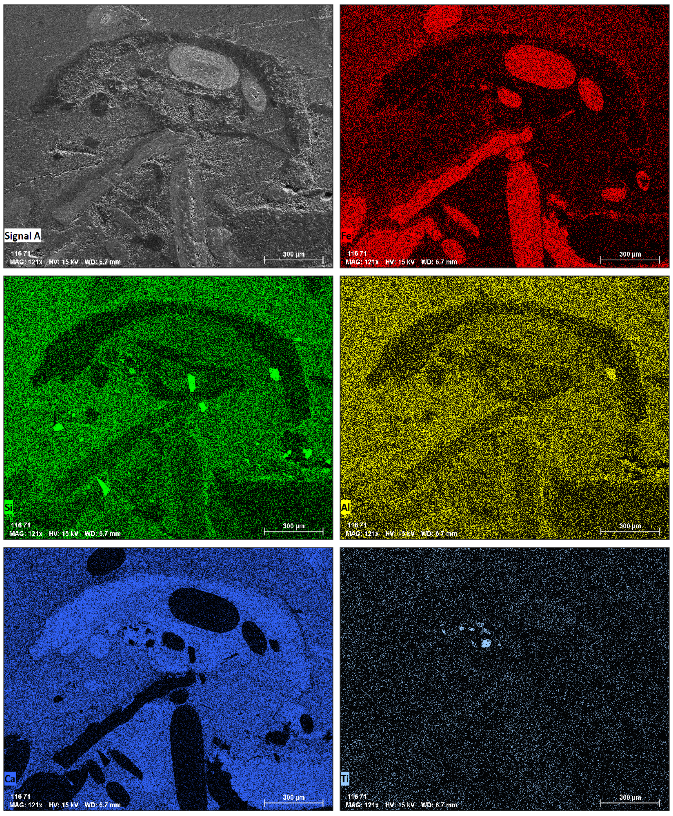

Fig. S59: Hidra Fm, Israel SEM-EDS elemental maps (sample IO-OM 37). As for Fig. S54 but showing ooids from a different sample from the same formation and omitting element designations for Cl and P. Ellipsoidal goethite ooids are embedded within a Ca-rich bivalve test.

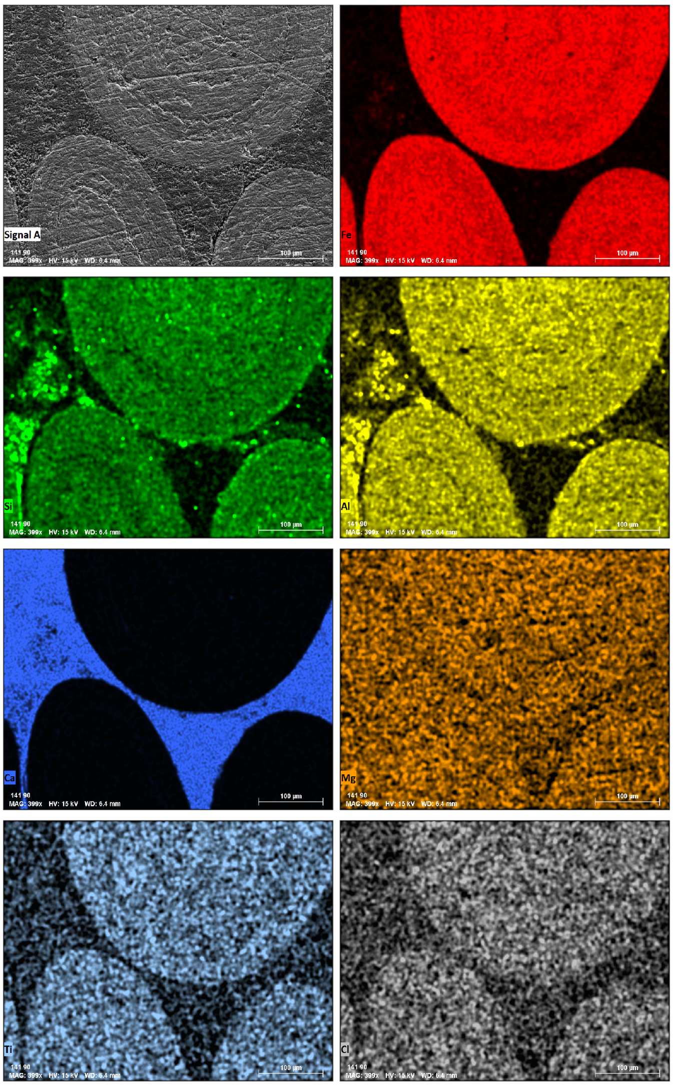

Fig. S60: Hidra Fm, Israel SEM-EDS elemental maps (sample IO-OM 43). As for Fig. S54 but showing ooids from a different sample from the same formation and including element designations for Mg (orange) and omitting element designations for P. Ellipsoidal goethite ooids are embedded within a Ca-rich cement. Correlation of Fe-rich ooids with Al, Ti, Cl and Si suggests the presence of silicates within the ooids.

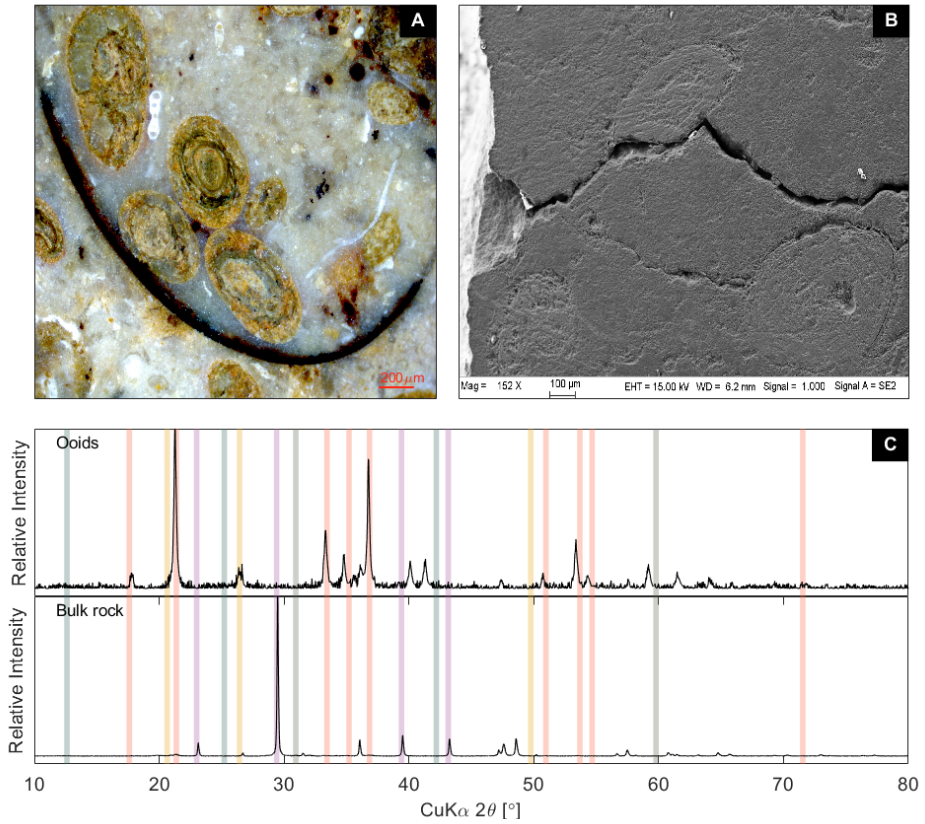

Fig. S61: Arroyofrío Bed, Chelva and Yatova Fms, Spain petrography (sample IO-OM 49). (**A**) Optical and (**B**) electron microscopy images and (**C**) XRD diffractogram of bulk rock and individual ooids. Vertical bars in (**C**) are the same as described in Fig. S25. The images display matrix-supported spherical goethite ooids in a calcite matrix located in a bivalve test.

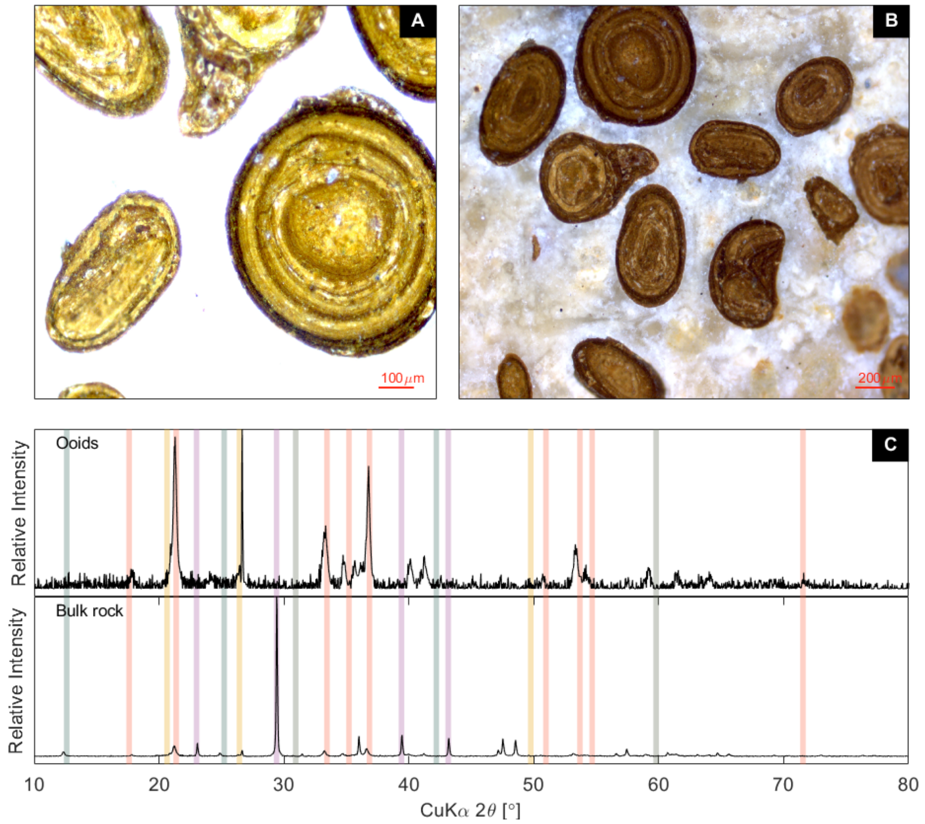

Fig. S62: Arroyofrío Bed, Chelva and Yatova Fms, Spain petrography (sample IO-OM 50). As for Fig. S61 but showing ooids from a different sample from the same formation. Vertical bars in (**C**) are the same as described in Fig. S25. SEM images were attempted for this sample but did not yield additional information due to porosity and epoxy resin artifacts.

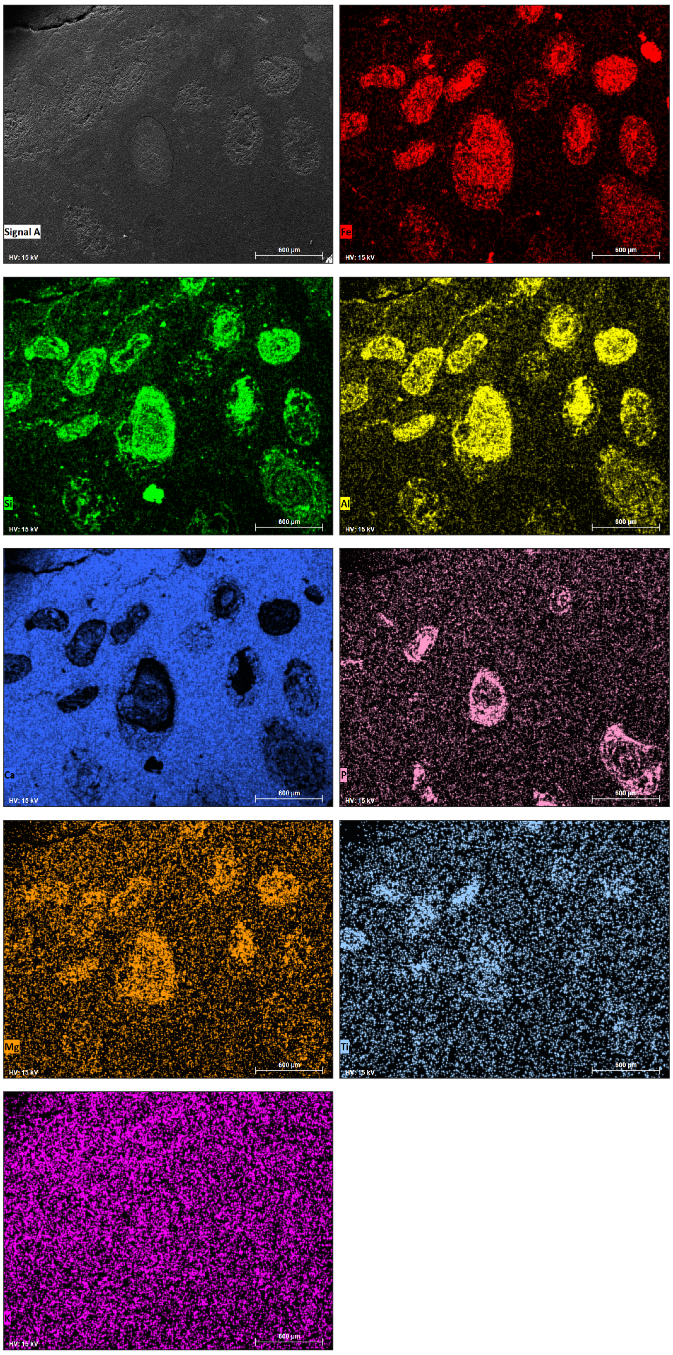

Fig. S63: Arroyofrío Bed, Chelva and Yatova Fms, Spain elemental maps (sample IO-OM 49). Element designations include Ca (blue), Fe (red), Si (green), P (pink), Mg (orange), K (purple), Ti (teal), and Al (yellow). Distinct features include Fe-rich ooids embedded in a carbonate cement as well as Si- and Al-rich detrital material; P-rich ooids are sparsely observed. All elements that yielded a signal above the noise threshold are shown.

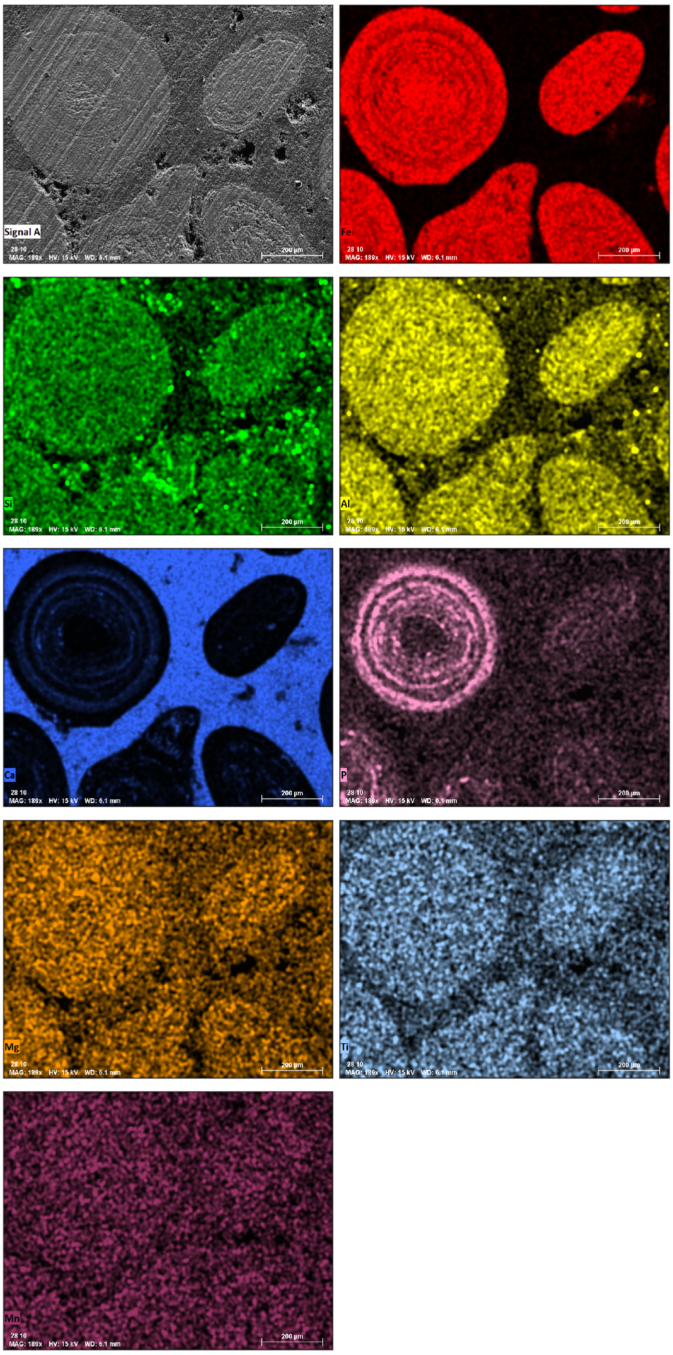

Fig. S64: Arroyofrío Bed, Chelva and Yatova Fms, Spain elemental maps (sample IO-OM 50). As for Fig. S63 but showing ooids from a different sample from the same formation and including element designations for Mn (fuchsia) and omitting element designations for K.

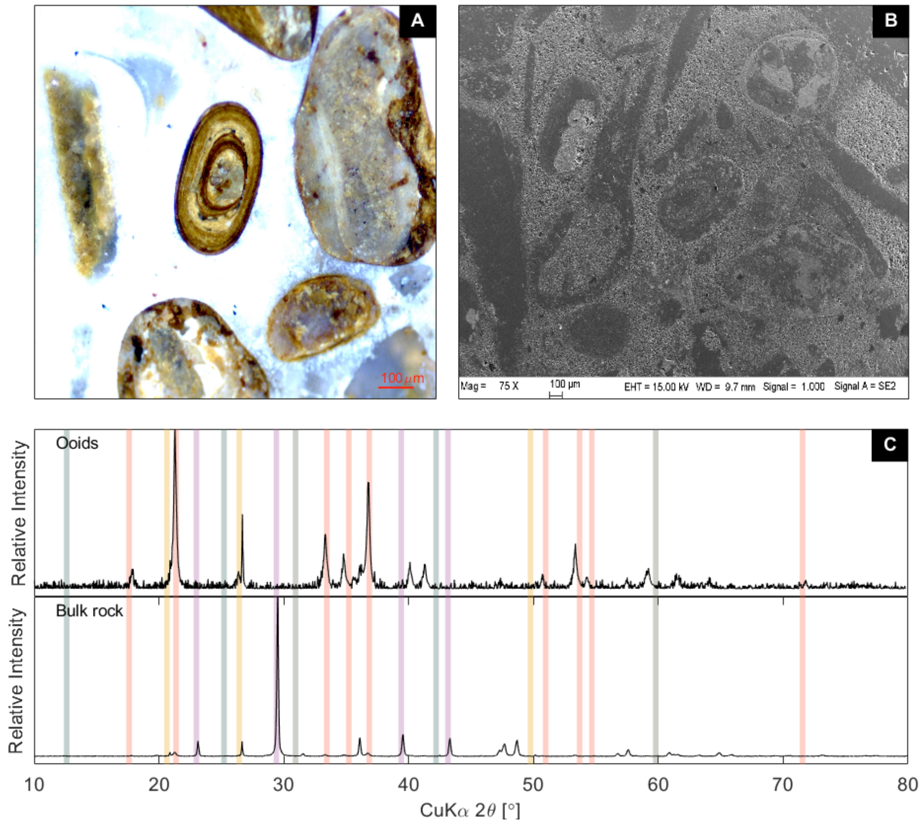

Fig. S65: Oolithe Ferrugineuse de Villers Fm, France petrography (sample IO-OM 51). (**A**) Optical and (**B**) electron microscopy images and (**C**) XRD diffractogram of bulk rock and individual ooids. Vertical bars in (**C**) are the same as described in Fig. S25. Images display small matrix-supported spherical goethite ooids as well as larger calcite ooids in a calcite matrix. The absence of multi-coatings on carbonate ooids, coupled with certain textural relationships, points towards a potential diagenetic replacement of iron oxides by carbonates.

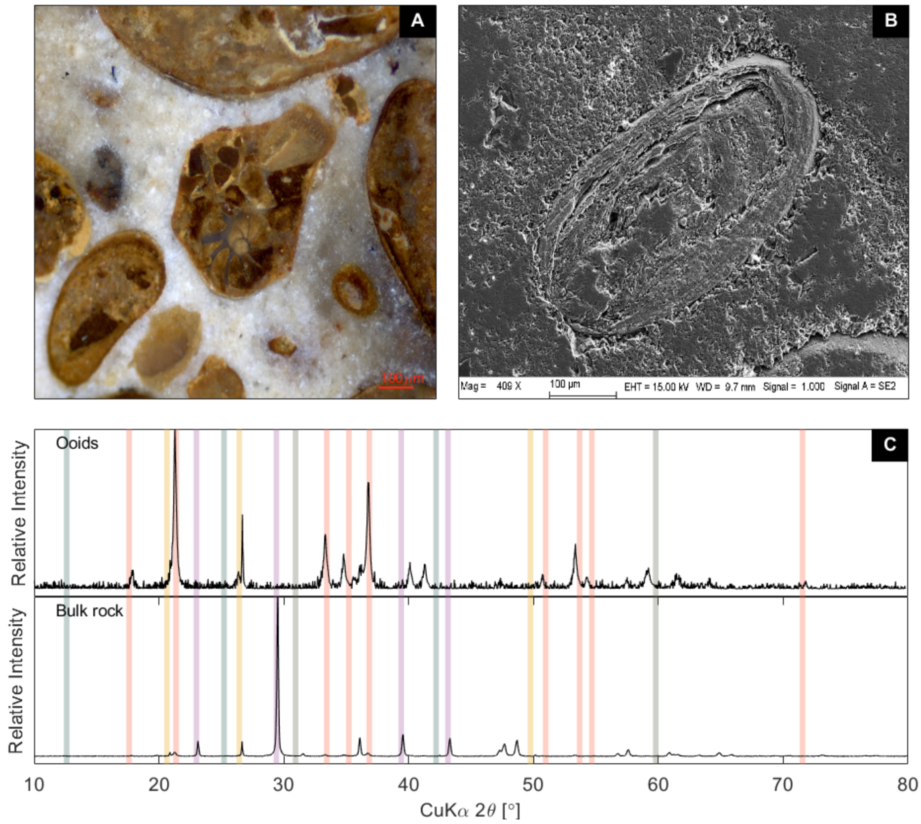

Fig. S66: Oolithe Ferrugineuse de Villers Fm, France petrography (sample IO-OM 52). As for Fig. S65 but showing ooids from a different sample from the same formation. Vertical bars in (**C**) are the same as described in Fig. S25. Note the presence of small ferruginous gastropod fossil in the central ooid.

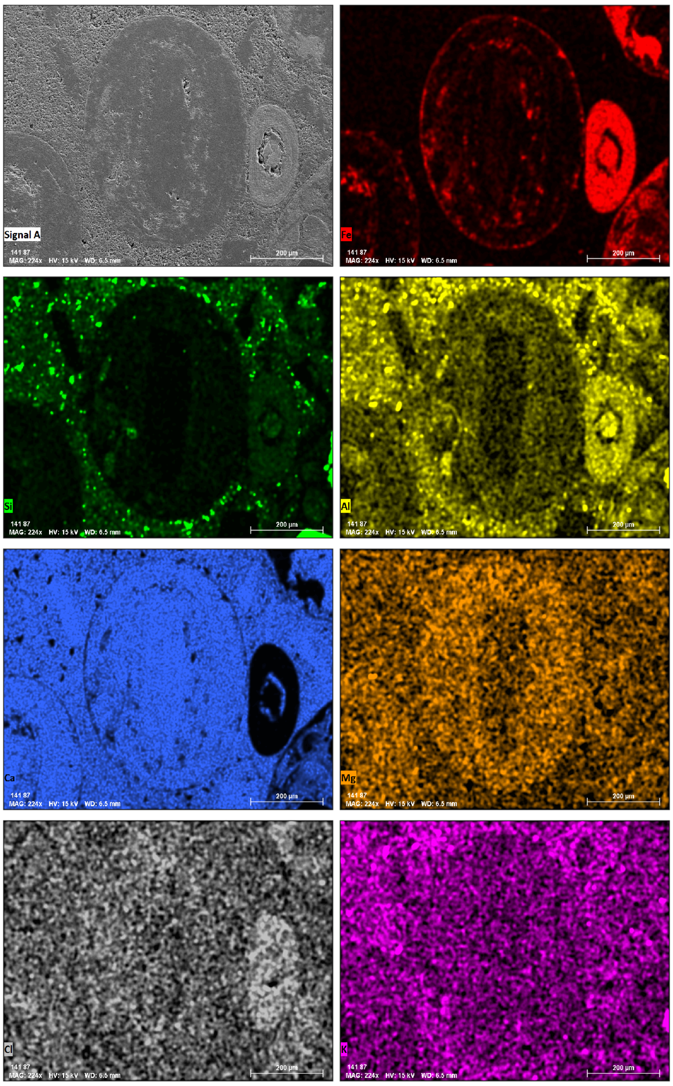

Fig. S67: Oolithe Ferrugineuse de Villers Fm, France elemental maps (sample IO-OM 51). Element designations include Ca (blue), Fe (red), Si (green), Mg (orange), K (purple), Cl (gray), and Al (yellow). Distinct features include fossil-rich carbonate—including corals and gastropods—with two distinct ooid types: (i) smaller, Fe-rich goethite ooids and (ii) larger, Ca-rich calcite ooids. The absence of multi-coatings on carbonate ooids, coupled with certain textural relationships, points towards a potential diagenetic replacement of iron oxides by carbonates. All elements that yielded a signal above the noise threshold are shown.

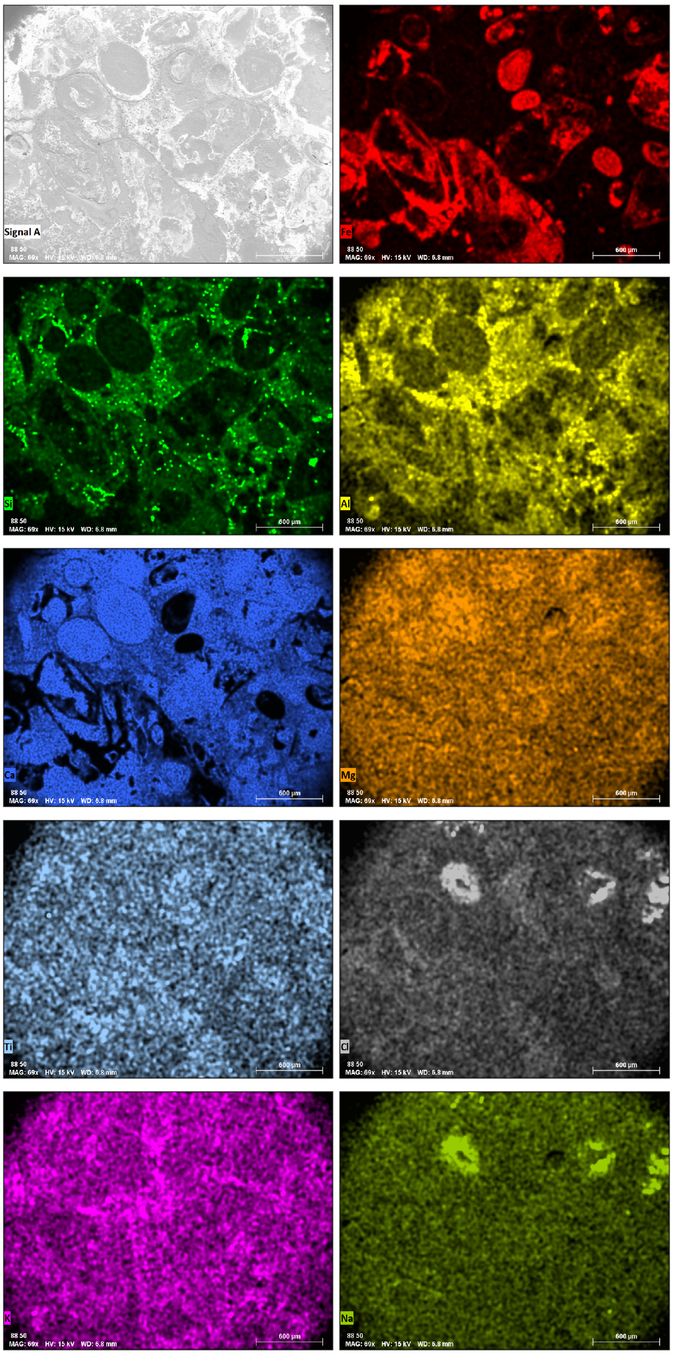

Fig. S68: Oolithe Ferrugineuse de Villers Fm, France elemental maps (sample IO-OM 52). As for Fig. S67 but showing ooids from a different sample from the same formation and including element designations for Ti (teal) and Na (bright green).

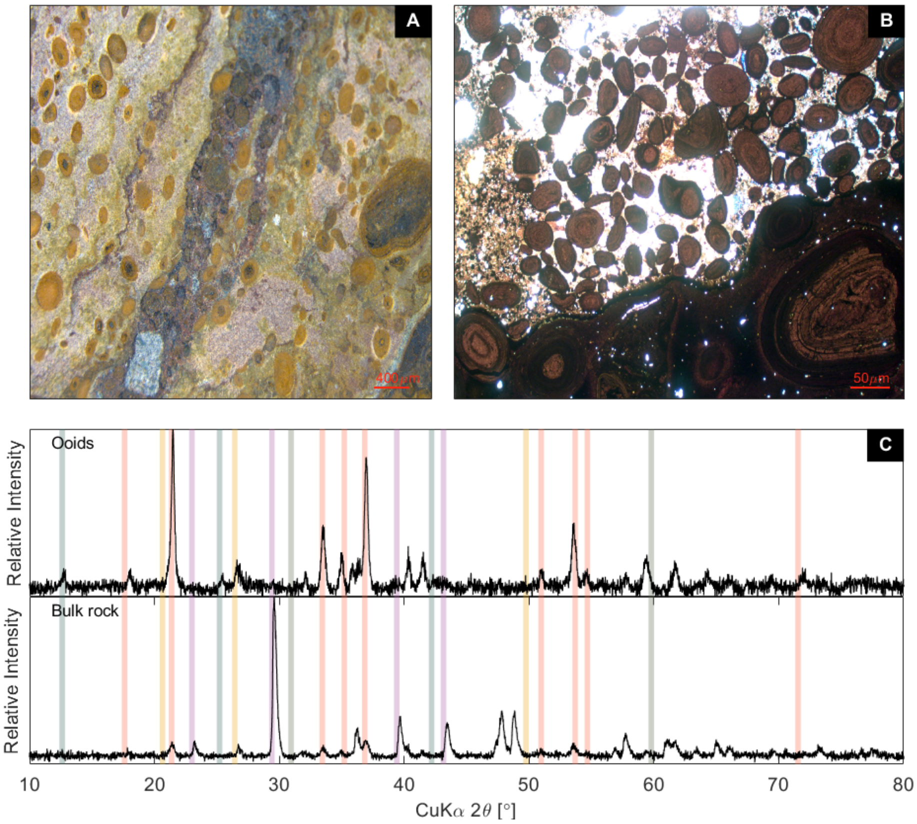

Fig. S69: Ifenthal Fm, Switzerland petrography (sample IO-OM 53). (**A-B**) Optical microscopy images (SEM images were attempted for this sample but did not yield additional information due to porosity and epoxy resin artifacts) and (**C**) XRD diffractogram of bulk rock and individual ooids. Vertical bars in (**C**) are the same as described in Fig. S25. Images display matrix-supported spherical goethite ooids in a siliceous dolomite matrix.

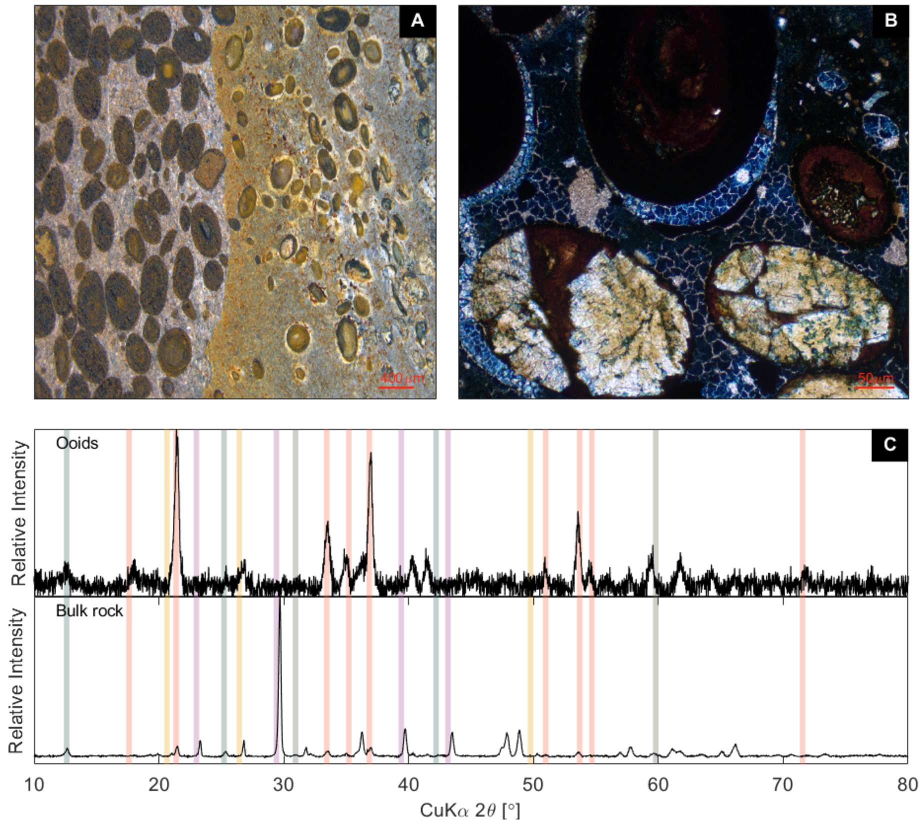

Fig. S70: Wutach Fm, Switzerland petrography (sample IO-OM 64). As for Fig. S69 but showing ooids from a different sample from the same set of formations. Vertical bars in (**C**) are the same as described in Fig. S25. Note that a minority of the goethite ooids were partly replaced by calcite.

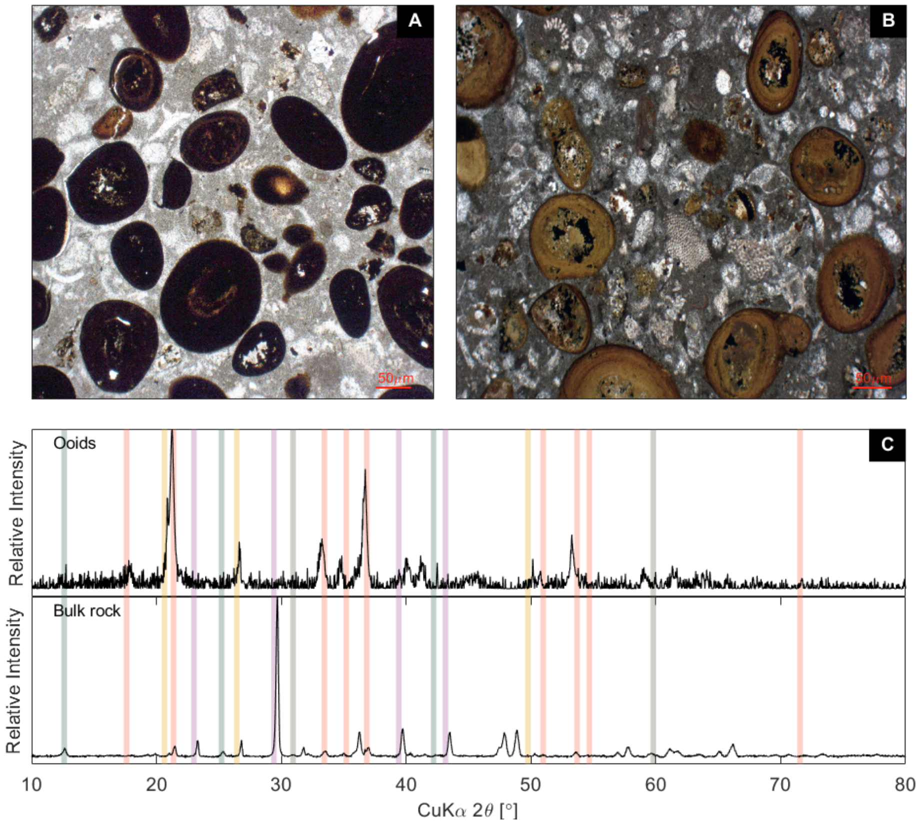

Fig. S71: "Humphriesioolith" Fm (informal), Switzerland petrography (sample IO-OM 55). As for Fig. S69 but showing ooids from a different sample from the same set of formations. Vertical bars in (**C**) are the same as described in Fig. S25. Note that minority of the goethite ooids were partly replaced by calcite.

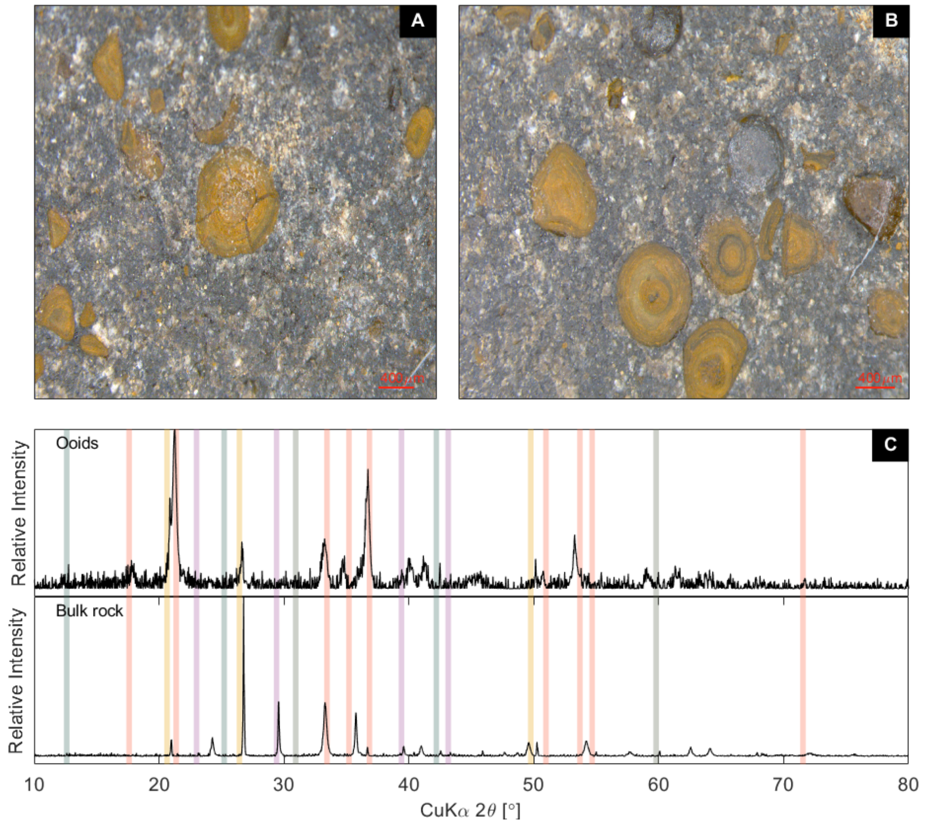

Fig. S72: “Parkinsoni-Württembergica Schichten” (informal), Switzerland petrography (sample IO-OM 56). As for Fig. S69 but showing ooids from a different sample from the same set of formations. Vertical bars in (**C**) are the same as described in Fig. S25.

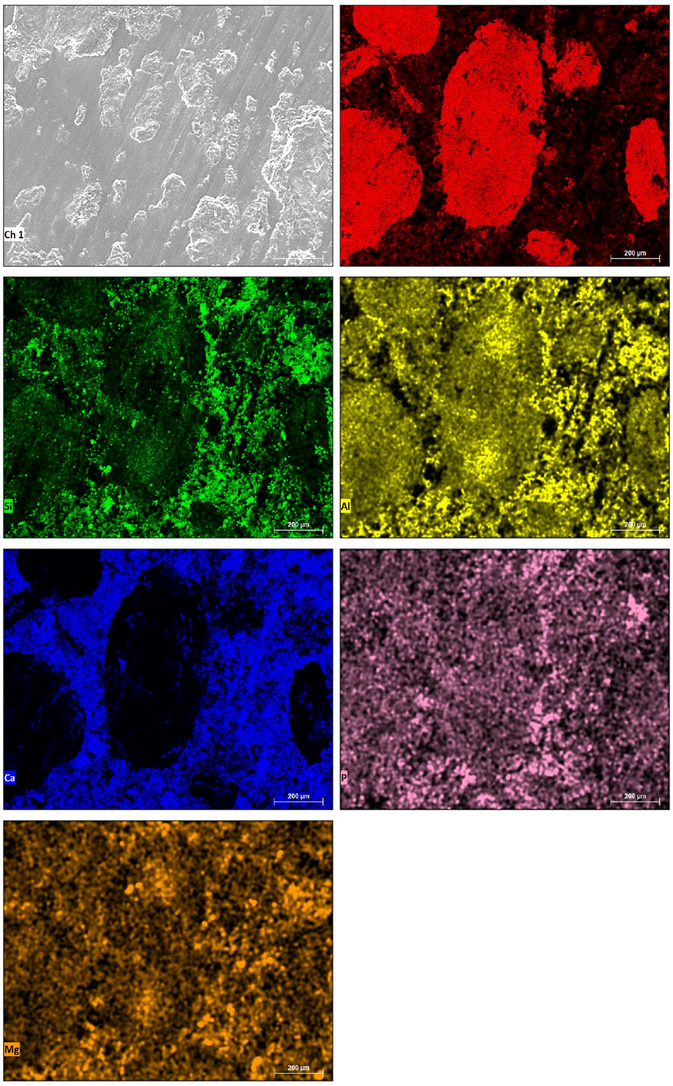

Fig. S73: Ifenthal Fm, Switzerland elemental maps (sample IO-OM 53). Element designations include Ca (blue), Fe (red), Si (green), Mg (orange), P (pink), and Al (yellow). Distinct features include Fe-rich goethite ooids embedded in a Ca- and Mg-rich carbonate cement as well as traces of Al and Si within ooids, indicating the presence of silicates. All elements that yielded a signal above the noise threshold are shown.

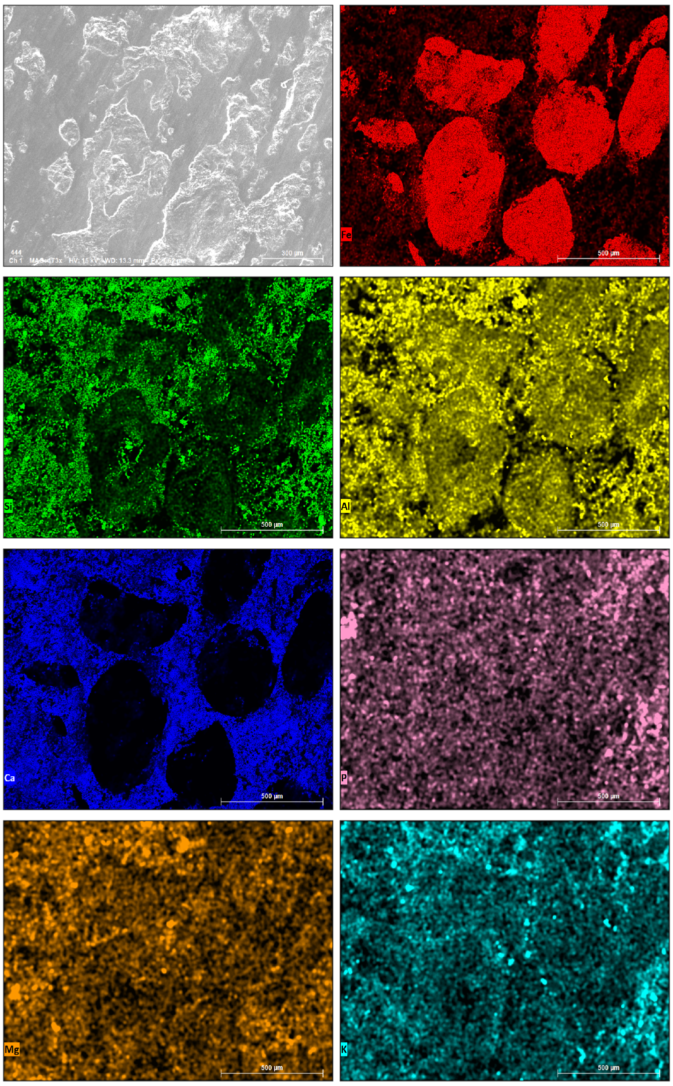

Fig. S74: Ifenthal Fm, Switzerland elemental maps (sample IO-OM 53). As for Fig. S73 but showing different ooids from the same sample and including elemental designations for K (teal).

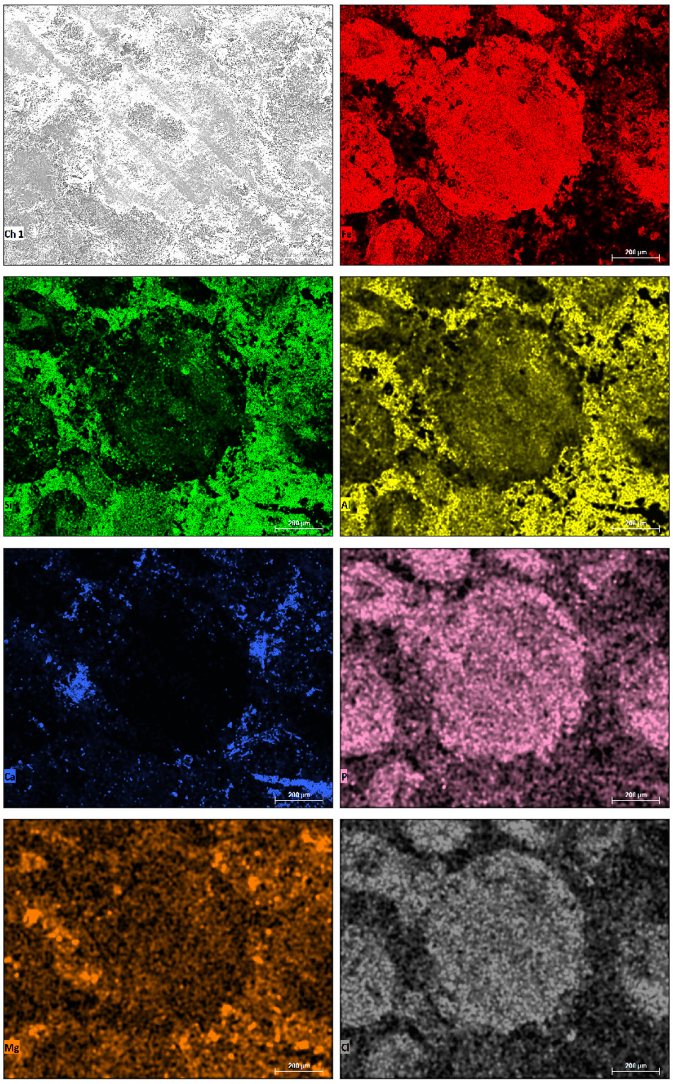

Fig. S75: Wutach Fm, Switzerland elemental maps (sample IO-OM 54). As for Fig. S73 but showing ooids from a different sample from the same set of formations and including elemental designations for Cl (gray).

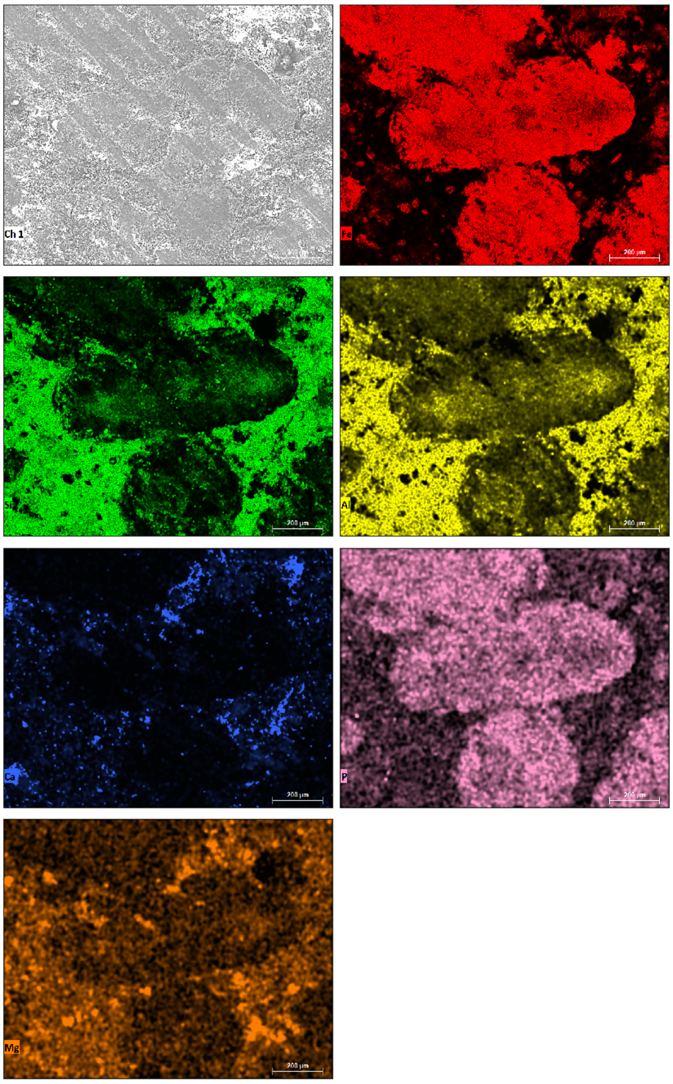

Fig. S76: Wutach Fm, Switzerland elemental maps (sample IO-OM 54). As for Fig. S75 but showing different ooids from the same sample and omitting elemental designations for Cl.

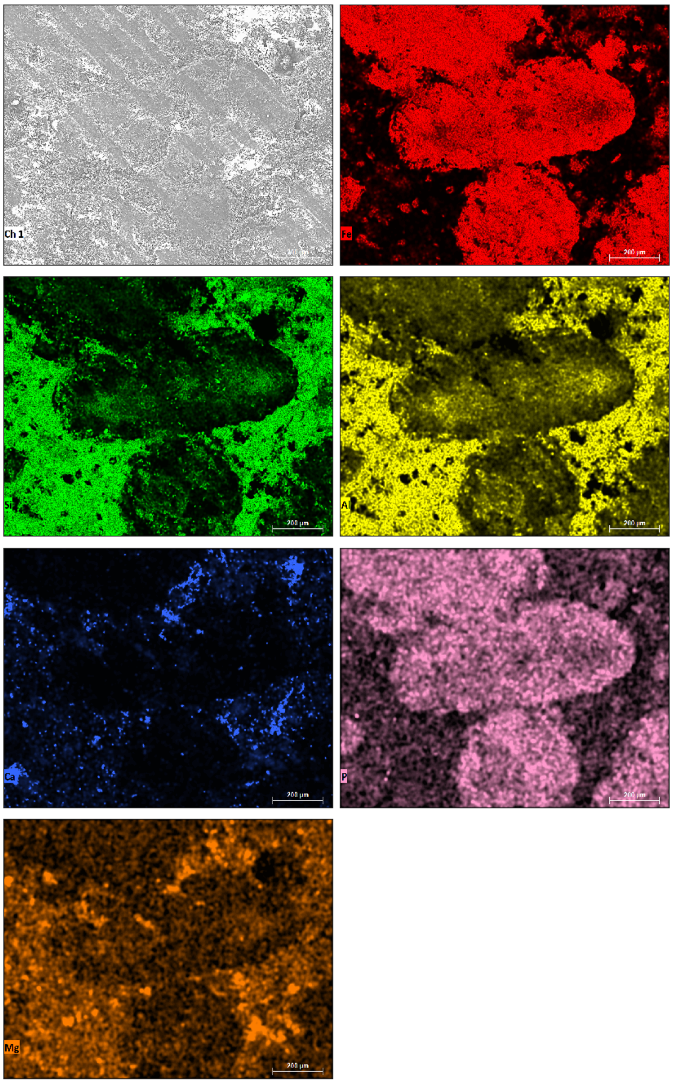

Fig. S77: “Humphriesioolith” Fm (informal), Switzerland elemental maps (sample IO-OM 55). As for Fig. S73 but showing ooids from a different sample from the same set of formations.

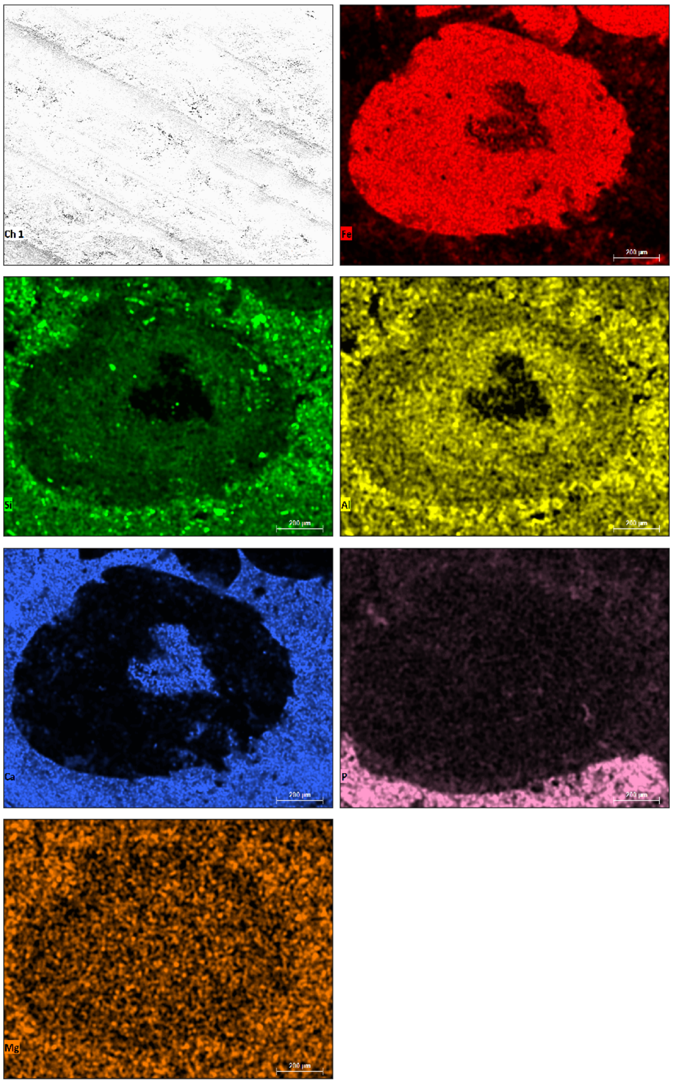

Fig. S78: "Humphriesioolith" Fm (informal), Switzerland elemental maps (sample IO-OM 55). As for Fig. S77 but showing different ooids from the same sample.

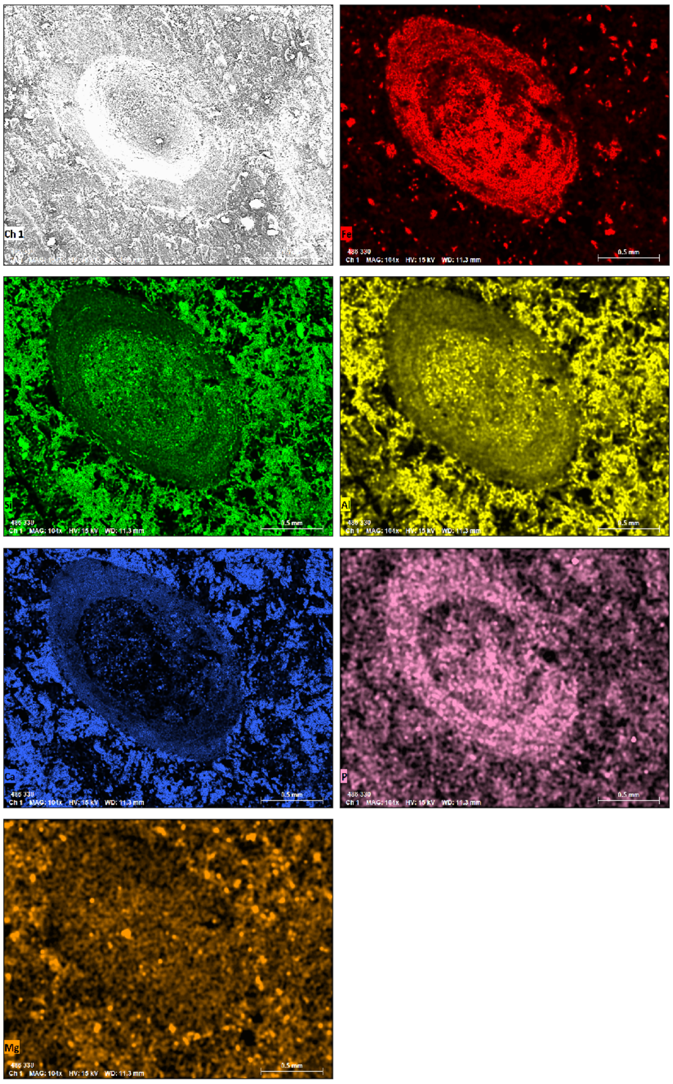

Fig. S79: "Parkinsoni-Württembergica Schichten" (informal), Switzerland elemental maps (sample IO-OM 56). As for Fig. S73 but showing ooids from a different sample from the same set of formations.

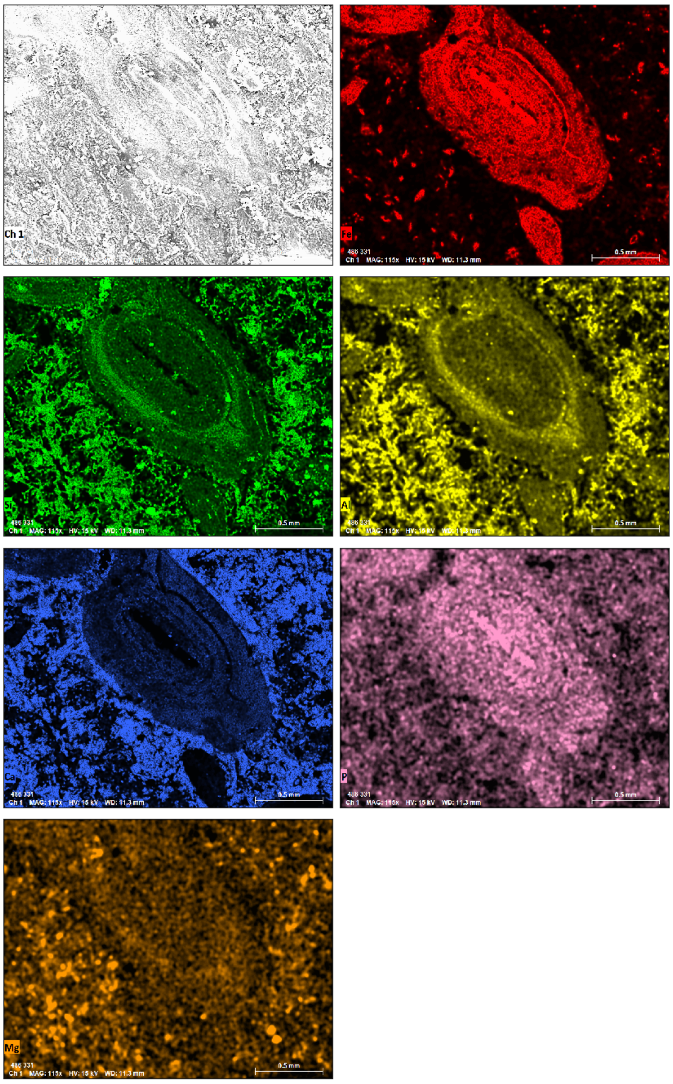

Fig. S80: "Parkinsoni-Württembergica Schichten" (informal), Switzerland elemental maps (sample IO-OM 56). As for Fig. S79 but showing different ooids from the same sample.

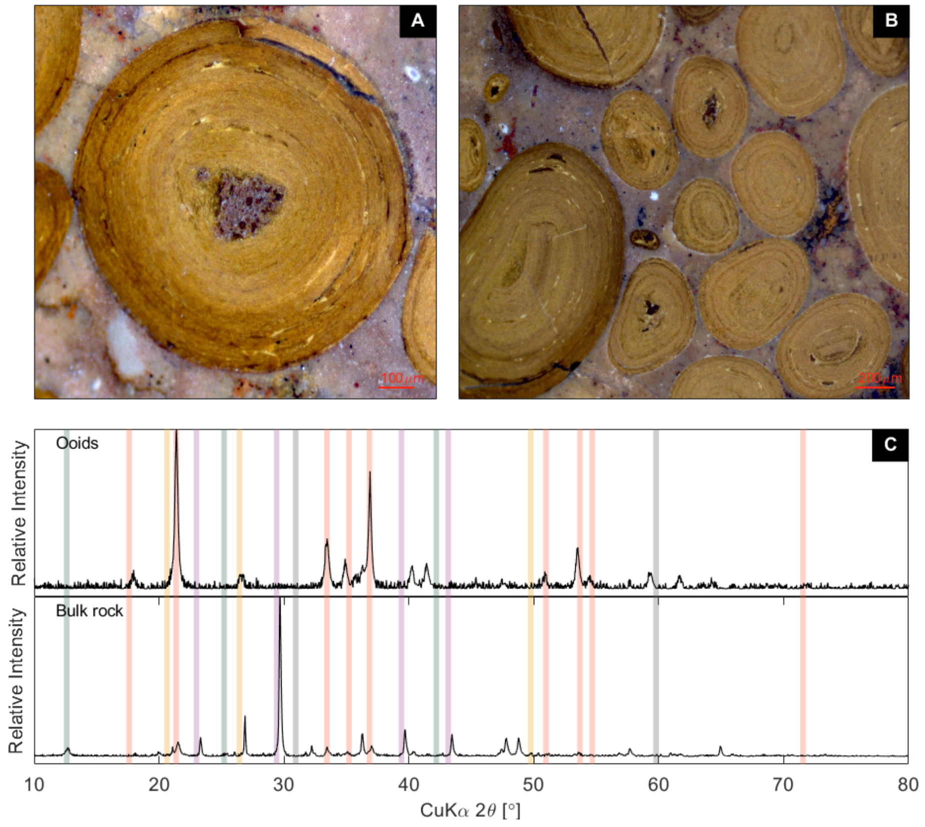

Fig. S81: Wedelsandstein Fm (the Blaukalk Member), Germany petrography (sample IO-OM 59). (**A-B**) Optical microscopy images (SEM images were attempted for this sample but did not yield additional information due to porosity and epoxy resin artifacts) and (**C**) XRD diffractogram of bulk rock and individual ooids. Vertical bars in (**C**) are the same as described in Fig. S25. Images display matrix-supported spherical goethite ooids in a dolomite matrix.

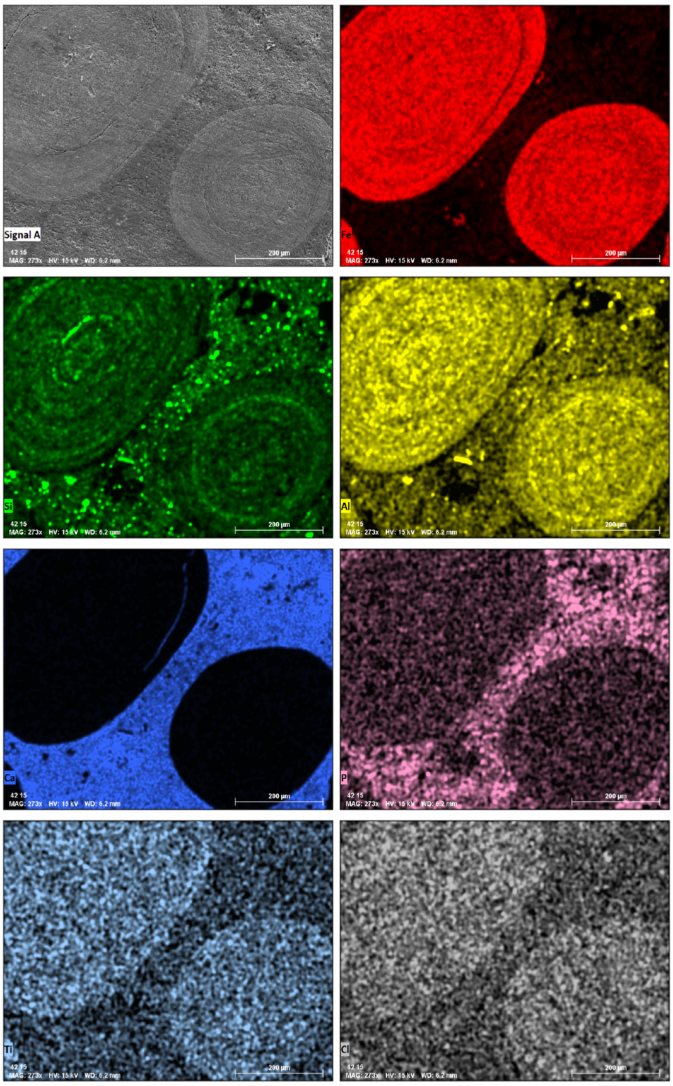

Fig. S82: Wedelsandstein Fm (the Blaukalk Member), Germany elemental maps (sample IO-OM 59). Element designations include Ca (blue), Fe (red), Si (green), Ti (teal), Cl (gray), P (pink), and Al (yellow). Distinct features include Fe-rich goethite ooids embedded in a Ca- and P-rich carbonate cement.

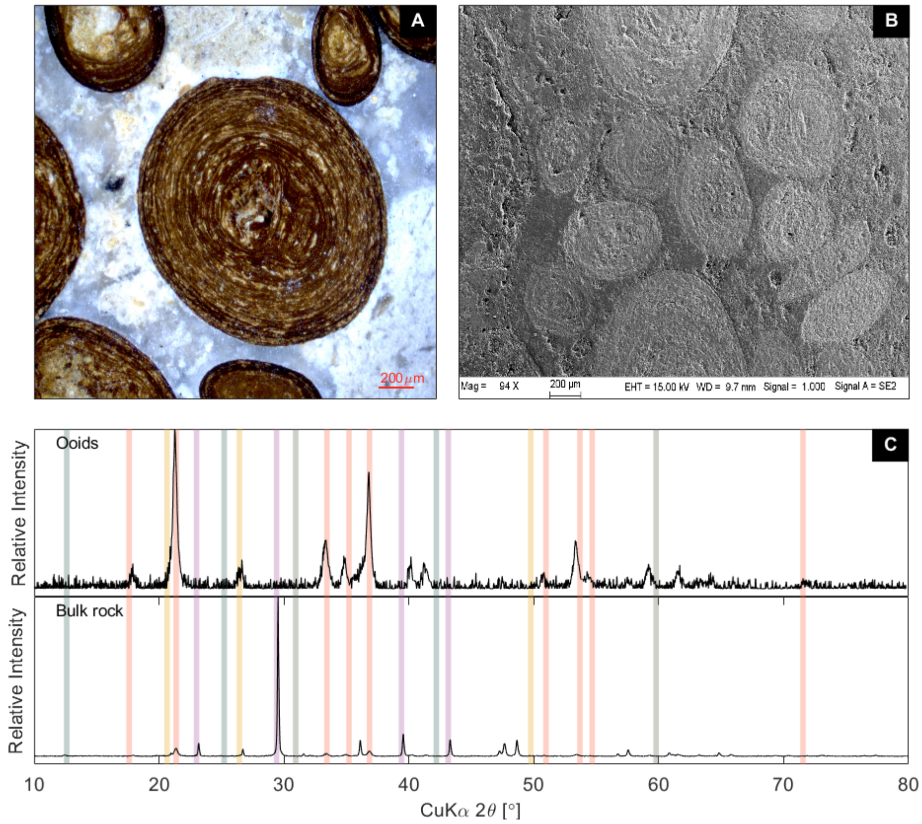

Fig. S83: Oolithe Ferrugineuse de Bayeux Fm, France petrography (sample IO-OM 61, subsample 1). (**A**) Optical and (**B**) electron microscopy images and (**C**) XRD diffractogram of bulk rock and individual ooids. Vertical bars in (**C**) are the same as described in Fig. S25. Images display matrix-supported spherical goethite ooids in a dolomite matrix.

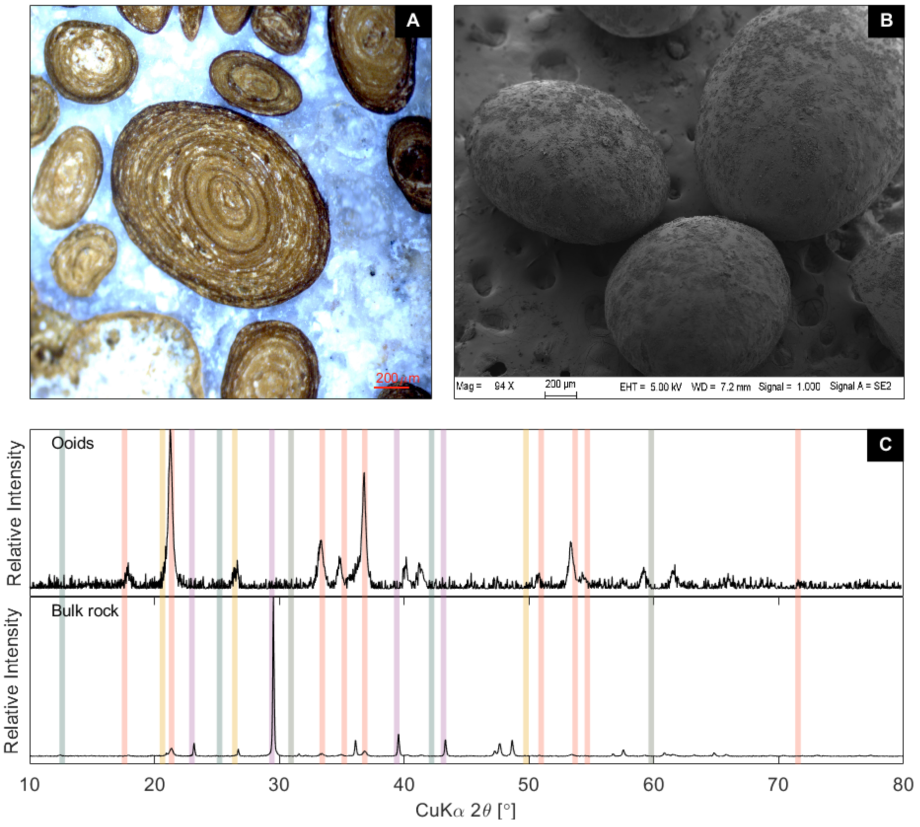

Fig. S84: Oolithe Ferrugineuse de Bayeux Fm, France petrography (sample IO-OM 61, subsample 2). As for Fig. S83 but showing ooids from a different subsample of the same sample. Vertical bars in (**C**) are the same as described in Fig. S25.

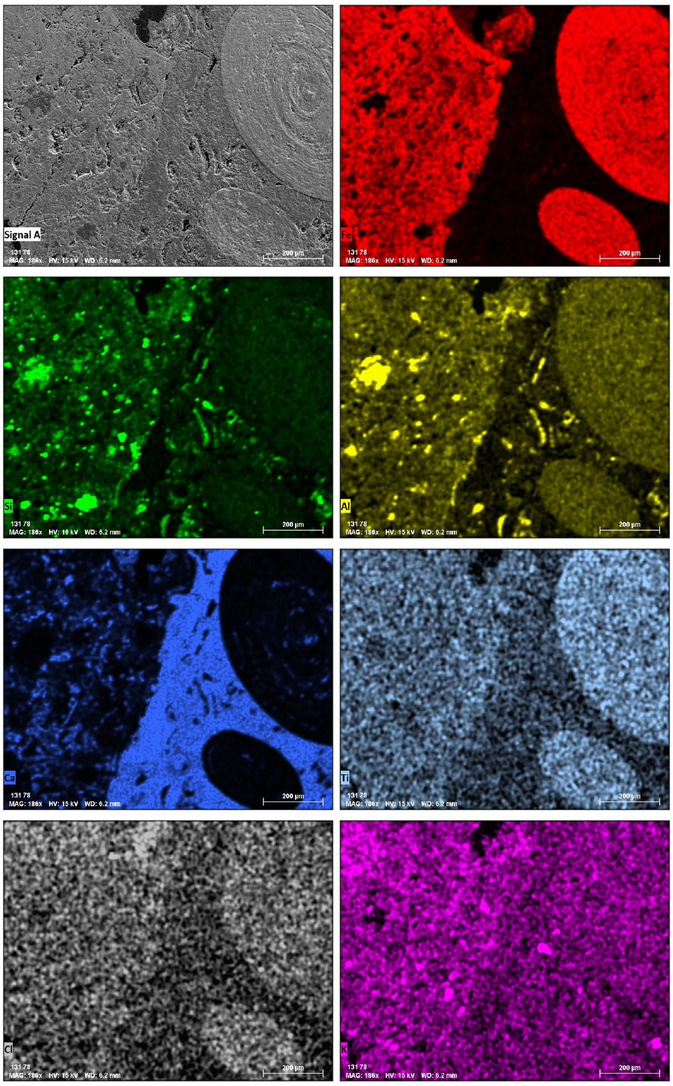

Fig. S85: Oolithe Ferrugineuse de Bayeux Fm, France elemental maps (sample IO-OM 61, subsample 1). Element designations include Ca (blue), Fe (red), Si (green), K (purple), Ti (teal), Cl (gray), and Al (yellow). Distinct features include Fe-rich goethite ooids embedded in a Ca- and Mg-rich carbonate cement. All elements that yielded a signal above the noise threshold are shown.

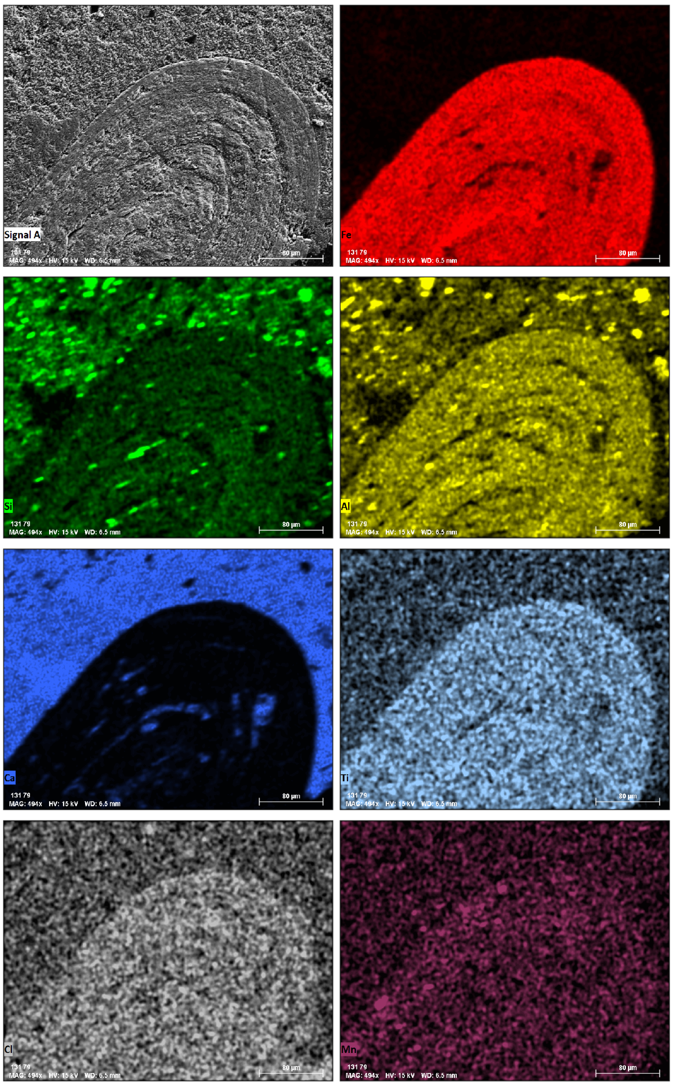

Fig. S86: Oolithe Ferrugineuse de Bayeux Fm, France elemental maps (sample IO-OM 61, subsample 2). As for Fig. S85 but showing different ooids from the same sample, omitting elemental designations for K and including elemental designations for Mn (fuchsia).

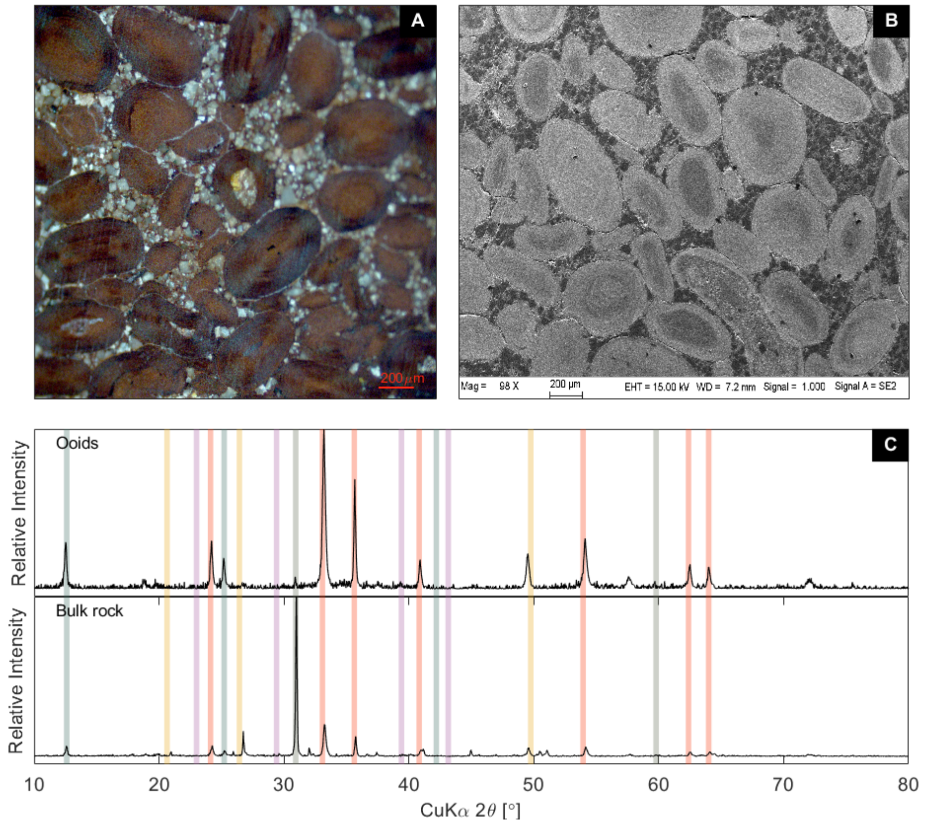

Fig. S87: Presles Fm, Belgium petrography (sample IO-OM 72). (**A**) Optical and (**B**) electron microscopy images and (**C**) XRD diffractogram of bulk rock and individual ooids. Vertical bars in (**C**) are the same as described in Fig. S25. Images display matrix-supported spherical hematite ooids in a dolomite matrix. Electron microscopy image additionally shows that ooid cores are less dense than ooid rims, potentially indicating early replacement of primary Fe-silicates by hematite.

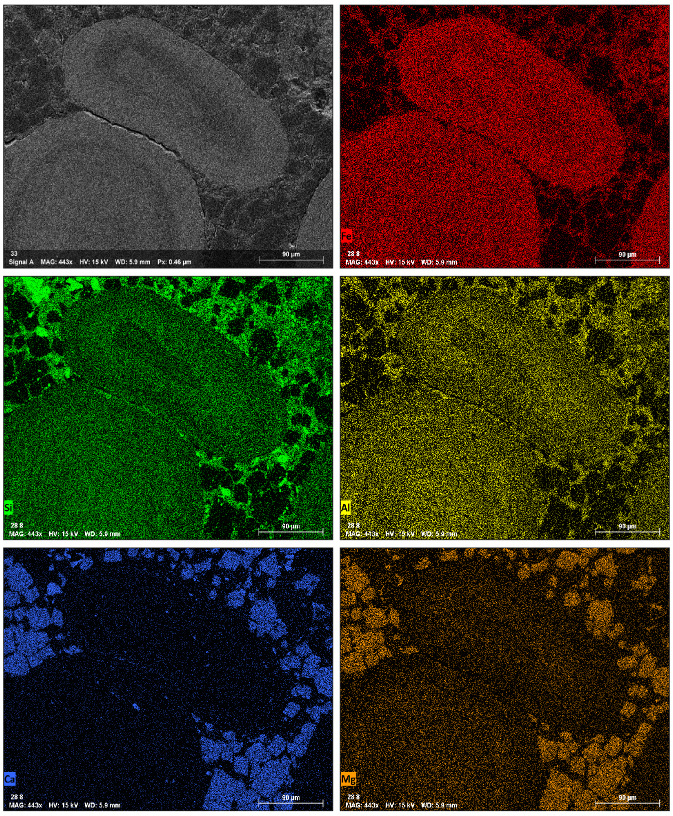

Fig. S88: Presles Fm, Belgium elemental maps (sample IO-OM 72). Element designations include Ca (blue), Fe (red), Si (green), Mg (orange), and Al (yellow). Distinct features include Fe-rich hematite ooids containing Si, Mg, and Al embedded in a Ca- and Mg-rich carbonate cement that is occasionally enriched in Al and Si. All elements that yielded a signal above the noise threshold are shown.

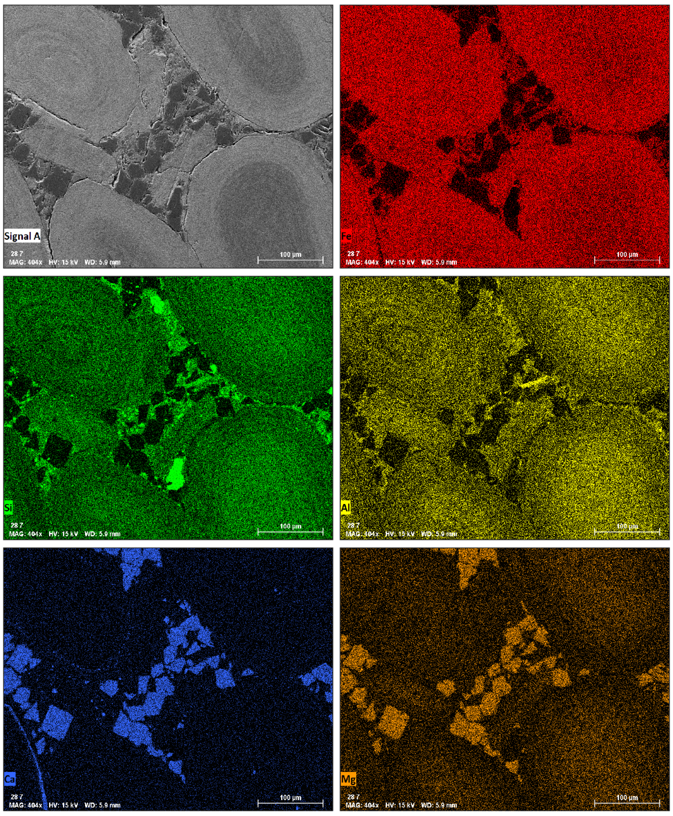

Fig. S89: Presles Fm, Belgium elemental maps (sample IO-OM 72). As for Fig. S88 but showing different ooids from the same sample.

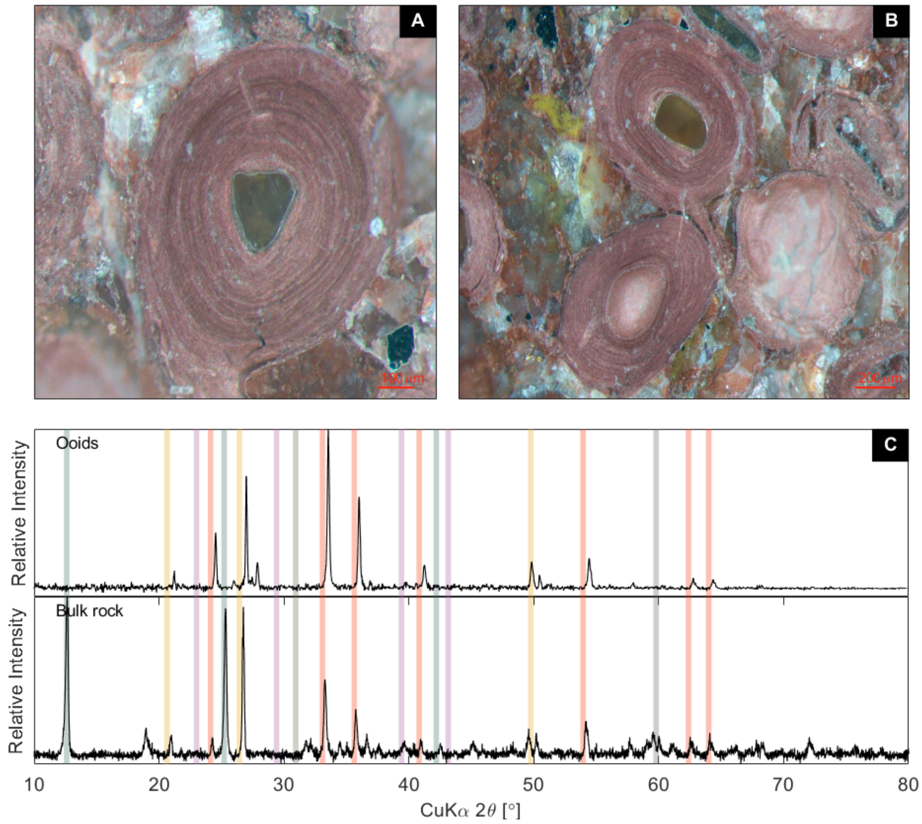

Fig. S90: Westmoreland and Kirkland Fms, Clinton Group, USA petrography (sample IO-OM 76). (**A**-**B**) Optical microscopy images (SEM images were attempted for this sample but did not yield additional information due to porosity and epoxy resin artifacts) and (**C**) XRD diffractogram of bulk rock and individual ooids. Vertical bars in (**C**) are the same as described in Fig. S25. Images display matrix-supported spherical hematite ooids in a silicious dolomite matrix.

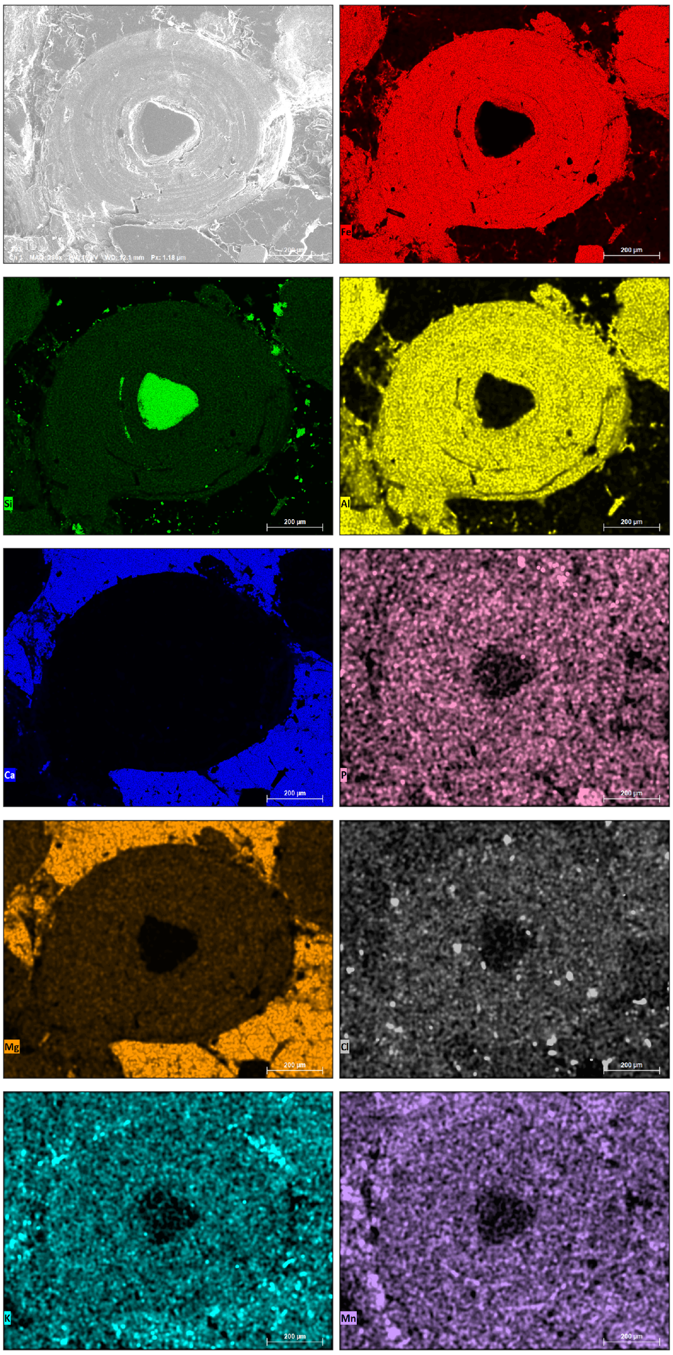

Fig. S91: Westmoreland and Kirkland Fms, Clinton Group, USA elemental maps (sample IO-OM 76). Element designations include Ca (blue), Fe (red), Si (green), Mg (orange), P (pink), Mn (fuchsia), K (teal), Cl (gray), and Al (yellow). Distinct features include Ca- and Mg-rich carbonate cement containing hematite ooids rich in Si, Mg and Al, suggesting the presence of Fe-silicates such as chamosite. All elements that yielded a signal above the noise threshold are shown.

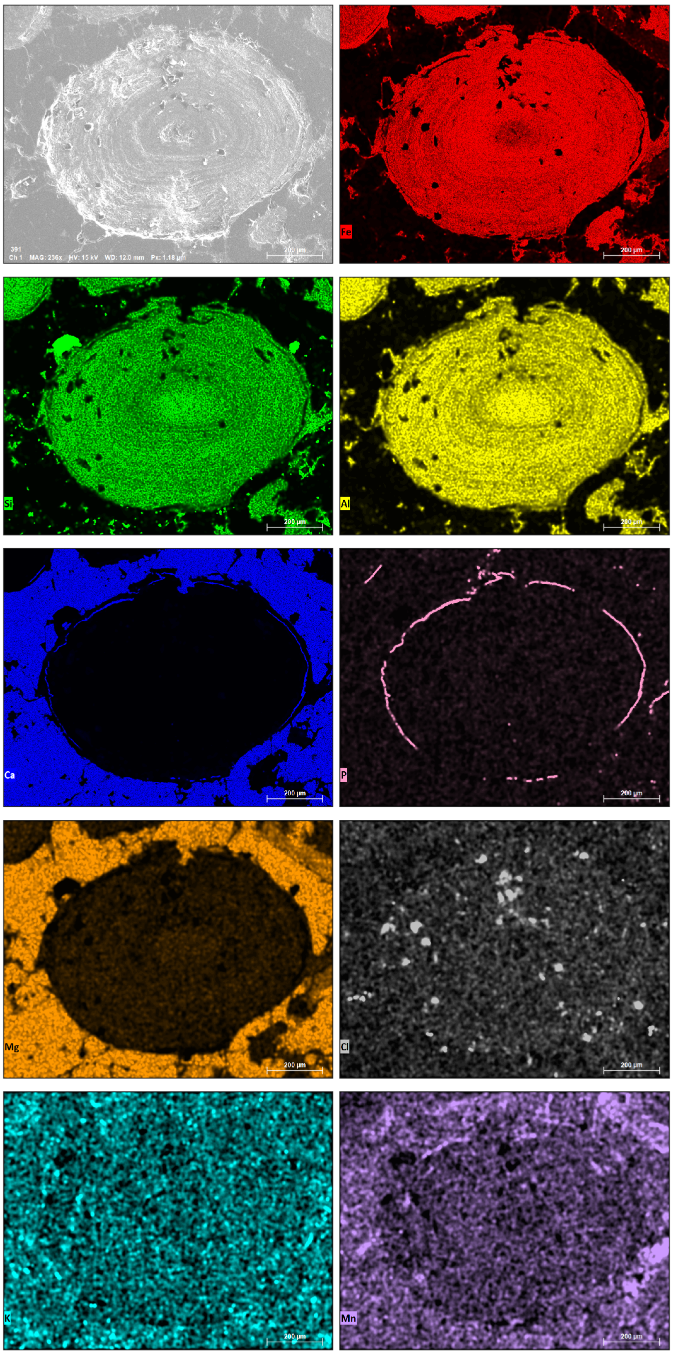

Fig. S92: Westmoreland and Kirkland Fms, Clinton Group, USA petrography (sample IO-OM 76). As for Fig. S91 but showing different ooids from the same sample.

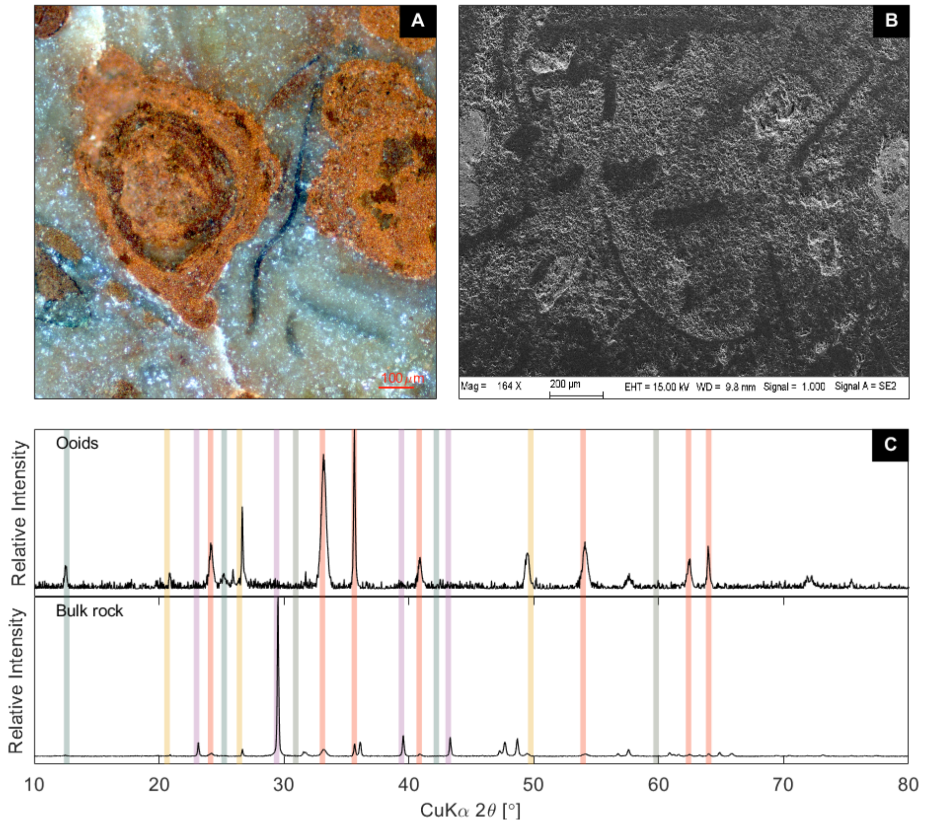

Fig. S93: Skovde Limestone, Gullhogen Fm, Sweden petrography (sample IO-OM 78). (**A**) Optical and (**B**) electron microscopy images and (**C**) XRD diffractogram of bulk rock and individual ooids. Vertical bars in (**C**) are the same as described in Fig. S25. Images display matrix-supported spherical hematite ooids in a silicious dolomite matrix with dispersed bivalve test fossils.

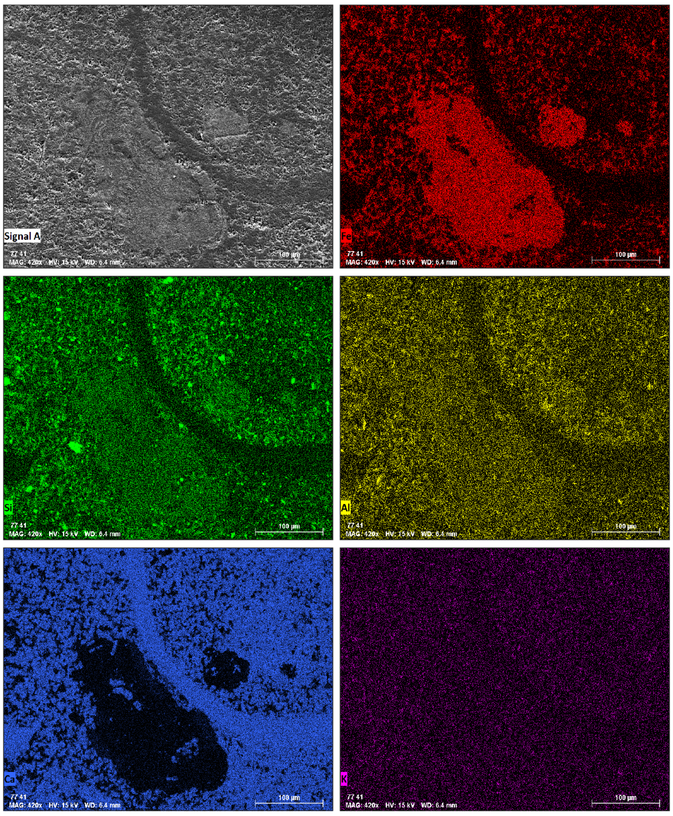

Fig. S94: Skovde Limestone, Gullhogen Fm, Sweden elemental maps (sample IO-OM 78). Element designations include Ca (blue), Fe (red), Si (green), K (purple), and Al (yellow). Distinct features include Ca-rich carbonate cement containing hematite ooids as well as the presence of bivalve tests. All elements that yielded a signal above the noise threshold are shown.

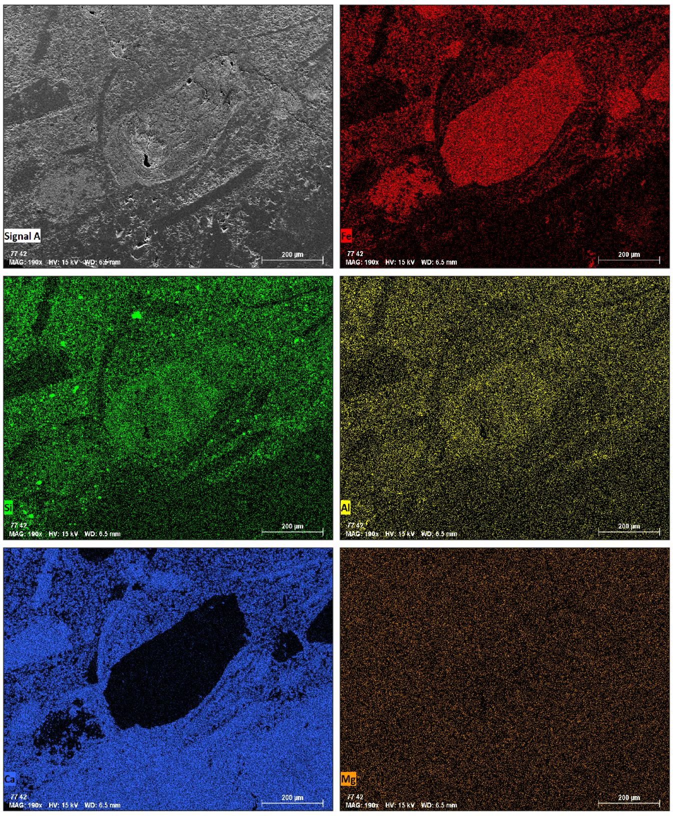

Fig. S95: Skovde Limestone, Gullhogen Fm, Sweden elemental maps (sample IO-OM 78). As for Fig. S94 but showing different ooids from the same sample.

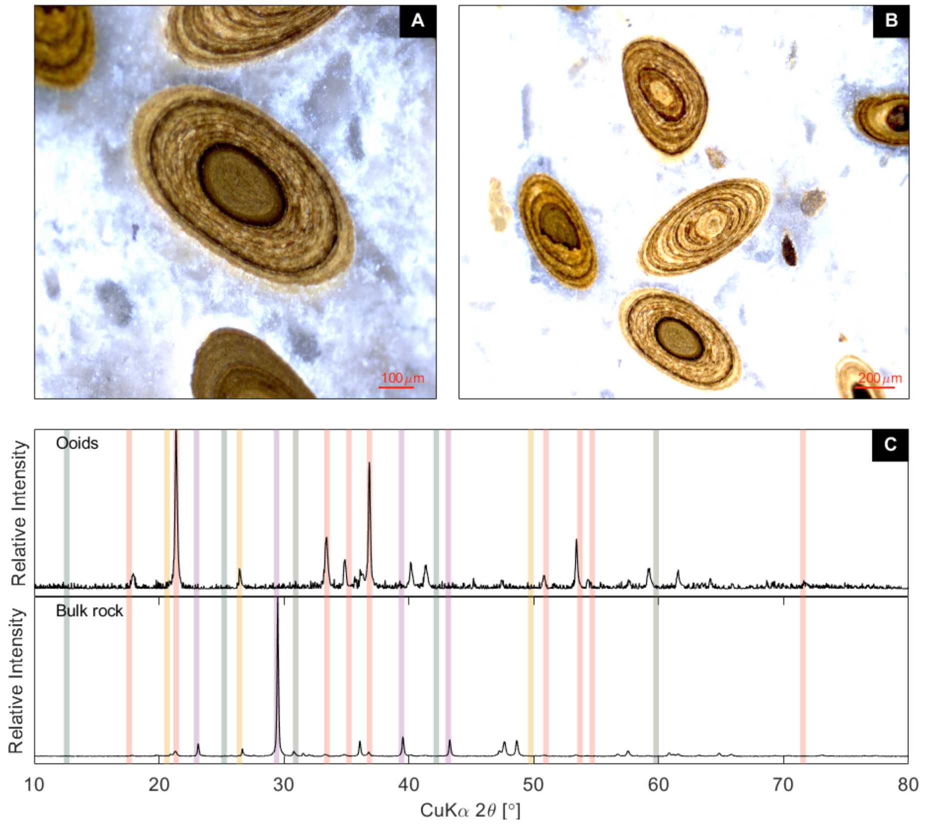

Fig. S96: Aseri Fm, Estonia petrography (sample IO-OM 67). (**A-B**) Optical microscopy images (SEM images were attempted for this sample but did not yield additional information due to porosity and epoxy resin artifacts) and (**C**) XRD diffractogram of bulk rock and individual ooids. Vertical bars in (**C**) are the same as described in Fig. S25. Images display matrix-supported spherical goethite ooids in a dolomite matrix.

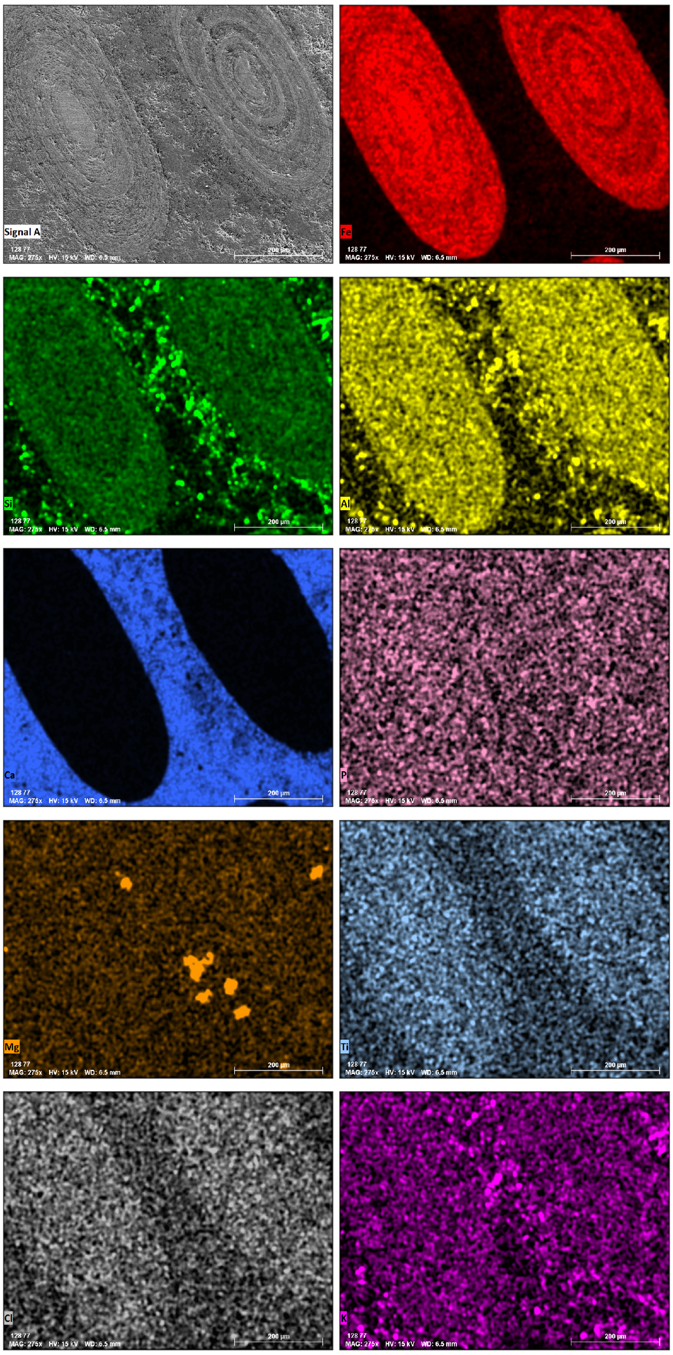

Fig. S97: Aseri Fm, Estonia elemental maps (sample IO-OM 67). Element designations include Ca (blue), Fe (red), Si (green), Mg (orange), Ti (teal), K (purple), Cl (gray), P (pink), and Al (yellow). Distinct features include Ca-rich carbonate cement containing Fe-rich goethite ooids. All elements that yielded a signal above the noise threshold are shown.

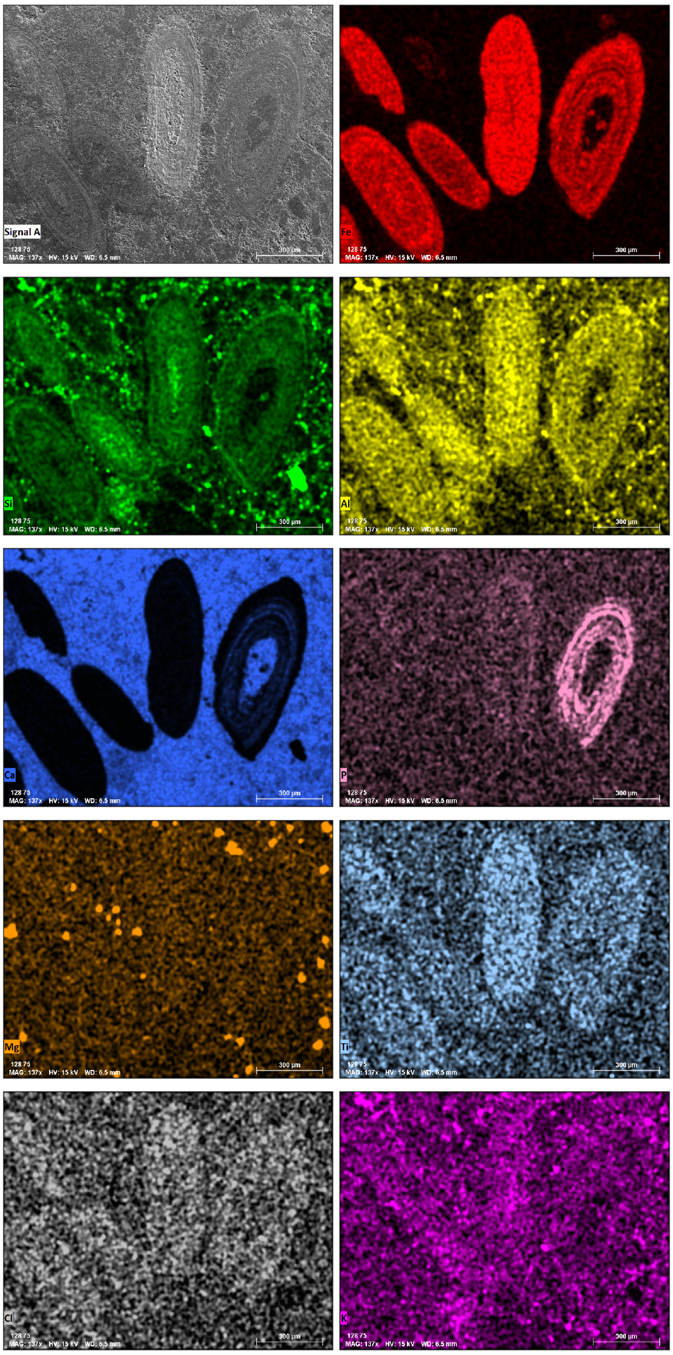

Fig. S98: Aseri Fm, Estonia elemental maps (sample IO-OM 67). As for Fig. S97 but showing different ooids from the same sample.

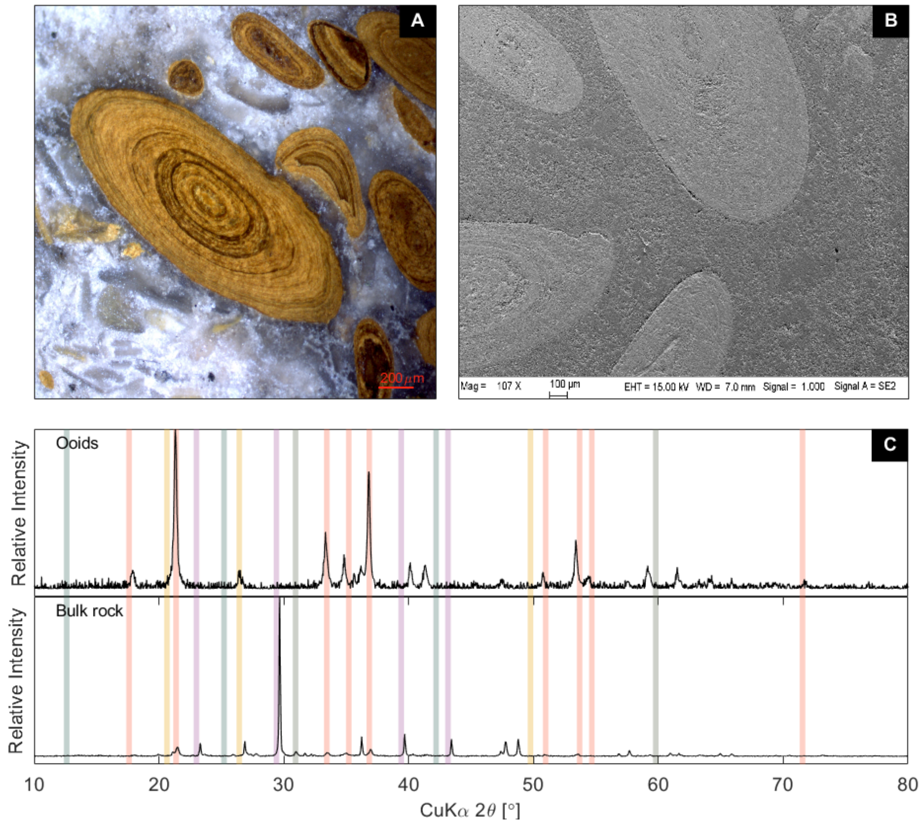

Fig. S99: Kunda Oolite Bed, Sillaoru Fm, Estonia and Russia petrography (sample IO-OM 70). (**A**) Optical and (**B**) electron microscopy images and (**C**) XRD diffractogram of bulk rock and individual ooids. Vertical bars in (**C**) are the same as described in Fig. S25. Images display matrix-supported spherical goethite ooids in a dolomite matrix; ooid texture indicate early deformation before lithification.

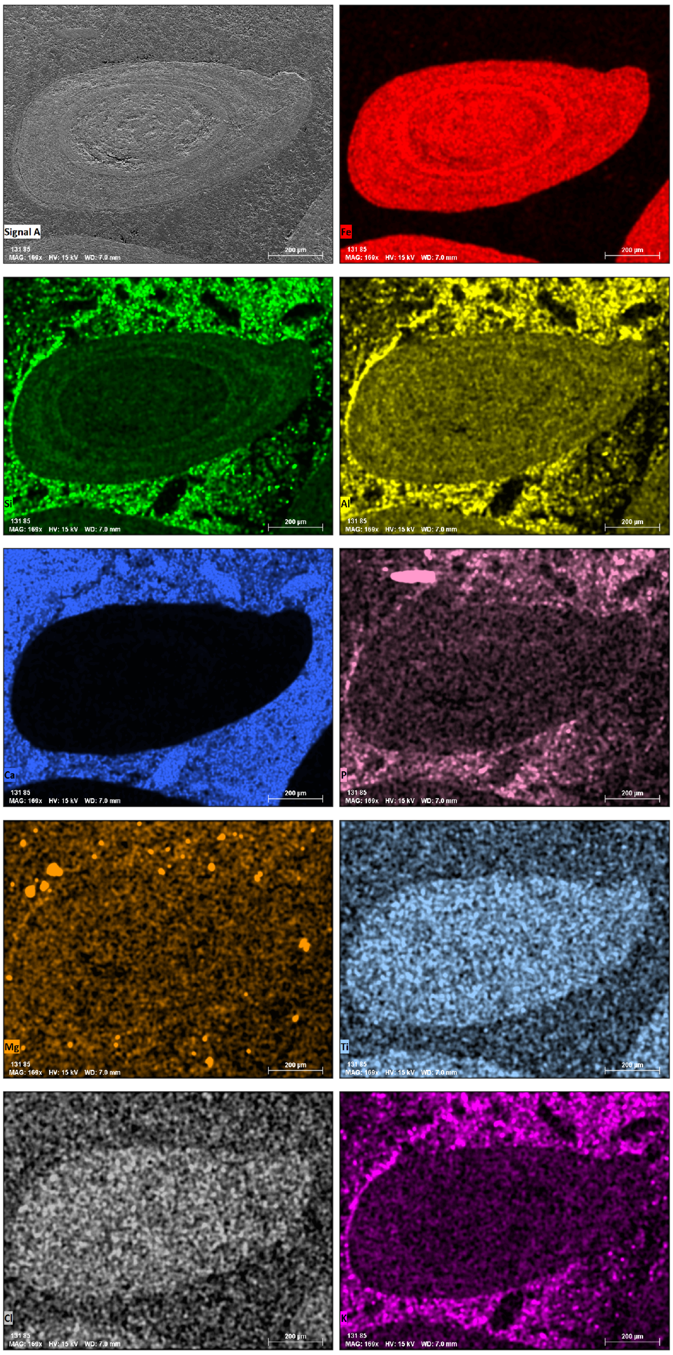

Fig. S100: Kunda Oolite Bed, Sillaoru Fm, Estonia and Russia elemental maps (sample IO-OM 70). Element designations include Ca (blue), Fe (red), Si (green), Mg (orange), Ti (teal), K (purple), Cl (gray), P (pink), and Al (yellow). Distinct features include Ca- and occasionally P-rich carbonate cement containing Fe-rich goethite ooids enriched in Cl and Ti. All elements that yielded a signal above the noise threshold are shown.

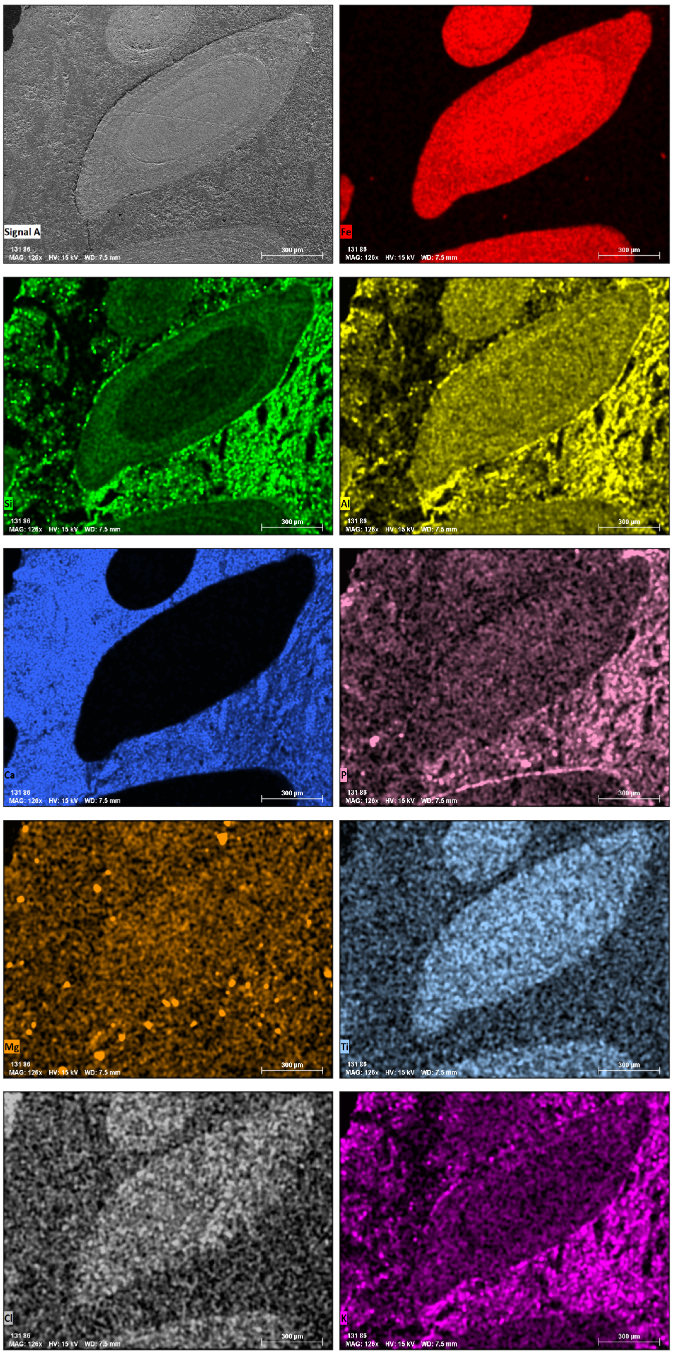

Fig. S101: Kunda Oolite Bed, Sillaoru Fm, Estonia and Russia elemental maps (sample IO-OM 70). As for Fig. S100 but showing different ooids from the same sample.

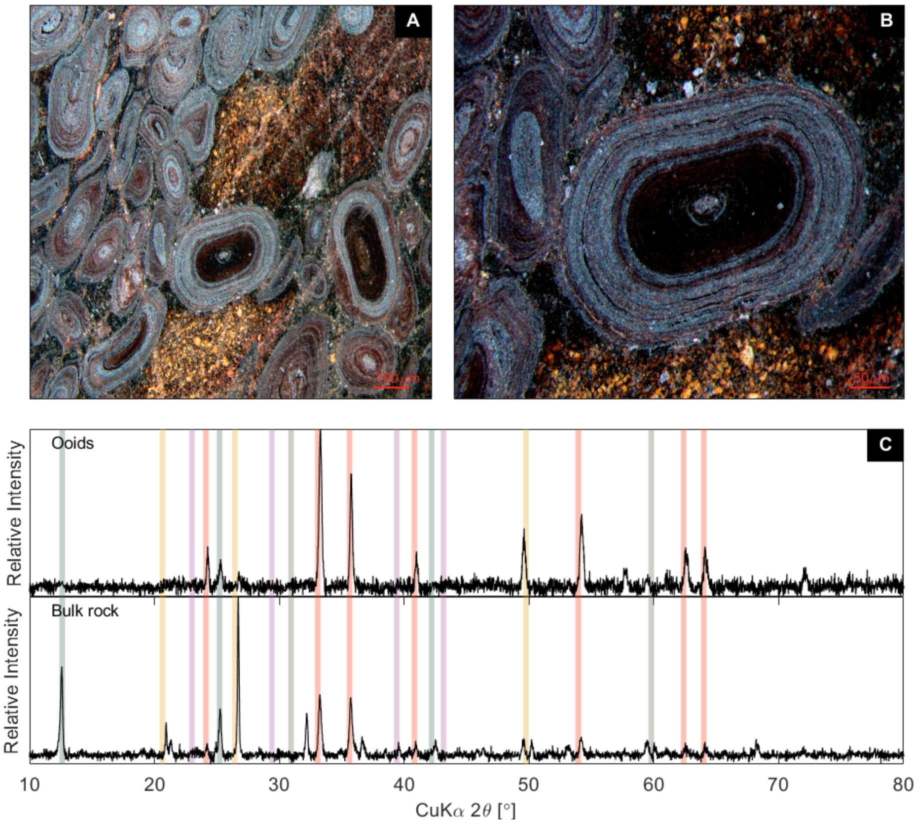

Fig. S102: Šárka Fm, Czechia petrography (sample IO-OM 81). (**A-B**) Optical microscopy images (SEM images were attempted for this sample but did not yield additional information due to porosity and epoxy resin artifacts) and (**C**) XRD diffractogram of bulk rock and individual ooids. Vertical bars in (**C**) are the same as described in Fig. S25. Images display grain-supported spherical hematite ooids that are deformed at times and hosted in a siliceous matrix.

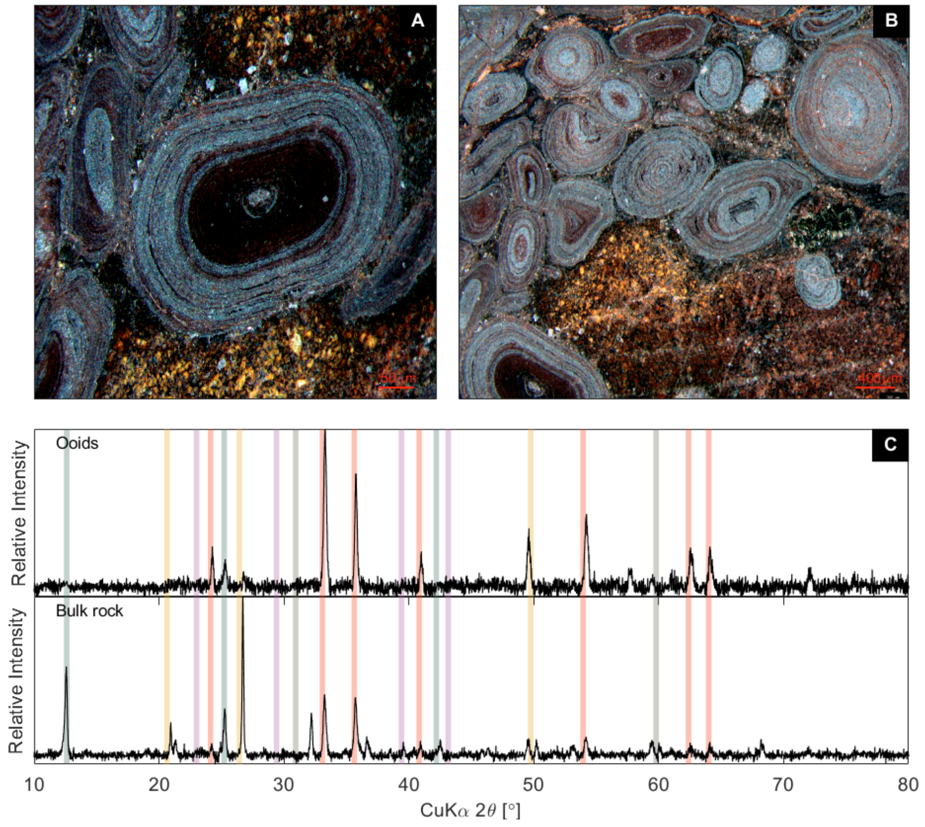

Fig. S103: Šárka Fm, Czechia petrography (sample IO-OM 82). As for Fig. S102 but showing ooids from a different sample from the same formation. Vertical bars in (**C**) are the same as described in Fig. S25.

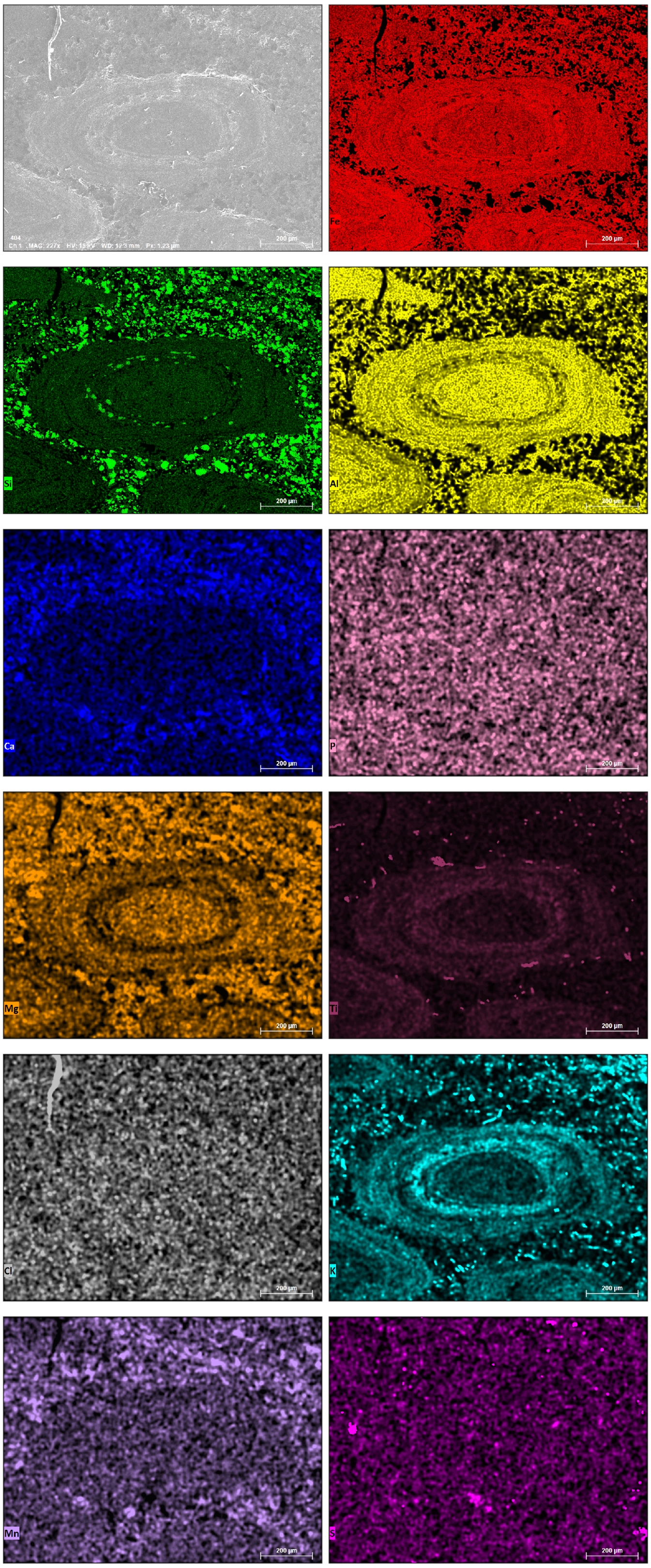

Fig. S104: Caption on following page.

Fig. S104: Šárka Fm, Czechia elemental maps (sample IO-OM 81). Element designations include Ca (blue), Fe (red), Si (green), Mg (orange), Ti (dark pink), K (teal), Cl (gray), P (pink), Mn (fuchsia), S (bright purple), and Al (yellow). Distinct features include Ca- and Mg-rich cement blended with K-, Si- and Al-rich cement as well as hematite ooids with an Al-, Mg, and Fe-rich core, indicating the presence of Fe-rich silicates. All elements that yielded a signal above the noise threshold are shown.

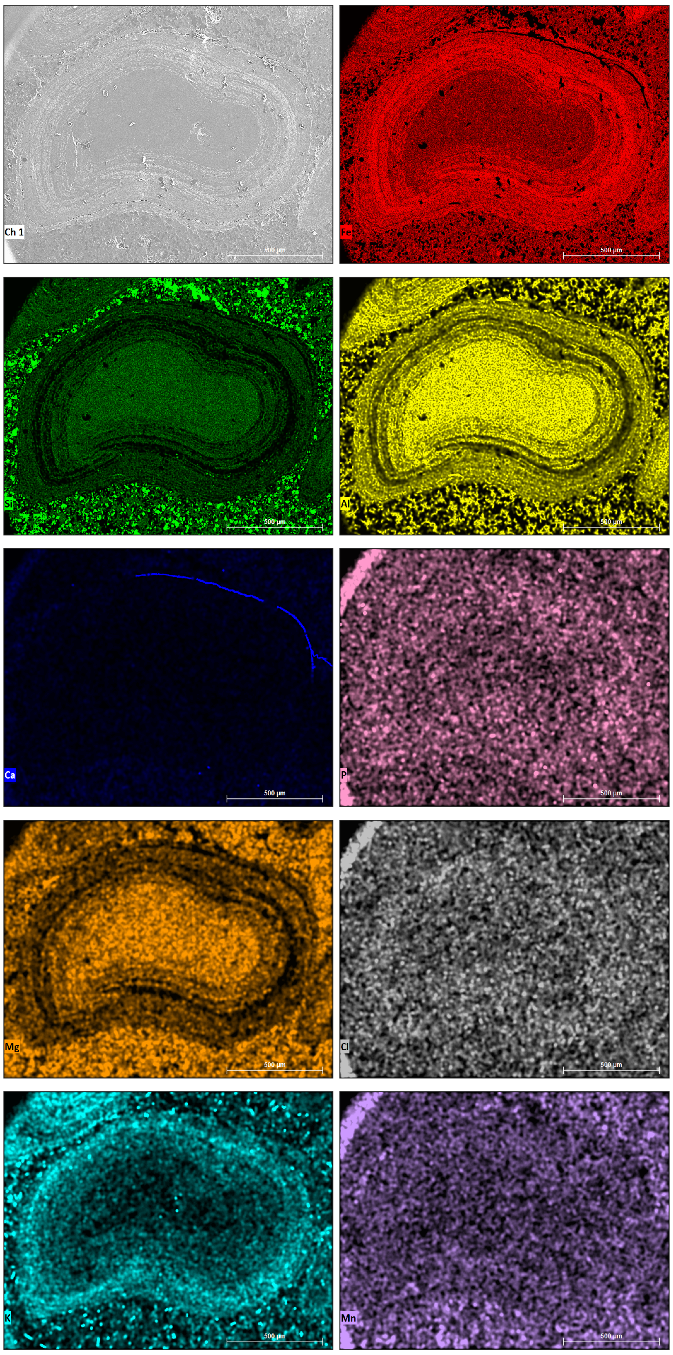

Fig. S105: Šárka Fm, Czechia elemental maps (sample IO-OM 82). As for Fig. S104 but showing ooids from a different sample from the same formation and omitting elemental designations for Ti and S.

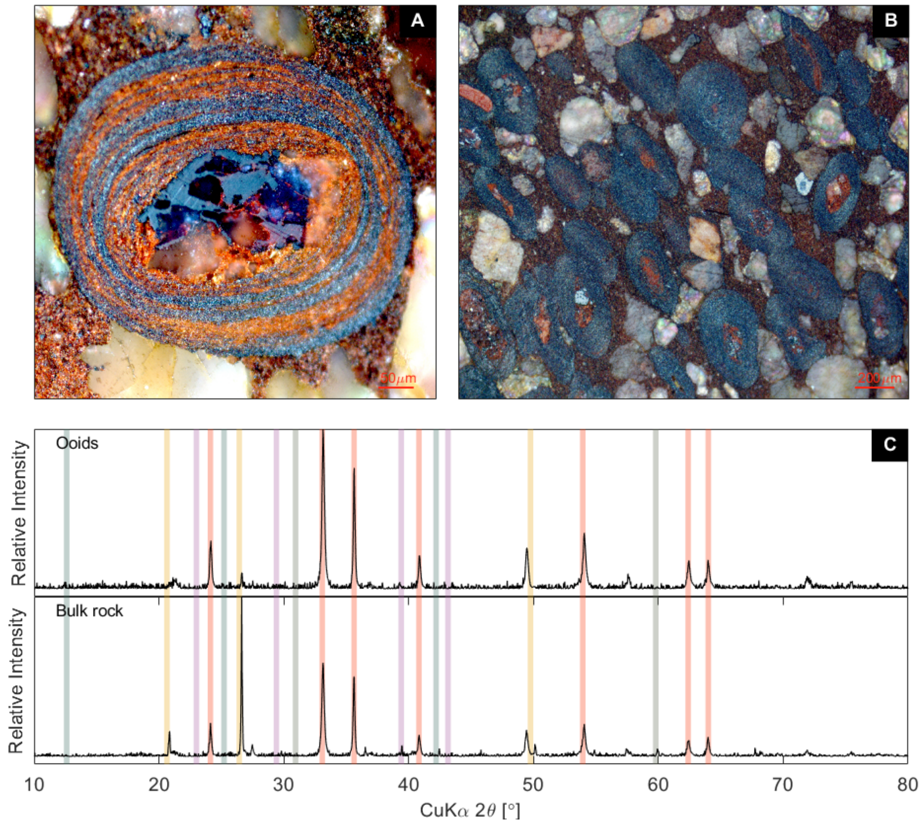

Fig. S106: Bliss Fm, USA petrography (sample IO-OM 85). (**A**-**B**) Optical microscopy images (SEM images were attempted for this sample but did not yield additional information due to porosity and epoxy resin artifacts) and (**C**) XRD diffractogram of bulk rock and individual ooids. Vertical bars in (**C**) are the same as described in Fig. S25. Images display matrix-supported spherical hematite ooids hosted in a siliceous matrix.

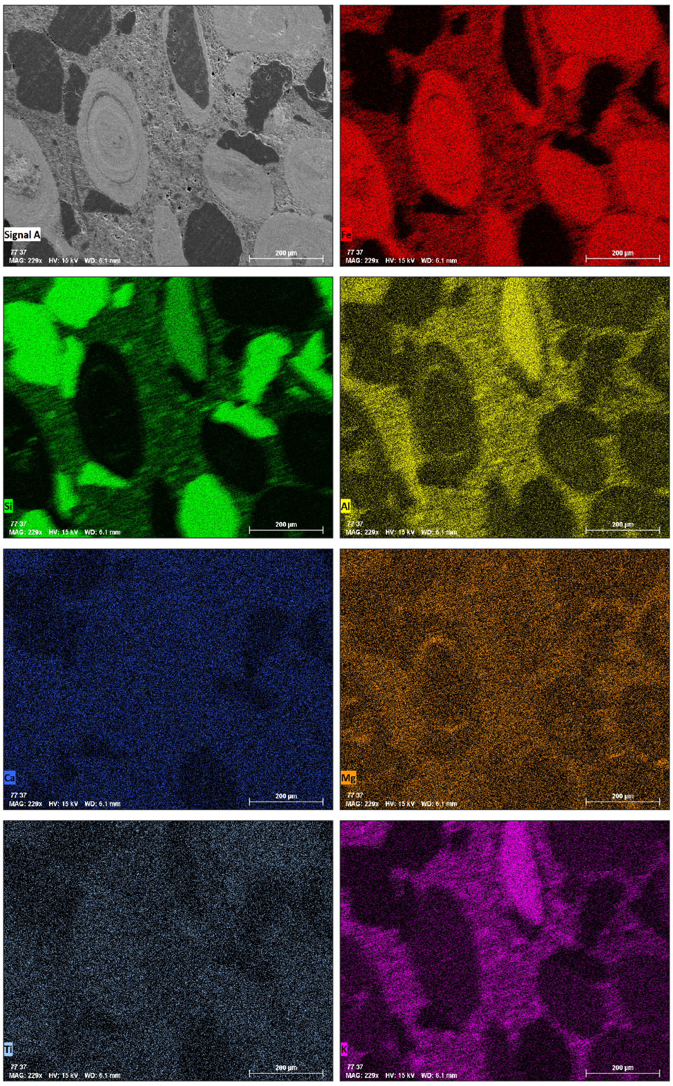

Fig. S107: Bliss Fm, USA elemental maps (sample IO-OM 85). Element designations include Ca (blue), Fe (red), Si (green), Mg (orange), K (purple), Ti (teal), and Al (yellow). Distinct features include a matrix rich in quartz grains and Fe-rich hematite ooids. All elements that yielded a signal above the noise threshold are shown.

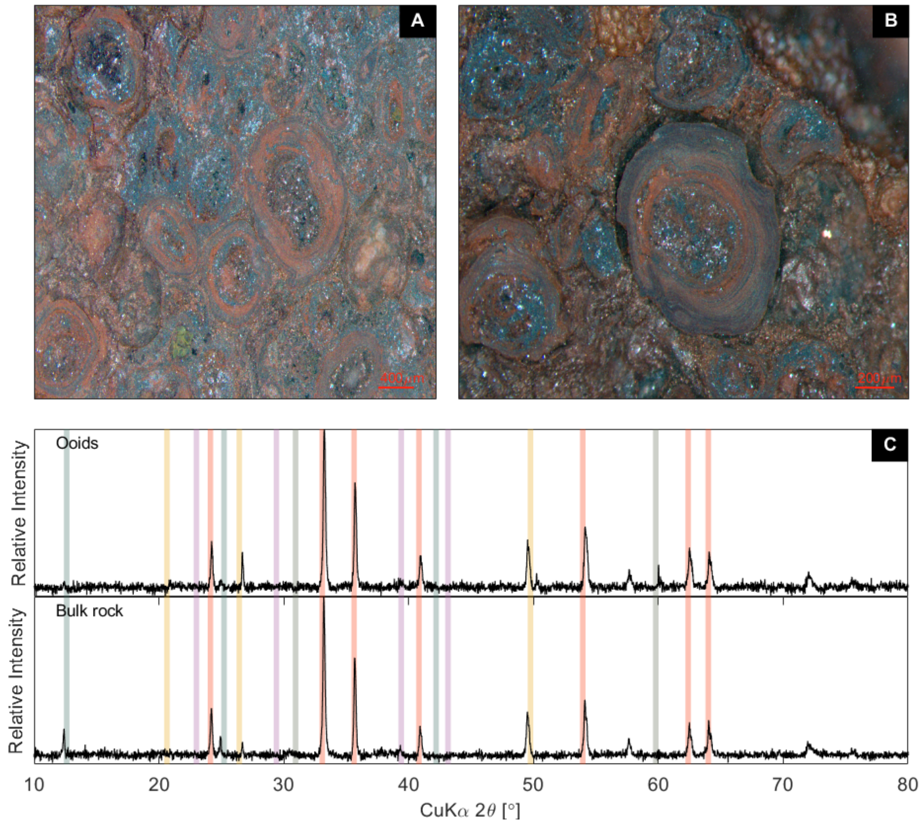

Fig. S108: Galeros Fm, Chuar Group, USA petrography (sample IO-OM 89). (**A**-**B**) Optical microscopy images (SEM images were attempted for this sample but did not yield additional information due to porosity and epoxy resin artifacts) and (**C**) XRD diffractogram of bulk rock and individual ooids. Vertical bars in (**C**) are the same as described in Fig. S25. Images display grain-supported spherical hematite ooids hosted in a siliceous-ferruginous matrix.

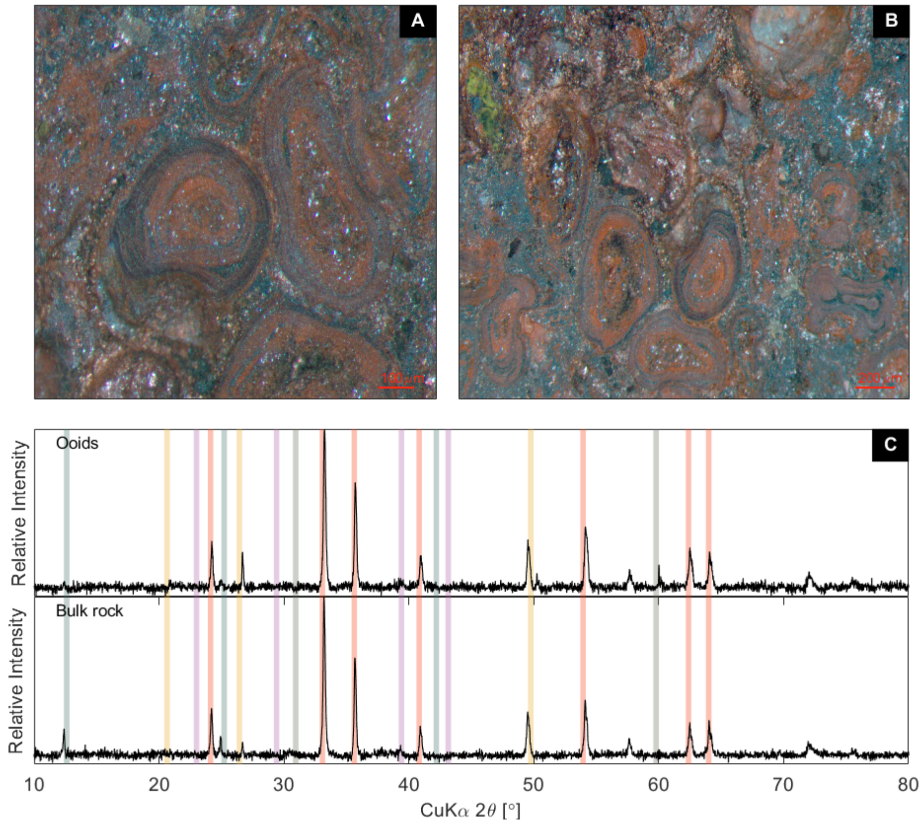

Fig. S109: Galeros Fm, Chuar Group, USA petrography (sample IO-OM 90). As for Fig. S108 but showing ooids from a different sample from the same formation. Vertical bars in (**C**) are the same as described in Fig. S25.

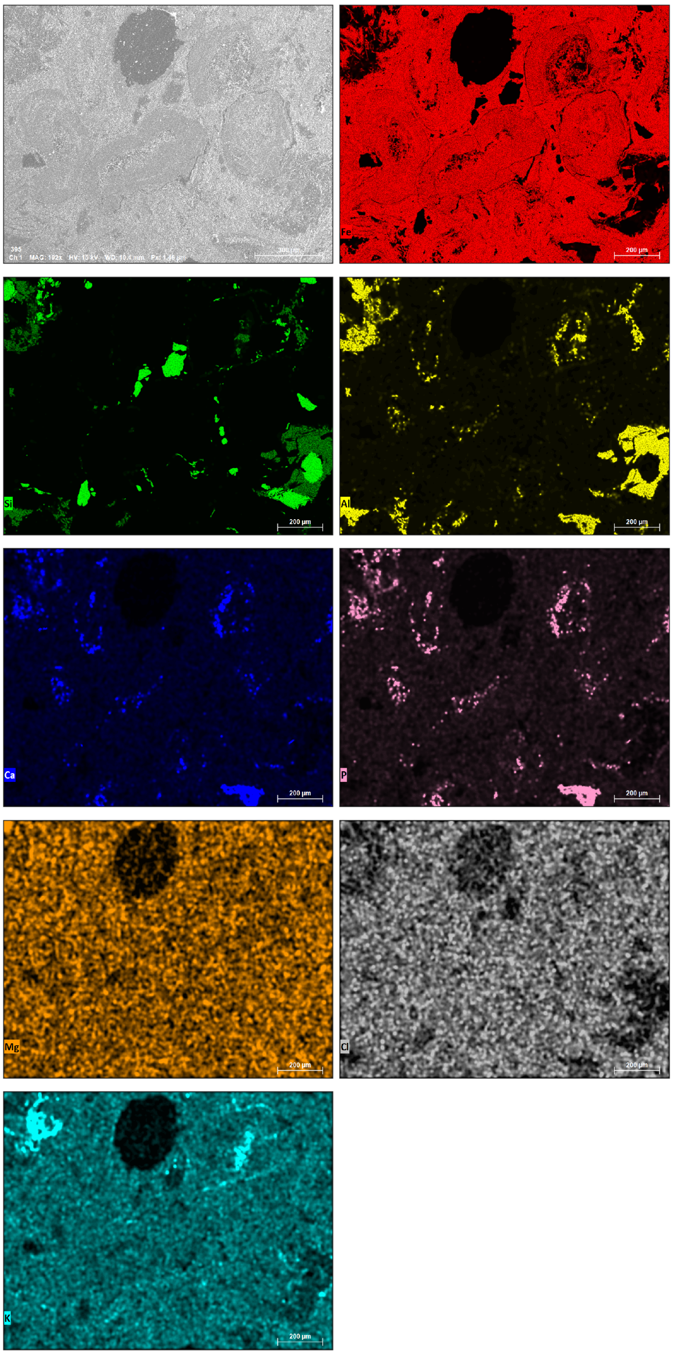

Fig. S110: Galeros Fm, Chuar Group, USA elemental maps (sample IO-OM 89). Element designations include Ca (blue), Fe (red), Si (green), Mg (orange), K (aquamarine), P (pink), Cl (gray), and Al (yellow). Distinct features include a matrix rich in quartz grains and Fe-rich hematite ooids. All elements that yielded a signal above the noise threshold are shown.

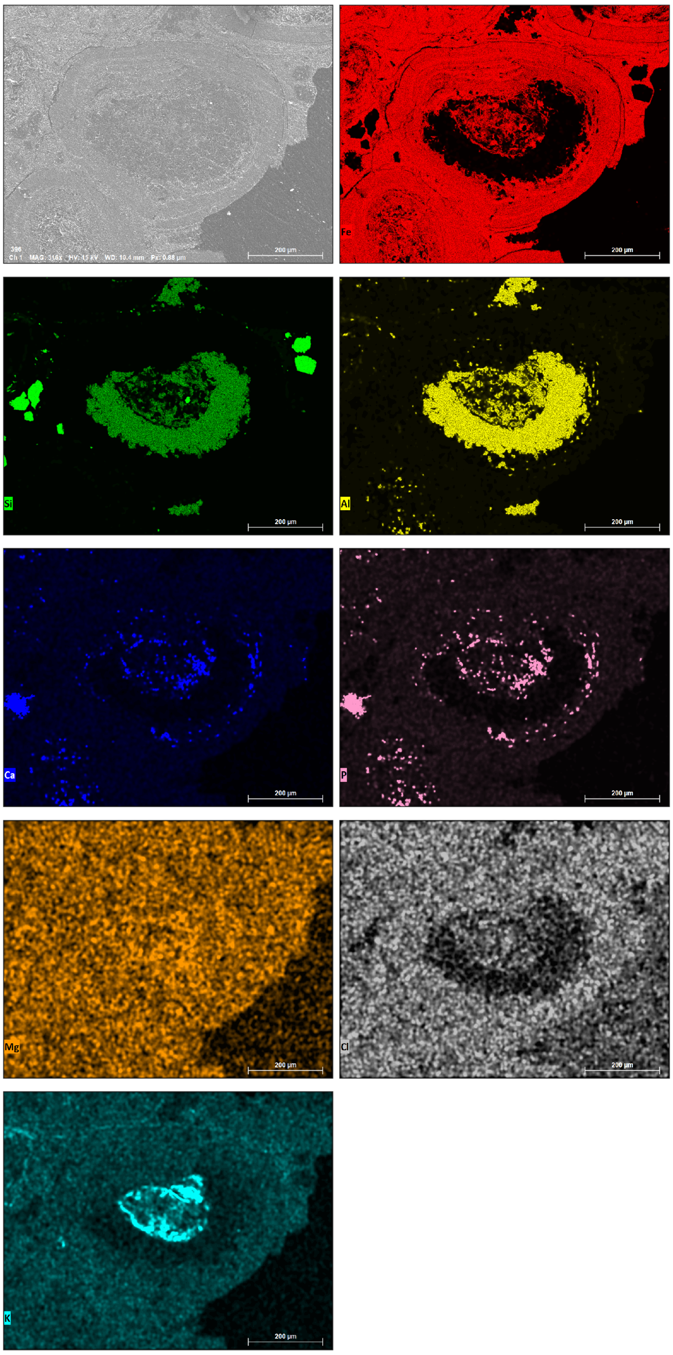

Fig. S111: Galeros Fm, Chuar Group, USA elemental maps (sample IO-OM 90). As for Fig. S110 but showing ooids from a different sample from the same formation.

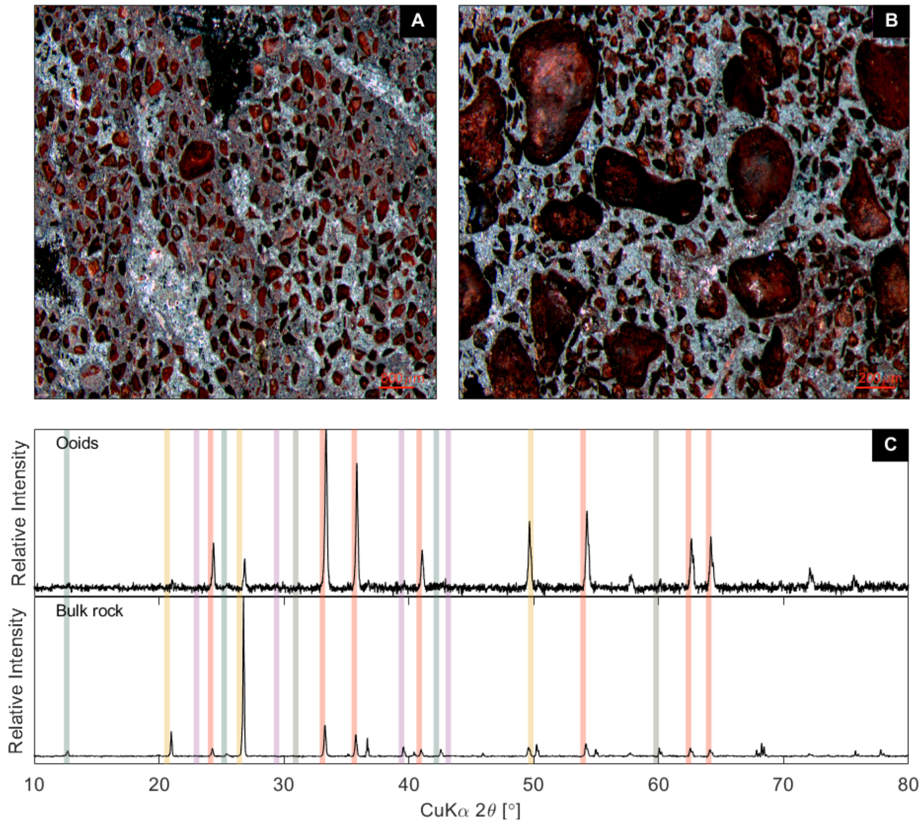

Fig. S112: McClure Fm, Katherine Group, Canada petrography (sample IO-OM 91). (**A-B**) Optical microscopy images (SEM images were attempted for this sample but did not yield additional information due to porosity and epoxy resin artifacts) and (**C**) XRD diffractogram of bulk rock and individual ooids. Vertical bars in (**C**) are the same as described in Fig. S25. Images display matrix-supported sub-spheroidal hematite pizoliths hosted in a siliceous-ferruginous matrix.

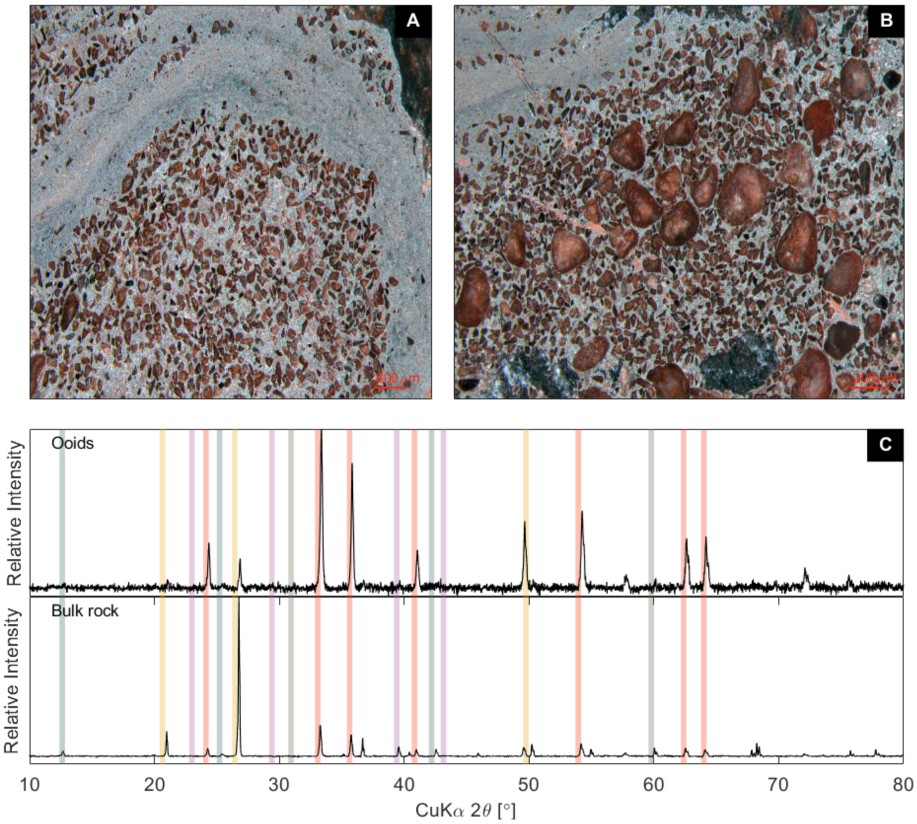

Fig. S113: McClure Fm, Katherine Group, Canada petrography (sample IO-OM 92). As for Fig. S112 but showing ooids from a different sample from the same formation. Vertical bars in (**C**) are the same as described in Fig. S25. Images display an hematite nodule filled with matrix-supported sub-spheroidal hematite pizoliths.

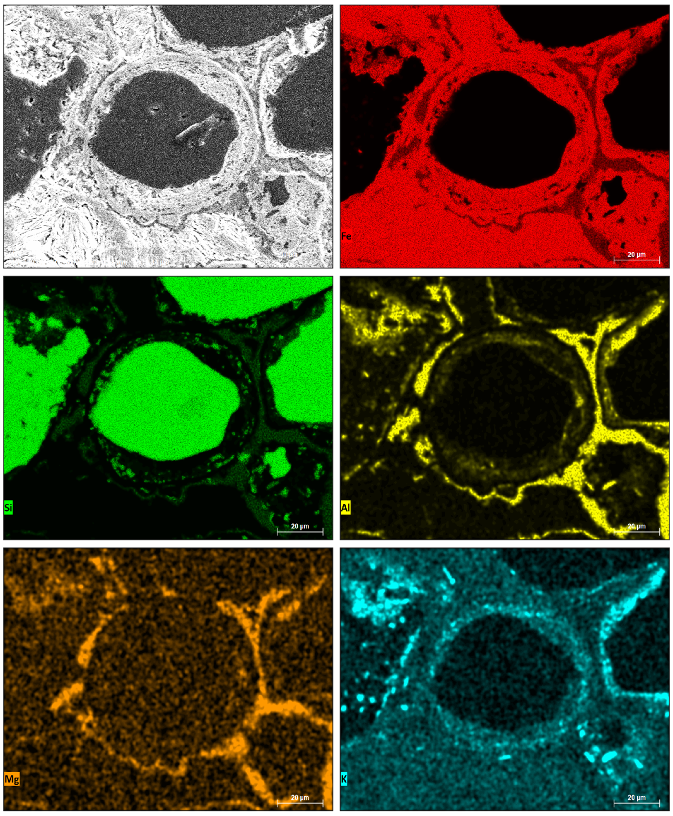

Fig. S114: McClure Fm, Katherine Group, Canada elemental maps (sample IO-OM 91). Element designations include Fe (red), Si (green), Mg (orange), K (aquamarine), and Al (yellow). Distinct features include coated hematite grains embedded in an Al- Mg- K- and Si-rich matrix, which we attribute to Fe-rich silicates. All elements that yielded a signal above the noise threshold are shown.

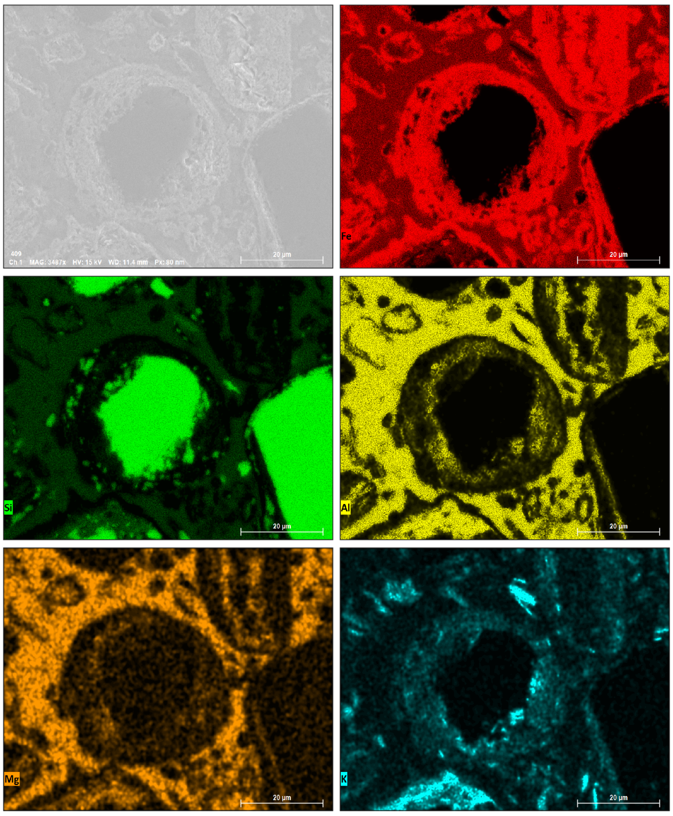

Fig. S115: McClure Fm, Katherine Group, Canada elemental maps (sample IO-OM 92). As for Fig. S114 but showing ooids from a different sample from the same formation.

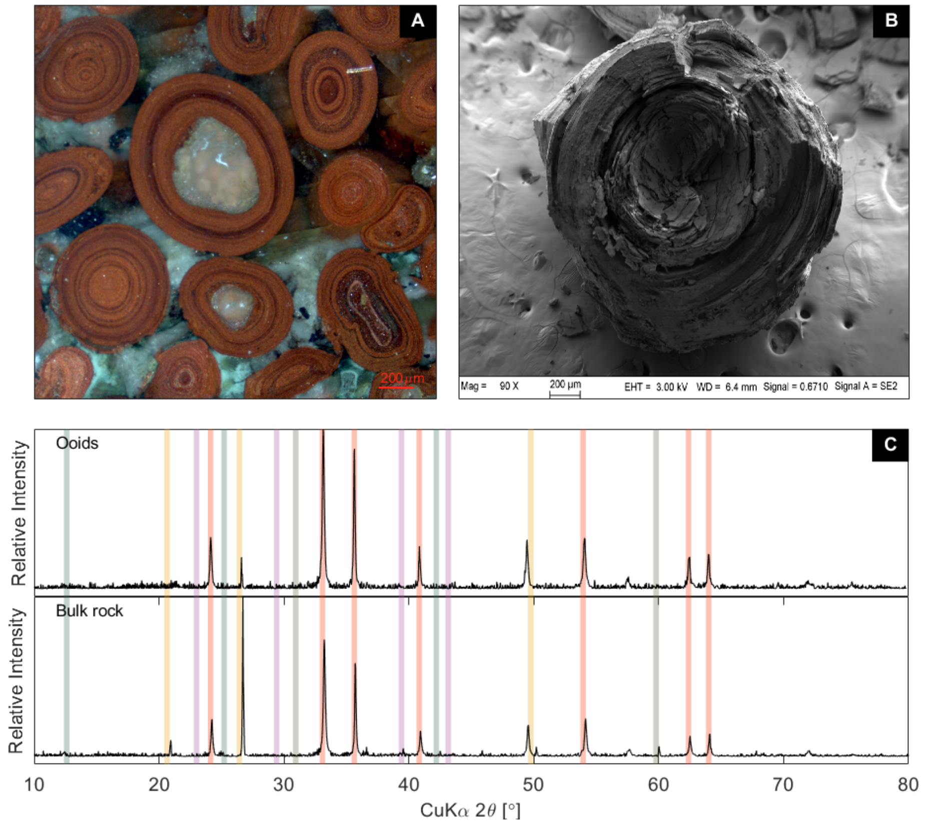

Fig. S116: Sherwin Ironstone, Sherwin Fm, Roper Group, Australia petrography (sample IO-OM 94). (**A**) Optical and (**B**) electron microscopy images and (**C**) XRD diffractogram of bulk rock and individual ooids. Vertical bars in (**C**) are the same as described in Fig. S25. Images display matrix-supported spherical hematite ooids in a siliceous matrix. Note the preserved fine lamination of hematite ooids.

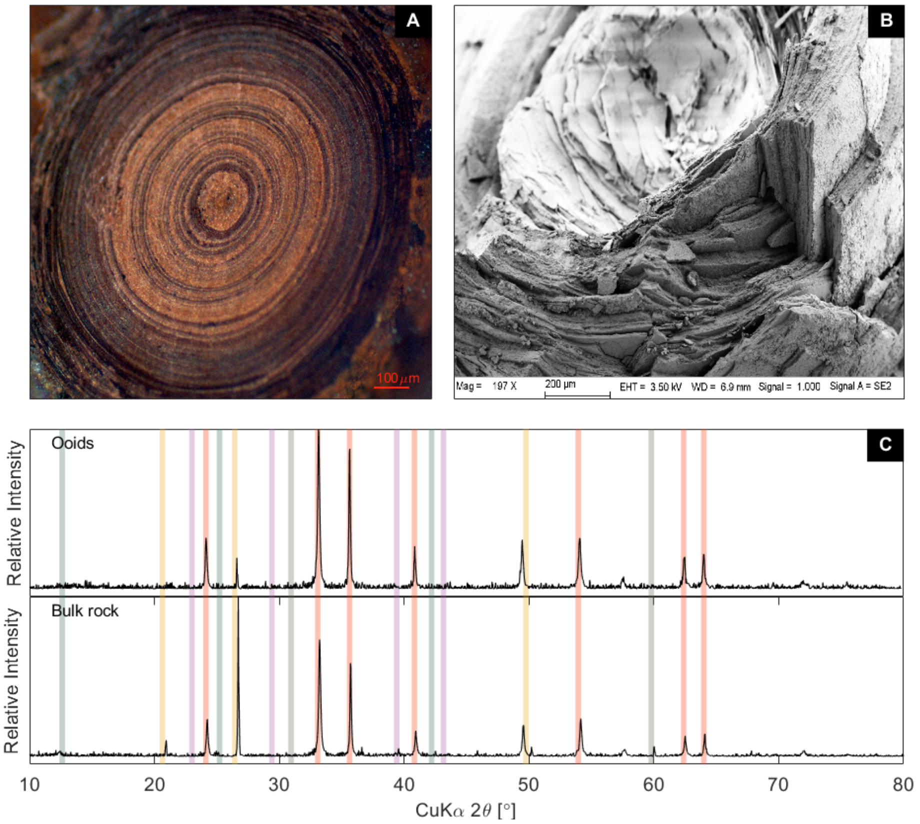

Fig. S117: Sherwin Ironstone, Sherwin Fm, Roper Group, Australia petrography (sample IO-OM 96). As for Fig. S116 but showing ooids from a different sample from the same formation. Vertical bars in (**C**) are the same as described in Fig. S25.

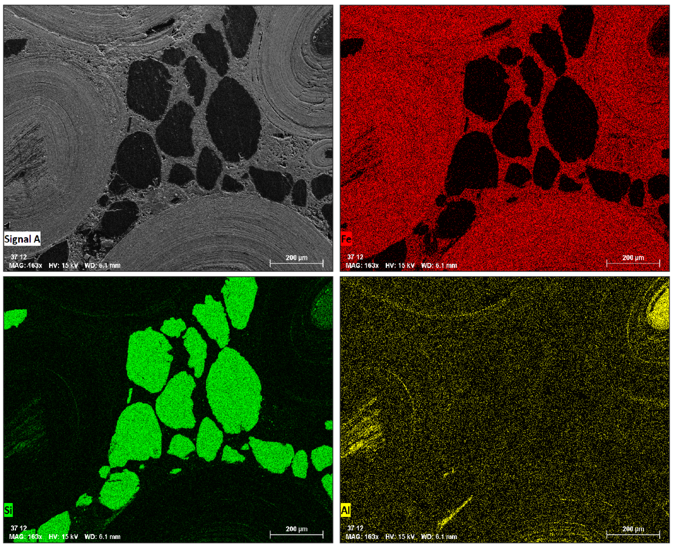

Fig. S118: Sherwin Ironstone, Sherwin Fm, Roper Group, Australia elemental maps (sample IO-OM 94). Element designations include Fe (red), Si (green), and Al (yellow). Distinct features include hematite ooids containing Al-rich cores. All elements that yielded a signal above the noise threshold are shown.

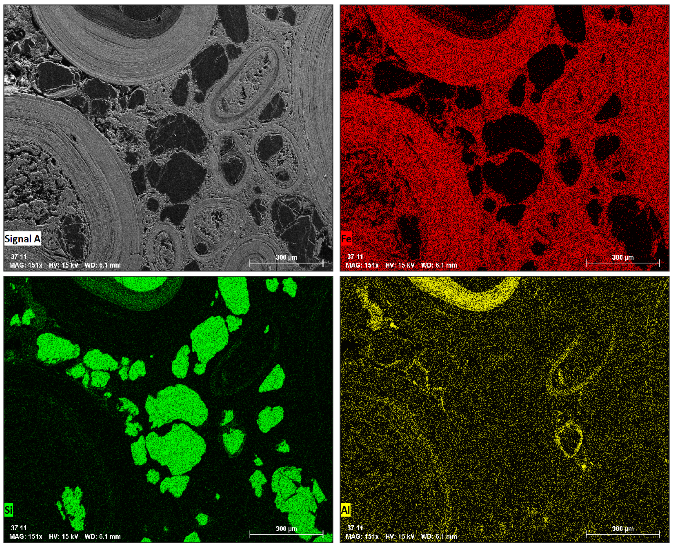

Fig. S119: Sherwin Ironstone, Sherwin Fm, Roper Group, Australia elemental maps (sample IO-OM 96). As for Fig. S118 but showing ooids from a different sample from the same formation.

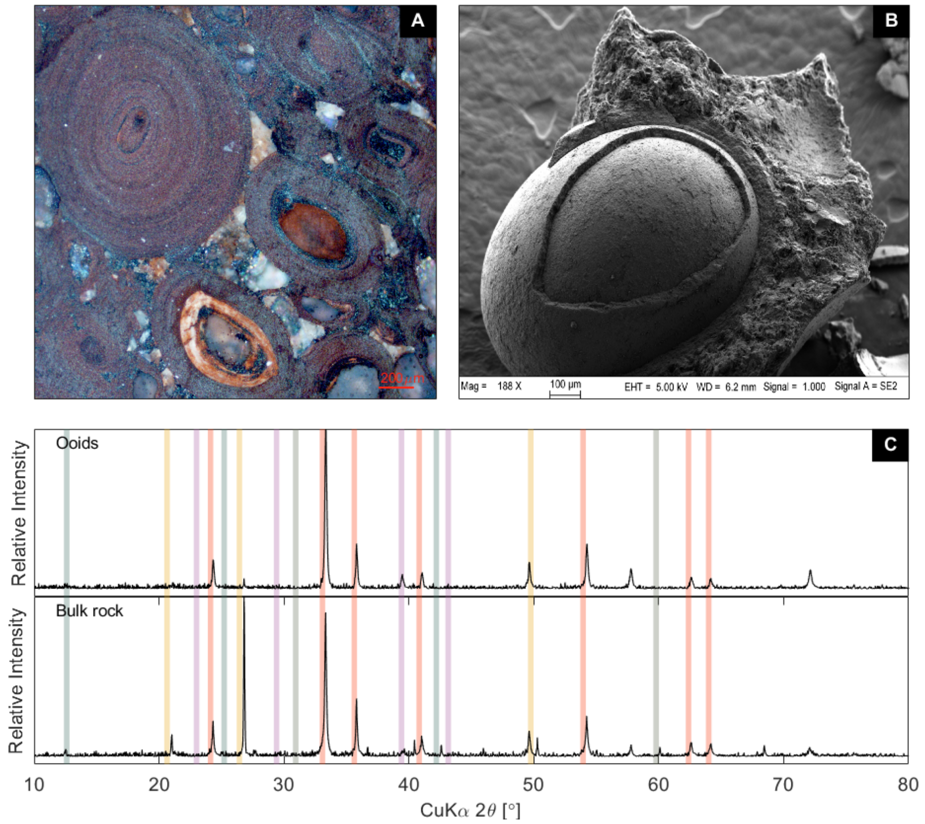

Fig. S120: Chuanlinggou Fm, Changcheng Group, China petrography (sample IO-OM 98). (**A**) Optical and (**B**) electron microscopy images and (**C**) XRD diffractogram of bulk rock and individual ooids. Vertical bars in (**C**) are the same as described in Fig. S25. Images display matrix-supported spherical hematite ooids in a siliceous matrix. Note the preserved fine lamination of hematite ooids.

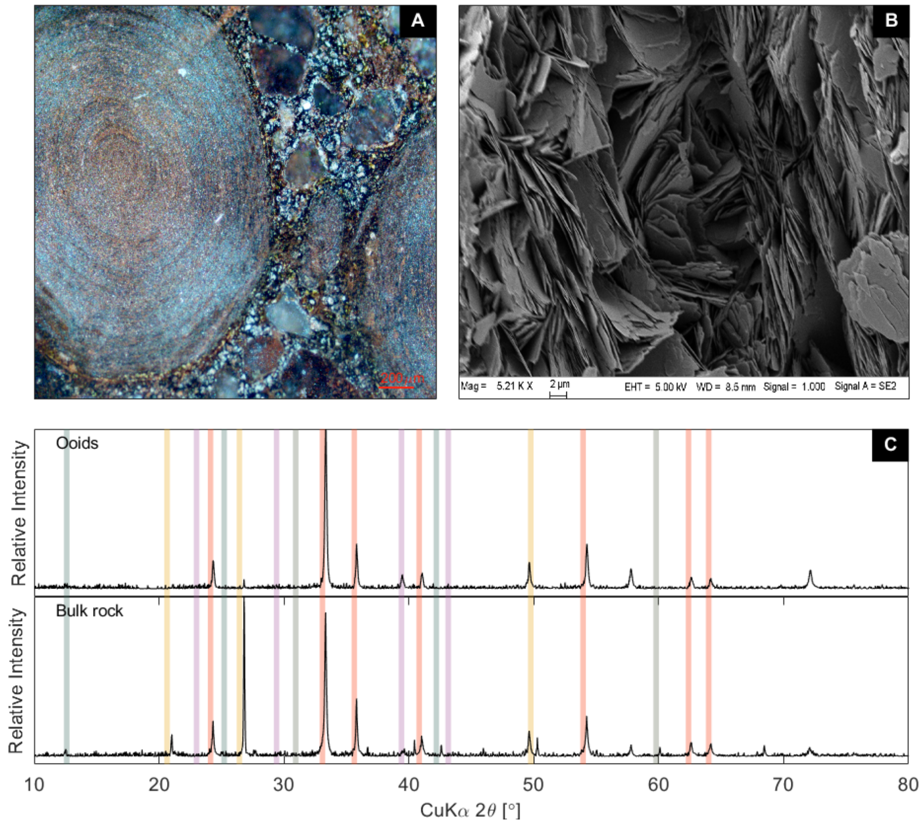

Fig. S121: Chuanlinggou Fm, Changcheng Group, China petrography (sample IO-OM 99). As for Fig. S120 but showing ooids from a different sample from the same formation. Vertical bars in (**C**) are the same as described in Fig. S25.

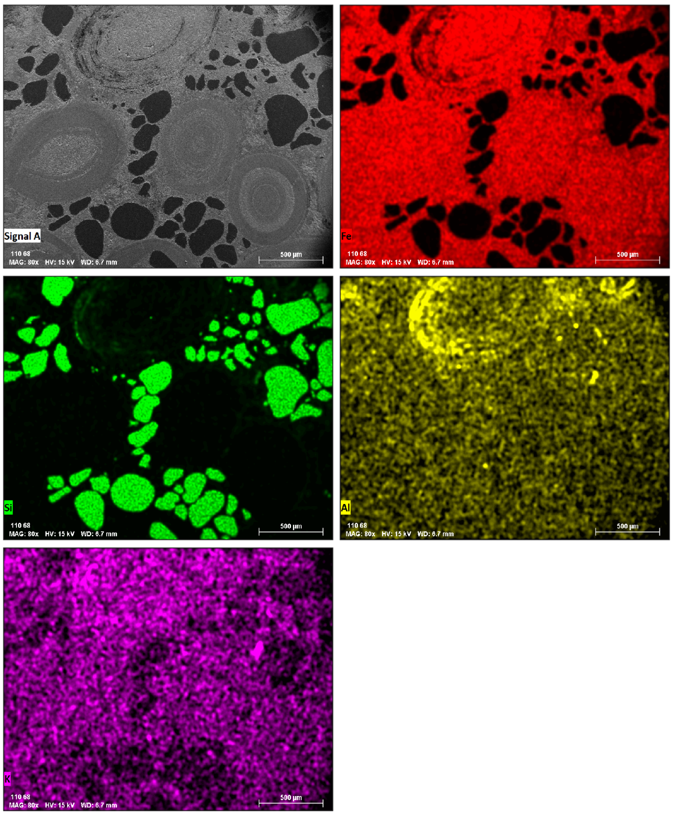

Fig. S122: Chuanlinggou Fm, Changcheng Group, China elemental maps (sample IO-OM 98). Element designations include Fe (red), Si (green), K (purple), and Al (yellow). Distinct features include hematite ooids embedded within Si-rich grains. All elements that yielded a signal above the noise threshold are shown.

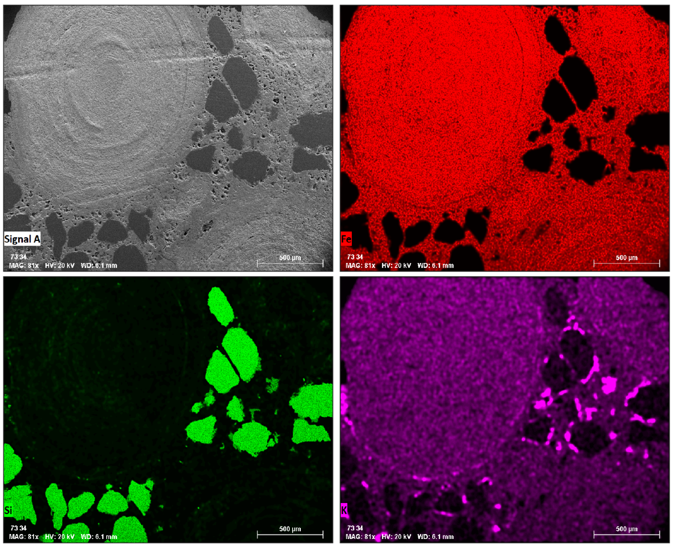

Fig. S123: Chuanlinggou Fm, Changcheng Group, China elemental maps (sample IO-OM 99). As for Fig. S122 but showing ooids from a different sample from the same formation and omitting elemental designations for Al.

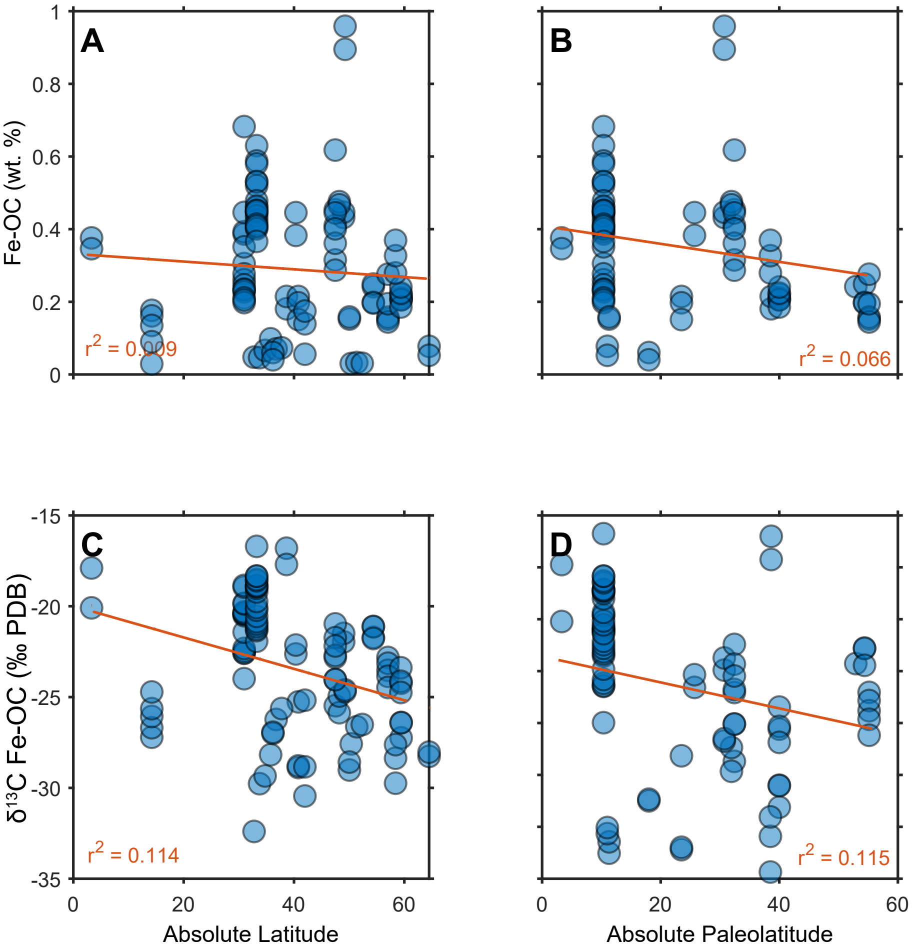

Fig. S124: Fe-OC signal correlation with (paleo)latitude. (**A**-**B**) Fe-OC loadings and (**C**-**D**) δ^{13}C values for all iron ooids included in this study plotted as function of (**A**, **C**) modern sampling latitude and (**B**, **D**) estimated paleolatitude at the time of formation. Paleolatitudes were calculated using the Paleolatitude Calculator and additional references[268–272]. Red lines lines are best-fit OLS regressions, including coefficients of determination (r^2 values); no statistically significant correlation is observed in any panel.

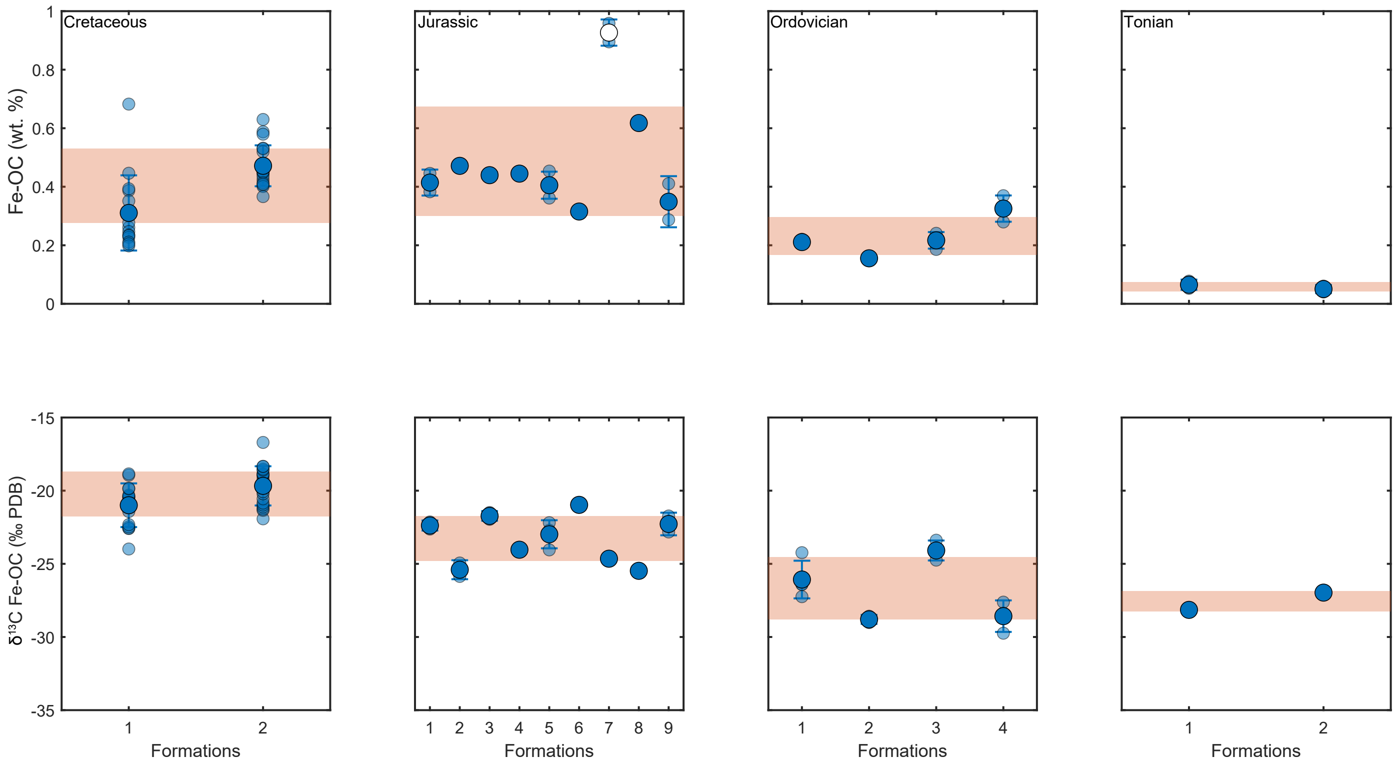

Fig. S125: Fe-OC signal intra-period spatial variability. (**top**) Fe-OC loadings and (**bottom**) δ^{13}C values for several formations collected from the Cretaceous, Jurassic, Ordovician, and Tonian periods. Semi-transparent markers are results for individual samples, whereas opaque markers are formation means $\pm 2\sigma$ uncertainty. Pink shaded regions represent the mean $\pm 2\sigma$ uncertainty for all formations within a given period. Formation numbers refer to: Cretaceous, 1 = Hatira Fm ($n = 15$), 2 = Hidra Fm ($n = 20$); Jurassic, 1 = Arroyofrío Bed ($n = 2$), 2 = Wedelsandstein Fm ($n = 2$), 3 = Oolithe Ferrugineuse de Villers Fm ($n = 2$), 4 = Humphriesi-Schichten Fm ($n = 1$), 5 = "Humphriesioolith" ($n = 3$), 6 = Ifenthal Fm ($n = 1$), 7 = Oolithe Ferrugineuse de Bayeux Fm ($n = 2$; note open marker as this is formation is a statistical outlier at the 2σ level), 8 = "Parkinsoni-Württembergica Schichten" ($n = 1$), 9 = Wutach Fm ($n = 2$); Ordovician, 1 = Aseri Fm ($n = 4$), 2 = Šárka Fm ($n = 3$), 3 = Sillaoru Fm ($n = 3$), 4 = Skovde Limestone ($n = 2$); Tonian, 1 = McClure Fm ($n = 2$), 2 = Galeros Fm ($n = 2$).

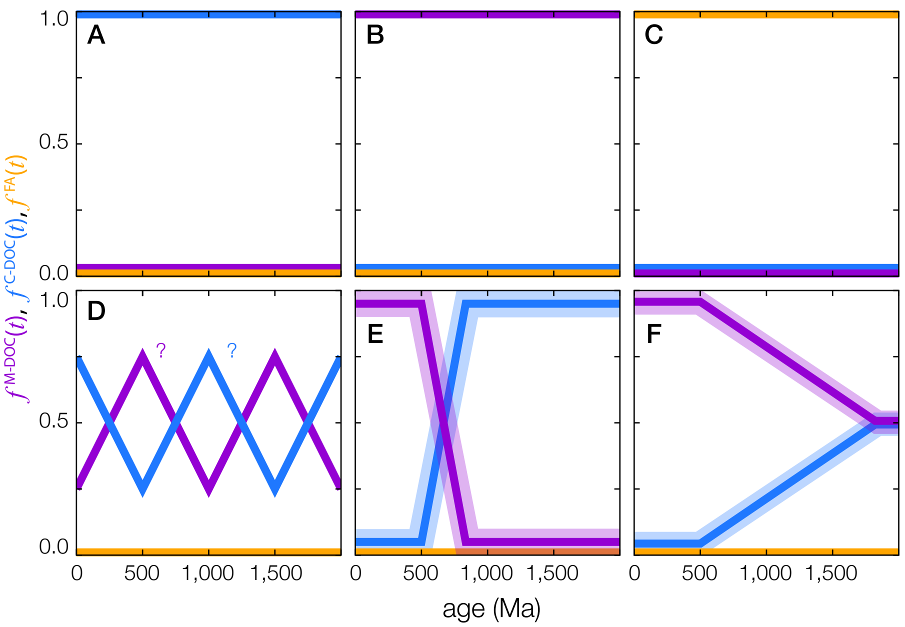

Fig. S126: Schematic representation of different $f^i(t)$ scenarios considered here. These include: (**A**) cyanobacteria only ($f^{\text{C-DOC}}(t) = 1$, $f^{\text{M-DOC}}(t) = f^{\text{FA}}(t) = 0$), (**B**) modern-marine analog only ($f^{\text{M-DOC}}(t) = 1$, $f^{\text{C-DOC}}(t) = f^{\text{FA}}(t) = 0$), (**C**) soil humics only ($f^{\text{FA}}(t) = 1$, $f^{\text{M-DOC}}(t) = f^{\text{C-DOC}}(t) = 0$), (**D**) unconstrained $f^i(t)$ (minimize propagated error), (**E**) “Rise of Algae” (including $\pm 5\,\%$ uniform error about each prediction, shaded regions), and (**F**) “Proterozoic active eukaryotes” (including $\pm 5\,\%$ uniform error about each prediction, shaded regions). Colors refer to: blue = C-DOC, red = M-DOC, orange = FA.

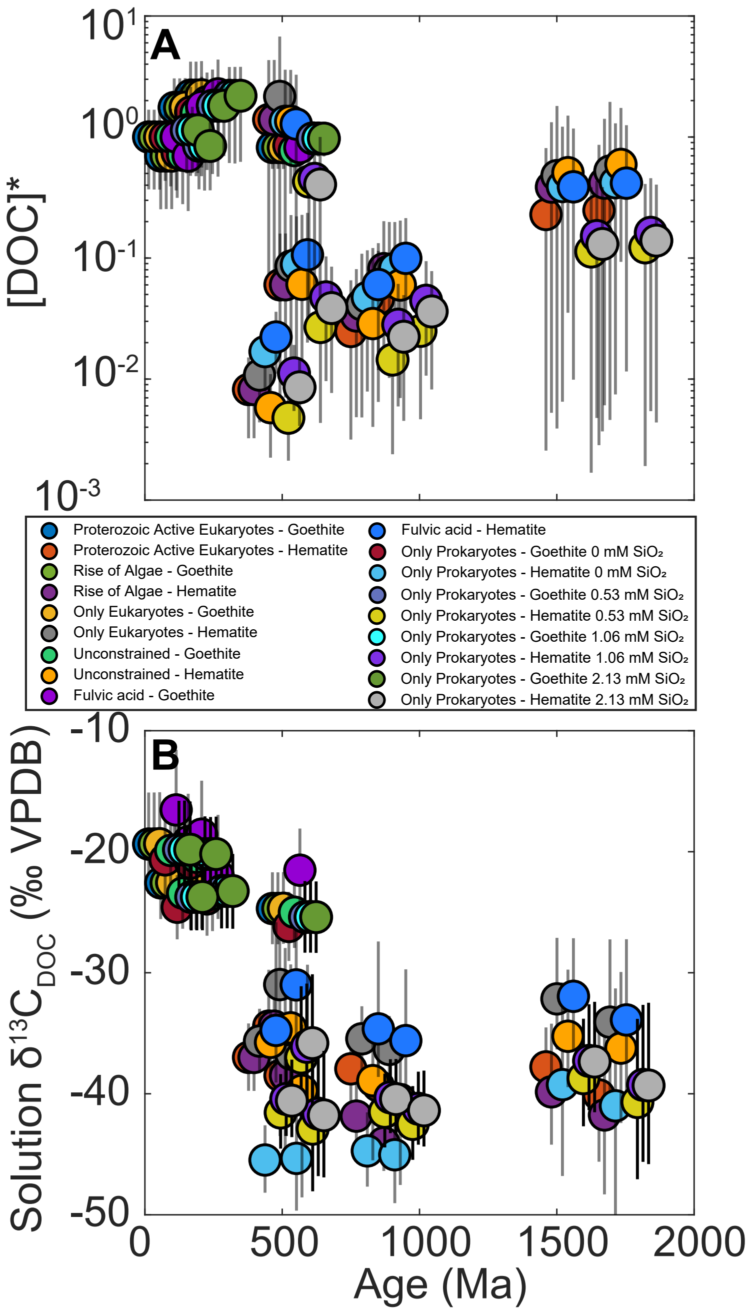

Fig. S127: Predicted Earth-history DOC concentrations and δ^{13}C values for all model scenarios considered here. Estimated DOC (**A**) concentrations and (**B**) δ^{13}C values over the past 2000 Ma for all model scenarios [cyanobacteria only; including predictions for multiple silica concentrations, modern-marine analog only, soil humics only, unconstrained $f^i(t)$, "Rise of algae" and "Proterozoic active eukaryotes" separated by mineralogy and binned into 100 Ma periods. Vertical lines are 95 % confidence intervals from Monte Carlo propagated uncertainty.

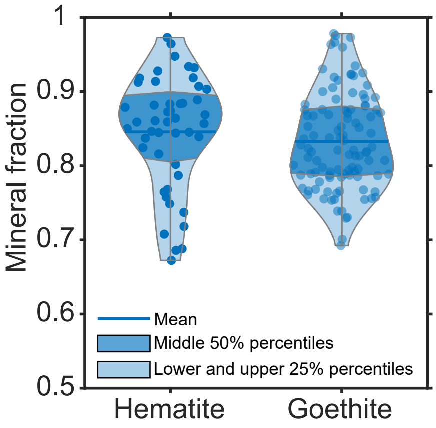

Fig. S128: Fraction of iron oxide in each ooid included in our record, separated by mineralogy and reported as violin plots. Percentages are derived from XRF-based Fe content, scaled by the fractional wt % Fe in each ideal mineral formula [i.e., FeO(OH) = 63 wt % Fe; Fe_2O_3 = 70 wt % Fe]. Iron oxides are the dominant ooid mineral phase in all cases.

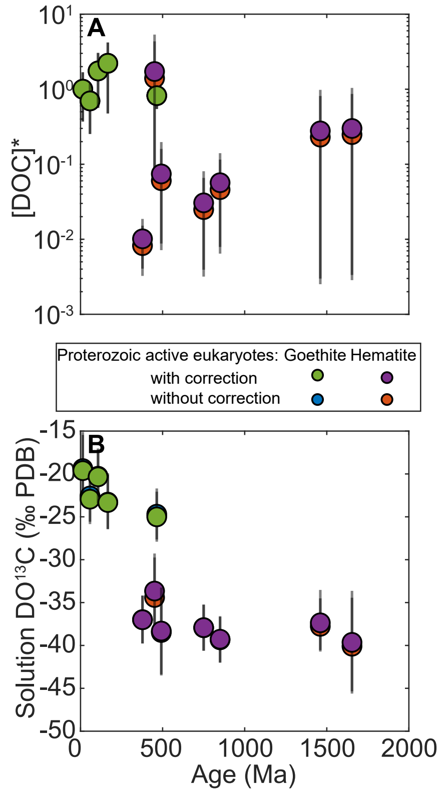

Fig. S129: Predicted Earth-history DOC concentrations and δ^{13}C values with and without XRF-based ooid oxide content correction. Estimated DOC (**A**) concentrations and (**B**) δ^{13}C values over the past 2000 Ma for the "Proterozoic active eukaryotes" model scenario, separated by mineralogy and showing the effect of correcting measured Fe-OC loadings for accessory mineral content using XRF results. Vertical lines are $95\,\%$ confidence intervals from Monte Carlo propagated uncertainty. Given that ooids are consistently dominated by iron oxides with small accessory-phase contributions, this correction results in a statistically insignificant shift at all time points.

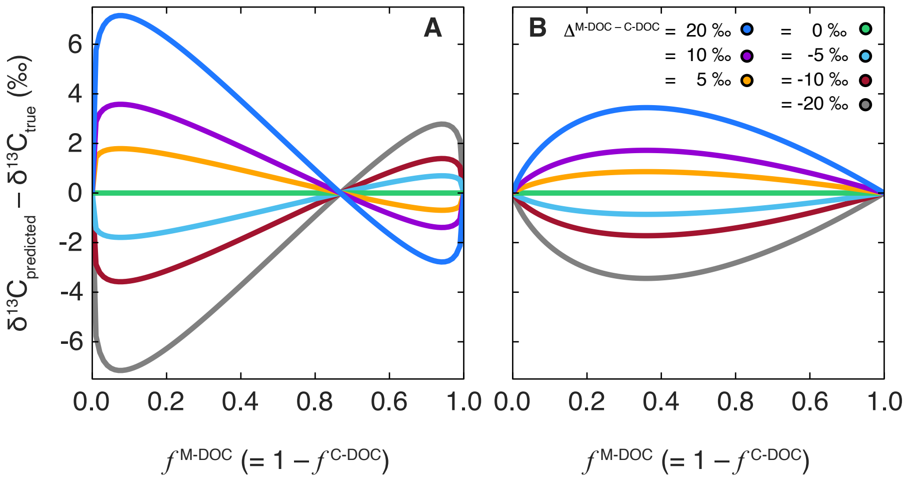

Fig. S130: Reconstructed marine DOC δ^{13}C bias as a function of end-member δ^{13}C offsets. Biases result from the fact that generally $w^{i,m}(t) \neq f^i(t)$ due to differential loadings between end members (Assumption 5). Both cases show reconstructed bias as a function of the δ^{13}C difference between M-DOC and C-DOC ($\Delta^{\text{M-DOC–C-DOC}}$; line colors) for mixtures of M-DOC and C-DOC using experimentally calculated loadings (Figs. S18-S19). Conditions are (**A**) $[\text{DOC}]^*(t) = 0.1$ as reconstructed by hematite (representing Proterozoic conditions) and (**B**) $[\text{DOC}]^*(t) = 1$ as reconstructed by goethite (representing Phanerozoic conditions).

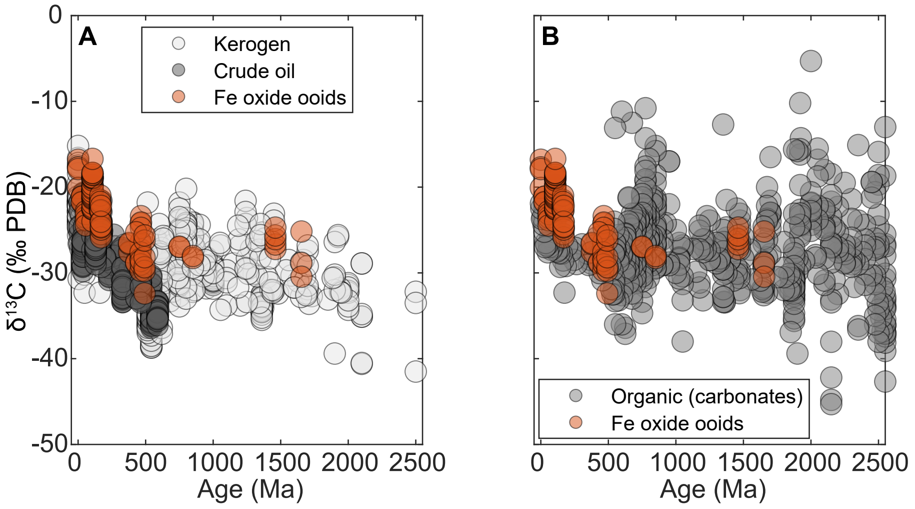

Fig. S131: Compilation of organic δ^{13}C records from different geological materials through time. Materials included are (**A**) kerogen (light gray), crude oil (dark gray), and (**B**) carbonate-associated OC from carbonate rocks (drak gray)[56]; our iron ooid Fe-OC record is also shown in both panels (orange). Observed temporal trends are remarkably similar, particularly for kerogen, crude oil, and Fe-OC. In contrast, scatter in carbonate-associated OC signals likely arises from diagenetic alteration, which is expected to drive δ^{13}C toward more positive values.

Supplementary References

[125] Torn, M. S., Trumbore, S. E., Chadwick, O. A., Vitousek, P. M. & Hendricks, D. M. Mineral control of soil organic carbon storage and turnover. *Nature* **389**, 170–173 (1997).

[126] Murphy, K. R. *et al.* Measurement of dissolved organic matter fluorescence in aquatic environments: an interlaboratory comparison. *Environmental Science & Technology* **44**, 9405–9412 (2010).

[127] Halevy, I. & Bachan, A. The geologic history of seawater pH. *Science* **355**, 1069–1071 (2017).

[128] Schwertmann, U. & Murad, E. Effect of pH on the formation of goethite and hematite from ferrihydrite. *Clays and Clay Minerals* **31**, 277–284 (1983).

[129] Hansel, C. M. *et al.* Secondary mineralization pathways induced by dissimilatory iron reduction of ferrihydrite under advective flow. *Geochimica et Cosmochimica Acta* **67**, 2977–2992 (2003).

[130] Hansel, C. M., Benner, S. G., Nico, P. & Fendorf, S. Structural constraints of ferric (hydr)oxides on dissimilatory iron reduction and the fate of Fe(II). *Geochimica et Cosmochimica Acta* **68**, 3217–3229 (2004).

[131] ThomasArrigo, L. K., Byrne, J. M., Kappler, A. & Kretzschmar, R. Impact of organic matter on iron (II)-catalyzed mineral transformations in ferrihydrite–organic matter coprecipitates. *Environmental Science & Technology* **52**, 12316–12326 (2018).

[132] Ferretti, A. *et al.* Armoured sponge spicules from Panarea Island (Italy): implications for their fossil preservation. *Palaeogeography, Palaeoclimatology, Palaeoecology* **536**, 109379 (2019).

[133] Ingalls, M., Frantz, C. M., Snell, K. E. & Trower, E. J. Carbonate facies-specific stable isotope data record climate, hydrology, and microbial communities in Great Salt Lake, UT. *Geobiology* **18**, 566–593 (2020).

[134] Pei, Y., Suarez-Gonzalez, P., Duda, J.-P. & Reitner, J. Organic matter influence on ooid formation: new insights into classic examples (Great Salt Lake, USA; Triassic Germanic Basin, Germany). *bioRxiv* 2023–04 (2023).

[135] Flemming, H.-C. & Wingender, J. Relevance of microbial extracellular polymeric substances (EPSs) part I: structural and ecological aspects. *Water Science and Technology* **43**, 1–8 (2001).

[136] Lowenstam, H. A. & Weiner, S. *On Biomineralization* (Oxford University Press, USA, 1989).

[137] Chan, C., Emerson, D. & Luther III, G. The role of microaerophilic Fe-oxidizing microorganisms in producing banded iron formations. *Geobiology* **14**, 509–528 (2016).

[138] Emerson, D. *et al.* A novel lineage of proteobacteria involved in formation of marine Fe-oxidizing microbial mat communities. *PLoS ONE* **2**, e667 (2007).

[139] Kappler, A. *et al.* An evolving view on biogeochemical cycling of iron. *Nature Reviews Microbiology* **19**, 360–374 (2021).

[140] Halbach, M., Koschinsky, A. & Halbach, P. Report on the discovery of *Gallionella ferruginea* from an active hydrothermal field in the deep sea. *InterRidge News* **10**, 18–20 (2001).

[141] Jakus, N. *et al.* Nitrate removal by a novel lithoautotrophic nitrate-reducing, iron(II)-oxidizing culture enriched from a pyrite-rich limestone aquifer. *Applied and Environmental Microbiology* **87**, e00460–21 (2021).

[142] Sudek, L. A., Templeton, A. S., Tebo, B. M. & Staudigel, H. Microbial ecology of Fe(hydr)oxide mats and basaltic rock from Vailulu'u seamount, American Samoa. *Geomicrobiology Journal* **26**, 581–596 (2009).

[143] Edwards, K. J., Rogers, D. R., Wirsen, C. O. & McCollom, T. M. Isolation and characterization of novel psychrophilic, neutrophilic, Fe-oxidizing, chemolithoautotrophic α- and γ-proteobacteria from the deep sea. *Applied and Environmental Microbiology* **69**, 2906–2913 (2003).

[144] Makita, H. Iron-oxidizing bacteria in marine environments: recent progresses and future directions. *World Journal of Microbiology and Biotechnology* **34**, 110 (2018).

[145] Zavarzina, D. G., Gavrilov, S. N. & Zhilina, T. N. Direct Fe(III) reduction from synthetic ferrihydrite by haloalkaliphilic lithotrophic sulfidogens. *Microbiology* **87**, 164–172 (2018).

[146] Aoki, M., Nakahara, N., Kusube, M. & Syutsubo, K.-i. Metagenome-assembled genome sequence of marine Rhizobiaceae sp. strain MnEN-MB40S, obtained from manganese-oxidizing enrichment culture. *Microbiology Resource Announcements* **11**, e00645–22 (2022).

[147] Brisbarre, N. *et al.* *Clostridium caminithermale* sp. nov., a slightly halophilic and moderately thermophilic bacterium isolated from an Atlantic deep-sea hydrothermal chimney. *International Journal of Systematic and Evolutionary Microbiology* **53**, 1043–1049 (2003).

[148] Ahmad, W. *et al.* *Marinobacter salinexigens* sp. nov., a marine bacterium isolated from hadal seawater of the Mariana Trench. *International Journal of Systematic and Evolutionary Microbiology* **70**, 3794–3800 (2020).

[149] Christman, G. D., León-Zayas, R. I., Zhao, R., Summers, Z. M. & Biddle, J. F. Novel clostridial lineages recovered from metagenomes of a hot oil reservoir. *Scientific Reports* **10**, 8048 (2020).

[150] Cao, J. *et al.* *Marinobacter profundi* sp. nov., a slightly halophilic bacterium isolated from a deep-sea sediment sample of the New Britain Trench. *Antonie van Leeuwenhoek* **112**, 425–434 (2019).

[151] Handley, K. M., Hery, M. & Lloyd, J. R. *Marinobacter santoriniensis* sp. nov., an arsenate-respiring and arsenite-oxidizing bacterium isolated from hydrothermal sediment. *International Journal of Systematic and Evolutionary Microbiology* **59**, 886–892 (2009).

[152] Bonis, B. M. & Gralnick, J. A. *Marinobacter subterrani*, a genetically tractable neutrophilic Fe(II)-oxidizing strain isolated from the Soudan iron mine. *Frontiers in Microbiology* **6**, 719 (2015).

[153] Jain, A., Bonis, B. M. & Gralnick, J. A. Oligo-heterotrophic activity of *Marinobacter subterrani* creates an indirect Fe(II) oxidation phenotype in gradient tubes. *Applied and Environmental Microbiology* **87**, e01367–21 (2021).

[154] Flieder, M. *et al.* Novel taxa of Acidobacteriota implicated in seafloor sulfur cycling. *The ISME Journal* **15**, 3159–3180 (2021).

[155] Longnecker, K., Sievert, S. M., Sylva, S. P., Seewald, J. S. & Kujawinski, E. B. Dissolved organic carbon compounds in deep-sea hydrothermal vent fluids from the East Pacific Rise at 9° 50' N. *Organic Geochemistry* **125**, 41–49 (2018).

[156] Yamashita, Y., Mori, Y. & Ogawa, H. Hydrothermal-derived black carbon as a source of recalcitrant dissolved organic carbon in the ocean. *Science Advances* **9**, eade3807 (2023).

[157] Yanitskiy, A. Oligocene oolitic iron ores of northern Turgai and its genesis. *AN USSR: Moscow, Russia* (1960).

[158] Slipchenko, B. About two genetic types of oolitic iron ores of the Lisakovsk deposit (Northern Kazakhstan). *Russian Geological Journal* **41**, 53–61 (1981).

[159] Iakovleva, A. I. Palynological reconstruction of the Eocene marine palaeoenvironments in south of Western Siberia. *Acta Palaeobotanica* **51**, 229–248 (2011).

[160] Rudmin, M. *et al.* Origin of oligocene channel ironstones of Lisakovsk deposit (Turgay depression, northern Kazakhstan). *Ore Geology Reviews* **138**, 104391 (2021).

[161] Gnibidenko, Z. N., Lebedeva, N. K. & Levicheva, A. V. Magnetostratigraphy of the Campanian-Maastrichtian Bakchar Basin (southeastern West Siberia). *Russian Geology and Geophysics* **56**, 1652–1661 (2015).

[162] Rudmin, M., Mazurov, A. & Banerjee, S. Origin of ooidal ironstones in relation to warming events: Cretaceous-Eocene Bakchar deposit, south-east Western Siberia. *Marine and Petroleum Geology* **100**, 309–325 (2018).

[163] Lebedeva, N. K., Kuzmina, O. B., Sobolev, E. S. & Khazina, I. V. Stratigraphy of Upper Cretaceous and Cenozoic deposits of the Bakchar iron ore deposit (southwestern Siberia): new data. *Stratigraphy and Geological Correlation* **25**, 76–98 (2017).

[164] Podobina, V. Regional stratigraphy and its dependency on tectonic movements (case study: Upper Cretaceous and Paleogene stages in Western Siberia). *IOP Conference Series: Earth and Environmental Science* **24**, 12012 (2015).

[165] Rudmin, M., Banerjee, S. & Mazurov, A. Compositional variation of glauconites in upper Cretaceous-Paleogene sedimentary iron-ore deposits in South-eastern Western Siberia. *Sedimentary geology* **355**, 20–30 (2017).

[166] Rosenfeld, A. & Hirsch, F. The Cretaceous of Israel. In *Geological Framework of the Levant, Volume II: the Levantine Basin and Israel*, 393–436 (Geological Survey of Israel, 2005).

[167] Sandler, A., Harlavan, Y. & Steinitz, G. Early formation of K-feldspar in shallow-marine sediments at near-surface temperatures (southern Israel): evidence from K-Ar dating. *Sedimentology* **51**, 323–338 (2004).

[168] Hall, J. K., Krasheninnikov, V. A., Hirsch, F., Benjamini, C. & Flexer, A. *Geological framework of the Levant, Volume II: the Levantine Basin and Israel* (Geological Survey of Israel, 2005).

[169] Hirsch, F., Rosenfeld, A. & Honigstein, A. Early Cretaceous ostracodes from the south-eastern slopes of Mount Hermon. *Current Research, Geological Survey of Israel* **9**, 90–91 (1994).

[170] Bachmann, M. & Hirsch, F. Lower Cretaceous carbonate platform of the eastern Levant (Galilee and the Golan Heights): stratigraphy and second-order sea-level change. *Cretaceous Research* **27**, 487–512 (2006).

[171] Ramajo, J., Aurell, M. & Cepria, J. Facies analysis of the Arroyofrío ferruginous oolitic Bed in Sierra de Arcos (Jurassic, northern Iberian Chain). *Journal of Iberian Geology* **28**, 45–64 (2002).

[172] Ramajo, J. *et al.* The Middle-upper Jurassic boundary oolitic ironstone level (Arroyofrío bed) in northeastern Iberian Range: a genetic and depositional model. In *28th IAS Meeting of Sedimentology*, vol. 359 (2011).

[173] Pueyo-Anchuela, Ó., Ramajo, J., Gil Imaz, A. & Meléndez Hevia, G. Analysis of anisotropy of magnetic susceptibility in iron-oolitic beds: a potential tool for paleocurrent identification. *International Journal of Earth Sciences* **102**, 1131–1149 (2013).

[174] Bonte, A. & Hatrival, J. N. Notice explicative de la feuille Rethel à 1/50000. *Carte Géologique de la France BRGM Orléans* **29**, 1–12 (1966).

[175] Courville, P., Bonnot, A., Dudicourt, J. C. & Cuif, G. L'assemblage à Cardioceras (Cardioceras) cordatum (J. Sowerby, 1812) et sa place au sein de la succession ammonitique de l'Oxfordien Inférieur (Jurassique Supérieur). Apport des données ardennaises (Province Subboréale). *Annales de Paléontologie* **97**, 9–33 (2011).

[176] Burkhalter, R. M. Die Passwang-Alloformation (unteres Aalénien bis unteres Bajocien) im zentralen und nördlichen Schweizer Jura. *Eclogae Geologicae Helvetiae* **89**, 875–934 (1996).

[177] Bläsi, H., Deplazes, G., Schellmann, M. & Traber, D. Sedimentologie und Stratigraphie des 'Braunen Doggers' und seiner westlichen äquivalente. *Nagra Arbeitsbericht, NAB* 12–51 (2013).

[178] Schunck, S., Rickli, J., Wohlwend, S., Weissert, H. & Vance, D. Continental weathering as the source of iron in Jurassic iron oolites from Switzerland. *Swiss Journal of Geosciences* **116**, 4 (2023).

[179] Wohlwend, S. *et al.* TBO Trüllikon-1-1: Data Report – Dossier IV: Microfacies, Bio- and Chemostratigraphic Analysis. *Nagra Arbeitsbericht, NAB* (2021).

[180] Wohlwend, S. *et al.* TBO Marthalen-1-1: Data Report – Dossier IV: Microfacies, Bio- and Chemostratigraphic Analysis. *Nagra Arbeitsbericht, NAB* (2021).

[181] Wohlwend, S. *et al.* TBO Bözberg-1-1: Data Report – Dossier IV: Microfacies, Bio- and Chemostratigraphic Analysis. *Nagra Arbeitsbericht, NAB* (2022).

[182] Vomvoris, S., Claudel, A., Blechschmidt, I. & Muller, H. The Swiss radioactive waste management program-brief history, status, and outlook. *Journal of Nuclear Fuel Cycle and Waste Technology* **1**, 9–27 (2013).

[183] Frei, A. *Die Mineralien des Eisenbergwerks Herznach im Lichte morphogenetischer Untersuchungen*. Ph.D. thesis, ETH Zurich (1952).

[184] Gehring, A. U. & Heller, F. Timing of natural remanent magnetization in ferriferous limestones from the Swiss Jura mountains. *Earth and planetary science letters* **93**, 261–272 (1989).

[185] Hallam, A. A review of the broad pattern of Jurassic sea-level changes and their possible causes in the light of current knowledge. *Palaeogeography, Palaeoclimatology, Palaeoecology* **167**, 23–37 (2001).

[186] Hesselbo, S. P., Robinson, S. A., Surlyk, F. & Piasecki, S. Terrestrial and marine extinction at the Triassic-Jurassic boundary synchronized with major carbon-cycle perturbation: a link to initiation of massive volcanism? *Geology* **30**, 251–254 (2002).

[187] Dietze, V., Kutz, M., Franz, M. & Bosch, K. Stratigraphy of the Kahlenberg near Ringsheim (Upper Rhine Valley, SW Germany) with emphasis on the Laeviuscula and Sauzei zones (Lower Bajocian, Middle Jurassic). *Palaeodiversity* **2**, 19–65 (2009).

[188] Dietze, V. *et al.* Die Humphriesianum-Zone (Unter-Bajocium, Mitteljura) am Kahlenberg bei Ringsheim (Oberrheingraben, SW Deutschland). *Palaeodiversity* **6**, 29–61 (2013).

[189] Rioult, M. Le stratotype du Bajocien. *Colloque du Jurassique Luxembourg 1962, Comptes Rendus et Mémoires* **3**, 239–258 (1964).

[190] Rioult, M. *et al.* Outcrop sequence stratigraphy of the Anglo-Paris Basin, Middle to Upper Jurassic (Normandy, Maine, Dorset). *Bulletin Centres de Recherches Exploration-Production Elf Aquitaine* **15**, 101–194 (1991).

[191] Préat, A., Mamet, B. L., De Ridder, C., Boulvain, F. & Gillan, D. Iron bacterial and fungal mats, Bajocian stratotype (Mid-Jurassic, Northern Normandy, France). *Sedimentary Geology* **137**, 107–126 (2000).

[192] Boulvain, F. *et al.* Les formations du Frasnien de la Belgique. *Memoirs of the Geological Survey of Belgium* **44**, 1–125 (1999).

[193] Denayer, J., Pacyna, D. & Frédéric, B. *Le minerai de fer en Wallonie: cartographie, historique et géologie* (Edition de la Région Wallonne, Belgium, 2011).

[194] Da Silva, A.-C. *et al.* Magnetic susceptibility as a high-resolution correlation tool and as a climatic proxy in Paleozoic rocks–merits and pitfalls: examples from the Devonian in Belgium. *Marine and Petroleum Geology* **46**, 173–189 (2013).

[195] Cotter, E. & Link, J. E. Deposition and diagenesis of Clinton ironstones (Silurian) in the Appalachian Foreland Basin of Pennsylvania. *Geological Society of America Bulletin* **105**, 911–922 (1993).

[196] Brett, C. E. *et al.* Early Silurian condensed intervals, ironstones, and sequence stratigraphy in the Appalachian foreland basin. *New York State Museum Bulletin* **491**, 89–143 (1998).

[197] McLaughlin, P. I., Emsbo, P. & Brett, C. E. Beyond black shales: the sedimentary and stable isotope records of oceanic anoxic events in a dominantly oxic basin (Silurian; Appalachian Basin, USA). *Palaeogeography, Palaeoclimatology, Palaeoecology* **367**, 153–177 (2012).

[198] Sullivan, N. B. & Brett, C. E. Integrating magnetic susceptibility data with sequence stratigraphy in the ironstone bearing successions (Lower Silurian) of eastern North America. *Stratigraphy* **10**, 261–280 (2013).

[199] Matheson, E. J. & Pufahl, P. K. Clinton ironstone revisited and implications for Silurian Earth system evolution. *Earth-Science Reviews* **215**, 103527 (2021).

[200] Holmer, L. E. Lower Viruan discontinuity surfaces in central Sweden. *Geologiska Föreningen i Stockholm Förhandlingar* **105**, 29–42 (1983).

[201] Jaanusson, V. The Viruan (Middle Ordovician) of Kinnekulle and northern Billingen, Västergötland. *Bulletin of the Geological Institutions of the University of Uppsala* **43**, 1–73 (1964).

[202] Lindskog, A., Eriksson, M. E., Bergström, S. M. & Young, S. A. Lower-Middle ordovician carbon and oxygen isotope chemostratigraphy at Hällekis, Sweden: implications for regional to global correlation and palaeoenvironmental development. *Lethaia* **52**, 204–219 (2019).

[203] Lindström, M. Sedimentary folds and the development of limestone in an Early Ordovician sea. *Sedimentology* **2**, 243–292 (1963).

[204] Orviku, K. On the lithostratigraphy of the Volkhov and Kunda stages in Estonia. *Transactions of the Institute of Geology, Academy of Sciences of the Estonian SSR* **5**, 45–87 (1960).

[205] Plado, J., Preeden, U., Pesonen, L., Mertanen, S. & Puura, V. Magnetic history of Early and Middle Ordovician sedimentary sequence, northern Estonia. *Geophysical Journal International* **180**, 147–157 (2010).

[206] Kirsimäe, K. & Jørgensen, P. Mineralogical and Rb-Sr isotope studies of low-temperature diagenesis of lower Cambrian clays of the Baltic paleobasin of North Estonia. *Clays and Clay Minerals* **48**, 95–105 (2000).

[207] Meidla, T., Ainsaar, L. & Hints, O. The Ordovician system in Estonia. In *4th Annual Meeting of IGCP 591, Estonia, 10–19 June 2014*, vol. 4 (2014).

[208] Männil, R., Kaljo, D. & Nestor, H. *Field Meeting Estonia 1990, an Excursion Guidebook* (Estonian Academy of Sciences Tallinn, 1990).

[209] Nielsen, A. T. Ordovician sea level changes: a Baltoscandian perspective. In *The Great Ordovician Biodiversification Event*, 84–93 (Columbia University Press, 2004).

[210] Sturesson, U. & Bauert, H. Origin and palaeogeographical distribution of the Viruan iron and phosphate ooids in Estonia: evidence from mineralogical and chemical compositions. *Sedimentary Geology* **93**, 51–72 (1994).

[211] Mägi, S. A characterization of the type section of the Ontikan Subseries. *Proceedings of the Academy of Sciences of the Estonian SSR* **33**, 104–112 (1984).

[212] Sturesson, U., Dronov, A. & Saadre, T. Lower Ordovician iron ooids and associated oolitic clays in Russia and Estonia: a clue to the origin of iron oolites? *Sedimentary Geology* **123**, 63–80 (1999).

[213] Männil, R. & Meidla, T. The Ordovician system of the East European Platform (Estonia, Latvia, Lithuania, Byelorussia, parts of Russia, the Ukraine and Moldova). *The Ordovician System of the East European Platform and Tuva (Southeastern Russia)* **28**, 1–52 (1994).

[214] Kříž, J. The Silurian of the Prague Basin (Bohemia)–tectonic, eustatic and volcanic controls on facies and faunal development. *Special Papers in Palaeontology* **44**, 179–203 (1991).

[215] Havlíček, V. Development of a linear sedimentary depression exemplified by the Prague Basin (Ordovician–Middle Devonian; Barrandian area–central Bohemia). *Sborník Geologickỳch Věd, Geologie* **35**, 7–48 (1981).

[216] Chlupáč, I. & Turek, V. *Palaeozoic of the Barrandian: Cambrian to Devonian* (Czech Geological Survey, 1998).

[217] Franke, W. The mid-European segment of the Variscides: tectonostratigraphic units, terrane boundaries and plate tectonic evolution. *Geological Society, London, Special Publications* **179**, 35–61 (2000).

[218] Žák, J. & Sláma, J. How far did the Cadomian 'terranes' travel from Gondwana during early Palaeozoic? A critical reappraisal based on detrital zircon geochronology. *International Geology Review* **60**, 319–338 (2018).

[219] Kraft, P. & Kraft, J. New dendroid graptolites from the Klabava Formation of the Bohemian Lower Ordovician. *Journal of the Czech Geological Society* **49**, 119–123 (2004).

[220] Petránek, J. & Van Houten, F. B. *Phanerozoic Ooidal Ironstones: Contribution to the International Geological Correlation Programme: Project 277-Phanerozoic Ooidal Ironstones* (Czech Geological Survey, 1997).

[221] Kraft, P. & Bruthansova, J. Preservation of fossils in the Sarka Formation (Darriwilian, Czech Republic). *Estonian Journal of Earth Sciences* **72**, 136–137 (2023).

[222] Kukal, Z. & Jager, O. Siliciclastic signal of the variscan orogenesis; Devonian Srbsko Formation of central Bohemia. *Věstník Ústředního ústavu Geologického* **63**, 65–80 (1988).

[223] Mergl, M. New brachiopods (Cambrian-Ordovician) from Algeria and Morocco (Mediterranean Province). *Časopis pro Mineralogii a Geologii* **28**, 337–348 (1983).

[224] Zicha, O., Bruthansová, J. & Kraft, P. Epibionts on shells in the šárka formation: a sparsely occupied niche in the lower to middle Darriwilian (Oretanian, Ordovician) in the Prague Basin (Czech Republic). *Palaeogeography, Palaeoclimatology, Palaeoecology* **550**, 109401 (2020).

[225] Mergl, M. New occurrences of the problematic fossil Berenicea vetera and associated stromatolic structures in the early Ordovician of the Prague Basin (Central Bohemia). *Zprávy o Geologickŷch Vŷzkumech* **46**, 180–184 (2013).

[226] Taylor, J. F., Myrow, P. M., Ripperdan, R. L., Loch, J. D. & Ethington, R. L. Paleoceanographic events and faunal crises recorded in the Upper Cambrian and Lower Ordovician of west Texas and southern New Mexico. In *GSA Field Guide 5: Field Trips in the Southern Rocky Mountains, USA*, 167–183 (Geological Society of America, 2004).

[227] Amato, J. M. & Mack, G. H. Detrital zircon geochronology from the Cambrian-Ordovician Bliss Sandstone, New Mexico: Evidence for contrasting Grenville-age and Cambrian sources on opposite sides of the Transcontinental Arch. *Geological Society of America Bulletin* **124**, 1826–1840 (2012).

[228] Dehler, C. M. *et al.* Neoproterozoic Chuar Group (800–742 Ma), Grand Canyon: a record of cyclic marine deposition during global cooling and supercontinent rifting. *Sedimentary Geology* **141**, 465–499 (2001).

[229] Dehler, C. *et al.* Synthesis of the 780–740 Ma Chuar, Uinta Mountain, and Pahrump (ChUMP) groups, western USA: implications for Laurentia-wide cratonic marine basins. *Geological Society of America Bulletin* **129**, 607–624 (2017).

[230] Johnston, D. T. *et al.* An emerging picture of Neoproterozoic ocean chemistry: insights from the Chuar Group, Grand Canyon, USA. *Earth and Planetary Science Letters* **290**, 64–73 (2010).

[231] Horodyski, R. J. & Bloeser, B. Possible eukaryotic algal filaments from the Late Proterozoic Chuar Group, Grand Canyon, Arizona. *Journal of Paleontology* **57**, 321–326 (1983).

[232] Ford, T. D. & Breed, W. J. Late Precambrian Chuar Group, Grand Canyon, Arizona. *Geological Society of America Bulletin* **84**, 1243–1260 (1973).

[233] Walcott, C. D. *Pre-Cambrian Igneous Rocks of the Unkar Terrane, Grand Canyon of the Colorado, Arizona* (United States Geological Survey, 1895).

[234] Dehler, C. M., Porter, S. M., Timmons, J. M. & Karlstrom, K. The Neoproterozoic Earth system revealed from the Chuar Group of Grand Canyon. *Grand Canyon Geology: Two Billion Years of Earth's History (Geological Society of America Special Paper)* **489**, 49–72 (2012).

[235] Porter, S. M. & Knoll, A. H. Testate amoebae in the Neoproterozoic Era: evidence from vase-shaped microfossils in the Chuar Group, Grand Canyon. *Paleobiology* **26**, 360–385 (2000).

[236] Porter, S. M. & Riedman, L. A. Systematics of organic-walled microfossils from the ca. 780–740 Ma Chuar Group, Grand Canyon, Arizona. *Journal of Paleontology* **90**, 815–853 (2016).

[237] Dehler, C. *et al.* Precise U-Pb age models refine Neoproterozoic western Laurentian rift initiation, correlation, and Earth system changes. *Precambrian Research* **396**, 107156 (2023).

[238] Rooney, A. D. *et al.* Coupled Re-Os and U-Pb geochronology of the Tonian Chuar Group, Grand Canyon. *Geological Society of America Bulletin* **130**, 1085–1098 (2018).

[239] Long, D. & Turner, E. *Formal Definition of the Neoproterozoic Mackenzie Mountains Supergroup (Northwest Territories), and Formal Stratigraphic Nomenclature for Terrigenous Clastic Units of the Katherine Group* (Geological Survey of Canada, 2013).

[240] Hume, G. S. & Link, T. A. *Canol Geological Investigations in the Mackenzie River Area, Northwest Territories and Yukon* (Geological Survey of Canada, 1945).

[241] Greenman, J. W., Rainbird, R. H. & Turner, E. C. High-resolution correlation between contrasting early Tonian carbonate successions in NW Canada highlights pronounced global carbon isotope variations. *Precambrian Research* **346**, 105816 (2020).

[242] Turner, E. C. Stratigraphy of the Mackenzie Mountains supergroup in the Wernecke Mountains, Yukon. In *Yukon Exploration and Geology*, 207–231 (Yukon Geological Survey, 2010).

[243] Greenman, J. & Rainbird, R. H. *Stratigraphy of the Upper Nelson Head, Aok, Grassy Bay, and Boot Inlet Formations in the Brock Inlier, Northwest Territories (NTS 97-A, D)* (Geological Survey of Canada, 2018).

[244] Milton, J. E., Hickey, K. A., Gleeson, S. A. & Friedman, R. M. New U-Pb constraints on the age of the Little Dal Basalts and Gunbarrel-related volcanism in Rodinia. *Precambrian Research* **296**, 168–180 (2017).

[245] Halverson, G. P., Hoffman, P. F., Schrag, D. P., Maloof, A. C. & Rice, A. H. N. Toward a Neoproterozoic composite carbon-isotope record. *Geological Society of America Bulletin* **117**, 1181–1207 (2005).

[246] Macdonald, F. A. *et al.* Calibrating the Cryogenian. *Science* **327**, 1241–1243 (2010).

[247] Jackson, M., Sweet, I. P. & Powell, T. G. Studies on petroleum geology and geochemistry, middle Proterozoic, McArthur Basin Northern Australia I: Petroleum potential. *Australian Petroleum Exploration Association* **28**, 283 (1988).

[248] Abbott, S. T. & Sweet, I. P. Tectonic control on third-order sequences in a siliciclastic ramp-style basin: An example from the Roper Superbasin (Mesoproterozoic), northern Australia. *Australian Journal of Earth Sciences* **47**, 637–657 (2000).

[249] Mui, M. D. *et al.* Proterozoic microfossils from the Roper Group, Northern Territory, Australia. *BMR Journal of Australian Geology & Geophysics* **3**, 17 (1978).

[250] Jackson, M., Muir, M. & Sweet, I. Sedimentology of the Middle Proterozoic McArthur Basin, Northern Australia–field excursion 13A. In *12th International Sedimentological Congress*, vol. 12 (1986).

[251] Ahmad, M. & Munson, T. *Geology and Mineral Resources of the Northern Territory* (Northern Territory Geological Survey, 2013).

[252] Kralik, M. Rb-Sb age determinations on precambrian carbonate rocks of the Carpentarian McArthur Basin, Northern Territories, Australia. *Precambrian Research* **18**, 157–170 (1982).

[253] Yang, B. *et al.* Spatial and temporal variation in detrital zircon age provenance of the hydrocarbon-bearing upper Roper Group, Beetaloo sub-basin, Northern Territory, Australia. *Precambrian Research* **304**, 140–155 (2018).

[254] Kendall, B., Creaser, R. A., Gordon, G. W. & Anbar, A. D. Re–Os and Mo isotope systematics of black shales from the Middle Proterozoic Velkerri and Wollogorang formations, McArthur Basin, northern Australia. *Geochimica et Cosmochimica Acta* **73**, 2534–2558 (2009).

[255] Johnson, B. W. & Wing, B. A. Limited Archaean continental emergence reflected in an early Archaean ^{18}O-enriched ocean. *Nature Geoscience* **13**, 243–248 (2020).

[256] Planavsky, N. J. *et al.* Low mid-Proterozoic atmospheric oxygen levels and the delayed rise of animals. *Science* **346**, 635–638 (2014).

[257] Chu, X., Zhang, T., Zhang, Q. & Lyons, T. W. Sulfur and carbon isotope records from 1700 to 800 Ma carbonates of the Jixian section, northern China: implications for secular isotope variations in Proterozoic seawater and relationships to global supercontinental events. *Geochimica et Cosmochimica Acta* **71**, 4668–4692 (2007).

[258] Zhang, S. *et al.* Pre-Rodinia supercontinent Nuna shaping up: a global synthesis with new paleomagnetic results from North China. *Earth and Planetary Science Letters* **353**, 145–155 (2012).

[259] Planavsky, N. *et al.* Iron-oxidizing microbial ecosystems thrived in late paleoproterozoic redox-stratified oceans. *Earth and Planetary Science Letters* **286**, 230–242 (2009).

[260] Songnian, L. & Huimin, L. A precise U-Pb single zircon age determination for the volcanics of Dahongyu Formation, Changcheng System in Jixian. *Acta Geoscientica Sinica* **22**, 137–145 (1991).

[261] Gao, L. Z. *et al.* SHRIMP zircon ages: Basis for refining the chronostratigraphic classification of the Meso-and Neoproterozoic strata in North China old land. *Acta Geoscientica Sinica* **29**, 366–376 (2008).

[262] Li, H. K. *et al.* The base age of the Changchengian system at the northern North China Craton should be younger than 1670 Ma: constraints from zircon U–Pb LA-MC-ICPMS dating of a granite-porphyry dike in Miyun County, Beijing. *Earth Science Frontiers* **18**, 108–120 (2011).

[263] Peng, P., Liu, F., Zhai, M. & Guo, J. Age of the Miyun dyke swarm: Constraints on the maximum depositional age of the Changcheng System. *Chinese Science Bulletin* **57**, 105–110 (2012).

[264] Zhang, Y.-B. *et al.* Diagenetic xenotime dating to constrain the initial depositional time of the Yan-Liao Rift. *Precambrian Research* **271**, 20–32 (2015).

[265] Duan, C. *et al.* U-Pb ages and Hf isotopes of detrital zircon grains from the Mesoproterozoic Chuanlinggou Formation in North China Craton: implications for the geochronology of sedimentary iron deposits and crustal evolution. *Minerals* **8**, 547 (2018).

[266] Gao, L. *et al.* Recognition of Meso-and Neoproterozoic stratigraphic framework in North and South China. *Acta Geoscientica Sinica* **30**, 433–446 (2009).

[267] Zhang, S., Zhao, Y., Ye, H., Hu, J.-M. & Wu, F. New constraints on ages of the Chuanlinggou and Tuanshanzi formations of the Changcheng System in the Yan-Liao area in the northern North China Craton. *Acta Petrologica Sinica* **29**, 2481–2490 (2013).

[268] van Hinsbergen, D. J. J. *et al.* A paleolatitude calculator for paleoclimate studies. *PLoS ONE* **10**, e0126946 (2015).

[269] Park, J. K. & Aitken, J. D. Paleomagnetism of the Katherine Group in the Mackenzie Mountains: implications for post-Grenville (Hadrynian) apparent polar wander. *Canadian Journal of Earth Sciences* **23**, 308–323 (1986).

[270] MacLennan, S. A. *et al.* Geologic evidence for an icehouse Earth before the Sturtian global glaciation. *Science Advances* **6**, eaay6647 (2020).

[271] Krs, M., Krsová, M., Pruner, P., Chvojka, R. & Havlíček, V. Palaeomagnetism, palaeogeography and the multicomponent analysis of Middle and Upper Cambrian rocks of the Barrandian in the Bohemian Massif. *Tectonophysics* **139**, 1–20 (1987).

[272] Wilde, P., Barnes, C. & Williams, S. Oceanography in the Ordovician. *Advances in Ordovician Geology* **90**, 283–298 (1991).

[273] Hansell, D. A. *et al.* Compilation of dissolved organic matter (DOM) data obtained from the global ocean surveys from 1994 to 2020 (NCEI Accession 0227166). Tech. Rep., NOAA National Centers for Environmental Information (2021).

[274] Gomez-Saez, G. V. *et al.* Molecular evidence for abiotic sulfurization of dissolved organic matter in marine shallow hydrothermal systems. *Geochimica et Cosmochimica Acta* **190**, 35–52 (2016).

[275] Gomez-Saez, G. V. *et al.* Sulfurization of dissolved organic matter in the anoxic water column of the Black Sea. *Science Advances* **7**, eabf6199 (2021).

[276] Poulton, S. W. & Canfield, D. E. Ferruginous conditions: a dominant feature of the ocean through Earth's history. *Elements* **7**, 107–112 (2011).

[277] Garcia, A. K., Cavanaugh, C. M. & Kacar, B. The curious consistency of carbon biosignatures over billions of years of Earth-life coevolution. *The ISME Journal* **15**, 2183–2194 (2021).

[278] Knoll, A. H. & Carroll, S. B. Early animal evolution: emerging views from comparative biology and geology. *Science* **284**, 2129–37 (1999).

[279] Pires, N. D. & Dolan, L. Morphological evolution in land plants: new designs with old genes. *Philosophical Transactions of the Royal Society B: Biological Sciences* **367**, 508–518 (2012).

[280] Nguyen, K. *et al.* Absence of biomarker evidence for early eukaryotic life from the Mesoproterozoic Roper Group: searching across a marine redox gradient in mid-Proterozoic habitability. *Geobiology* **17**, 247–260 (2019).

[281] Javaux, E. J. Challenges in evidencing the earliest traces of life. *Nature* **572**, 451–460 (2019).

[282] Bhattacharyya, D. P. & Kakimoto, P. K. Origin of ferriferous ooids; an SEM study of ironstone ooids and bauxite pisoids. *Journal of Sedimentary Research* **52**, 849–857 (1982).

[283] Boso, M. A. & Monaldi, C. R. Oolitic stratabound iron ores in the Silurian of Argentina and Bolivia. In *Stratabound Ore Deposits in the Andes*, 175–186 (Springer, 1990).

[284] Oyarzún, J. M. The metalliferous ore deposits of Chile and Argentina, and their geologic framework. In *Stratabound Ore Deposits in the Andes*, 61–78 (Springer, 1990).

[285] Cotter, E. Diagenetic alteration of chamositic clay minerals to ferric oxide in oolitic ironstone. *Journal of Sedimentary Research* **62**, 54–60 (1992).

[286] Lempart, M., Derkowski, A., Luberda-Durnaś, K., Skiba, M. & Błachowski, A. Dehydrogenation and dehydroxylation as drivers of the thermal decomposition of Fe-chlorites. *American Mineralogist* **103**, 1837–1850 (2018).

[287] Korenaga, J. Archean geodynamics and the thermal evolution of Earth. *Archean Geodynamics and Environments Geophysical Monograph Series* **164**, 7–32 (2006).

[288] Korenaga, J. Crustal evolution and mantle dynamics through Earth history. *Philosophical Transactions of the Royal Society A: Mathematical, Physical and Engineering Sciences* **376**, 20170408 (2018).

[289] Fischer, W. W., Hemp, J. & Johnson, J. E. Evolution of oxygenic photosynthesis. *Annual Review of Earth and Planetary Sciences* **44**, 647–683 (2016).

[290] Wilkes, E. B. & Pearson, A. A general model for carbon isotopes in red-lineage phytoplankton: interplay between unidirectional processes and fractionation by RuBisCO. *Geochimica et Cosmochimica Acta* **265**, 163–181 (2019).
